# Attractive Ni^…^O Interactions Enable Non‐Alternating Ethylene‐Carbon Monoxide Copolymerization

**DOI:** 10.1002/anie.6761777

**Published:** 2026-06-30

**Authors:** Yajun Zhao, Michele Tomasini, Inigo Göttker‐Schnetmann, Laura Falivene, Lucia Caporaso, Stefan Mecking

**Affiliations:** ^1^ Chair of Chemical Materials Science Department of Chemistry University of Konstanz Konstanz Germany; ^2^ Department of Chemistry University of Salerno Salerno Italy

**Keywords:** nickel catalyst, polyethylene, polymer chemistry, polymer science, polymerization catalysts

## Abstract

In‐chain functional groups can reduce the environmental impact of polyethylene waste and enhance its recyclability. A significant advance was the realization of the long‐sought catalytic copolymerization of carbon monoxide with ethylene in a non‐alternating manner, providing keto‐polyethylene materials with low densities of photodegradable keto groups in the chain. Despite this breakthrough, the state‐of‐the‐art catalysts’ limited carbon monoxide tolerance is a hurdle in further developing more sustainable polyethylenes. Here we show how these fundamental issues can be addressed by implementation of attractive Ni···O interactions in novel as well as state‐of‐the‐art neutral nickel catalyst motifs. Incorporation of *P*‐bound 2,6‐diphenoxyphenyl moieties into both phosphine‐imidate and phosphine‐phenolate ligand frameworks provides highly active and robust catalysts that generate keto‐polyethylenes not accessible to date. Theoretical calculations reveal that Ni···O interactions lower *cis/trans* isomerization barriers of coordinated ethylene, thereby driving ethylene insertion along the desired non‐alternating pathway. At the same time, steric constraint raises the energy barriers for carbon monoxide insertion and reductive elimination, effectively suppressing undesired extensive carbon monoxide insertion and catalyst deactivation. The concept uncovered enables operating conditions, productivities and in‐chain functional group concentrations not possible with existing catalysts, and provides perspectives for putting much needed environmentally benign polyolefins into practice.

## Graphical abstract

Attractive Ni···O interactions are established in phosphine‐imidated and phosphine‐phenolate nickel catalysts, enabling non‐alternating ethylene‐carbon monoxide copolymerization, resulting in superior CO tolerance, productivity and stability compared to the state‐of‐the‐art benchmark. The interactions drive the desired non‐alternating pathway via lowering *cis/trans* isomerization barriers of coordinated ethylene. This concept uncovered advancing catalytic access to in‐chain functionalized polyethylenes and much needed environmentally benign plastics.

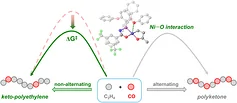

## 1 Introduction

Carbon monoxide (CO) is widely used as a building block in the large‐scale synthesis of small molecular carbonyl compounds. Typically, d^8^ metal catalysts are employed [1, 2]. Electron‐deficient d^0^ metal catalysts, such as those used in traditional olefin polymerization processes, are deactivated by CO due to their extreme oxophilicity [3]. As a comonomer in olefin co‐polymerizations, carbon monoxide affords in‐chain carbonyl groups, a unique feature compared to the side chain functional groups arising from polar vinyl comonomers [4]. The terpolymerization of ethylene and propylene with CO yields alternating polyketones [CH_2_CH(R)C(=O)]*_n_*, which are applied as high melting (*T*_m_ ca. 220° C) engineering thermoplastics [5]. Their strictly alternating nature arises from the strong preference of the used cationic palladium catalysts for carbon monoxide incorporation, due to its much preferred binding compared to ethylene in combination with facile insertion [6, 7]. A deviation from a strictly alternating microstructure was first observed with neutral phosphine‐sulfonate palladium catalysts [8, 9], however, the brittle polymers obtained suffered from low molecular weights (*M*_n_ < 3 kg/mol) and/or high carbon monoxide incorporations (> 40 mol%) [10]. Keto‐polyethylene materials were enabled by the use of advanced nickel catalysts in combination with gas feed systems for controlled high ethylene / CO ratios [11]. This affords high molecular weight ductile keto‐polyethylenes that are on par with commercial HDPE regarding their processing and tensile properties. At the same time, the targeted keto incorporations of ca. 1 mol% enable photodegradation. This may alleviate the environmental persistency of polyethylene waste and litter [12, 13, 14, 15]. As an elegant alternative to provide low CO concentrations, metal carbonyls can be employed as demonstrated by the generation of polyethylenes with isolated keto units with customized neutral palladium catalysts [16, 17].

**FIGURE 1:**
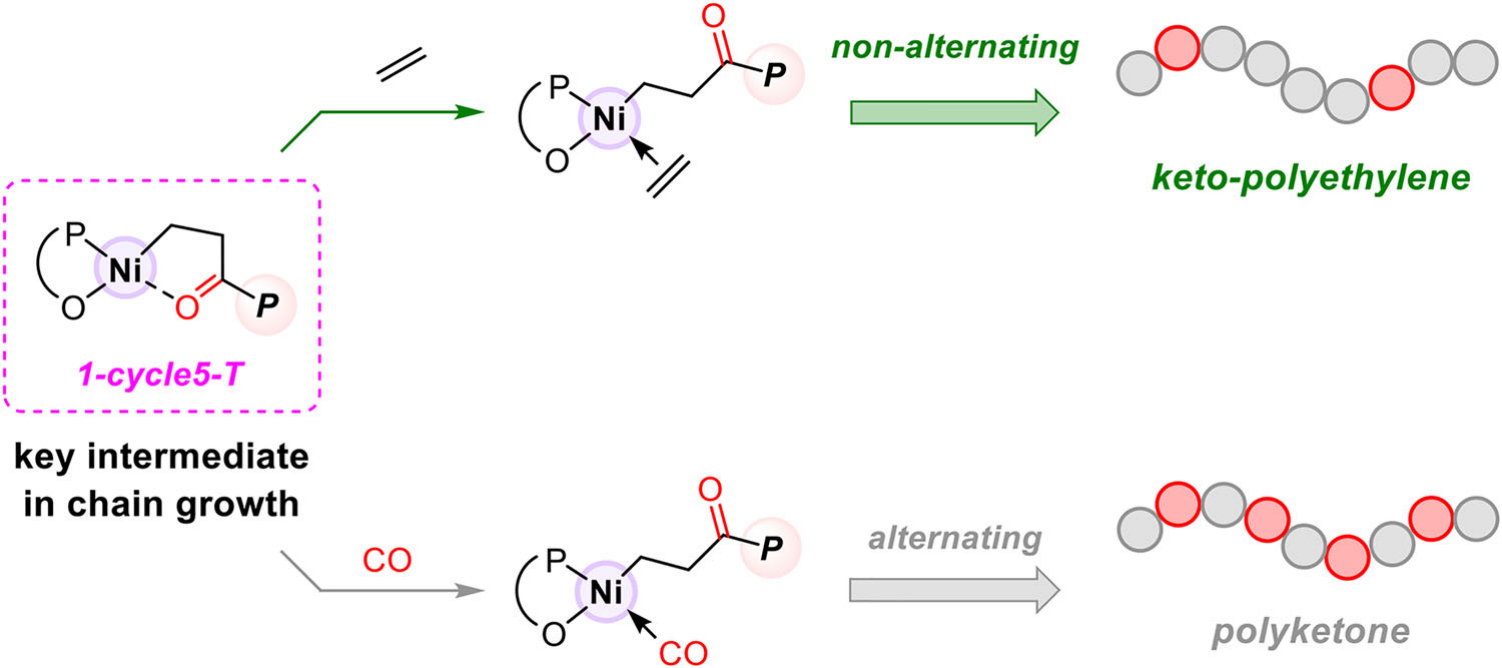
Alternating and non‐alternating pathways of ethylene‐CO copolymerization from the key intermediate (chelated **1‐cycle5‐T**).

Despite these breakthroughs, the catalysts’ limited CO tolerance poses a major hurdle in further developing these materials. At this point, the polymerization to keto‐polyethylenes is practically limited to low CO feeds (ca. ≤ 1 mol%) and ketone incorporations of ca. 2 mol%, beyond this polymer productivities become low and undesirable alternating microstructures prevail. In depth theoretical and experimental studies have elucidated the mechanistic origin of this unique reactions’ challenges [18]. A five‐membered chelate formed after CO incorporation and a subsequent ethylene incorporation into the growing chain is a decisive key intermediate (Figure 1). Further chain growth requires opening of this chelate by monomer binding, and can occur either along the desired non‐alternating pathway through incorporation of an ethylene monomer, or along the non‐desirable alternating pathway through another CO incorporation. The preference between these pathways is determined by the nature and the barriers of the rate limiting steps, which depend on the catalyst structure. In addition, a CO‐specific irreversible catalyst deactivation pathway was identified that arises from the electrophilic nature of the Ni‐acyls formed upon incorporation of CO into the growing chain [19].

Recent studies of olefin polymerization by different neutral Ni(II) catalysts have elucidated that weak non‐covalent interactions in the catalyst framework can significantly impact the energies of intermediates and transition states of olefin polymerizations [20, 21, 22]. Oxygen‐donor substituents positioned proximal to the nickel center can establish distinctive Ni···O interactions that dramatically influence catalytic selectivity and polymer microstructures, as observed for ethylene homopolymerization reactions (with a neutral salicylaldiminato‐Ni(II) catalyst, not compatible with CO co‐polymerizations) [22]. Motivated by this picture we explored the impact of appropriately placed oxygen‐donor substituents in P,O‐coordinating ligands on the outlined challenges in Ni(II)‐catalyzed carbon monoxide copolymerization.

## 2 Results and Discussion

### 2.1 Phosphine‐Imidate Ni(II) Catalysts

**FIGURE 2:**
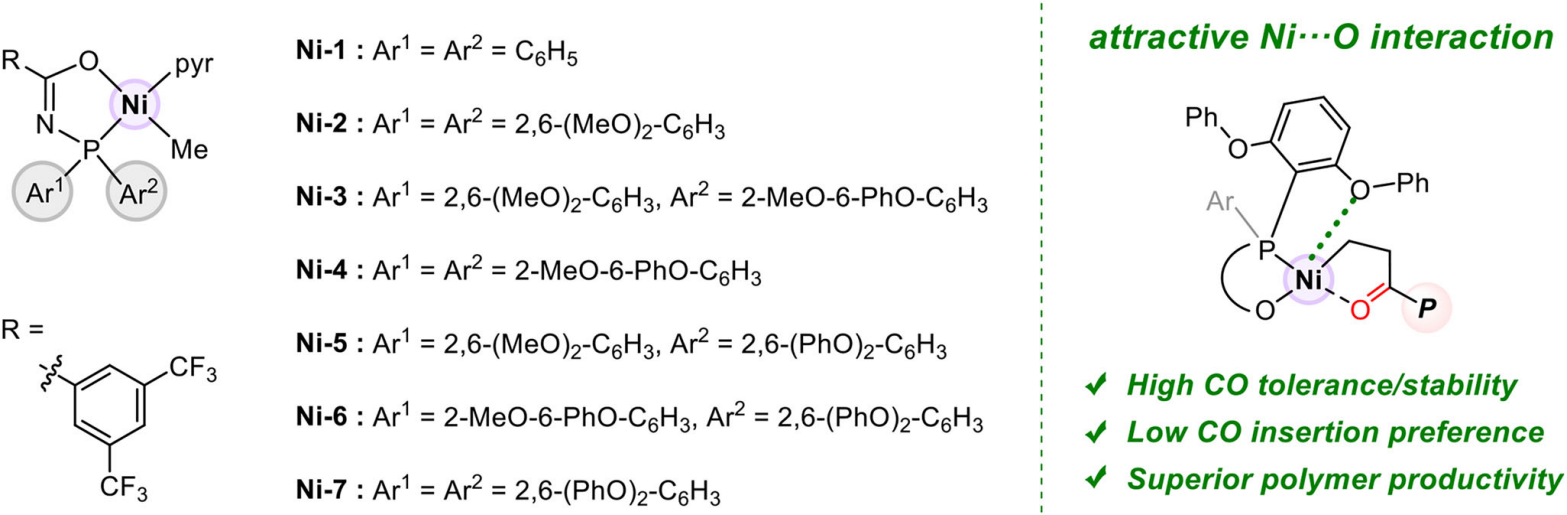
This work: Phosphine‐imidate Ni(II) precatalysts design by leveraging *P*‐bound moieties to implant Ni···O interactions. Me, methyl group; Ph, phenyl group; pyr, pyridine.

As a backbone structure phosphine‐imidates were explored, this choice was guided by the tolerance toward polar groups of such Ni(II)‐catalysts in ethylene copolymerization with acrylates [23]. Exposure of the known reference complex **Ni‐1** to carbon monoxide / ethylene (CO / E) mixtures resulted in the formation of only a small amount of low‐molecular‐weight oligomers (*M*_n_ < 1 kg/mol), even at CO concentrations below 1 mol%. High‐temperature nuclear magnetic resonance (NMR) spectroscopy revealed olefinic end groups, evidencing chain transfer by ß‐hydride elimination (Figures S25 and S26) [24]. A series of neutral phosphine‐imidate Ni(II) precatalysts with variable alkyl‐ and aryloxy substitution in their *P*‐bound aryl moieties (Figure 2) were accessed by reaction of the corresponding phosphine‐amides with [(tmeda)NiMe_2_] (tmeda = *N,N,N’,N’*‐tetramethyl ethylene diamine) in excess pyridine (cf. Supporting Information for details of synthetic procedures and characterization). Single‐crystal x‐ray diffraction (scXRD) analyses of complexes **Ni‐2** and **Ni‐7** confirmed that the closest distance between the nickel center and ortho‐oxygen atoms of *P*‐bound aryls are shorter than the sum of van der Waals radii [25], suggesting the presence of attractive Ni···O interactions (Figures S22 and S23). These indications from the solid‐state structures of the precatalysts indeed corroborate theoretical studies of the key transition states and intermediates (vide infra).

**FIGURE 3:**
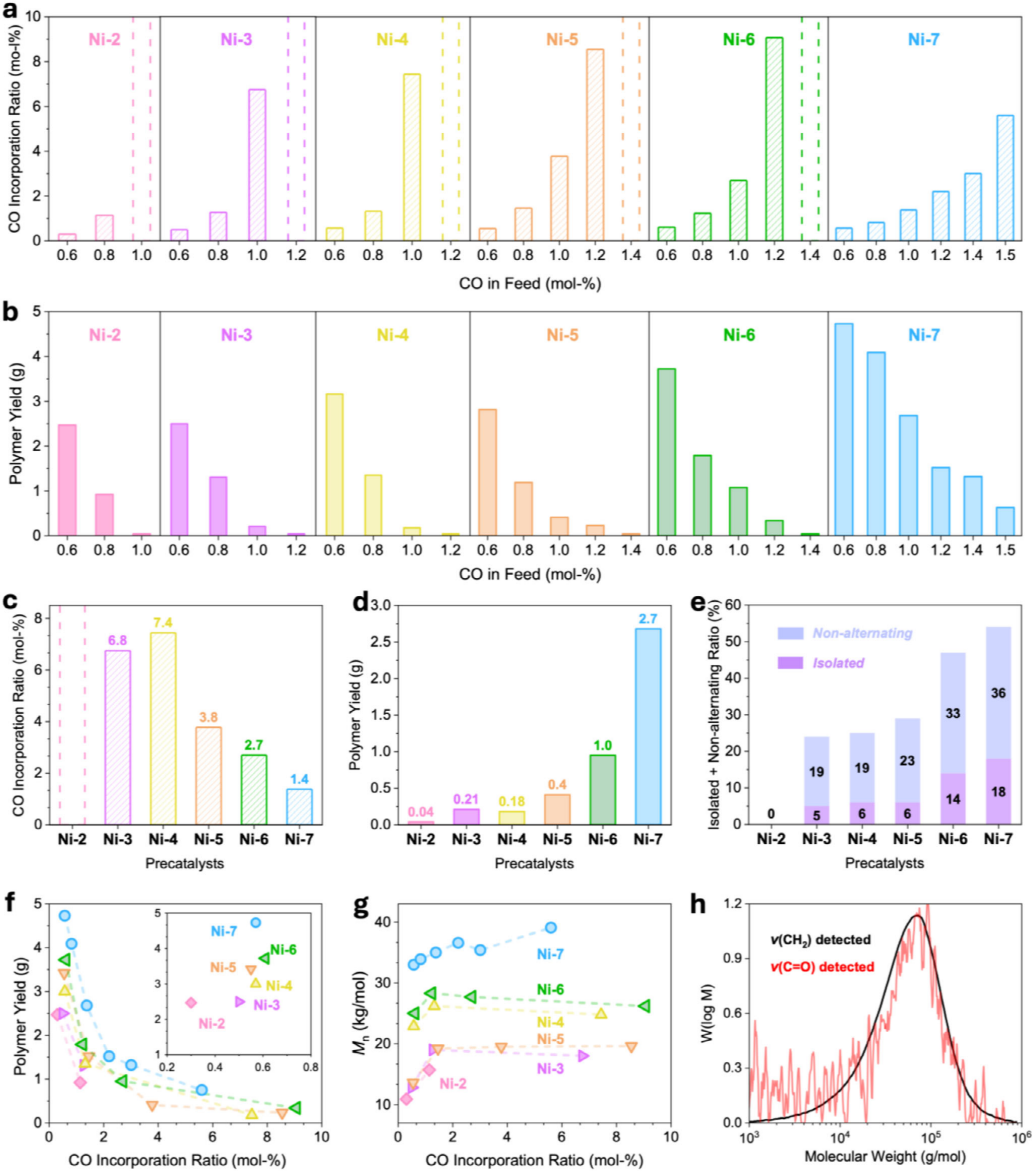
Ethylene‐CO co‐polymerization by **Ni‐2** to **Ni‐7**. (a) CO incorporation ratios, determined by NMR spectroscopy, as a function of CO concentration in the feed. (b) Polymer yields as a function of CO feed concentration. (c‐e), Comparison of catalytic performances under identical conditions (1.0 mol% CO in feed). (c) CO incorporation ratios, and (d) polymer yields for different precatalysts. (e) Relative fraction of isolated and non‐alternating ketone motifs derived from NMR spectroscopy. (f) Polymer yields, and (g) molecular weight (*M*_n_) of copolymers as function of CO incorporation ratios. (h) SEC traces of copolymer catalyzed by **Ni‐7** (5.6 mol% ketone repeat units, Table S4, entry 29) with CH_2_ sensitive IR detection (black) and C=O sensitive detection (red). Blank columns in (a) and (c) indicate catalyst deactivation and/or formation of trace amounts of alternating polyketone. All reactions were carried out with 5 µmol precatalyst loading, 10 bar, 100 mL toluene, 100 °C, 75 min, unless specified otherwise (cf. Table S4).

The catalysts' behavior toward CO / E mixtures was studied in a custom pressure reactor setup, which is capable of reliably dosing small amounts of CO feed into an excess ethylene feed, and automatically replenishes monomers to the reactor when monomer consumption results in a pressure decrease to a certain threshold [26]. Exposure of **Ni‐2** through **Ni‐7** to variable CO / E ratios (Table S4) resulted in the formation of keto‐polyethylenes, with keto contents increasing (Figure 3a) and polymer yields decreasing (Figure 3b) with increasing CO feed concentration, as expected. However, dramatic differences are observed between the catalysts. With **Ni‐2**, already at a CO feed of 1 mol% only traces of polymer are formed. The shift of the IR carbonyl absorption from 1714 cm^−1^ observed for keto‐polyethylenes formed at lower CO feeds to 1695 cm^−1^ is indicative of the formation of alternating polyketone motifs (Figure S125) [10, 27]. Prominently, **Ni‐7**, featuring *P*‐bound 2,6‐diphenoxyphenyl moieties exclusively, showed superior CO tolerance across a wide operational concentration range (up to 1.5 mol% CO in feed, and polymer ketone contents up to 5.6 mol%). Mass flow monitoring of the polymerization experiments revealed that **Ni‐7** displayed a high stability under reaction conditions, with frequent repressurization events indicating rapid and sustained monomer consumption (Figures S145–S150).

Considering the role of the ortho‐substituents, comparison of polymerizations at a given CO feed (1 mol%) shows that the ketone incorporation in the polymer chains decreased progressively for precatalysts **Ni‐2** to **Ni‐7** (Figure 3c). This reveals bulky phenoxy groups reduce the preference for CO incorporation. At the same time, polymer yields increase strongly. For example, **Ni‐7** afforded an order of magnitude higher yield than **Ni‐3** (2.7 g vs. 0.21 g, Figure 3d). This general trend is not surprising, as with decreasing ketone incorporation less chelate opening events (Figure 1) that slow down chain growth occur for a given mass of chain. Remarkably, however, also for a given CO incorporation, productivities increase strongly with the portion of phenoxy substituents, **Ni‐7** standing out in its capability to grow keto‐polyethylene chains (Figure 3f). Comparison of **Ni‐4** and **Ni‐5**, which both bear two methoxy and phenoxy substituents in the *P*‐bound moieties, shows that for this case also the substitution pattern significantly impacts catalytic properties. **Ni‐4**, with a symmetric‐substituted phosphine moiety, afforded much higher CO incorporations (7.4 mol% vs. 3.8 mol%) versus **Ni‐5** under otherwise identical conditions. This further hints toward the spatial arrangement of steric hindrance around the nickel center affecting CO incorporation preference and catalyst productivity (vide infra).

Note the keto‐polyethylene generated by **Ni‐7** possesses a desirable microstructure of predominantly isolated and non‐alternating keto groups in the polyethylene chain (Figure 3e). Beneficially, polymer molecular weights also increase from **Ni‐2** through **Ni‐7**, and **Ni‐7** produced high molecular weight keto‐polyethylenes consistently across all CO feeds probed and for all polymers formed (Figure 3g). High‐temperature size exclusion chromatography (SEC) with composition‐sensitive detection revealed a uniform ketone distribution throughout all molecular weight fractions (Figure 3h). This also underlines the well‐behaved nature of the catalytic polymerization reaction. Wide‐angle x‐ray scattering (WAXS) analysis revealed diffraction patterns consistent with commercial HDPE, confirming the ketone moieties do not disturb polyethylene crystallinity and are incorporated in the crystalline lamellae (Figure S126).

### 2.2 Probing of Concept in State‐of‐the‐Art Catalysts

**FIGURE 4:**
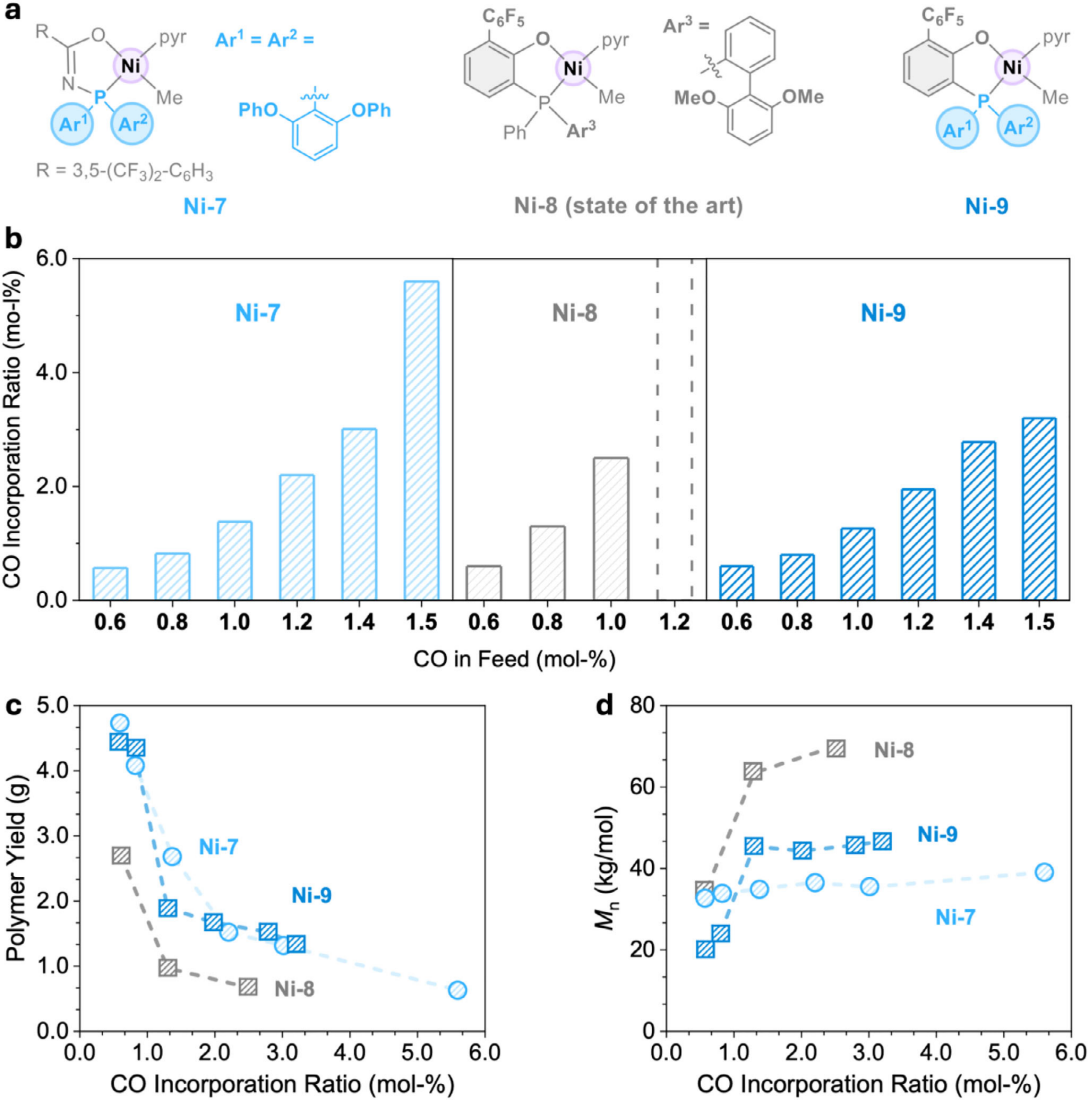
Probing of concept versus the state‐of‐the‐art catalyst. (a) Structures of phosphine‐phenolate **Ni‐8** and **Ni‐9**. (b) CO incorporation ratios, determined by NMR spectroscopy, as a function of CO concentration in the feed. Blank columns in (b) indicate catalyst deactivation and/or formation of trace amounts of alternating polyketone. (c) Plot of polymer yield, (d) molecular weights (*M*_n_) of copolymers against CO incorporation ratio. All reactions were carried out with 5 µmol precatalyst loading, 10 bar, 100 mL toluene, 100° C, 75 min, unless specified otherwise (cf. Table S4). Me, methyl group; Ph, phenyl group; pyr, pyridine.

Encouraged by the above findings, the underlying concept was probed for the phosphine‐phenolate ligand backbone of **Ni‐8**, this being the best‐performing system reported to date for non‐alternating copolymerization to produce keto‐polyethylenes (Figure 4a) [11, 28, 29]. Catalyst **Ni‐9** with *P*‐bound 2,6‐diphenoxyphenyl moieties features a distinctly different and superior behavior compared to the state‐of‐the‐art benchmark (cf. Supporting Information for details of catalyst synthesis and characterization).

**Ni‐9** operated effectively across a broad CO feed range (up to 1.5 mol%), comparable to **Ni‐7**, and has a significantly lower preference for CO incorporation than **Ni‐8** under identical conditions (Figure 4b). Notably, **Ni‐7** and **Ni‐9** demonstrated substantially enhanced productivities compared to **Ni‐8**, also for a given keto‐polyethylene composition (Figure 4c). All three catalysts produce high molecular weight keto‐polyethylenes under the conditions studied (Figure 4d). The presence of CO significantly increased polymer molecular weights for all catalysts, consistent with previous observations (Figure 4d) that the presence of CO reduces ß‐hyride elimination [11].

### 2.3 Mechanistic Analysis by Theoretical Methods

**FIGURE 5:**
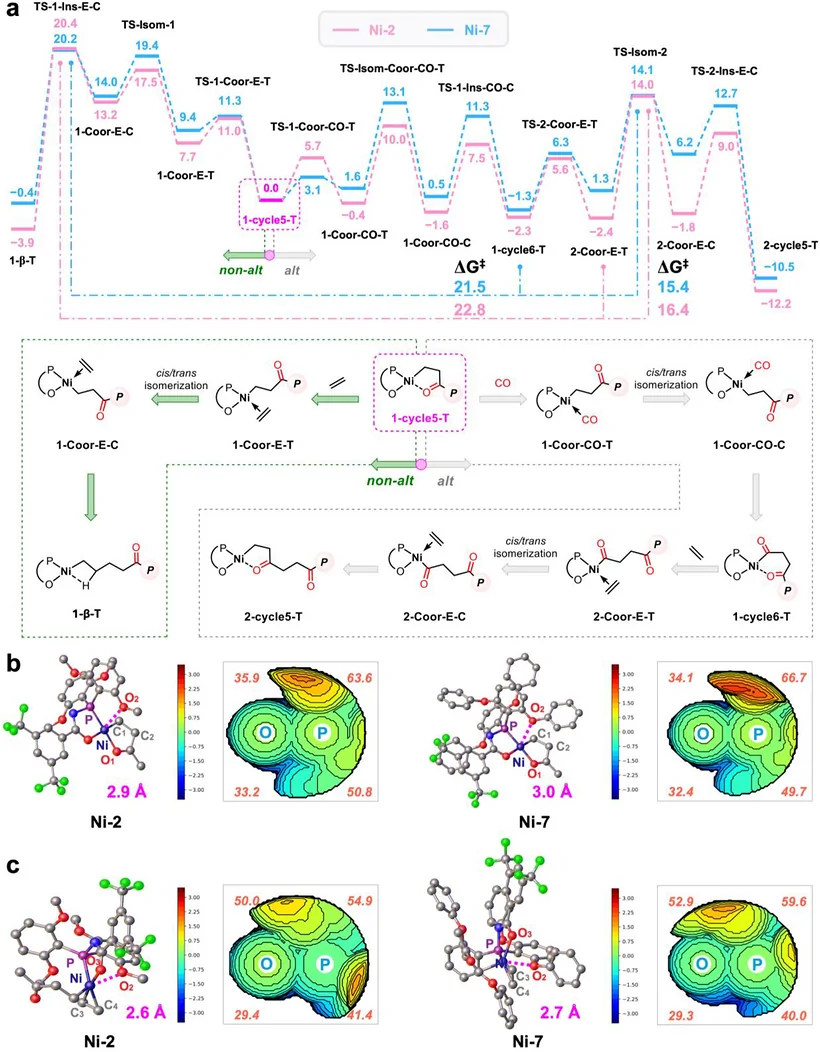
DFT studies of ethylene‐CO co‐polymerization pathways. (a) Free Gibbs energies (Δ*G*_tol_ in kcal/mol) of the key steps for non‐alternating and alternating CO incorporation with precatalysts **Ni‐2** (pink) and **Ni‐7** (blue). The labels 1‐… refer to species involved in monomer incorporation (ethylene or CO) from chelated **1‐cycle5‐T** intermediates and the 2‐… for the next (second) monomer (ethylene + CO) incorporation. (b) Geometries analysis and topographic steric maps of chelated **1‐cycle5‐T** intermediates. (c) Geometries analysis and topographic steric maps of transition states **TS‐Isom‐1**.

The mechanistic origin of the observed catalyst performance was elucidated by density functional theory (DFT) studies of the phosphine‐imidate complexes **Ni‐2** and **Ni‐7**, and the phosphine‐phenolate **Ni‐9** as well as the benchmark system **Ni‐8** (Figures 5a and S162). Following established protocols [18, 26], the **1‐cycle5‐T** chelate intermediate was identified as the common starting point for the competitive non‐alternating and alternating pathways, and was defined as the zero‐point energy reference. Structural analysis of **1‐cycle5‐T** in **Ni‐2** and **Ni‐7** unveiled distinctive stabilizing Ni···O interactions (2.9 and 3.0 Å) between the nickel center and the oxygen atoms of the *P*‐bound moieties, with distances significantly shorter than the sum of their van der Waals radii (Figure 5b) [25]. Quantum Theory of Atoms in Molecules (QTAIM) analysis of these contacts [30] in the relevant structures reveals the presence of a bond critical point between the Ni center and the oxygen atom, and non‐covalent interaction (NCI) plots [31] show an attractive interaction region in the same area, characterized by a green isosurface typically associated with weak van der Waals‐type interactions (Figures S166–S171).

### Non‐Alternating Pathway

Ethylene coordination to **1‐cycle5‐T** proceeds via **TS‐1‐Coor‐E‐T** to yield the **1‐Coor‐E‐T** intermediate. The subsequent *cis/trans* isomerization represents the key step of the non‐alternating pathway that governs its competition with the undesired alternating pathway (vide infra). In fact, for the benchmark **Ni‐8**, this step is the primary bottleneck (24.5 kcal/mol), which penalizes the non‐alternating route. In contrast, **Ni‐2** and **Ni‐7** exhibit substantially lower isomerization barriers (17.5 and 19.4 kcal/mol, respectively). Geometric analysis reveals this facilitation stems from the stabilizing Ni···O interactions (2.6 and 2.7 Å), which allow the transition state to access a stable square‐pyramidal geometry (Figure 5c) [18].

The 2,6‐diphenoxyphenyl‐substituted phosphine‐phenolate catalyst **Ni‐9** is leveraged by the same principle, achieving a 6.1 kcal/mol reduction in the isomerization barrier relative to **Ni‐8** (**TS‐Isom‐1** at 18.4 kcal/mol, Figure S162). Notably, all attempts to enforce oxygen coordination in **Ni‐8** were unsuccessful, as the system lacks the necessary architectural framework, and preferentially adopts a strained tetrahedral transition state. In the oxygen‐stabilized catalysts, the significant lowering of the **TS‐Isom‐1** barrier reconfigures the energy landscape of competing pathways: Isomerization is no longer the rate‐limiting step, and the subsequent ethylene insertion (**TS‐1‐Ins‐E‐C**) emerges as the new rate‐limiting event along this path, with barriers centered around 20 kcal/mol across the series of catalysts.

### Alternating Pathway

CO coordination to **1‐cycle5‐T** proceeds through transition state **TS‐1‐Coor‐CO‐T** to form the **1‐Coor‐CO‐T** intermediate, at ‐0.4 and 1.6 kcal/mol for **Ni‐2** and **Ni‐7**, respectively. Following *cis/trans* isomerization via **TS‐Isom‐Coor‐CO‐T** (cf. Supporting Information for details), CO insertion occurs through **TS‐1‐Ins‐CO‐C** with barriers of 7.5 and 11.3 kcal/mol, leading to the formation of the stable **1‐cycle6‐T** chelate. The 3.8 kcal/mol higher CO insertion barrier for **Ni‐7** likely arises from increased steric hindrance during migratory insertion, as the bulky phenoxy moieties restrict the approach geometry.

Subsequent ethylene coordination occurs through the opening of the **1‐cycle6‐T** chelate to form the **2‐Coor‐E‐T** intermediate. A comparative analysis reveals significant thermodynamic differences between **Ni‐2** and **Ni‐7**, which rationalize their different catalytic activities. For **Ni‐2**, **1‐cycle6‐T** (at −2.3 kcal/mol) and **2‐Coor‐E‐T** (at −2.4 kcal/mol) represent the global energy minima. This stabilization traps the catalyst along the alternating pathway and provides a starting point for the deactivation mechanism via reductive elimination (vide infra). In contrast, for **Ni‐7**, these intermediates are notably less stable: **1‐cycle6‐T** sits at −1.3 kcal/mol, and **2‐Coor‐E‐T** at 1.3 kcal/mol. This upward shift in energy contributes to lowering the overall activation barriers, including those for CO insertion, by approximately 2 kcal/mol relative to **Ni‐2**, consistent with the higher catalytic activity observed experimentally for the phenoxy‐substituted system. Finally, the ΔΔ*G* ^‡^ along the two pathways remains small, yet it aligns with the lower CO incorporation observed for **Ni‐7** (Table S4, entries 24–29).

The phosphine‐phenolate systems, **Ni‐8** and **Ni‐9**, undergo direct CO insertion via **TS‐1‐Ins‐CO‐T** (13.8 and 14.2 kcal/mol, respectively, Figure S162), as the high energy barriers for *cis/trans* isomerization preclude alternative routes (cf. Supporting Information for more details). For **Ni‐9,** the relatively lower energy barrier for ethylene coordination (**TS‐2‐Coor‐E‐C** at 13.4 kcal/mol) along the alternating pathway, combined with a more accessible isomerization along the non‐alternating pathway (**TS‐Isom‐1** at 18.4 kcal/mol), results in higher productivity than **Ni‐8** during the co‐polymerization. Consistent with the imidate series, the energy difference between the rate‐limiting states of the two pathways aligns well with the lower CO incorporation observed for **Ni‐9** (Table S4, entries 30–39).

### Deactivation Pathway

**FIGURE 6:**
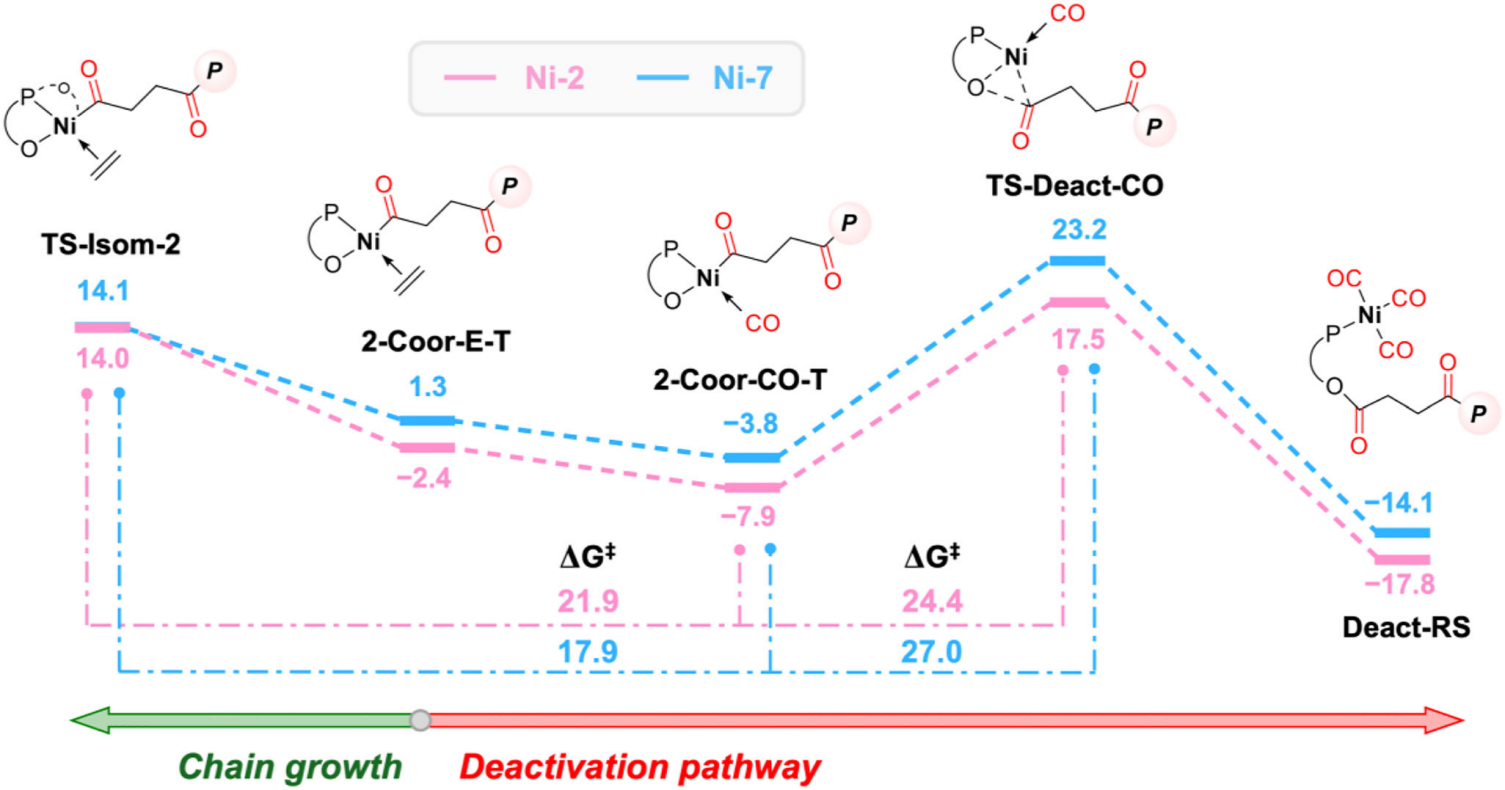
Comparison of the free energies (Δ*G*_tol_ in kcal/mol) of the key steps for deactivation via reductive elimination (red arrow) and chain growth (green arrow) from **2‐Coor‐E‐T** for **Ni‐2** (pink) and **Ni‐7** (blue). The zero‐energy reference is again chelated **1‐cycle5‐T** intermediates.

To rationalize the experimentally observed superior CO tolerance of **Ni‐7**, we investigated reductive elimination from the nickel‐acyl intermediates, a reaction that results in irreversible catalyst deactivation [19], for both phosphine‐imidate Ni(II) systems (Figure 6). Starting from the **2‐Coor‐E‐T** intermediate, ethylene displacement by CO at the nickel center is energetically favorable for both catalysts (5.5 kcal/mol for **Ni‐2** and 5.1 kcal/mol for **Ni‐7**), leading to the formation of **2‐Coor‐CO‐T** at −7.9 and −3.8 kcal/mol for **Ni‐2** and **Ni‐7**, respectively. Subsequent reductive elimination proceeds through the transition state **TS‐Deact‐CO** at 17.5 and 23.2 kcal/mol, with **Ni‐7** exhibiting a higher penalty (by 5.7 kcal/mol) due to steric repulsion between the acyl chain and the bulkier phenoxy substituents. The resulting **Deact‐RS** products, featuring a Ni(0) center stabilized by coordination with the phosphorus atom and three CO molecules, represent a thermodynamically stable species.

The mechanistic origin of the different CO tolerance is revealed by comparing the energy barriers for chain growth versus deactivation. For **Ni‐2**, the transition state **TS‐Isom‐2** along the chain growth pathway lies only 3.5 kcal/mol below **TS‐Deact‐CO** along the deactivation route. Moreover, the stability of **Ni‐2** intermediates, such as **2‐Coor‐E‐T** (at −2.4 kcal/mol) and **2‐Coor‐CO‐T** (at −7.9 kcal/mol), may thermodynamically trap the catalyst in resting states susceptible to reductive elimination. In contrast, **Ni‐7** exhibits a 9.1 kcal/mol kinetic barrier difference between chain growth (**TS‐Isom‐2** at 14.1 kcal/mol) and undesired termination (**TS‐Deact‐CO** at 23.2 kcal/mol) that effectively suppresses deactivation even under high CO concentrations.

## 3 Conclusion

The introduction of *P*‐bound 2,6‐diphenoxyphenyl moieties results in superior CO tolerance, productivity and stability compared to the state‐of‐the‐art benchmark as demonstrated for both phosphine‐imidate and phosphine‐phenolate Ni(II) catalysts. This addresses current key limitations of non‐alternating ethylene‐CO co‐polymerizations: (1) The large operating window of CO feed concentrations much facilitates experimental catalyst development and practical implementation, as these are easier to control and maintain than extremely low feeds (< 0.5 mol%) with conventional polymerization setups. (2) The catalysts’ capability to operate efficiently in the growth of ketone‐containing chains facilitates access to existing keto‐polyethylenes for studies of their materials properties, and more importantly provides materials with much higher keto‐contents than accessible to date, opening the window to novel materials’ compositions. Theoretical studies reveal that the catalysts’ unique reactivity profile originates from Ni···O interactions, which reduces the barriers of the desirable non‐alternating pathway. At the same time the bulky 2,6‐diphenoxyphenyl substituents raise the barrier for undesirable excessive CO insertion barriers, as well as for CO‐induced catalyst deactivation via reductive elimination. The concept uncovered provides perspectives for advancing catalytic access to in‐chain functionalized polyethylenes and much needed environmentally benign plastics.

## Author Contributions

**Yajun Zhao**: conceptualization, investigation, writing – original draft, methodology, formal analysis, writing – review and editing, data curation, funding acquisition, validation, and visualization. **Michele Tomasini**: writing – review and editing, investigation, writing – original draft, methodology, formal analysis, visualization, data curation, and validation. **Inigo Göttker‐schnetmann**: writing – review and editing, formal analysis, software, resources, validation, and visualization. **Laura Falivene**: software, formal analysis, writing – review and editing, validation, and resources. **Lucia Caporaso**: supervision, conceptualization, funding acquisition, writing – original draft, writing – review and editing, project administration, methodology, and validation. **Stefan Mecking**: conceptualization, writing – original draft, funding acquisition, project administration, supervision, writing – review and editing, methodology, and visualization.

## Conflicts of Interest

The authors declare no conflicts of interest.

## Supporting information




**Supporting File 1**: anie73353‐sup‐0001‐SuppMat.docx.

**Supporting File 2**: anie73353‐sup‐0002‐cif.zip

## Data Availability

The data that supports the findings of this study are available in the supplementary material of this article.
