# Supplementary material for: Attractive Ni…O Interactions Enable Non‐Alternating Ethylene‐Carbon Monoxide Copolymerization
Source: Angew Chem Int Ed Engl. 2026 Jun 30;65(36):e6761777. doi: 10.1002/anie.6761777 (PMC13528558; doi:10.1002/anie.6761777)
Supplement: Supplementary file 1 [file ANIE-65-e6761777-s002.docx]

Supporting Information

***Attractive Ni···O Interactions Enable Nonalternating Ethylene-Carbon Monoxide Copolymerization***

**Contents**

[**1. Supplementary Methods**	3](#_Toc231203373)

[**1.1 General methods and materials**	3](#_Toc231203374)

[**1.2 Characterization techniques**	3](#_Toc231203375)

[**1.3 Procedure for copolymerization of ethylene with CO**	5](#_Toc231203376)

[**2. Details of Synthetic Procedure**	7](#_Toc231203377)

[**2.1 Synthesis of phosphine amides**	7](#_Toc231203378)

[**2.2 Synthesis of phosphine phenols**	16](#_Toc231203379)

[**2.3 Synthesis of nickel complexes**	19](#_Toc231203380)

[**2.4 Crystallographic details**	30](#_Toc231203381)

[**3. Additional Polymerization Data and Polymer Characterization**	36](#_Toc231203382)

[**3.1 NMR analysis**	37](#_Toc231203383)

[**3.2 DSC traces**	54](#_Toc231203384)

[**3.3 SEC traces**	71](#_Toc231203385)

[**3.4 FT-IR spectra**	88](#_Toc231203386)

[**3.5 WAXS data**	89](#_Toc231203387)

[**4. Mass Flow Profiles of Ethylene/CO Polymerizations**	90](#_Toc231203388)

[**5. Density Functional Theory (DFT)**	107](#_Toc231203389)

[**5.1 Computational details**	107](#_Toc231203390)

[**5.2 Reaction pathways**	108](#_Toc231203391)

[**5.3 Steric and NCI/AIM analysis**	110](#_Toc231203392)

[**5.4 Additional comments**	113](#_Toc231203393)

[**5.5 Cartesian coordinates**	114](#_Toc231203394)

[**5.5.1 Geometries of Ni-2**	114](#_Toc231203395)

[**5.5.2 Geometries of Ni-7**	144](#_Toc231203396)

[**5.5.3 Geometries of Ni-9**	185](#_Toc231203397)

[**6. Supplementary References**	226](#_Toc231203398)

# **1. Supplementary Methods**

All manipulations of air and moisture sensitive materials were carried out under inert gas atmosphere using standard glovebox and *Schlenk* techniques.

## **1.1 General methods and materials**

Solvents were dried and degassed using standard laboratory techniques. THF was distilled from sodium benzophenone ketyl, benzene from sodium, dichloromethane was distilled from CaH_2_, and pentane and toluene were dried and degassed by passing through columns equipped with alumina and *BASF R3-11* catalyst. Ethylene (grade 3.5) and carbon monoxide (grade 4.7) was supplied by *Air Liquide* and used as received. The nickel precursor [(tmeda)NiMe_2_] was prepared according to a reported procedure. ^1^ All other commercially available reagents and starting materials were supplied by *Sigma Aldrich*, *Acros*, *TCI*, or *ABCR*. CDCl_3_ was supplied by *Sigma Aldrich*, all other deuterated solvents by *Eurisotop*.

## **1.2 Characterization techniques**

Nuclear magnetic resonance (NMR) spectra were recorded on a *Bruker Avance III HD 400* (^1^H: 400 MHz, ^13^C: 101 MHz, ^31^P: 162 MHz, ^19^F: 376 MHz). ^1^H chemical shifts were referenced to the solvent’s residual proton signals (CDCl_3_: 7.26 ppm, C_2_D_2_Cl_4_: 6.0 ppm, C_6_D_6_: 7.16 ppm). ^13^C chemical shifts were referenced to the carbon signal of the deuterated solvent (CDCl_3_: 77.16 ppm, C_2_D_2_Cl_4_: 74.00 ppm, C_6_D_6_: 128.06 ppm). ^19^F chemical shifts were referenced to external BF_3_·OEt_2_. NMR spectra of keto-polyethylene were acquired in 1,1,2,2-tetrachloroethane-*d_2_* (C_2_D_2_Cl_4_, TCE-*d*_2_). Calculation of the CO incorporation ratio and the selectivity are according to the reported literature. ^2, 3, 4, 5^

Differential scanning calorimetry (DSC) measurements were carried out on a *Netzsch DSC 204 F1* instrument (software: *Netzsch Proteus Thermal Analysis*, version 6.1.0) with heating and cooling rates of 10 K∙min^-1^ under a nitrogen atmosphere. Crystallinities were determined by assuming a melt enthalpy of 293 J∙g^-1^ for 100% crystalline polyethylene.

Size exclusion chromatography (SEC) was performed on a *PolymerChar GPC-IR* instrument equipped with PSS Polefin Linear XL columns (3 × 30 cm, additional guard column), an infrared detector (IR5 MCT or IR4 dual wavelength for methylene and carbonyl bands (1715 cm^-1^)) and a four-capillary viscometer at 160 °C in 1,2-dichlorobenzene at 1.0 mL∙min^-1^ flow rate. Sample molecular weights were analyzed via universal calibration employing narrow polyethylene standards. The raw data was evaluated with *PSS WinGPC UniChrom* software.

FT-IR spectra of polymers were acquired on *a Perkin Elmer Spectrum 100* instrument.

Wide angle X-ray scattering (WAXS) diffractograms were recorded on a *D8 Discover* instrument (Bruker) with a *Vantec* (Bruker) detector.

X-ray diffraction analysis was performed at 100 K on a *STOE IPDS-2T* diffractometer, equipped with a graphite-monochromated radiation source (λ = 0.71073 Å) and an image plate detection system. Crystals were placed on a fine glass fiber with grease or oil. The selection, integration and averaging procedure of the measured reflex intensities, the determination of the unit cell dimensions and a least-squares fit of the 2Θ values as well as data reduction, LP-correction and space group determination were performed using the *X-Area software* package delivered with the diffractometer. ^6^ Using *Olex2*, ^7^ the structure was solved with the ShelXT ^8^ structure solution program using intrinsic phasing and refined with the *Olex2* ^9^ refine refinement package using Gauss-Newton minimization. Graphical representations were created by the *ORTEP-3 V2.02.* for Windows XP software package. ^10^

Topographical maps of the calculated key intermediates and transition states, as well as corresponding percent buried volume data (%V_Bur_) were generated by *Cavallo's SambVca 2.1* (Salerno molecular buried volume calculation) program. ^11^ For all complexes, xz-plane and the z-axis were defined following the methods for the Unsymmetric complex in *SambVca 2.1-Library*. ^12, 13, 14^

Calculation of the CO incorporation ratio and the selectivity are according to the reported literature. ^2, 3, 4, 5^

Typical ^1^H NMR spectrum of keto-polyethylene for calculation clarification.

The overall keto content was determined as the ratio of keto groups to the combined ethylene repeat units and keto groups.

$$\text{n}_{\text{CO}}=\frac{\text{I}_{a}+\text{I}_{b}+\text{I}_{c+c'+c''}+\text{I}_{APK}}{4}$$

$$\text{n}_{\text{CH}}=\frac{\text{I}_{f}+\text{I}_{e}}{2}$$

$$\text{n}_{\text{CH2}}=\frac{\text{I}_{\text{-CH2-}}\text{+ I}_{h}+\text{I}_{a}+\text{I}_{b}+\text{I}_{c+c'+c''}+\text{I}_{APK}}{2}$$

$$\text{n}_{\text{CH3}}=\frac{\text{I}_{\text{k+d}}}{3}$$

$$\text{CO}_{\text{i.r.}}\text{[mol\%]}=\frac{\text{n}_{\text{CO}}}{\text{n}_{\text{CO}}+\text{(}\frac{\text{n}_{\text{CH}}+\text{n}_{\text{CH2}}+\text{n}_{\text{CH3}}}{2}\text{)}}$$

The portion of isolated (I), non-alternating (NA) and alternating (A) keto groups, respectively, was determined as the ratio of the integrals of the respective proton signal and the sum of integrals of all protons adjacent to ketone groups.

$$\text{I [\%]}=\frac{\text{I}_{a}}{\text{I}_{a}+\text{I}_{b}+\text{I}_{c+c'+c''}+\text{I}_{APK}}$$

$$\text{NA [\%]}=\frac{\text{I}_{b}+\text{I}_{c+c'+c''}}{\text{I}_{a}+\text{I}_{b}+\text{I}_{c+c'+c''}+\text{I}_{APK}}$$

$$\text{A [\%]}=\frac{\text{I}_{APK}}{\text{I}_{a}+\text{I}_{b}+\text{I}_{c+c'+c''}+\text{I}_{APK}}$$

## **1.3 Procedure for copolymerization of ethylene with CO**

Polymerizations were conducted in a *Büchi* miniclave reactor (Figure S1) with a 300 mL vessel, equipped with a heating and cooling jacket connected to a thermostat, a magnetically coupled mechanical pitched-blade stirrer, a nitrogen/vacuum supply and a thermocouple couple dipping into the reaction mixture. Gas flows of ethylene and CO were individually monitored and supplied via *Bronkhorst* mass flow controllers. The gas supply and temperature were controlled via *LabVision Software* provided by *Hitec Zang*. ^15^

**Figure S1.** Flow diagram of the setup for the copolymerization of ethylene with CO. ^15^

Prior to all polymerization experiments, the reactor was evacuated and heated for 3 h (thermostat temperature: 100 °C). The reactor was flushed with nitrogen, evacuated three times, and filled with 100 mL of toluene via cannula transfer. 5 μmol of catalyst precursor was dissolved in 2 mL of toluene, and the solution was transferred into the reactor via syringe. Immediately after addition, the stirring rate was increased to 1000 rpm and the reactor was pressurized with the desired mixture of ethylene and CO to the designated pressure. During the pressurization procedure, the temperature was adjusted to the desired reaction temperature. A constant pressure was maintained over the entire polymerization experiment by feeding both monomers automatically (to replenish consumed gaseous monomer). After the desired reaction time, the gas flow was stopped, and the reactor carefully vented and cooled. The reactor content was poured into 500 mL of methanol and stirred for 30 minutes. The precipitated polymer was filtrated, washed with methanol and dried in a vacuum oven (60 °C, 30 mbar) overnight.

.

# **2. Details of Synthetic Procedure**

## **2.1 Synthesis of phosphine amides**

Compounds **L-1** to **L-7** were obtained as follows. At room temperature *n*‐BuLi (2.5 M in hexane, 1.1 mmol, 1.1 equiv.) was added dropwise to a solution of amide 1 mmol, 1.0 equiv.) in THF (20 mL) at -40 ºC. The reaction mixture was stirred for 30 min to give a colorless solution. A slurry of disubstituted phosphine chloride (Ar^1^Ar^2^PCl, 1.05 mmol, 1.05 eq) in THF was slowly added, and the solution was allowed to warm to ambient temperature and stirred for 2 h. All volatiles were removed, and the product was extracted with DCM. The solution was filtered through a pad of Celite, and the volatiles were removed under vacuum until a precipitate formed. The solution was triturated with DCM/pentane. After the filtration and dryness, a white or light-yellow solid was isolated.

**L-1** was obtained in 46% yield, 202 mg.

^1^H NMR (400 MHz, CDCl_3_-*d*_1_) δ 8.28-8.22 (m, 2H, 3- and 5-H), 8.07-8.00 (m, 1H, 1-H), 7.52-7.40 (m, 10H, 10- to 14-H), 6.5 (br, 1H, 8-H).

^31^P NMR (162 MHz, CDCl_3_-*d*_1_) δ 28.76.

^19^F NMR (376 MHz, CDCl_3_-*d*_1_) δ -62.87.

**L-2** was obtained in 64% yield, 359 mg.

^1^H NMR (400 MHz, Benzene-*d*_6_) δ 9.45 (br, 1H, 8-H), 8.23 (s, 2H, 3- and 5-H), 7.68 (s, 1H, 1-H), 7.03-6.96 (m, 2H, 12-H), 6.22 (dd, *J* = 8.3, 2.6 Hz, 1H), 3.33 (s, 12H, 15-H).

^31^P NMR (162 MHz, Benzene-*d*_6_) δ -1.29.

^19^F NMR (376 MHz, Benzene-*d*_6_) δ -62.71.

**L-3** was obtained in 40% yield, 250 mg.

^1^H NMR (400 MHz, Benzene-*d*_6_) δ 9.44 (br, 1H, 8-H), 8.31 (s, 2H, 3- and 5-H), 7.67 (s, 1H, 1-H), 6.97 (t, *J* = 8.3 Hz, 1H, 12-H), 6.90 (t, *J* = 7.7 Hz, 2H, 16- and 20-H), 6.83 (t, *J* = 8.2 Hz, 1H, 12’-H), 6.75-6.66 (m, 3H, 17-, 18- and 19-H), 3.40 (s, 3H, 21-H), 3.30 (s, 6H, 21’-H).

^31^P NMR (162 MHz, Benzene-*d*_6_) δ -0.06.

^19^F NMR (376 MHz, Benzene-*d*_6_) δ -62.87.

**L-4** was obtained in 76% yield, 520 mg.

^1^H NMR (400 MHz, Benzene-*d*_6_) δ 9.39 (br, 1H, 8-H), 8.08 (s, 2H, 3- and 5-H), 7.64 (s, 1H, 1-H), 6.98-6.89 (m, 4H, 16- and 20-H), 6.82 (t, *J* = 8.2 Hz, 2H, 12-H), 6.79-6.70 (m, 6H, 17-, 18- and 19-H), 6.32 (dd, *J* = 8.5, 1.9 Hz, 2H, 11- and 13-H), 6.15 (dd, *J* = 8.5, 3.2 Hz, 2H, 11’- and 13’-H), 3.28 (s, 6H, 21-H).

^31^P NMR (162 MHz, Benzene-*d*_6_) δ -0.36.

^19^F NMR (376 MHz, Benzene-*d*_6_) δ -62.66.

**L-5** was obtained in 86% yield, 590 mg.

^1^H NMR (400 MHz, Benzene-*d*_6_) δ 9.43 (br, 1H, 8-H), 8.03 (s, 2H, 3- and 5-H), 7.65 (s, 1H, 1-H), 7.00-6.94 (m, 5H,12-, 16- and 20-H), 6.90-6.83 (m, 4H, 17- and 19-H), 6.78 (t, *J* = 7.4 Hz, 2H, 18-H), 6.68 (t, *J* = 8.2 Hz, 1H, 12’-H), 6.40 (dd, *J* = 8.2, 2.3 Hz, 2H, 11- and 13-H), 6.15 (dd, *J* = 8.6, 2.8 Hz, 2H, 11’- and 13’-H), 3.29 (s, 6H, 21-H).

^31^P NMR (162 MHz, Benzene-*d*_6_) δ -0.32.

^19^F NMR (376 MHz, Benzene-*d*_6_) δ -62.69.

**L-6** was obtained in 88% yield, 660 mg.

^1^H NMR (400 MHz, Benzene-*d*_6_) δ 9.34 (br, 1H, 8-H), 8.02 (s, 2H, 3- and 5-H), 7,61 (s, 1H, 1-H), 7.00-6.92 (m, 6H, 16- and 20-H), 6.87-6.73 (m, 10H, 17-, 18-, 19 and 12-H), 6.71-6.63 (m, 1H, 12’-H), 6.35 (d, *J* = 8.2 Hz, 2H, 11- and 13-H), 6.31 (dd, *J* = 8.2, 1.8 Hz, 1H, 11’- and 13’-H), 6.13 (dd, *J* = 8.2, 3.5 Hz, 1H, 11’- and 13’-H), 3.26 (s, 3H, 21-H).

^31^P NMR (162 MHz, Benzene-*d*_6_) δ -0.00.

^19^F NMR (376 MHz, Benzene-*d*_6_) δ -62.65.

**L-7** was obtained in 80% yield, 647 mg.

^1^H NMR (400 MHz, Benzene-*d*_6_) δ 9.30 (s, 1H, 8-H), 8,01 (br, 2H, 3- and 5-H), 7,58 (br, 1H, 1-H), 6.99-6,95 (m, 8H, 16- and 20-H), 6.84-6,79 (m, 12H, 17-, 18- and 19-H), 6,70-6,66 (m, 2H, 12-H), 6.33 (dd, *J* = 8.2, 2.5 Hz, 4H, 11- and 13-H).

^13^C NMR (101 MHz, Benzene-*d*_6_) δ 160.90 (d, J = 10.7 Hz, C7), 156.55 (C10, C14, C15), 137.25 (C4), 131.41 (C2), 131.14 (C6), 130.85 (C12), 129.68 (C17, C19), 124.22 (CF_3_), 123.88 (C16, C20), 122.05 (C1), 120.57 (C3), 120.30 (C5), 119.89 (C11, C13), 112.44 (C9). The signals for C18 are overlapped by C_6_D_6_.

^31^P NMR (162 MHz, Benzene-*d*_6_) δ -0.06.

^19^F NMR (376 MHz, Benzene-*d*_6_) δ -62.87.

**Figure S2.** ^1^H NMR spectrum (CDCl_3_ , 300 K) of **L-1**.

**Figure S3.** ^1^H NMR spectrum of **L-2** in C_6_D_6_ at 300 K.

**Figure S4.** ^1^H NMR spectrum of **L-3** in C_6_D_6_ at 300 K.

**Figure S5.** ^1^H NMR spectrum of **L-4** in C_6_D_6_ at 300 K.

**Figure S6.** ^1^H NMR spectrum of **L-5** in C_6_D_6_ at 300 K.

**Figure S7.** ^1^H NMR spectrum of **L-6** in C_6_D_6_ at 300 K.

**Figure S8.** ^1^H NMR spectrum of **L-7** in C_6_D_6_ at 300 K.

**Figure S9.** ^13^C NMR spectrum of **L-7** in C_6_D_6_ at 300 K.

## **2.2 Synthesis of phosphine phenols**

Compound **L-8** was prepared according to a reported procedure. ^16^

To obtain **L-9**, at room temperature *n*-BuLi (1.2 mL, 2.5 M in hexane, 3.0 mmol, 1.1 equiv.) was added dropwise to a solution of 2-phenoxytetrahydro-2H-pyran (0.5 g, 2.8 mmol, 1.0 equiv.) in diethyl ether (30 mL). The reaction mixture was stirred for 2 hours to yield a white suspension. The obtained suspension was added dropwise to a solution of bis(diphenoxyphenyl) phosphine chloride (PAr_2_Cl, 1.65 g, 2.8 mmol, 1.0 equiv.) in THF (10 mL) at -78 °C. The mixture was warmed to room temperature slowly over 8 hours to afford a clear solution. Volatiles were removed under vacuum, and the residue was dissolved in 10 mL of degassed ethyl acetate, and washed with water (2 × 20 mL). The aqueous phase was extracted with ethyl acetate (10 mL). The combined organic phases were concentrated, and the obtained crude product **9a** was employed for the next step without further purification.

In a 50 mL Schlenk flask, *n*-BuLi (0.92 mL, 2.5 M in hexane, 2.3 mmol, 1.1 equiv.) was added dropwise to a solution of **9a** (1.52 g, 2.1 mmol) in 15 mL of THF at -78 °C. The mixture was stirred for 1 hour at this temperature. Hexafluorobenzene (1.93 g, 10.4 mmol, 5 equiv.) was added in 5 mL of THF. The reaction mixture was warmed to room temperature slowly over 8 hours, concentrated, and dissolved in 10 mL of degassed ethyl acetate and washed with water (2 × 20 mL). The organic phases were concentrated, and the crude product **9b** was employed for the next step without further purification.

The obtained **9b** (1 g, 1.12 mmol) was dissolved in 20 mL of degassed MeOH/DCM (8:2, v/v), and *p*-toluenesulfonic acid monohydrate (*p*-TsOH·H_2_O, 1.06g, 5.60 mmol, 5 equiv.) was added. After stirring at room temperature overnight, the mixture was added slowly into a solution of 5 g NaHCO_3_ in 60 mL of water and stirred for 30 minutes. The organic phase was separated, and the aqueous phase was extracted with ethyl acetate (2 × 20 mL). The combined organic phases were concentrated, and the residue was purified by column chromatography on silica using petrol ether/ethyl acetate = 10/1 as eluent to afford pure **L-9** (0.45 g, 0.72 mmol, 63%).

^1^H NMR (400 MHz, Benzene-*d*_6_) δ 7.98 (ddd, *J* = 9.3, 7.7, 1.7 Hz, 1H, 8-H), 7.59 (br, 1H, OH), 7.01-6.96 (m, 8H, 20-, and 24-H), 6.90-6.79 (m, 12H, 21-, 22- and 23-H), 6.74-6.65 (m, 2H, 16-H), 6.60-6.54 (m, 1H, 10-H), 6.43 (dd, *J* = 8.2, 2.9 Hz, 4H, 15- and 17-H).

^13^C NMR (101 MHz, Benzene-*d*_6_) δ 159.86 (d, *J* = 9.0 Hz, C14, C18), 156.99 (d, *J* = 15.0 Hz, C12), 156.56 (C19), 138.07 (d, *J* = 22.2 Hz, C8), 132.80 (C13), 130.20 (C16), 129.51 (C21, C23), 129.14 (C11), 128.21 (C7), 123.23 (C22), 122.32 (d, *J* = 8.4 Hz, C9), 118.68 (C20, C24), 117.91 (d, *J* = 18.3 Hz, C10), 113.20 (C15, C17).

^31^P NMR (162 MHz, Benzene-*d*_6_) δ -60.70.

^19^F NMR (376 MHz, Benzene-*d*_6_) δ -139.81 (dd, J = 23.8, 7.8 Hz), -157.16 (t, J = 21.5 Hz), -163.98 (td, J = 23.5, 7.9 Hz).

**Figure S10.** ^1^H NMR spectrum (C_6_D_6_, 300 K ) of **L-9**.

**Figure S11.** ^13^C NMR spectrum of **L-9** in C_6_D_6_ at 300 K.

## **2.3 Synthesis of nickel complexes**

General procedure for the synthesis of nickel complexes: To [(tmeda)NiMe_2_] (1.05 equiv.) and the corresponding phosphine amides or phosphine phenols, respectively (100 μmol, 1 equiv.), a solution of pyridine (25 equiv.) in 5 mL of benzene was added. Gas evolution (methane) was observed. After 8 hours at room temperature, volatiles were removed in vacuo. The residue was dispersed with benzene again, and the formed nickel black formed during the reaction was removed via centrifugation. The yellow solution was frozen in liquid nitrogen und the solvent removed by freezing drying to give the desired product as a yellow powder.

**Ni-1** was obtained in 41% yield, 24 mg.

^1^H NMR (400 MHz, Benzene-*d*_6_) δ 8.90 (s, 2H, 3- and 5-H), 8.48 (br, 2H, 14- and 18-H), 8.12-8.02 (m, 4H, 9- and 13-H), 7.77 (s, 1H, 1-H), 7.14-7.07 (m, 6H, 10-, 11- and 12-H, overlapping with solvent), 6.79 (br, 1H, 16-H), 6.47 (br, 2H, 15- and 17-H), -0.23 (d, *J* = 4.7 Hz, 3H, 19-H).

^31^P NMR (162 MHz, Benzene-*d*_6_) δ 78.19.

^19^F NMR (376 MHz, Benzene-*d*_6_) δ -62.53.

**Ni-2** was obtained in 47% yield, 33 mg.

^1^H NMR (400 MHz, Benzene-*d*_6_) δ 8.97 (s, 2H, 3- and 5-H), 8.80 (br, 2H, 14- and 18-H), 7.73 (s, 1H, 1-H), 7.14-7.10 (m, 2H, 11-H, overlapping with solvent), 6.80 (br, 1H, 16-H), 6.48 (br, 2H, 15- and 17-H), 6.34 (dd, J = 8.3, 3.7 Hz, 4H, 10- and 12-H), 3.35 (s, 12H, 20-H), -0.30 (d, *J* = 5.6 Hz, 33H, 19-H).

^31^P NMR (162 MHz, Benzene-*d*_6_) δ 57.27.

^19^F NMR (376 MHz, Benzene-*d*_6_) δ -62.33.

**Ni-3** was obtained in 75% yield, 58 mg.

^1^H NMR (400 MHz, Benzene-*d*_6_) δ 8.68 (s, 2H, 3- and 5-H), 8.63 (br, 2H, 14- and 18-H), 7.71 (s, 1H, 1-H), 7.14-7.06 (m, 3H, 22-, 23- and 24-H, overlapping with solvent), 7.04-6.95 (m, 3H, 11-, 21- and 25-H), 6.82-6.74 (m, 2H, 15- and 17-H), 6.63 (dd, *J* = 7.7, 4.2 Hz, 1H, 11’-H), 6.49-6.41 (m, 2H, 10- and 12-H), 6.39-6.34 (m, 1H, 16-H), 6.33-6.28 (m, 2H, 10’- and 12’-H), 3.44 (s, 3H, 26-H), 3.34 (s, 3H, 26-H), 3.23 (s, 3H, 26-H), -0.24 (d, *J* = 5.7 Hz, 3H, 19-H).

^31^P NMR (162 MHz, Benzene-*d*_6_) δ 56.59.

^19^F NMR (376 MHz, Benzene-*d*_6_) δ -62.37.

**Ni-4** was obtained in 90% yield, 75 mg.

^1^H NMR (400 MHz, Benzene-*d*_6_) δ 8.55 (s, 2H, 14- and 18-H), 8.36 (br, 2H, 3- and 5-H), 7.68 (s, 1H, 1-H), 7.10-7.05 (m, 2H, 21- and 25-H), 7.02-6.98 (m, 3H, 22-, 23- and 24-H), 6.93-6.89 (m, 3H, 22’-, 23’- and 24’-H), 6.85-6.81 (m, 2H, 21’- and 25’-H), 6.77-6.69 (m, 3H, 11- and 16-H), 6.61-6.56 (m, 2H, 15- and 17-H), 6.43-6.31 (m, 4H, 10- and 12-H), 3.42 (s,6H, 26-H), 3.27 (s,6H, 26’-H), -0.16 (d, *J* = 5.7 Hz, 1H, 19-H).

^31^P NMR (162 MHz, Benzene-*d*_6_) δ 56.58.

^19^F NMR (376 MHz, Benzene-*d*_6_) δ -62.23.

**Ni-5** was obtained in 86% yield, 71 mg.

^1^H NMR (400 MHz, Benzene-*d*_6_) δ 8.61 (s, 2H, 3- and 5-H), 8.50 (br, 2H, 14- and 18-H), 7.74 (s, 1H, 1-H), 7.08-7.00 (m, 9H, 21-, 22-, 24-, 25- and 11-H), 6.87 -6.81 (m, 3H, 11’-, 23-H), 6.74 (br, 1H, 16-H), 6.61 (dd, *J* = 8.1, 3.3 Hz, 2H, 10- and 12-H), 6.31 (br, 2H, 15- and 17-H), 6.29 (dd, *J* = 8.3, 3.8 Hz, 1H, 10’- and 12’-H), 3.37 (s,6H, 26-H), -0.18 (d, *J* = 5.8 Hz, 1H, 19-H).

^31^P NMR (162 MHz, Benzene-*d*_6_) δ 56.85.

^19^F NMR (376 MHz, Benzene-*d*_6_) δ -62.32.

**Ni-6** was obtained in 95% yield, 85 mg.

^1^H NMR (400 MHz, Benzene-*d*_6_) δ 8.40 (br, 2H, 14- and 18-H), 8.26 (s, 2H, 3- and 5-H), 7.70 (s, 1H, 1-H), [7.14-7.08 (m, 2H), 7.06-7.00 (m, 4H), 6.98-6.91 (m, 4H) all belong to 21- to 25-H], 6.89-6.83 (m, 5H, 21’- to 25’-H), 6.81-6.77 (m, 1H, 16-H), 6.75-6.69 (m, 2H, 11-H), [6.65-6.611 (m, 1H), 6.59-6.55 (m, 1H), 6.54-6.50 (m, 1H,) all belong to 10- and 12-H], 6.40-6.35 (m, 2H, 15- and 17-H), 6.34-6.30 (m, 1H, 10’- and 12’-H),3.32 (s, 3H, 26-H), -0.10 (d, *J* = 5.8 Hz, 1H, 19-H).

^31^P NMR (162 MHz, Benzene-*d*_6_) δ 57.25.

^19^F NMR (376 MHz, Benzene-*d*_6_) δ -62.15.

**Ni-7** was obtained in 95% yield, 92 mg.

^1^H NMR (400 MHz, Benzene-*d*_6_) δ 8.35 (br, 2H, 14- and 18-H), 8.19 (s, 2H, 3- and 5-H), 7.70 (s, 1H, 1-H), 7.02-6.94 (m, 16H, 21-, 22-, 24- and 25-H), 6.83-6.75 (m, 6H, 11- and 23- H), 6.69-6.66 (m, 1H, 16-H), 6.54 (dd, *J* = 8.2, 3.4 Hz, 4H, 10- and 12-H), 6.34 (br, 2H, 15- and 17-H), -0.06 (d, *J* = 5.9 Hz, 1H, 19-H).

^13^C NMR (101 MHz, Benzene-*d*_6_) δ 175.33 (C7), 158.83 (C9, C13), 157.92 (C20), 150.08 (C14, C18), 138.59 (d, *J* = 18.3 Hz, C8, 130.84 (C2, C6), 129.61 (C3, C5), 129.30 (C22, C24), 124.95 (C16), 122.75 (C15, C17), 120.89 (C1), 120.45 (C4), 118.88 (21, C25), 114.61 (C10), C114.58 (C12), -15.01 (d, *J* = 34.8 Hz, C19).

^31^P NMR (162 MHz, Benzene-*d*_6_) δ 57.46.

^19^F NMR (376 MHz, Benzene-*d*_6_) δ -62.74.

**Ni-9** was obtained in 92% yield, 87 mg.

^1^H NMR (400 MHz, Benzene-*d*_6_) δ 8.29 (br, 2H, 19- and 23-H), 8.08 (ddd, *J* = 11.0, 7.7, 1.7 Hz, 1H, 8-H), 7.09-7.08 (m, 1H, 9-H), 7.04-7.00 (m, 16H, 26-, 27-, 29- and 30-H), 6.85-6.82 (m, 4H, 28-H), 6.77-6.74 (m, 3H, 10-, and 16-H), 6.49 (dd, *J* = 8.2, 3.4 Hz, 4H, 15- and 17-H), 6.45-6.42 (m, 2H, 20- and 22-H), 6.38 (td, *J* = 7.6, 1.8 Hz, 1H, 21-H), , -0.45 (d, *J* = 6.5 Hz, 3H, 24-H).

^13^C NMR (101 MHz, Benzene-*d*_6_) δ 172.28 (d, *J* = 25.2 Hz, C12), 159.69 (d, *J* = 1.5 Hz, C14, C18), 157.43 (C19, C23), 156.39 (C25), 138.32 (C21), 133.87 (d, *J* = 52.4 Hz, C13), 130.57 (C16), 129.59 (C27, C29), 124.31 (d, *J* = 54.4 Hz, C11), 123.47 (C16, C28), 122.78 (d, *J* = 32.6 Hz, C20, C22), 119.71 (C26, C30), 119.33 (d, *J* = 21.1 Hz, C7), 115.01 (d, *J* = 45.6 Hz, C10), 112.96 (d, *J* = 45.1 Hz, C8),112.0 (C15, C17), -15.48 (d, *J* = 34.5 Hz, C24).

^31^P NMR (162 MHz, Benzene-*d*_6_) δ -1.10.

^19^F NMR (376 MHz, Benzene-*d*_6_) -138.36 (dd, *J* = 24.1, 7.0 Hz), -161.00 (t, *J* = 21.4 Hz), -166.13 (td, *J* = 23.7, 6.8 Hz).

**Figure S12.** ^1^H NMR spectrum of **Ni-1** in C_6_D_6_ at 300 K.

**Figure S13.** ^1^H NMR spectrum of **Ni-2** in C_6_D_6_ at 300 K.

**Figure S14.** ^1^H NMR spectrum of **Ni-3** in C_6_D_6_ at 300 K.

**Figure S15.** ^1^H NMR spectrum of **Ni-4** in C_6_D_6_ at 300 K.

**Figure S16.** ^1^H NMR spectrum of **Ni-5** in C_6_D_6_ at 300 K.

**Figure S17.** ^1^H NMR spectrum of **Ni-6** in C_6_D_6_ at 300 K.

**Figure S18.** ^1^H NMR spectrum of **Ni-7** in C_6_D_6_ at 300 K.

**Figure S19.** ^13^C NMR spectrum of **Ni-7** in C_6_D_6_ at 300 K.

**Figure S20.** ^1^H NMR spectrum of **Ni-9** in C_6_D_6_ at 300 K.

**Figure S21.** ^13^C NMR spectrum of **Ni-9** in C_6_D_6_ at 300 K.

## **2.4 Crystallographic details**

**Figure S22.** Crystal structure of complex **Ni-2** (50% probability ellipsoids), hydrogen atoms are omitted for clarity.

**Experimental**

Single crystals of C_33.5_H_36_F_6_N_2_NiO_5_P [**Ni-2**, 0.5 pentane] were grown at 298 K from toluene solution after layering with pentane. A suitable crystal was selected and mounted on a glass fiber on a STOE IPDS 2T diffractometer. The crystal was kept at 100 K during data collection. Using Olex2 ^7^, the structure was solved with the SHELXT ^8^ structure solution program using Intrinsic Phasing and refined with the SHELXL ^17^ refinement package using Least Squares minimisation.

**Table S1.** Crystal data and structure refinement of complex **Ni-2**.

| Identification code | **CCDC 2515315** |
| --- | --- |
| Empirical formula | C_33.5_H_36_F_6_N_2_NiO_5_P |
| Formula weight | 750.32 |
| Temperature/K | 100 |
| Crystal system | triclinic |
| Space group | P-1 |
| a/Å | 10.6664(14) |
| b/Å | 13.5675(18) |
| c/Å | 14.3022(19) |
| α/° | 107.694(10) |
| β/° | 103.439(10) |
| γ/° | 108.636(10) |
| Volume/Å^3^ | 1739.5(4) |
| Z | 2 |
| ρ_calc_g/cm^3^ | 1.433 |
| μ/mm^‑1^ | 0.678 |
| F(000) | 776.0 |
| Crystal size/mm^3^ | 0.35 × 0.143 × 0.03 |
| Radiation | Mo Kα (λ = 0.71073) |
| 2Θ range for data collection/° | 3.636 to 53.904 |
| Index ranges | -13 ≤ h ≤ 13, -17 ≤ k ≤ 17, -18 ≤ l ≤ 16 |
| Reflections collected | 18061 |
| Independent reflections | 7373 [R_int_ = 0.1687, R_sigma_ = 0.2693] |
| Data/restraints/parameters | 7373/0/420 |
| Goodness-of-fit on F^2^ | 0.753 |
| Final R indexes [I>=2σ (I)] | R_1_ = 0.0889, wR_2_ = 0.2139 |
| Final R indexes [all data] | R_1_ = 0.2765, wR_2_ = 0.2970 |
| Largest diff. peak/hole / e Å^-3^ | 0.63/-0.33 |
| Special details | 1 disordered molecule of pentane removed from a  solvent accessible void (339.7 Å^3^) at  -0.212, 0.288, 0.5 in the unit cell by  the Olex solvent mask |

**Figure S23.** Crystal structure of complex **Ni-7** (50% probability ellipsoids), hydrogen atoms are excluded for clarity.

**Experimental**

Single crystals of C_51_H_37_F_6_N_2_NiO_5_P **[Ni-7]** were grown at 298 K from a toluene solution after layering with pentane. A suitable crystal was selected and mounted on a glass fiber on a STOE IPDS 2T diffractometer. The crystal was kept at 100 K during data collection. Using Olex2 ^7^, the structure was solved with the SHELXT ^8^ structure solution program using Intrinsic Phasing and refined with the SHELXL ^17^ refinement package using Least Squares minimisation.

**Table S2.** Crystal data and structure refinement of complex **Ni-7**.

| Identification code | **CCDC 2515318** |
| --- | --- |
| Empirical formula | C_51_H_37_F_6_N_2_NiO_5_P |
| Formula weight | 961.50 |
| Temperature/K | 100 |
| Crystal system | monoclinic |
| Space group | P2_1_/c |
| a/Å | 9.8062(3) |
| b/Å | 9.2941(2) |
| c/Å | 47.8878(15) |
| α/° | 90 |
| β/° | 95.287(2) |
| γ/° | 90 |
| Volume/Å^3^ | 4345.9(2) |
| Z | 4 |
| ρ_calc_g/cm^3^ | 1.470 |
| μ/mm^‑1^ | 0.561 |
| F(000) | 1976.0 |
| Crystal size/mm^3^ | 0.45 × 0.25 × 0.05 |
| Radiation | Mo Kα (λ = 0.71073) |
| 2Θ range for data collection/° | 4.172 to 53.604 |
| Index ranges | -12 ≤ h ≤ 12, -11 ≤ k ≤ 11, -60 ≤ l ≤ 60 |
| Reflections collected | 28512 |
| Independent reflections | 9130 [R_int_ = 0.0314, R_sigma_ = 0.0327] |
| Data/restraints/parameters | 9130/0/597 |
| Goodness-of-fit on F^2^ | 1.132 |
| Final R indexes [I>=2σ (I)] | R_1_ = 0.0493, wR_2_ = 0.1295 |
| Final R indexes [all data] | R_1_ = 0.0723, wR_2_ = 0.1443 |
| Largest diff. peak/hole / e Å^-3^ | 0.68/-0.56 |

**Figure S24.** Crystal structure of complex **Ni-8** (30% probability ellipsoids), hydrogen atoms are excluded for clarity.

**Experimental**

Single crystals of C_43_H_41_F_5_NNiO_3_P **[Ni-8, 1 pentane]** were grown from toluene solution after layering with pentane. A suitable crystal was selected and mounted on a glas fiber on a STOE IPDS 2T diffractometer. The crystal was kept at 100 K during data collection. Using Olex2 ^7^, the structure was solved with the SHELXT ^8^ structure solution program using Intrinsic Phasing and refined with the SHELXL ^17^ refinement package using Least Squares minimisation.

**Table S3.** Crystal data and structure refinement of complex **Ni-8**.

| Identification code | **CCDC 2515317** | |
| --- | --- | --- |
| Empirical formula | C_43_H_41_F_5_NNiO_3_P | |
| Formula weight | 804.45 | |
| Temperature/K | 100 | |
| Crystal system | monoclinic | |
| Space group | P2_1_/n | |
| a/Å | 20.8405(6) | |
| b/Å | 14.8480(4) | |
| c/Å | 26.6480(9) | |
| α/° | 90 | |
| β/° | 107.585(2) | |
| γ/° | 90 | |
| Volume/Å^3^ | 7860.6(4) | |
| Z | 8 | |
| ρ_calc_g/cm^3^ | 1.360 | |
| μ/mm^‑1^ | 0.598 | |
| F(000) | 3344.0 | |
| Crystal size/mm^3^ | 0.5 × 0.187 × 0.01 | |
| Radiation | Mo Kα (λ = 0.71073) | |
| 2Θ range for data collection/° | 3.206 to 53.642 | |
| Index ranges | -26 ≤ h ≤ 26, -18 ≤ k ≤ 18, -33 ≤ l ≤ 33 | |
| Reflections collected | 96039 | |
| Independent reflections | 16749 [R_int_ = 0.1644, R_sigma_ = 0.1148] | |
| Data/restraints/parameters | 16749/0/889 | |
| Goodness-of-fit on F^2^ | 1.124 | |
| Final R indexes [I>=2σ (I)] | R_1_ = 0.1222, wR_2_ = 0.3585 | |
| Final R indexes [all data] | R_1_ = 0.1962, wR_2_ = 0.3858 | |
| Largest diff. peak/hole / e Å^-3^ | 0.78/-0.93 | |
| Special details | 8 disordered molecules of pentane removed from 2  solvent accessible voids (918.7 Å^3^ each) at  0 -0.505 0.5 and 0.5 -0.046 0 in the unit cell  by the Olex solvent mask  two independent molecules of **Ni-8** in  the asymmetric unit |  |

# **3. Additional Polymerization Data and Polymer Characterization**

| # | Cat. | CO feed  [mol%] | Yield  [g] | TOF^b^ | CO incorp.^c^  [mol%] | I/NA/A^d^  [%] | *M*_n_  [kg/mol]^e^ | *M*_w_/*M*_n_^e^ | T_m_ [°C]  (Cryst. [%])^f^ |
| --- | --- | --- | --- | --- | --- | --- | --- | --- | --- |
| 1 | **Ni-1** | 0.6 | 0.23 | 1.3 | 0.12 | 53 / 27 / 20 | 0.9 | 1.2 | 112 (77) |
| 2 | **Ni-1** | 0.8 | 0.18 | 1.0 | 1.01 | 8/ 20 / 72 | 0.8 | 1.2 | 110 (77) |
| 3 | **Ni-2** | 0.6 | 2.47 | 14.1 | 0.30 | 24 / 39 / 37 | 10.9 | 2.1 | 132 (74) |
| 4 | **Ni-2** | 0.8 | 0.92 | 5.3 | 1.14 | 19 / 36 / 45 | 15.7 | 2.0 | 134 (70) |
| 5 | **Ni-2** | 1.0 | 0.04 | 0.2 | - | 0 / 0 / 100 | - | - | - |
| 6 | **Ni-3** | 0.6 | 2.50 | 14.3 | 0.50 | 17 / 36 / 47 | 12.8 | 2.1 | 133 (70) |
| 7 | **Ni-3** | 0.8 | 1.31 | 7.5 | 1.27 | 20 / 35 / 45 | 19.0 | 2.3 | 134 (66) |
| 8 | **Ni-3** | 1.0 | 0.21 | 1.2 | 6.75 | 5 / 19 / 76 | 18.0 | 1.9 | 131 (53) |
| 9 | **Ni-3** | 1.2 | 0.05 | 0.3 | - | 0 / 0 / 100 | - | - | - |
| 10 | **Ni-4** | 0.6 | 3.16 | 18.1 | 0.57 | 29 / 41 / 30 | 22.9 | 2.0 | 135 (67) |
| 11 | **Ni-4** | 0.8 | 1.35 | 7.7 | 1.32 | 21 / 39 / 40 | 26.2 | 2.2 | 134 (67) |
| 12 | **Ni-4** | 1.0 | 0.18 | 1.0 | 7.44 | 6 / 23 / 71 | 24.8 | 1.8 | 133 (52) |
| 13 | **Ni-4** | 1.2 | 0.04 | 0.2 | - | 0 / 0 / 100 | - | - | - |
| 14 | **Ni-5** | 0.6 | 2.81 | 16.1 | 0.55 | 17 / 35 / 48 | 13.6 | 1.9 | 133 (69) |
| 15 | **Ni-5** | 0.8 | 1.19 | 6.8 | 1.46 | 18 / 36 / 46 | 19.2 | 2.1 | 133 (69) |
| 16 | **Ni-5** | 1.0 | 0.41 | 1.5 | 3.78 | 6 / 19 / 75 | 19.5 | 1.8 | 141 (65) |
| 17 | **Ni-5** | 1.2 | 0.23 | 1.3 | 8.55 | 3 / 14 / 83 | 19.6 | 1.7 | 133 (55) |
| 18 | **Ni-5** | 1.4 | 0.04 | 0.2 | - | 0 / 0 / 100 | - | - | - |
| 19 | **Ni-6** | 0.6 | 3.72 | 21.3 | 0.61 | 25 / 38 / 37 | 25.0 | 1.8 | 134 (68) |
| 20 | **Ni-6** | 0.8 | 1.79 | 10.2 | 1.23 | 25 / 42 / 33 | 28.3 | 2.1 | 133 (66) |
| 21 | **Ni-6** | 1.0 | 1.08 | 6.2 | 2.70 | 14 / 33 / 53 | 27.7 | 3.0 | 132 (64) |
| 22 | **Ni-6** | 1.2 | 0.34 | 1.9 | 9.07 | 5 / 20 / 75 | 26.2 | 3.3 | 135 (55) |
| 23 | **Ni-6** | 1.4 | 0.04 | 0.6 | - | 0 / 0 / 100 | - | - | - |
| 24 | **Ni-7** | 0.6 | 4.73 | 27.0 | 0.57 | 30 / 41 / 29 | 33.0 | 1.7 | 134 (65) |
| 25 | **Ni-7** | 0.8 | 4.09 | 23.4 | 0.82 | 34 / 40 / 26 | 33.9 | 1.7 | 134 (64) |
| 26 | **Ni-7** | 1.0 | 2.68 | 15.3 | 1.38 | 18 / 36 / 46 | 35.0 | 2.2 | 135 (59) |
| 27 | **Ni-7** | 1.2 | 1.52 | 8.7 | 2.20 | 14 / 30 / 56 | 36.6 | 2.0 | 135 (57) |
| 28 | **Ni-7** | 1.4 | 1.32 | 7.5 | 3.01 | 9 / 25 / 66 | 35.4 | 2.2 | 135 (58) |
| 29 | **Ni-7** | 1.5 | 0.63 | 3.6 | 5.60 | 6 / 23 / 71 | 39.1 | 2.6 | 134 (53) |
| 30 | **Ni-8** | 0.6 | 2.72 | 15.5 | 0.55 | 28 / 39 / 33 | 35.1 | 2.4 | 136 (69) |
| 31 | **Ni-8** | 0.8 | 0.97 | 5.5 | 1.30 | 35 / 39 / 26 | 63.5 | 1.9 | 135 (61) |
| 32 | **Ni-8** | 1.0 | 0.67 | 3.8 | 2.50 | 20 / 39 / 41 | 70.0 | 2.5 | 137 (59) |
| 33 | **Ni-8** | 1.2 | 0.03 | 0.2 | - | 0 / 0 / 100 | - | - | - |
| 34 | **Ni-9** | 0.6 | 4.45 | 25.4 | 0.60 | 39 / 38 / 23 | 20.4 | 2.5 | 132 (64) |
| 35 | **Ni-9** | 0.8 | 4.37 | 25.0 | 0.80 | 39 / 48 / 13 | 24.1 | 2.6 | 132 (65) |
| 36 | **Ni-9** | 1.0 | 1.89 | 10.8 | 1.26 | 32 / 36 / 32 | 45.4 | 1.8 | 133 (60) |
| 37 | **Ni-9** | 1.2 | 1.67 | 9.5 | 1.95 | 18 / 33 / 49 | 44.3 | 2.1 | 133 (59) |
| 38 | **Ni-9** | 1.4 | 1.52 | 8.7 | 2.78 | 12 / 30 / 58 | 45.7 | 2.3 | 134 (57) |
| 39 | **Ni-9** | 1.5 | 1.34 | 7.7 | 3.20 | 8 / 25 / 67 | 46.7 | 2.7 | 133 (56) |
| a) polymerization conditions: 100 mL toluene, 100 °C, 5 μmol precatalyst, 1000 rpm, 10 bar, 75 minutes; b) turn over frequency in 10^3^ mol[monomer] mol^-1^[Ni] h^-1^; c) CO incorporation ratio determined by ^1^H NMR; d) ratio of isolated, non-alternating and alternating keto groups determined by ^1^H; e) determined by gel permeation chromatography in 1,2-dichlorobenzene at 160 °C against linear calibration versus polyethylene standards; f) determined by differential scanning calorimetry (10 K min^-1^), data from second heating cycle. | | | | | | | | | |

**Table S4.** Copolymerization of ethylene with CO catalyzed by various Ni complexes.^a^

## **3.1 NMR analysis**

**Figure S25.** ^1^H NMR spectrum of keto-polyethylene obtained with **Ni-1** (Table S4, entry 1).

**Figure S26.** ^1^H NMR spectrum of keto-polyethylene obtained with **Ni-1** (Table S4, entry 2).

**Figure S27.** ^1^H NMR spectrum of keto-polyethylene obtained with **Ni-2** (Table S4, entry 3).

**Figure S28.** ^1^H NMR spectrum of keto-polyethylene obtained with **Ni-2** (Table S4, entry 4).

**Figure S29.** ^1^H NMR spectrum of keto-polyethylene obtained with **Ni-3** (Table S4, entry 6).

**Figure S30.** ^1^H NMR spectrum of keto-polyethylene obtained with **Ni-3** (Table S4, entry 7).

**Figure S31.** ^1^H NMR spectrum of keto-polyethylene obtained with **Ni-3** (Table S4, entry 8).

**Figure S32.** ^1^H NMR spectrum of keto-polyethylene obtained with **Ni-4** (Table S4, entry 10).

**Figure S33.** ^1^H NMR spectrum of keto-polyethylene obtained with **Ni-4** (Table S4, entry 11).

**Figure S34.** ^1^H NMR spectrum of keto-polyethylene obtained with **Ni-4** (Table S4, entry 12).

**Figure S35.** ^1^H NMR spectrum of keto-polyethylene obtained with **Ni-5** (Table S4, entry 14).

**Figure S36.** ^1^H NMR spectrum of keto-polyethylene obtained with **Ni-5** (Table S4, entry 15).

**Figure S37.** ^1^H NMR spectrum of keto-polyethylene obtained with **Ni-5** (Table S4, entry 16).

**Figure S38.** ^1^H NMR spectrum of keto-polyethylene obtained with **Ni-5** (Table S4, entry 17).

**Figure S39.** ^1^H NMR spectrum of keto-polyethylene obtained with **Ni-6** (Table S4, entry 19).

**Figure S40.** ^1^H NMR spectrum of keto-polyethylene obtained with **Ni-6** (Table S4, entry 20).

**Figure S41.** ^1^H NMR spectrum of keto-polyethylene obtained with **Ni-6** (Table S4, entry 21).

**Figure S42.** ^1^H NMR spectrum of keto-polyethylene obtained with **Ni-6** (Table S4, entry 22).

**Figure S43.** ^1^H NMR spectrum of keto-polyethylene obtained with **Ni-7** (Table S4, entry 24).

**Figure S44.** ^13^C NMR spectrum of keto-polyethylene obtained with **Ni-7** (Table S4, entry 24).

**Figure S45.** ^1^H NMR spectrum of keto-polyethylene obtained with **Ni-7** (Table S4, entry 25).

**Figure S46.** ^1^H NMR spectrum of keto-polyethylene obtained with **Ni-7** (Table S4, entry 26).

**Figure S47.** ^1^H NMR spectrum of keto-polyethylene obtained with **Ni-7** (Table S4, entry 27).

**Figure S48.** ^1^H NMR spectrum of keto-polyethylene obtained with **Ni-7** (Table S4, entry 28).

**Figure S49.** ^1^H NMR spectrum of keto-polyethylene obtained with **Ni-7** (Table S4, entry 29).

**Figure S50.** ^1^H NMR spectrum of keto-polyethylene obtained with **Ni-8** (Table S4, entry 30).

**Figure S51.** ^1^H NMR spectrum of keto-polyethylene obtained with **Ni-8** (Table S4, entry 31).

**Figure S52.** ^1^H NMR spectrum of keto-polyethylene obtained with **Ni-8** (Table S4, entry 32).

**Figure S53.** ^1^H NMR spectrum of keto-polyethylene obtained with **Ni-9** (Table S4, entry 34).

**Figure S54.** ^1^H NMR spectrum of keto-polyethylene obtained with **Ni-9** (Table S4, entry 35).

**Figure S55.** ^1^H NMR spectrum of keto-polyethylene obtained with **Ni-9** (Table S4, entry 36).

**Figure S56.** ^1^H NMR spectrum of keto-polyethylene obtained with **Ni-9** (Table S4, entry 37).

**Figure S57.** ^1^H NMR spectrum of keto-polyethylene obtained with **Ni-9** (Table S4, entry 38).

**Figure S58.** ^1^H NMR spectrum of keto-polyethylene obtained with **Ni-9** (Table S4, entry 39).

## **3.2 DSC traces**

**Figure S59.** DSC traces of keto-polyethylene obtained with **Ni-1** (Table S4, entry 1, red curve, 1^st^ heating; black curve, 1^st^ cooling; blue curve: 2^nd^ heating).

**Figure S60.** DSC traces of keto-polyethylene obtained with **Ni-1** (Table S4, entry 2, red curve, 1^st^ heating; black curve, 1^st^ cooling; blue curve: 2^nd^ heating).

**Figure S61.** DSC traces of keto-polyethylene obtained with **Ni-2** (Table S4, entry 3, red curve, 1^st^ heating; black curve, 1^st^ cooling; blue curve: 2^nd^ heating).

**Figure S62.** DSC traces of keto-polyethylene obtained with **Ni-2** (Table S4, entry 4, red curve, 1^st^ heating; black curve, 1^st^ cooling; blue curve: 2^nd^ heating).

**Figure S63.** DSC traces of keto-polyethylene obtained with **Ni-3** (Table S4, entry 6, red curve, 1^st^ heating; green curve, 1^st^ cooling; blue curve: 2^nd^ heating).

**Figure S64.** DSC traces of keto-polyethylene obtained with **Ni-3** (Table S4, entry 7, red curve, 1^st^ heating; green curve, 1^st^ cooling; blue curve: 2^nd^ heating).

**Figure S65.** DSC traces of keto-polyethylene obtained with **Ni-3** (Table S4, entry 8, red curve, 1^st^ heating; green curve, 1^st^ cooling; blue curve: 2^nd^ heating).

**Figure S66.** DSC traces of keto-polyethylene obtained with **Ni-4** (Table S4, entry 10, red curve, 1^st^ heating; green curve, 1^st^ cooling; blue curve: 2^nd^ heating).

**Figure S67.** DSC traces of keto-polyethylene obtained with **Ni-4** (Table S4, entry 11, red curve, 1^st^ heating; green curve, 1^st^ cooling; blue curve: 2^nd^ heating).

**Figure S68.** DSC traces of keto-polyethylene obtained with **Ni-4** (Table S4, entry 12, red curve, 1^st^ heating; green curve, 1^st^ cooling; blue curve: 2^nd^ heating).

**Figure S69.** DSC traces of keto-polyethylene obtained with **Ni-5** (Table S4, entry 14, red curve, 1^st^ heating; green curve, 1^st^ cooling; blue curve: 2^nd^ heating).

**Figure S70.** DSC traces of keto-polyethylene obtained with **Ni-5** (Table S4, entry 15, red curve, 1^st^ heating; green curve, 1^st^ cooling; blue curve: 2^nd^ heating).

**Figure S71.** DSC traces of keto-polyethylene obtained with **Ni-5** (Table S4, entry 16, red curve, 1^st^ heating; green curve, 1^st^ cooling; blue curve: 2^nd^ heating).

**Figure S72.** DSC traces of keto-polyethylene obtained with **Ni-5** (Table S4, entry 17, red curve, 1^st^ heating; green curve, 1^st^ cooling; blue curve: 2^nd^ heating).

**Figure S73.** DSC traces of keto-polyethylene obtained with **Ni-6** (Table S4, entry 19, red curve, 1^st^ heating; green curve, 1^st^ cooling; blue curve: 2^nd^ heating).

**Figure S74.** DSC traces of keto-polyethylene obtained with **Ni-6** (Table S4, entry 20, red curve, 1^st^ heating; green curve, 1^st^ cooling; blue curve: 2^nd^ heating).

**Figure S75.** DSC traces of keto-polyethylene obtained with **Ni-6** (Table S4, entry 21, red curve, 1^st^ heating; green curve, 1^st^ cooling; blue curve: 2^nd^ heating).

**Figure S76.** DSC traces of keto-polyethylene obtained with **Ni-6** (Table S4, entry 22, red curve, 1^st^ heating; green curve, 1^st^ cooling; blue curve: 2^nd^ heating).

**Figure S77.** DSC traces of keto-polyethylene obtained with **Ni-7** (Table S4, entry 24, red curve, 1^st^ heating; black curve, 1^st^ cooling; blue curve: 2^nd^ heating).

**Figure S78.** DSC traces of keto-polyethylene obtained with **Ni-7** (Table S4, entry 25, red curve, 1^st^ heating; black curve, 1^st^ cooling; blue curve: 2^nd^ heating).

**Figure S79.** DSC traces of keto-polyethylene obtained with **Ni-7** (Table S4, entry 26, red curve, 1^st^ heating; black curve, 1^st^ cooling; blue curve: 2^nd^ heating).

**Figure S80.** DSC traces of keto-polyethylene obtained with **Ni-7** (Table S4, entry 27, red curve, 1^st^ heating; black curve, 1^st^ cooling; blue curve: 2^nd^ heating).

**Figure S81.** DSC traces of keto-polyethylene obtained with **Ni-7** (Table S4, entry 28, red curve, 1^st^ heating; black curve, 1^st^ cooling; blue curve: 2^nd^ heating).

**Figure S82.** DSC traces of keto-polyethylene obtained with **Ni-7** (Table S4, entry 29, red curve, 1^st^ heating; black curve, 1^st^ cooling; blue curve: 2^nd^ heating).

**Figure S83.** DSC traces of keto-polyethylene obtained with **Ni-8** (Table S4, entry 30, red curve, 1^st^ heating; black curve, 1^st^ cooling; blue curve: 2^nd^ heating).

**Figure S84.** DSC traces of keto-polyethylene obtained with **Ni-8** (Table S4, entry 31, red curve, 1^st^ heating; black curve, 1^st^ cooling; blue curve: 2^nd^ heating).

**Figure S85.** DSC traces of keto-polyethylene obtained with **Ni-8** (Table S4, entry 32, red curve, 1^st^ heating; black curve, 1^st^ cooling; blue curve: 2^nd^ heating).

**Figure S86.** DSC traces of keto-polyethylene obtained with **Ni-9** (Table S4, entry 34, red curve, 1^st^ heating; black curve, 1^st^ cooling; blue curve: 2^nd^ heating).

**Figure S87.** DSC traces of keto-polyethylene obtained with **Ni-9** (Table S4, entry 35, red curve, 1^st^ heating; black curve, 1^st^ cooling; blue curve: 2^nd^ heating).

**Figure S88.** DSC traces of keto-polyethylene obtained with **Ni-9** (Table S4, entry 36, red curve, 1^st^ heating; black curve, 1^st^ cooling; blue curve: 2^nd^ heating).

**Figure S89.** DSC traces of keto-polyethylene obtained with **Ni-9** (Table S4, entry 37, red curve, 1^st^ heating; black curve, 1^st^ cooling; blue curve: 2^nd^ heating).

**Figure S90.** DSC traces of keto-polyethylene obtained with **Ni-9** (Table S4, entry 38, red curve, 1^st^ heating; black curve, 1^st^ cooling; blue curve: 2^nd^ heating).

**Figure S91.** DSC traces of keto-polyethylene obtained with **Ni-9** (Table S4, entry 39, red curve, 1^st^ heating; black curve, 1^st^ cooling; blue curve: 2^nd^ heating).

## **3.3 SEC traces**

**Figure S92.** SEC trace of keto-polyethylene obtained with **Ni-1** (Table S4, entry 1).

**Figure S93.** SEC trace of keto-polyethylene obtained with **Ni-1** (Table S4, entry 2).

**Figure S94.** SEC trace of keto-polyethylene obtained with **Ni-2** (Table S4, entry 3).

**Figure S95.** SEC trace of keto-polyethylene obtained with **Ni-2** (Table S4, entry 4).

**Figure S96.** SEC trace of keto-polyethylene obtained with **Ni-3** (Table S4, entry 6).

**Figure S97.** SEC trace of keto-polyethylene obtained with **Ni-3** (Table S4, entry 7).

**Figure S98.** SEC trace of keto-polyethylene obtained with **Ni-3** (Table S4, entry 8).

**Figure S99.** SEC trace of keto-polyethylene obtained with **Ni-4** (Table S4, entry 10).

**Figure S100.** SEC trace of keto-polyethylene obtained with **Ni-4** (Table S4, entry 11).

**Figure S101.** SEC trace of keto-polyethylene obtained with **Ni-4** (Table S4, entry 12).

**Figure S102.** SEC trace of keto-polyethylene obtained with **Ni-5** (Table S4, entry 14).

**Figure S103.** SEC trace of keto-polyethylene obtained with **Ni-5** (Table S4, entry 15).

**Figure S104.** SEC trace of keto-polyethylene obtained with **Ni-5** (Table S4, entry 16).

**Figure S105.** SEC trace of keto-polyethylene obtained with **Ni-5** (Table S4, entry 17).

**Figure S106.** SEC trace of keto-polyethylene obtained with **Ni-6** (Table S4, entry 19).

**Figure S107.** SEC trace of keto-polyethylene obtained with **Ni-6** (Table S4, entry 20).

**Figure S108.** SEC trace of keto-polyethylene obtained with **Ni-6** (Table S4, entry 21).

**Figure S109.** SEC trace of keto-polyethylene obtained with **Ni-6** (Table S4, entry 22).

**Figure S110.** SEC trace of keto-polyethylene obtained with **Ni-7** (Table S4, entry 24).

**Figure S111.** SEC trace of keto-polyethylene obtained with **Ni-7** (Table S4, entry 25).

**Figure S112.** SEC trace of keto-polyethylene obtained with **Ni-7** (Table S4, entry 26).

**Figure S113.** SEC trace of keto-polyethylene obtained with **Ni-7** (Table S4, entry 27).

**Figure S114.** SEC trace of keto-polyethylene obtained with **Ni-7** (Table S4, entry 28).

**Figure S115.** SEC trace of keto-polyethylene obtained with **Ni-7** (Table S4, entry 29).

**Figure S116.** SEC trace of keto-polyethylene obtained with **Ni-8** (Table S4, entry 30).

**Figure S117.** SEC trace of keto-polyethylene obtained with **Ni-8** (Table S4, entry 31).

**Figure S118.** SEC trace of keto-polyethylene obtained with **Ni-8** (Table S4, entry 32).

**Figure S119.** SEC trace of keto-polyethylene obtained with **Ni-9** (Table S4, entry 34).

**Figure S120.** SEC trace of keto-polyethylene obtained with **Ni-9** (Table S4, entry 35).

**Figure S121.** SEC trace of keto-polyethylene obtained with **Ni-9** (Table S4, entry 36).

**Figure S122.** SEC trace of keto-polyethylene obtained with **Ni-9** (Table S4, entry 37).

**Figure S123.** SEC trace of keto-polyethylene obtained with **Ni-9** (Table S4, entry 38).

**Figure S124.** SEC trace of keto-polyethylene obtained with **Ni-9** (Table S4, entry 39).

## **3.4 FT-IR spectra**

**Figure S125.** Typical FT-IR spectrum of the obtained copolymers (grey line: Table S4, entry 5 obtained by **Ni-2**; orange line: Table S4, entry 24 obtained by **Ni-7**; blue line: Table S4, entry 26 obtained by **Ni-7**; red line: Table S4, entry 27 obtained by **Ni-7**; green line: Table S4, entry 29 obtained by **Ni-7**. Note: for clarity the absorbance of the alternating copolymer (grey line) was reduced.

## **3.5 WAXS data**

**Figure S126.** WAXS data of copolymers and HDPE with full hkl-assignment of the observable reflexes in the 2θ range of 16-46^o^. Reflexes correspond to the orthorhombic unit cell of polyethylene. (orange line: commercial HDPE from Lyondellbasell; red line: reference compound from ADMET copolymerization/hydrogenation; ^18^ green line: Table S4, entry 27 obtained by **Ni-7**; blue line: Table S4, entry 29 obtained by **Ni-7**).

# **4. Mass Flow Profiles of Ethylene/CO Polymerizations**

**Figure S127.** Ethylene and CO uptake of copolymerization catalyzed by **Ni-1** (Table S4, entry 1).

**Figure S128.** Ethylene and CO uptake of copolymerization catalyzed by **Ni-1** (Table S4, entry 2).

**Figure S129.** Ethylene and CO uptake of copolymerization catalyzed by **Ni-2** (Table S4, entry 3).

**Figure S130.** Ethylene and CO uptake of copolymerization catalyzed by **Ni-2** (Table S4, entry 4).

**Figure S131.** Ethylene and CO uptake of copolymerization catalyzed by **Ni-3** (Table S4, entry 6).

**Figure S132.** Ethylene and CO uptake of copolymerization catalyzed by **Ni-3** (Table S4, entry 7).

**Figure S133.** Ethylene and CO uptake of copolymerization catalyzed by **Ni-3** (Table S4, entry 8).

**Figure S134.** Ethylene and CO uptake of copolymerization catalyzed by **Ni-4** (Table S4, entry 10).

**Figure S135.** Ethylene and CO uptake of copolymerization catalyzed by **Ni-4** (Table S4, entry 11).

**Figure S136.** Ethylene and CO uptake of copolymerization catalyzed by **Ni-4** (Table S4, entry 12).

**Figure S137.** Ethylene and CO uptake of copolymerization catalyzed by **Ni-5** (Table S4, entry 14).

**Figure S138.** Ethylene and CO uptake of copolymerization catalyzed by **Ni-5** (Table S4, entry 15).

**Figure S139.** Ethylene and CO uptake of copolymerization catalyzed by **Ni-5** (Table S4, entry 16).

**Figure S140.** Ethylene and CO uptake of copolymerization catalyzed by **Ni-5** (Table S4, entry 17).

**Figure S141.** Ethylene and CO uptake of copolymerization catalyzed by **Ni-6** (Table S4, entry 19).

**Figure S142.** Ethylene and CO uptake of copolymerization catalyzed by **Ni-6** (Table S4, entry 20).

**Figure S143.** Ethylene and CO uptake of copolymerization catalyzed by **Ni-6** (Table S4, entry 21).

**Figure S144.** Ethylene and CO uptake of copolymerization catalyzed by **Ni-6** (Table S4, entry 22).

**Figure S145.** Ethylene and CO uptake of copolymerization catalyzed by **Ni-7** (Table S4, entry 24).

**Figure S146.** Ethylene and CO uptake of copolymerization catalyzed by **Ni-7** (Table S4, entry 25).

**Figure S147.** Ethylene and CO uptake of copolymerization catalyzed by **Ni-7** (Table S4, entry 26).

**Figure S148.** Ethylene and CO uptake of copolymerization catalyzed by **Ni-7** (Table S4, entry 27).

**Figure S149.** Ethylene and CO uptake of copolymerization catalyzed by **Ni-7** (Table S4, entry 28).

**Figure S150.** Ethylene and CO uptake of copolymerization catalyzed by **Ni-7** (Table S4, entry 29).

**Figure S151.** Ethylene and CO uptake of copolymerization catalyzed by **Ni-8** (Table S4, entry 30).

**Figure S152.** Ethylene and CO uptake of copolymerization catalyzed by **Ni-8** (Table S4, entry 31).

**Figure S153.** Ethylene and CO uptake of copolymerization catalyzed by **Ni-8** (Table S4, entry 32).

**Figure S154.** Ethylene and CO uptake of copolymerization catalyzed by **Ni-9** (Table S4, entry 34).

**Figure S155.** Ethylene and CO uptake of copolymerization catalyzed by **Ni-9** (Table S4, entry 35).

**Figure S156.** Ethylene and CO uptake of copolymerization catalyzed by **Ni-9** (Table S4, entry 36).

**Figure S157.** Ethylene and CO uptake of copolymerization catalyzed by **Ni-9** (Table S4, entry 37).

**Figure S158.** Ethylene and CO uptake of copolymerization catalyzed by **Ni-9** (Table S4, entry 38).

**Figure S159.** Ethylene and CO uptake of copolymerization catalyzed by **Ni-9** (Table S4, entry 39).

# **5. Density Functional Theory (DFT)**

## **5.1 Computational details**

All stationary points energies involved in both alternating and non-alternating mechanisms of ethylene/CO copolymerization were calculated, using the **1-cycle5-T** intermediate as the reference point. Geometry optimizations were carried out with the Gaussian16 package ^19^ at the GGA BP86 level. ^20, 21, 22^ The electronic structures were modelled with the 6-31G(d) basis set for H, C, N, F, and O atoms,^23^ while Ni was treated with the quasi-relativistic LANL2DZ effective core potential (ECP). ^24, 25, 26^ A conformers search was performed with CREST Version 3.0, ^27, 28^ at the GFN2-xTB level ^29^ for each stationary point. The automatic principal component analysis (PCA) extension in CREST was then used to filter the geometries selected for DFT optimization. All optimized structures were verified as minima or transition states through vibrational frequency analyses, and no symmetry constraints were applied. Free energies were obtained from single-point calculations performed on the BP86/6-31G(d)/LANL2DZ-optimized geometries, using the M06 functional ^30^ with the triple-ζ TZVP basis set for main-group elements ^31^ and the LANL2DZ ECP for Ni. Solvent effects were modelled with the PCM approach ^32, 33^ using toluene as the solvent. Thermal corrections from the gas-phase frequency calculations at the BP86/6-31G(d)/LANL2DZ ECP level were added to the (M06/TZVP/LANL2DZ ECP) electronic energies in solution. QTAIM analyses, including the search for bond critical points, were performed using Multiwfn.^34^ NCI analyses were carried out using NCIPLOT 4.0,^35^ following the reduced density gradient approach for the visualization of non-covalent interactions.

## **5.2 Reaction pathways**

**Figure S160.** Free energies (ΔG_tol_ in kcal/mol) of the key steps for non-alternating (in green) and alternating ethylene and carbon monoxide copolymerization (in grey) with **Ni-2**. Alternative pathways in red.

**Figure S161.** Free energies (ΔG_tol_ in kcal/mol) of the key steps for non-alternating (in green) and alternating ethylene and carbon monoxide copolymerization (in grey) with **Ni-7**. Alternative pathways in red.

**Figure S162.** Free energies (ΔG_tol_ in kcal/mol) of the key steps for non-alternating (in green) and alternating ethylene and carbon monoxide copolymerization (in grey) with **Ni-9**. Alternative pathways in red.

## **5.3 Steric and NCI/AIM analysis**

**Figure S163.** Geometries and steric maps (xy plane) of **1-cycle5-T** intermediate of **Ni-2**.

**Figure S164.** Geometries and steric maps (xy plane) of **1-cycle5-T** intermediate of **Ni-7**.

**Figure S165.** Geometries and steric maps (xy plane) of **1-cycle5-T** intermediate of **Ni-9**.

**Figure S166.** NCI and AIM analysis of **1-cycle5-T** intermediate of **Ni-2**.

**Figure S167.** NCI and AIM analysis of **1-cycle5-T** intermediate of **Ni-7**.

**Figure S168.** NCI and AIM analysis of **TS-Isom-1** transition state of **Ni-2**.

**Figure S169.** NCI and AIM analysis of **TS-Isom-1** transition state of **Ni-7**.

**Figure S170.** NCI and AIM analysis of **TS-Isom-2** transition state of **Ni-2**.

**Figure S171.** NCI and AIM analysis of **TS-Isom-2** transition state of **Ni-7**.

## **5.4 Additional comments**

In **Ni-7**, CO insertion via **TS-1-Ins-CO-T** is disfavored by a more electronegative Ni center (q_Ni−2_ = 0.26 < q_Ni−7_ = 0.27 < q_Ni−9_ = 0.30 < q_Ni−8_= 0.31). Furthermore, the barrier for **Ni-7** is higher than for **Ni-2** due to greater steric hindrance (NE %V_Bur_ = 63.6 in **Ni-2** vs NE %V_Bur_ = 66.7 in **Ni-7**).

Isomerization barriers are higher for the phosphine-phenolate series than for the imidates due to increased steric crowding. This is quantitatively supported by the higher buried volume of **Ni-9** (%V_Bu_ = 51.7) compared to **Ni-7** (%V_Bur_ = 45.8), which hinders the geometric reorganization required for the *cis/trans* isomerization.

## **5.5 Cartesian coordinates**

### **5.5.1 Geometries of Ni-2**

**1-cycle5-T**

SCF Done: -2737.29198367 A.U.

Ni	1.572489	-1.894380	-0.129175

P	1.529203	0.240130	-0.038629

O	-0.367885	-1.741083	0.000215

O	2.371127	-1.033436	2.555826

O	1.595506	3.162939	0.506767

O	4.300148	1.485417	-0.102378

O	0.606535	0.390940	-2.832271

N	-0.143003	0.607691	-0.100226

C	-0.838214	-0.524082	-0.041171

C	-2.336997	-0.402584	-0.018549

C	-2.956414	0.861271	-0.067488

C	-4.355680	0.961789	-0.050496

C	-5.153580	-0.193110	0.019414

C	-4.534659	-1.452238	0.069848

C	-3.134508	-1.559972	0.049318

C	2.020791	1.081981	1.531566

C	2.325856	0.333237	2.696113

C	2.556817	0.973901	3.933433

H	2.800479	0.394472	4.826846

C	2.465489	2.368190	4.011106

H	2.640435	2.865068	4.972332

C	2.146102	3.140015	2.886593

H	2.070050	4.226554	2.972823

C	1.921084	2.494329	1.654085

C	2.394976	1.066497	-1.443107

C	3.699814	1.615311	-1.330613

C	4.333458	2.234829	-2.427176

H	5.329727	2.671171	-2.325166

C	3.676395	2.274233	-3.663473

H	4.165267	2.756618	-4.517666

C	2.419126	1.683856	-3.833803

H	1.938857	1.695329	-4.814813

C	1.794818	1.058871	-2.732997

C	3.462137	-2.063191	-0.474378

H	4.122278	-1.302404	-0.028226

H	3.527432	-1.978068	-1.578295

C	3.846928	-3.485090	-0.013651

H	4.739383	-3.923115	-0.512893

H	4.094744	-3.477712	1.070642

C	2.651489	-4.389688	-0.153457

C	2.780873	-5.890006	-0.231866

H	3.431592	-6.270465	0.577522

H	1.791596	-6.369691	-0.178684

H	3.269828	-6.169774	-1.185324

O	1.516649	-3.853639	-0.195219

H	-2.639870	-2.534674	0.081271

H	-6.243673	-0.112128	0.034815

H	-2.320947	1.750181	-0.114098

C	-5.010987	2.317241	-0.175811

C	-5.376633	-2.699086	0.211009

F	-6.637927	-2.522705	-0.280625

F	-5.510287	-3.070079	1.519480

F	-4.826210	-3.759750	-0.445052

F	-4.266734	3.299319	0.407243

F	-5.180018	2.674635	-1.484921

F	-6.247540	2.342307	0.401678

C	2.427193	-1.824619	3.744097

H	3.400562	-1.719424	4.263211

H	1.609431	-1.564433	4.443068

H	2.302233	-2.865463	3.408389

C	1.337450	4.564260	0.591233

H	2.242834	5.131200	0.885647

H	1.031529	4.867036	-0.421269

H	0.520381	4.782126	1.305576

C	-0.067867	0.386087	-4.090714

H	0.536704	-0.109834	-4.875353

H	-0.992143	-0.186462	-3.924026

H	-0.323864	1.412622	-4.417407

C	5.586583	2.073644	0.086801

H	5.845884	1.879128	1.138423

H	6.347355	1.611691	-0.572693

H	5.565001	3.166490	-0.090500

**TS-1-Coor-CO-T**

SCF Done: -2850.59766641 A.U.

Ni	-1.383169	-1.670667	0.735448

P	-1.404982	0.294703	-0.344069

O	0.549779	-1.283752	0.835497

O	-4.137671	1.261962	-1.299036

O	-0.378344	-1.289471	-2.575275

O	-1.534071	2.999976	-1.613168

O	-2.280714	0.716666	2.452825

N	0.265544	0.610018	-0.546077

C	0.984221	-0.262091	0.142600

C	2.476229	-0.068718	0.149579

C	3.062542	0.994797	-0.563413

C	4.455008	1.163732	-0.556850

C	5.277644	0.279772	0.162181

C	4.691398	-0.778198	0.874334

C	3.298286	-0.955893	0.868483

C	-2.195061	0.103101	-1.996688

C	-3.482630	0.612985	-2.313713

C	-4.044354	0.433032	-3.594597

H	-5.026421	0.845217	-3.837285

C	-3.335506	-0.295952	-4.557794

H	-3.770432	-0.435388	-5.554047

C	-2.095609	-0.874047	-4.262084

H	-1.574999	-1.467650	-5.016814

C	-1.542747	-0.696103	-2.976502

C	-1.957831	1.877156	0.417829

C	-1.881402	3.103270	-0.295032

C	-2.149022	4.331416	0.342449

H	-2.094794	5.273244	-0.208643

C	-2.480097	4.334129	1.704307

H	-2.684835	5.288064	2.203894

C	-2.542545	3.147606	2.443747

H	-2.787328	3.178578	3.507580

C	-2.271930	1.920143	1.799911

C	-3.341642	-1.864113	0.578543

H	-3.545742	-1.980361	-0.500317

H	-3.737901	-0.891770	0.915918

C	-3.998950	-2.997798	1.367522

C	-3.674851	-3.052244	2.859440

C	-4.591651	-3.913819	3.723603

H	-5.573770	-3.414705	3.835070

H	-4.785315	-4.893406	3.248682

H	-4.149017	-4.059077	4.721090

O	-2.721955	-2.446935	3.352076

H	2.831532	-1.779946	1.414626

H	6.362469	0.414726	0.167195

H	2.408681	1.678805	-1.111653

C	5.079715	2.264361	-1.382913

C	5.557176	-1.698806	1.704177

F	6.838981	-1.749936	1.238550

F	5.073510	-2.972913	1.715627

F	5.622885	-1.287600	3.004762

F	4.277611	3.364063	-1.454575

F	6.281021	2.664182	-0.874492

F	5.309336	1.857786	-2.667676

C	-5.411586	1.842373	-1.578286

H	-6.157357	1.072610	-1.857948

H	-5.721855	2.331264	-0.642584

H	-5.347142	2.598480	-2.384653

C	0.345100	-2.078613	-3.521957

H	-0.251876	-2.944022	-3.869831

H	0.661718	-1.473998	-4.393478

H	1.233464	-2.439131	-2.982995

C	-2.438206	0.719198	3.877556

H	-1.643940	1.316923	4.363952

H	-3.429611	1.117324	4.170370

H	-2.359825	-0.335678	4.172598

C	-1.305602	4.203681	-2.346704

H	-0.978087	3.879951	-3.345878

H	-2.229453	4.807896	-2.441227

H	-0.512893	4.817666	-1.877931

C	-1.097033	-3.419829	0.893026

O	-0.857268	-4.556671	0.821344

H	-5.107114	-2.956274	1.264846

H	-3.725298	-3.992989	0.954582

**1-Coor-CO-T**

SCF Done: -2850.60464325 A.U.

Ni	-1.249694	1.866366	-0.519567

P	-1.435067	-0.212999	0.258821

O	0.671854	1.464192	-0.338744

O	-2.008908	2.039386	2.133176

O	-1.720659	-2.676026	1.915053

O	-4.290248	-1.169754	0.734386

O	-0.688605	-1.428251	-2.304861

N	0.186712	-0.759776	0.297634

C	1.000386	0.243720	0.004719

C	2.477883	-0.032240	0.064033

C	3.398360	0.983810	-0.252938

C	4.777847	0.725997	-0.201889

C	5.252685	-0.542144	0.168090

C	4.331974	-1.555050	0.486232

C	2.952608	-1.305366	0.434088

C	-1.906154	-0.328307	2.036746

C	-2.084852	0.847608	2.809606

C	-2.313541	0.774534	4.200732

H	-2.454883	1.680739	4.793601

C	-2.342974	-0.478594	4.823365

H	-2.513161	-0.535597	5.904572

C	-2.148189	-1.660348	4.096377

H	-2.164934	-2.625626	4.607784

C	-1.926188	-1.581169	2.706922

C	-2.433297	-1.419551	-0.711433

C	-3.758610	-1.796050	-0.363236

C	-4.473273	-2.743452	-1.124189

H	-5.483658	-3.043721	-0.837557

C	-3.878487	-3.294308	-2.266661

H	-4.431834	-4.033036	-2.857533

C	-2.603500	-2.896029	-2.684006

H	-2.172329	-3.310630	-3.598015

C	-1.897360	-1.939874	-1.922995

C	-3.199100	2.011278	-0.855971

H	-3.495178	3.058436	-0.678174

C	-0.967648	3.585877	-0.935626

O	-0.698126	4.711824	-1.018461

H	2.223668	-2.084448	0.674174

H	6.327235	-0.739295	0.208382

H	3.017744	1.969563	-0.533180

C	4.831409	-2.904086	0.949725

C	5.755109	1.804448	-0.611738

C	-5.594478	-1.556441	1.171290

H	-5.634420	-2.632770	1.426893

H	-5.789473	-0.957768	2.073435

H	-6.362772	-1.332339	0.405970

C	-0.028609	-2.007049	-3.432930

H	0.937193	-1.485842	-3.504946

H	0.144947	-3.089985	-3.285454

H	-0.602431	-1.849566	-4.367192

C	-2.092226	3.254731	2.884287

H	-1.287651	3.316094	3.641509

H	-1.966599	4.060433	2.146326

H	-3.076817	3.357733	3.379625

C	-1.594587	-3.951400	2.545790

H	-1.370920	-4.658762	1.733469

H	-0.767053	-3.957916	3.280686

H	-2.534178	-4.254533	3.048366

F	3.987427	-3.910746	0.583094

F	4.944173	-2.958285	2.310001

F	6.062658	-3.194737	0.438447

F	5.313728	3.045746	-0.265825

F	6.979660	1.627261	-0.036493

F	5.949472	1.814252	-1.964123

C	-3.520051	1.598358	-2.289429

H	-4.626312	1.514605	-2.421769

H	-3.146832	0.582325	-2.527653

C	-3.071524	2.565053	-3.383550

C	-3.172228	2.055188	-4.819926

H	-3.120036	2.898253	-5.526327

H	-2.322211	1.374345	-5.020368

H	-4.096912	1.473038	-4.985658

H	-3.734939	1.369581	-0.138895

O	-2.662040	3.698593	-3.138894

**TS-1-Ins-CO-T**

SCF Done: -2850.57670726 A.U.

Ni	1.364613	-1.687601	-0.753919

P	1.299554	0.463159	0.045421

O	-0.518221	-1.513513	-0.762981

O	0.071161	0.684520	2.904836

O	4.104935	0.805624	0.445189

O	2.341513	0.241940	-2.723337

O	1.182054	3.423956	0.583211

N	-0.402433	0.677890	0.156400

C	-1.030908	-0.390712	-0.300317

C	-2.534565	-0.350055	-0.308668

C	-3.211956	0.788601	0.167685

C	-4.614289	0.824079	0.169555

C	-5.356386	-0.270433	-0.305775

C	-4.678892	-1.403726	-0.782647

C	-3.275103	-1.448243	-0.783916

C	2.091406	0.835752	1.672025

C	1.420755	0.886510	2.924336

C	2.141110	1.129814	4.116147

H	1.621214	1.184660	5.075395

C	3.529200	1.300170	4.069899

H	4.079432	1.492517	4.998060

C	4.229077	1.211729	2.859082

H	5.315658	1.325379	2.842345

C	3.504048	0.961314	1.679089

C	1.788535	1.851795	-1.068960

C	2.221935	1.572638	-2.389581

C	2.508317	2.611273	-3.300184

H	2.844953	2.392635	-4.316193

C	2.356033	3.940431	-2.885651

H	2.576647	4.750165	-3.590694

C	1.921128	4.256918	-1.592755

H	1.800544	5.302319	-1.299008

C	1.631777	3.211693	-0.689787

C	3.341704	-2.542351	-1.021943

H	3.822531	-2.623732	-2.008183

C	1.728506	-3.221579	-1.485745

O	1.677520	-4.295497	-1.998570

C	3.899230	-3.544653	-0.011868

H	4.988555	-3.395378	0.137247

H	3.793077	-4.580690	-0.396683

C	3.207591	-3.488002	1.353654

C	3.821585	-4.321268	2.468924

H	3.168909	-4.305271	3.354753

H	3.983665	-5.364111	2.138654

H	4.813993	-3.913404	2.740697

H	3.491132	-1.501828	-0.673706

O	2.199689	-2.806909	1.538667

H	-2.738762	-2.329636	-1.145120

H	-6.449108	-0.240115	-0.304038

H	-2.616785	1.632757	0.527611

C	-5.463019	-2.561075	-1.357873

C	-5.328237	2.022307	0.750264

C	2.530864	-0.087546	-4.102394

H	1.744587	0.364601	-4.736060

H	3.526494	0.233267	-4.467454

H	2.457087	-1.184313	-4.154960

C	0.934940	4.767963	0.995542

H	1.861533	5.375254	0.988868

H	0.172643	5.254403	0.356682

H	0.556116	4.692813	2.025644

C	-0.632965	0.693918	4.145805

H	-0.264931	-0.094853	4.830929

H	-0.562654	1.678235	4.649857

H	-1.682743	0.492213	3.885666

C	5.499404	1.093220	0.337150

H	6.112135	0.380233	0.923819

H	5.741864	0.990348	-0.731305

H	5.724542	2.124878	0.668595

F	-6.724359	-2.629340	-0.840296

F	-4.854846	-3.755792	-1.115515

F	-5.596357	-2.451316	-2.713156

F	-4.657621	3.183056	0.502248

F	-6.588910	2.160920	0.247574

F	-5.452588	1.922222	2.108749

**1-cycle6-C**

SCF Done: -2850.59281106 A.U.

Ni	1.442536	-1.242873	-1.361381

P	1.438439	0.270562	0.391258

O	-0.440028	-1.027379	-1.228756

O	0.479920	-1.499819	2.601616

O	4.161226	1.135763	1.268147

O	2.022913	1.162994	-2.329787

O	1.520633	2.812749	2.077887

N	-0.259165	0.395772	0.667551

C	-0.919582	-0.278765	-0.255559

C	-2.421795	-0.206427	-0.218647

C	-3.189869	-0.876870	-1.188706

C	-4.592372	-0.810300	-1.146813

C	-5.242118	-0.080684	-0.139056

C	-4.472942	0.587759	0.828985

C	-3.072021	0.529734	0.790710

C	2.265599	-0.064976	2.004907

C	1.678091	-0.941337	2.958694

C	2.322121	-1.229489	4.181667

H	1.848710	-1.880150	4.920658

C	3.586982	-0.687127	4.437261

H	4.088529	-0.911474	5.385662

C	4.233253	0.116090	3.488595

H	5.233632	0.505152	3.692699

C	3.579183	0.404835	2.273981

C	1.823849	2.003878	-0.118615

C	2.025324	2.259803	-1.498334

C	2.206062	3.573817	-1.979975

H	2.375168	3.761187	-3.043053

C	2.165990	4.640800	-1.074728

H	2.304044	5.663643	-1.443978

C	1.943670	4.429754	0.293049

H	1.902624	5.281815	0.975998

C	1.765016	3.112909	0.765267

C	1.200101	-2.364936	-2.853686

O	0.694757	-1.994664	-3.903123

C	1.771373	-3.801330	-2.724084

H	2.330512	-4.040112	-3.649042

H	0.899176	-4.479773	-2.688179

C	2.650580	-4.023708	-1.472073

H	2.007355	-3.999726	-0.568583

H	3.146707	-5.009302	-1.506661

C	3.663840	-2.909455	-1.309884

C	5.126267	-3.219764	-1.137912

H	5.710006	-2.295798	-1.010150

H	5.273676	-3.878878	-0.260327

H	5.502577	-3.779898	-2.016130

O	3.284404	-1.711679	-1.325515

C	-5.161499	1.318504	1.957973

C	-5.402859	-1.480545	-2.232310

C	1.330842	3.893456	2.990280

H	1.094851	3.423263	3.956721

H	2.246859	4.508145	3.095906

H	0.489590	4.542995	2.679905

C	5.445696	1.708582	1.504596

H	5.686713	2.276017	0.593089

H	5.432315	2.395222	2.373373

H	6.216953	0.929945	1.668988

C	-0.211162	-2.293799	3.564655

H	-1.157922	-2.580404	3.082858

H	0.358072	-3.207235	3.829819

H	-0.426974	-1.718407	4.485759

C	1.857362	1.377424	-3.739254

H	1.614579	0.387757	-4.154305

H	1.023085	2.075797	-3.937318

H	2.786042	1.769093	-4.198151

H	-6.333771	-0.031857	-0.108466

H	-2.457939	1.051536	1.530242

H	-2.673965	-1.445215	-1.967162

F	-4.832034	-2.648912	-2.641878

F	-5.516823	-0.686718	-3.337702

F	-6.671794	-1.767842	-1.818212

F	-6.396946	1.770945	1.595174

F	-5.342139	0.507278	3.044370

F	-4.440261	2.393132	2.384946

**TS-2-Coor-E-C**

SCF Done: -2929.15301561 A.U.

Ni	4.024370	13.313567	5.393738

P	1.727843	14.085701	5.493025

O	3.413698	12.327302	6.965188

O	1.923598	15.216374	8.417613

O	2.018349	16.413566	3.843544

O	1.415446	13.391640	2.580938

O	-1.001831	15.010554	6.317671

N	1.172080	13.071288	6.765752

C	2.139245	12.342125	7.284265

C	1.756247	11.396564	8.391876

C	2.712658	10.529691	8.951180

C	2.352345	9.655029	9.989694

C	1.038781	9.638668	10.483900

C	0.084417	10.506360	9.924886

C	0.437003	11.379287	8.885133

C	1.887513	15.833851	6.123943

C	1.964791	16.222260	7.488441

C	2.087662	17.582537	7.849998

H	2.124676	17.876876	8.901596

C	2.164018	18.562164	6.852941

H	2.255258	19.616433	7.138792

C	2.140709	18.214602	5.496657

H	2.222169	18.989091	4.730373

C	2.020846	16.855623	5.147676

C	0.191122	14.173852	4.454412

C	0.195920	13.767035	3.097332

C	-0.987140	13.755881	2.328610

H	-0.978991	13.432816	1.285405

C	-2.188210	14.163395	2.921714

H	-3.110294	14.152088	2.329159

C	-2.234047	14.585870	4.254801

H	-3.183135	14.903582	4.692762

C	-1.045662	14.594849	5.017201

C	5.819147	13.019819	5.936927

O	6.758954	12.592121	5.290885

C	6.027133	13.535404	7.385957

H	6.497839	12.705846	7.945903

H	5.061597	13.766997	7.858308

C	6.956589	14.757564	7.380800

H	7.223227	15.063090	8.411987

H	7.915128	14.508234	6.881147

C	6.338045	15.947143	6.646337

C	7.061279	17.282289	6.747611

H	7.026064	17.650021	7.790888

H	8.129827	17.171683	6.484879

H	6.585210	18.022474	6.087019

O	5.299806	15.826150	5.994830

C	-1.313361	10.550112	10.496949

C	3.375456	8.685338	10.534631

C	-2.223782	15.400509	6.942291

H	-1.952009	15.669224	7.973959

H	-2.678358	16.278550	6.442172

H	-2.955283	14.569420	6.959901

C	2.065291	17.388694	2.802515

H	2.017420	16.819040	1.862313

H	1.203038	18.081214	2.857031

H	3.006681	17.971406	2.832367

C	1.922352	15.570783	9.798464

H	2.856609	16.089636	10.093267

H	1.056512	16.212184	10.056216

H	1.846127	14.618362	10.344422

C	1.459740	12.969979	1.216040

H	1.131629	13.775572	0.530388

H	2.514881	12.728433	1.017020

H	0.836570	12.070608	1.045845

H	0.761414	8.959195	11.294093

H	-0.291781	12.057997	8.432863

H	3.734072	10.553367	8.562542

F	3.071727	8.280247	11.801996

F	4.623797	9.234982	10.569185

F	3.460978	7.559413	9.767084

F	-2.242841	10.858271	9.549413

F	-1.674643	9.359084	11.055793

F	-1.422723	11.496328	11.479366

C	4.317682	12.546522	3.545565

C	4.579733	13.922822	3.497334

H	5.142349	11.829843	3.620804

H	3.328228	12.160088	3.277595

H	3.804695	14.631851	3.189943

H	5.611493	14.287982	3.517393

**2-Coor-E-C**

SCF Done: -2929.1756779 A.U.

Ni	1.215941	-1.665649	-0.624007

P	1.594290	0.537062	0.035222

O	-0.628668	-1.142977	-0.262398

O	2.063590	-1.667192	1.875440

O	2.282567	3.051321	1.626368

O	4.502957	1.044079	0.032418

O	0.538116	2.209915	-2.254848

N	-0.022529	1.097730	0.239932

C	-0.876052	0.104580	0.084464

C	-2.329388	0.415353	0.328589

C	-3.300939	-0.598590	0.251151

C	-4.655222	-0.301121	0.475195

C	-5.056294	1.010156	0.773582

C	-4.085071	2.023207	0.850861

C	-2.730549	1.730653	0.634559

C	2.229061	0.696182	1.760179

C	2.357330	-0.489685	2.526159

C	2.752647	-0.445759	3.879654

H	2.853036	-1.360989	4.466978

C	3.000584	0.798350	4.472904

H	3.302248	0.838265	5.525928

C	2.858728	1.993539	3.755434

H	3.044966	2.950456	4.248943

C	2.464319	1.939392	2.402166

C	2.508043	1.735246	-1.027544

C	3.919440	1.852964	-0.916164

C	4.658191	2.727138	-1.736402

H	5.740774	2.822106	-1.625299

C	3.985456	3.470314	-2.715235

H	4.552600	4.153152	-3.357932

C	2.605764	3.333300	-2.904819

H	2.107029	3.898369	-3.695529

C	1.873290	2.450319	-2.081240

C	0.566728	-3.435558	-0.869748

C	-0.566637	-3.648812	-1.876448

H	-1.459464	-3.187311	-1.415246

H	-0.348568	-3.055154	-2.780180

C	-0.806737	-5.123437	-2.215563

C	0.334921	-5.749324	-3.022364

C	2.443997	-1.988260	-2.252934

H	2.113656	-2.867021	-2.820890

H	2.432666	-1.029042	-2.786746

C	3.184425	-2.133104	-1.073306

H	3.805054	-1.320012	-0.683831

H	3.370915	-3.133137	-0.664098

C	0.273637	-7.258491	-3.226553

H	-0.737629	-7.586727	-3.529355

H	1.014638	-7.569498	-3.978832

H	0.500911	-7.764246	-2.268335

H	-1.737080	-5.235370	-2.809678

H	-0.961286	-5.720768	-1.297097

O	0.998273	-4.338051	-0.162573

O	1.255102	-5.075963	-3.483285

C	-4.510309	3.451354	1.104032

C	-5.675509	-1.415722	0.455566

C	2.133493	-2.895072	2.615097

H	1.424930	-2.886072	3.464976

H	1.850705	-3.674611	1.892328

H	3.159336	-3.075625	2.989722

C	2.426723	4.331320	2.241901

H	3.458646	4.495269	2.610430

H	2.201380	5.063242	1.451927

H	1.714402	4.460708	3.079605

C	5.911530	1.158248	0.243401

H	6.482872	0.876952	-0.662624

H	6.192326	2.184039	0.550307

H	6.144562	0.455924	1.057685

C	-0.157640	2.946363	-3.260392

H	0.236532	2.730565	-4.273114

H	-1.203630	2.611450	-3.195966

H	-0.108775	4.036693	-3.072897

F	-3.567585	4.147016	1.799605

F	-5.675878	3.518975	1.810047

F	-4.716990	4.126754	-0.066676

F	-5.748828	-2.051549	1.662135

F	-5.366763	-2.367380	-0.472657

F	-6.928231	-0.957396	0.171136

H	-1.960930	2.504869	0.701696

H	-2.983180	-1.617002	0.014842

H	-6.111064	1.240523	0.945158

**TS-2-Ins-E-C**

SCF Done: -2929.15787523 A.U.

Ni	1.045869	-1.664290	-0.144419

P	1.748130	0.401579	-0.127178

O	-0.739111	-0.834065	-0.079914

O	2.144879	-1.041118	2.433760

O	2.796902	3.160660	0.374000

O	4.721492	0.373771	-0.226927

O	0.921778	1.115121	-2.930264

N	0.286774	1.292301	-0.202132

C	-0.753233	0.474730	-0.133408

C	-2.115591	1.113018	-0.112851

C	-2.248459	2.510467	-0.231000

C	-3.520366	3.101750	-0.221980

C	-4.674871	2.311344	-0.088733

C	-4.541395	0.919632	0.034415

C	-3.270720	0.321728	0.020571

C	2.507198	1.063779	1.415783

C	2.573293	0.251177	2.576806

C	3.048507	0.771698	3.800046

H	3.101517	0.142739	4.691564

C	3.445042	2.112761	3.862793

H	3.809493	2.519898	4.813049

C	3.378153	2.948898	2.740277

H	3.684770	3.994587	2.819171

C	2.905404	2.422373	1.520625

C	2.827665	0.881348	-1.544090

C	4.242877	0.819691	-1.433517

C	5.082953	1.178000	-2.507123

H	6.169920	1.145024	-2.403733

C	4.505502	1.566887	-3.722283

H	5.153019	1.848212	-4.560573

C	3.116514	1.575694	-3.893407

H	2.689134	1.848964	-4.860765

C	2.281183	1.208163	-2.816007

C	0.162803	-3.459662	0.195929

C	-1.102557	-3.818752	-0.578518

H	-1.658875	-2.869023	-0.696422

H	-0.834667	-4.169304	-1.589207

C	-1.949300	-4.864578	0.153723

C	-1.260545	-6.230437	0.240015

C	1.660495	-3.517827	-0.862072

H	1.803296	-4.539713	-0.474009

H	1.335999	-3.546019	-1.912161

C	2.707994	-2.532824	-0.540392

H	3.231097	-2.056664	-1.378955

H	3.330337	-2.706309	0.347229

C	-1.962463	-7.296043	1.074191

H	-3.038843	-7.358659	0.829851

H	-1.481751	-8.274278	0.920141

H	-1.893280	-7.025933	2.145552

H	-2.924125	-5.000783	-0.356963

H	-2.189004	-4.525217	1.179286

O	0.297892	-3.673118	1.401078

O	-0.193399	-6.454857	-0.328533

C	-5.766179	0.060320	0.246713

C	-3.653675	4.593632	-0.424099

C	2.050472	-1.868706	3.599029

H	1.600568	-2.804139	3.237437

H	3.049284	-2.065088	4.035807

H	1.398405	-1.406839	4.364907

C	3.087497	4.556670	0.441798

H	4.147224	4.742090	0.706522

H	2.890725	4.946284	-0.568146

H	2.432378	5.071455	1.170812

C	6.135427	0.340200	-0.029906

H	6.279142	-0.012026	1.002469

H	6.627427	-0.361485	-0.731583

H	6.585352	1.345638	-0.141662

C	0.318169	1.492610	-4.167860

H	0.535355	2.548646	-4.420257

H	0.649193	0.840690	-5.000156

H	-0.764157	1.369036	-4.014225

F	-5.996610	-0.168318	1.573389

F	-5.634857	-1.162033	-0.346117

F	-6.892368	0.641248	-0.257879

F	-3.700173	4.916439	-1.751957

F	-2.602408	5.275595	0.111072

F	-4.793855	5.084992	0.141687

H	-3.159873	-0.761468	0.113682

H	-1.339952	3.112209	-0.324044

H	-5.664828	2.774982	-0.079774

**2-cycle5-T**

SCF Done: -2929.19503731 A.U.

Ni	1.292080	-1.353493	-0.641042

P	1.523897	0.677036	-0.036055

O	-0.618820	-0.973141	-0.501537

O	2.065164	-1.322989	2.154440

O	1.953178	3.323493	1.245111

O	4.436767	1.480552	0.241946

O	0.746527	1.691853	-2.697194

N	-0.082848	1.276090	-0.018817

C	-0.922050	0.270797	-0.245361

C	-2.391717	0.588344	-0.205142

C	-3.335500	-0.427106	-0.445196

C	-4.709407	-0.137943	-0.412452

C	-5.157245	1.163942	-0.137948

C	-4.213545	2.177311	0.103340

C	-2.839562	1.895169	0.069418

C	2.050771	1.011866	1.703133

C	2.204411	-0.045670	2.636924

C	2.473885	0.223652	3.997677

H	2.599824	-0.591468	4.713568

C	2.571597	1.550824	4.429826

H	2.776014	1.757390	5.486797

C	2.403664	2.620407	3.540831

H	2.473628	3.649004	3.902605

C	2.139764	2.346043	2.183970

C	2.551635	1.708146	-1.170823

C	3.914509	2.011648	-0.911647

C	4.675679	2.795844	-1.802409

H	5.717958	3.039332	-1.583934

C	4.084485	3.252967	-2.986895

H	4.672437	3.864457	-3.680996

C	2.764989	2.919208	-3.312168

H	2.333583	3.257180	-4.257078

C	2.012237	2.126940	-2.418824

C	3.155338	-1.698638	-0.999410

H	3.892135	-1.132741	-0.407502

H	3.264545	-1.406894	-2.064146

C	3.338563	-3.219320	-0.821988

H	3.559035	-3.461909	0.240005

H	4.172848	-3.672410	-1.403335

C	2.042586	-3.919376	-1.112178

C	1.966341	-5.406119	-1.405972

H	2.822068	-5.912005	-0.925462

O	0.988057	-3.236809	-1.102464

C	1.894557	4.679721	1.684618

H	1.089977	4.828873	2.430311

H	2.859310	5.013911	2.115379

H	1.674420	5.270548	0.782927

C	2.016062	-2.397008	3.100816

H	1.784443	-3.300455	2.517391

H	2.988143	-2.528344	3.616068

H	1.223270	-2.229617	3.855077

C	5.786508	1.795858	0.581365

H	6.497820	1.411628	-0.175953

H	5.933078	2.887164	0.699227

H	5.970361	1.296413	1.544515

C	0.133898	2.117822	-3.913862

H	0.041711	3.220482	-3.957564

H	0.694404	1.760460	-4.800145

H	-0.869341	1.666654	-3.908852

C	-4.684741	3.566569	0.464931

C	-5.706122	-1.224869	-0.739259

H	-6.227113	1.387000	-0.112546

H	-2.092204	2.673048	0.249895

H	-2.972852	-1.437561	-0.652434

F	-5.875331	-1.364040	-2.088688

F	-5.304105	-2.440497	-0.267495

F	-6.937776	-0.968198	-0.210872

F	-3.819067	4.526697	0.031577

F	-5.908443	3.846129	-0.072739

F	-4.803408	3.722569	1.817361

C	0.637238	-6.024406	-0.955414

H	0.575817	-7.090266	-1.251474

H	-0.215420	-5.518663	-1.447070

C	0.448484	-5.919057	0.561655

C	-0.872971	-6.413520	1.128706

H	-1.164495	-7.388520	0.697889

H	-0.812980	-6.484410	2.225480

H	-1.669919	-5.692546	0.861915

H	2.092544	-5.538418	-2.500494

O	1.324578	-5.432435	1.276166

**TS-Isom-CO-T**

SCF Done: -2850.59018023 A.U.

Ni	-2.339591	3.462332	4.966434

P	-2.565086	1.229572	4.906958

O	-0.353554	2.817100	5.525448

O	-2.864249	3.069463	7.163520

O	-3.608632	-1.451153	5.944489

O	-5.377636	1.486990	4.587944

O	-1.683492	-0.732611	2.635229

N	-0.987531	0.611985	4.959497

C	-0.116790	1.564219	5.319756

C	1.315084	1.108261	5.470020

C	2.305889	2.028568	5.858437

C	3.641790	1.614572	5.988788

C	4.003005	0.281969	5.733277

C	3.011867	-0.636150	5.347021

C	1.675336	-0.229055	5.218329

C	-3.276699	0.805639	6.561061

C	-3.283668	1.805905	7.557108

C	-3.697880	1.537410	8.875871

H	-3.709224	2.321297	9.636274

C	-4.111556	0.238788	9.198483

H	-4.440708	0.019472	10.220366

C	-4.107422	-0.786443	8.243905

H	-4.422495	-1.793472	8.527171

C	-3.680284	-0.506080	6.929099

C	-3.552134	0.338220	3.643229

C	-4.947972	0.616458	3.619137

C	-5.806895	0.036982	2.665452

H	-6.877446	0.253558	2.666639

C	-5.263058	-0.828528	1.708691

H	-5.921087	-1.287009	0.961739

C	-3.892340	-1.109236	1.679328

H	-3.493940	-1.777123	0.912445

C	-3.031802	-0.521818	2.634137

C	-2.784598	3.565436	3.023322

H	-3.166433	4.572457	2.796672

C	-2.567817	5.222713	5.074458

O	-2.855682	6.345913	5.192894

H	0.890726	-0.929304	4.917375

H	5.043574	-0.037341	5.833983

H	2.010451	3.062664	6.058245

C	3.370102	-2.086313	5.119195

C	4.706094	2.628299	6.342832

C	-6.754105	1.873368	4.593258

H	-7.040527	2.359367	3.641010

H	-7.416442	1.006005	4.779457

C	-1.132984	-1.613625	1.654893

H	-1.551409	-2.635180	1.743363

H	-1.299014	-1.238729	0.625795

C	-2.446809	3.980235	8.201191

H	-1.939257	4.806164	7.685873

H	-3.319773	4.367405	8.757878

C	-3.939940	-2.799873	6.279744

H	-3.277537	-3.193927	7.074434

H	-4.995808	-2.892821	6.600783

F	2.642990	-2.630227	4.098904

F	3.123535	-2.847223	6.226013

F	4.688995	-2.246887	4.811384

F	5.148266	3.296039	5.236026

F	4.239526	3.568387	7.211978

F	5.797362	2.043381	6.915926

C	-1.478467	3.303922	2.286217

H	-1.682050	3.236174	1.189122

H	-1.026356	2.328263	2.549807

C	-0.432163	4.418076	2.427709

C	1.003319	4.039106	2.090602

H	1.057928	3.393248	1.195039

H	1.616756	4.942488	1.949725

H	1.414254	3.455463	2.936878

H	-3.557944	2.818881	2.792854

O	-0.737152	5.554914	2.780546

H	-3.788035	-3.376151	5.355086

H	-1.743899	3.482232	8.891675

H	-6.852500	2.594428	5.418030

H	-0.053562	-1.642847	1.865619

**1-Coor-CO-C**

SCF Done: -2850.60662985 A.U.

Ni	1.491207	-1.795927	-0.683047

P	1.475429	0.395297	-0.085976

O	-0.402416	-1.556575	-0.728934

O	4.272945	0.662628	0.429895

O	0.126920	0.720298	2.694881

O	1.664587	3.364826	0.333984

O	2.404956	-0.039289	-2.862142

N	-0.212127	0.705779	-0.059400

C	-0.885178	-0.368665	-0.421135

C	-2.381418	-0.250358	-0.502814

C	-3.017157	0.953283	-0.141261

C	-4.414170	1.058628	-0.206815

C	-5.192714	-0.029073	-0.638383

C	-4.557130	-1.226027	-1.003268

C	-3.159028	-1.340030	-0.934706

C	2.213512	0.764735	1.561263

C	3.630520	0.825553	1.632485

C	4.305921	1.033179	2.849989

H	5.396275	1.086449	2.887177

C	3.552430	1.163957	4.023070

H	4.065945	1.325302	4.977707

C	2.155703	1.076327	4.001713

H	1.594054	1.162026	4.934592

C	1.483836	0.861368	2.777741

C	2.088909	1.676156	-1.259682

C	2.096658	3.056424	-0.925490

C	2.521478	4.026218	-1.857850

H	2.531270	5.087407	-1.598568

C	2.922549	3.614802	-3.134825

H	3.249212	4.366861	-3.862158

C	2.903838	2.265207	-3.508134

H	3.207779	1.974268	-4.516045

C	2.478970	1.300445	-2.570784

H	-2.656093	-2.269683	-1.212800

H	-6.281334	0.056492	-0.690086

H	-2.395405	1.792387	0.183757

C	-5.373988	-2.382491	-1.533172

C	-5.090730	2.329216	0.254717

C	1.625930	4.739929	0.716879

H	2.633809	5.198462	0.693577

H	0.942024	5.321863	0.069183

H	1.246381	4.744930	1.749414

C	2.724685	-0.461992	-4.190382

H	2.057233	0.011093	-4.935969

H	3.778987	-0.239221	-4.444981

H	2.569108	-1.550965	-4.194407

C	5.701580	0.605686	0.428630

H	6.142948	1.562934	0.767270

H	6.071278	-0.220475	1.064670

H	5.987505	0.419850	-0.616871

C	-0.634673	0.796772	3.899628

H	-0.346211	0.003973	4.617441

H	-0.531529	1.786246	4.386970

H	-1.680915	0.649892	3.592236

F	-4.839454	-3.584498	-1.173480

F	-5.435683	-2.369010	-2.897178

F	-6.658760	-2.351039	-1.076264

F	-5.313848	2.314059	1.603622

F	-4.334534	3.431547	-0.007913

F	-6.302721	2.508190	-0.344728

C	1.193841	-3.699654	-1.190735

H	2.094070	-4.264265	-1.483038

C	0.438372	-4.384951	-0.059216

H	0.547062	-3.540081	-2.071360

H	0.097785	-5.403368	-0.355892

H	-0.493607	-3.824721	0.163868

C	1.197796	-4.518839	1.261384

C	0.586171	-5.465628	2.292919

O	2.237052	-3.906022	1.502668

H	0.726930	-6.513399	1.964065

H	1.076346	-5.329402	3.269250

H	-0.503577	-5.305235	2.388417

C	3.175408	-2.157137	-0.630406

O	4.312844	-2.412873	-0.633351

**TS-1-Ins-CO-C**

SCF Done: -2850.59108995 A.U.

Ni	-1.281668	-1.574895	0.456310

P	-1.432082	0.565874	0.092076

O	0.627132	-1.270629	0.411622

O	-4.275136	0.527564	-0.056079

O	-0.498426	1.527993	-2.714384

O	-2.123566	3.499088	0.180319

O	-1.725689	-0.367673	2.876609

N	0.213690	1.007578	-0.074852

C	0.996662	-0.037293	0.152082

C	2.480262	0.201265	0.108330

C	3.373367	-0.860002	0.343201

C	4.759429	-0.639231	0.295497

C	5.267551	0.637659	0.009931

C	4.373857	1.696524	-0.225371

C	2.988381	1.483762	-0.174785

C	-2.408420	1.062851	-1.381830

C	-3.819201	0.917318	-1.287888

C	-4.664171	1.161715	-2.387403

H	-5.747004	1.047575	-2.299971

C	-4.090884	1.557536	-3.602194

H	-4.738450	1.752764	-4.464615

C	-2.705064	1.700354	-3.739589

H	-2.284558	1.999934	-4.702127

C	-1.859201	1.444032	-2.637207

C	-1.993643	1.567323	1.530137

C	-2.274364	2.955355	1.425387

C	-2.670421	3.702431	2.554315

H	-2.891618	4.769039	2.471762

C	-2.764695	3.063097	3.796481

H	-3.068208	3.642410	4.675911

C	-2.465105	1.702996	3.943847

H	-2.529365	1.234889	4.928472

C	-2.070020	0.961892	2.811103

C	-1.456312	-3.705545	0.655470

H	-0.469413	-3.480107	1.107116

C	-2.761331	-2.426902	0.320111

O	-3.876884	-2.784757	0.108761

C	-1.293438	-4.536521	-0.614826

H	-0.592255	-4.055984	-1.324166

H	-2.258700	-4.616371	-1.158535

C	-0.822090	-5.973586	-0.333685

C	-0.390183	-6.785171	-1.551615

H	-1.115570	-6.686980	-2.380172

H	0.577823	-6.400720	-1.927030

H	-0.270778	-7.844244	-1.276033

H	-2.035643	-4.238260	1.425048

O	-0.796224	-6.439883	0.801207

H	2.278772	2.298004	-0.347754

H	6.346902	0.806642	-0.028736

H	2.967410	-1.852122	0.557516

C	4.906851	3.059786	-0.602319

C	5.706761	-1.773306	0.615063

C	-2.376698	4.895269	0.017489

H	-3.424820	5.151351	0.266952

H	-1.691248	5.506045	0.636295

H	-2.193419	5.102895	-1.047159

C	-1.730609	-1.007129	4.157254

H	-1.024809	-0.516206	4.853805

H	-2.744402	-1.016835	4.601337

H	-1.402899	-2.040095	3.968524

C	-5.657563	0.186568	0.078634

H	-6.308241	1.066502	-0.089926

H	-5.937066	-0.627257	-0.615505

H	-5.773481	-0.161230	1.115345

C	0.087302	1.884812	-3.966369

H	-0.224693	2.897143	-4.290336

H	1.173492	1.876556	-3.791589

H	-0.164258	1.153583	-4.759509

F	4.979818	3.209656	-1.959566

F	6.162448	3.270322	-0.112974

F	4.110291	4.062687	-0.137367

F	6.935090	-1.585589	0.052095

F	5.900712	-1.899483	1.960936

F	5.229454	-2.970330	0.170176

**1-cycle6-T**

SCF Done: -2850.61336581 A.U.

Ni	-1.300045	1.711805	-0.406298

P	-1.333527	-0.369039	0.206665

O	0.656581	1.450378	-0.435764

O	-1.478953	1.545509	2.379091

O	-2.166629	-3.036881	1.468769

O	-4.194714	-0.782933	0.029671

O	-0.182695	-2.087648	-2.092672

N	0.323954	-0.770964	0.286819

C	1.069532	0.275368	-0.051172

C	2.562068	0.091592	0.013843

C	3.118846	-1.142690	0.400193

C	4.512179	-1.302492	0.449520

C	5.365179	-0.237461	0.113296

C	4.808234	0.992445	-0.271760

C	3.414800	1.159394	-0.320941

C	-1.945261	-0.723509	1.903773

C	-1.996496	0.357983	2.822023

C	-2.510304	0.183089	4.124218

H	-2.565196	1.021953	4.821673

C	-2.918237	-1.093295	4.527559

H	-3.307078	-1.236469	5.542408

C	-2.816820	-2.198263	3.671161

H	-3.115048	-3.188809	4.022635

C	-2.320617	-2.012291	2.364512

C	-2.196522	-1.499287	-0.977412

C	-3.616030	-1.567790	-0.932643

C	-4.355407	-2.373647	-1.819865

H	-5.444700	-2.428050	-1.753500

C	-3.667304	-3.115938	-2.788657

H	-4.229914	-3.750877	-3.482738

C	-2.273649	-3.049335	-2.893746

H	-1.761281	-3.621859	-3.670226

C	-1.537951	-2.233456	-2.003282

C	-3.130436	2.032160	-0.396004

O	-3.845043	2.229971	0.580330

C	-3.708076	2.269084	-1.821662

H	-4.506846	3.031750	-1.752078

H	-4.179694	1.318335	-2.131867

C	-2.652091	2.667583	-2.883577

H	-2.039578	1.777445	-3.133323

H	-3.139053	3.016950	-3.811084

C	-1.681945	3.704262	-2.345189

C	-1.455625	4.994214	-3.089749

H	-2.408764	5.550883	-3.176627

H	-0.705063	5.615895	-2.579042

H	-1.124100	4.778697	-4.123709

O	-1.068985	3.480251	-1.279818

C	5.711364	2.125098	-0.701242

C	5.098708	-2.610953	0.926489

C	0.520617	-2.851918	-3.070983

H	1.581354	-2.595665	-2.931132

H	0.381820	-3.939799	-2.916317

H	0.210506	-2.586673	-4.101328

C	-2.510070	-4.356425	1.889463

H	-2.300401	-5.003296	1.024407

H	-1.894573	-4.680866	2.751016

H	-3.582374	-4.435839	2.156258

C	-1.748280	2.728040	3.138750

H	-2.835976	2.872383	3.268450

H	-1.244160	2.697583	4.123963

H	-1.334180	3.555345	2.543710

C	-5.618268	-0.741897	0.126724

H	-6.082058	-0.428128	-0.829055

H	-6.031599	-1.724316	0.426909

H	-5.831933	0.008808	0.900928

H	6.450208	-0.365210	0.151264

H	2.967810	2.113599	-0.612903

H	2.440385	-1.962108	0.654822

F	6.353277	-2.818865	0.428971

F	4.331118	-3.675728	0.556254

F	5.201354	-2.651440	2.287684

F	5.896190	2.133078	-2.056192

F	5.194432	3.341444	-0.367801

F	6.949527	2.035645	-0.134620

**TS-2-Coor-E-T**

SCF Done: -2929.16287171 A.U.

Ni	1.528768	-1.410666	-0.778900

P	1.376452	0.546065	0.189554

O	-0.458398	-1.388443	-0.606162

O	1.671925	-1.618287	2.159125

O	1.958057	3.094755	1.783686

O	4.167142	1.376660	0.267922

O	0.081460	2.378057	-1.900337

N	-0.310556	0.765296	0.345852

C	-0.963937	-0.311081	-0.083087

C	-2.464310	-0.283390	0.052736

C	-3.224257	-1.391915	-0.362681

C	-4.623354	-1.375311	-0.240712

C	-5.278545	-0.253632	0.291488

C	-4.518178	0.854120	0.704002

C	-3.119713	0.841611	0.589152

C	1.906347	0.735177	1.956561

C	1.951825	-0.419367	2.778750

C	2.251702	-0.337425	4.153897

H	2.297807	-1.237535	4.771542

C	2.486505	0.918626	4.724908

H	2.720343	0.990053	5.793295

C	2.410611	2.086918	3.957754

H	2.573008	3.058685	4.429749

C	2.105278	1.995673	2.583099

C	2.130369	1.907587	-0.793143

C	3.531089	2.120139	-0.693794

C	4.199774	3.049350	-1.513525

H	5.278127	3.200020	-1.426070

C	3.460636	3.783051	-2.448764

H	3.971971	4.509846	-3.090372

C	2.080561	3.594815	-2.588616

H	1.527895	4.167864	-3.336339

C	1.418118	2.648234	-1.776689

C	3.370604	-1.449129	-0.968141

O	4.062047	-0.806608	-1.752052

C	3.992730	-2.624606	-0.170464

H	3.538141	-2.666734	0.833867

H	5.081189	-2.456739	-0.074065

C	3.750529	-3.947304	-0.924171

H	4.266165	-3.918428	-1.908393

H	4.194502	-4.806170	-0.384229

C	2.286722	-4.248245	-1.205397

C	1.904401	-5.673010	-1.540240

H	2.564173	-6.077540	-2.330823

H	2.042711	-6.319974	-0.652086

H	0.854385	-5.722790	-1.865429

O	1.404415	-3.368770	-1.177125

C	-5.216888	2.093415	1.212956

C	-5.419218	-2.600170	-0.626742

C	-0.669638	3.113334	-2.866616

H	-1.701707	2.743056	-2.775259

H	-0.650096	4.200536	-2.655994

H	-0.301900	2.932166	-3.895663

C	2.097120	4.386753	2.373416

H	1.920748	5.100704	1.555076

H	1.350121	4.550108	3.174418

H	3.114371	4.542329	2.783439

C	1.340031	-2.736688	2.986974

H	0.553971	-2.473083	3.719230

H	0.958010	-3.505566	2.298146

H	2.225388	-3.129282	3.525633

C	5.595450	1.352814	0.258205

H	5.977958	1.003491	-0.718405

H	6.021925	2.346700	0.497871

H	5.882670	0.640433	1.046735

H	-6.367721	-0.242023	0.384341

H	-2.512387	1.691742	0.913033

H	-2.700886	-2.255464	-0.782551

F	-6.714276	-2.295452	-0.930334

F	-5.455245	-3.516214	0.387553

F	-4.882838	-3.233308	-1.708774

F	-4.472355	2.751649	2.145185

F	-5.461423	2.982110	0.202704

F	-6.423235	1.802311	1.781736

C	1.051250	-1.760034	-4.133448

C	1.185850	-0.612033	-3.440808

H	1.918232	-2.274360	-4.567393

H	0.074445	-2.236181	-4.280236

H	0.316154	-0.088291	-3.029540

H	2.162488	-0.136075	-3.298868

**2-Coor-E-T**

SCF Done: -2929.1756779 A.U.

Ni	-0.383582	1.563389	-1.361638

P	-1.164593	0.087918	0.130789

O	1.326672	0.563681	-1.030674

O	-4.137427	0.073486	1.077304

O	0.112601	1.965762	1.838144

O	-2.195465	-2.096120	1.862293

O	-1.986282	-0.691966	-2.651795

N	0.270087	-0.670964	0.682713

C	1.309362	-0.303287	-0.056671

C	2.626039	-0.962539	0.262602

C	2.722673	-1.905753	1.303957

C	3.951057	-2.521079	1.586540

C	5.096773	-2.205898	0.835645

C	4.999171	-1.266683	-0.202623

C	3.771913	-0.646474	-0.489086

C	-1.972601	0.907987	1.568650

C	-3.346047	0.824796	1.914053

C	-3.860538	1.516158	3.029940

H	-4.914509	1.430111	3.304172

C	-3.010706	2.340530	3.779186

H	-3.410996	2.878301	4.646129

C	-1.668944	2.514418	3.421255

H	-1.030691	3.189783	3.995353

C	-1.163797	1.816616	2.305822

C	-2.146144	-1.386338	-0.386457

C	-2.490976	-2.388750	0.561933

C	-3.099107	-3.594988	0.160242

H	-3.367794	-4.358811	0.893796

C	-3.352529	-3.810225	-1.200517

H	-3.823559	-4.747683	-1.517728

C	-3.005135	-2.858086	-2.165355

H	-3.200333	-3.058381	-3.220985

C	-2.391501	-1.653472	-1.757231

C	-1.905892	2.637640	-1.287645

C	-3.259146	2.218384	-1.878804

H	-3.084229	1.609666	-2.779153

H	-3.723961	1.545227	-1.131072

C	-4.174686	3.413509	-2.168063

C	-3.718339	4.254866	-3.363386

C	0.791585	2.924727	-2.504797

H	0.456831	3.930297	-2.227497

H	1.799712	2.625292	-2.201066

C	0.042063	2.138077	-3.364325

H	0.459220	1.201950	-3.756618

H	-0.874941	2.521555	-3.827939

C	-4.405303	5.603361	-3.543058

H	-4.032847	6.301998	-2.769104

H	-4.176360	6.017837	-4.536844

H	-5.499225	5.525618	-3.403883

H	-4.252995	4.068569	-1.280253

H	-5.206582	3.067538	-2.386843

O	-1.758182	3.719251	-0.726247

O	-2.845874	3.871048	-4.140962

C	6.230972	-0.876288	-0.986578

C	4.056106	-3.489702	2.741799

C	-5.502200	-0.130090	1.443966

H	-5.914299	-0.803105	0.676878

H	-5.588309	-0.608225	2.438776

H	-6.072083	0.819861	1.446665

C	0.953824	2.942465	2.457075

H	1.164649	2.681408	3.512002

H	1.890767	2.925548	1.881605

H	0.502387	3.951307	2.404272

C	-2.370958	-3.118914	2.843184

H	-1.782688	-4.022547	2.593620

H	-2.000809	-2.687235	3.784941

H	-3.437252	-3.396410	2.960541

C	-2.102136	-0.984067	-4.047411

H	-3.159308	-1.109461	-4.352492

H	-1.675502	-0.112222	-4.564960

H	-1.531183	-1.892720	-4.317884

F	7.185384	-1.850568	-0.959309

F	6.805379	0.257262	-0.483798

F	5.939723	-0.623187	-2.294232

F	5.058002	-4.397554	2.556591

F	2.899799	-4.187773	2.927829

F	4.320674	-2.842950	3.916332

H	3.681950	0.078146	-1.302778

H	1.819459	-2.147850	1.871040

H	6.049439	-2.698533	1.047187

**TS-Isom-2**

SCF Done: -2929.1496249 A.U.

Ni	1.817286	2.870532	-2.640597

P	1.734580	0.621312	-2.021969

O	3.741695	2.496713	-2.707578

O	-1.079722	0.395061	-1.513775

O	3.073102	-0.030643	0.708497

O	0.627416	-2.322593	-1.904295

O	1.751769	1.266594	-4.774626

N	3.413096	0.240810	-2.031733

C	4.136746	1.281336	-2.418074

C	5.622034	1.059117	-2.545874

C	6.170998	-0.218512	-2.325676

C	7.553944	-0.420867	-2.446482

C	8.404029	0.644874	-2.788256

C	7.854549	1.917743	-3.007552

C	6.470856	2.128175	-2.887071

C	0.984574	0.122071	-0.413082

C	-0.435079	0.117413	-0.331631

C	-1.114228	-0.152644	0.871134

H	-2.205901	-0.166134	0.911349

C	-0.364421	-0.407682	2.027397

H	-0.881605	-0.621364	2.969757

C	1.034178	-0.381192	2.000667

H	1.597137	-0.570947	2.917396

C	1.710477	-0.106445	0.789413

C	1.113500	-0.510298	-3.345871

C	0.643695	-1.839205	-3.184443

C	0.237925	-2.604340	-4.299855

H	-0.131935	-3.624620	-4.173024

C	0.327125	-2.053144	-5.583970

H	0.015203	-2.650951	-6.447889

C	0.820348	-0.757654	-5.787332

H	0.897850	-0.351534	-6.798553

C	1.218677	-0.003921	-4.666933

C	2.270673	4.207555	-1.385940

C	1.340108	4.124200	-0.162492

H	0.296732	4.161699	-0.514781

H	1.485014	3.132690	0.303996

C	1.617819	5.259318	0.832902

C	1.208781	6.636781	0.294898

C	0.723891	4.381851	-3.491266

H	0.688295	5.325736	-2.935655

H	1.124826	4.452988	-4.511078

C	-0.092714	3.290779	-3.105226

H	-0.366856	2.511354	-3.825100

H	-0.752549	3.364006	-2.232914

C	1.726313	7.843909	1.065155

H	2.804005	7.971999	0.845808

H	1.189511	8.752701	0.752743

H	1.635735	7.701860	2.157723

H	2.683308	5.281831	1.126770

H	1.044191	5.084243	1.766728

O	3.224154	4.942675	-1.465138

O	0.499380	6.757786	-0.701293

C	8.761234	3.085470	-3.322725

C	8.138083	-1.782889	-2.151272

C	-2.507645	0.386904	-1.520872

H	-2.799706	0.622783	-2.555085

H	-2.908352	-0.607194	-1.243020

H	-2.923500	1.152150	-0.836511

C	3.838471	-0.304987	1.880928

H	3.658119	-1.331670	2.255528

H	4.889541	-0.208821	1.570281

H	3.626641	0.421080	2.691105

C	0.231411	-3.678807	-1.700887

H	0.892300	-4.381076	-2.245159

H	0.324460	-3.850662	-0.618086

H	-0.818287	-3.851064	-2.010687

C	2.127767	1.738815	-6.074291

H	1.241590	1.923925	-6.711610

H	2.663250	2.682822	-5.896506

H	2.800880	1.019010	-6.575680

F	9.935740	2.681700	-3.888963

F	9.084720	3.787017	-2.195883

F	8.173751	3.965921	-4.181173

F	9.325395	-1.980483	-2.794166

F	7.297630	-2.789056	-2.523124

F	8.380371	-1.944699	-0.814914

H	6.031602	3.114878	-3.057729

H	5.492464	-1.037432	-2.069725

H	9.479355	0.481175	-2.896996

**TS-2-Ins-E-T**

SCF Done: -2929.14896669 A.U.

Ni	1.377865	-1.772358	0.325564

P	1.456802	0.525537	0.416287

O	-0.488600	-1.482250	0.378857

O	2.058598	-1.084860	2.775590

O	1.659880	3.410047	1.362574

O	4.275051	1.227421	0.443121

O	0.393343	1.713199	-2.206720

N	-0.229436	0.881584	0.373095

C	-0.928752	-0.233772	0.398319

C	-2.426547	-0.111072	0.454355

C	-3.238755	-1.258027	0.489207

C	-4.638362	-1.132032	0.527300

C	-5.240072	0.134046	0.532345

C	-4.425937	1.280829	0.499379

C	-3.029896	1.162913	0.461127

C	1.915295	1.176646	2.079556

C	2.155822	0.245038	3.119717

C	2.460255	0.673834	4.428285

H	2.646874	-0.045226	5.228916

C	2.501812	2.047607	4.698399

H	2.731766	2.386117	5.715192

C	2.242087	2.998065	3.702652

H	2.267360	4.062839	3.946329

C	1.941172	2.559415	2.396032

C	2.327916	1.553220	-0.841335

C	3.725859	1.766360	-0.689866

C	4.473590	2.480923	-1.647000

H	5.544165	2.649704	-1.509964

C	3.823076	2.977697	-2.783485

H	4.396291	3.537940	-3.530939

C	2.456662	2.754600	-2.990610

H	1.976053	3.132778	-3.895808

C	1.715014	2.026541	-2.033836

C	2.736413	-2.722787	-0.976627

C	2.897835	-2.109815	-2.356826

H	3.364281	-1.120133	-2.225162

H	3.591673	-2.759750	-2.931537

C	1.593545	-1.990656	-3.174040

C	1.270108	-3.202530	-4.054851

C	2.117886	-4.106848	-0.749073

H	1.703003	-4.530813	-1.677758

H	2.891153	-4.802069	-0.369906

C	1.048922	-3.720317	0.274743

H	1.221552	-4.058527	1.309845

H	0.005758	-3.844952	-0.043763

C	-0.121121	-3.222838	-4.678571

H	-0.870397	-3.446575	-3.894641

H	-0.390411	-2.237708	-5.101614

H	-0.176447	-3.998720	-5.457448

H	1.671766	-1.122830	-3.861192

H	0.729882	-1.757042	-2.519678

O	3.265680	-2.159794	0.060225

O	2.082640	-4.103190	-4.254689

C	-5.061649	2.650771	0.563349

C	-5.477527	-2.388722	0.563609

C	2.364333	-2.065968	3.770859

H	2.274509	-3.035566	3.260080

H	3.396318	-1.943294	4.150534

H	1.648793	-2.021187	4.614146

C	1.630932	4.811372	1.634123

H	2.617828	5.182891	1.973422

H	1.370212	5.289612	0.678172

H	0.864517	5.061712	2.393061

C	5.687237	1.328527	0.627733

H	6.010625	2.383655	0.721736

H	5.898603	0.795806	1.566548

H	6.238623	0.846532	-0.202418

C	-0.293393	2.276146	-3.323647

H	0.113440	1.908965	-4.287250

H	-1.338476	1.947876	-3.219800

H	-0.252723	3.382592	-3.310765

F	-6.313915	2.655370	0.020401

F	-4.323640	3.584781	-0.101757

F	-5.186722	3.087365	1.851108

F	-5.201743	-3.201672	-0.498738

F	-6.813541	-2.122240	0.535985

F	-5.231073	-3.120285	1.688938

H	-2.382180	2.043636	0.430325

H	-2.762709	-2.242356	0.483409

H	-6.328617	0.228049	0.553072

2-cycle5-C

**SCF Done: -2929.18038951 A.U.**

Ni	0.855196	-1.900284	-0.432609

P	1.085993	0.328516	0.007207

O	-0.963387	-1.560897	-0.155856

O	2.504535	-1.243857	2.080326

O	0.997317	3.128043	1.106970

O	3.739784	1.945808	-0.074013

O	0.055082	0.551847	-2.680806

N	-0.582284	0.767299	0.125850

C	-1.336659	-0.310922	0.042447

C	-2.822611	-0.127999	0.185331

C	-3.689691	-1.233620	0.111338

C	-5.076741	-1.052541	0.239709

C	-5.612827	0.228071	0.445741

C	-4.744893	1.331052	0.521963

C	-3.359285	1.157747	0.391744

C	1.721996	0.944621	1.625631

C	2.296019	0.030441	2.545177

C	2.626456	0.431713	3.858597

H	3.061676	-0.275227	4.568428

C	2.384774	1.754339	4.248532

H	2.634366	2.065830	5.269540

C	1.832537	2.689460	3.362385

H	1.661092	3.717657	3.690408

C	1.509647	2.282677	2.051485

C	1.816557	1.365138	-1.338929

C	3.038464	2.081515	-1.249445

C	3.512466	2.862940	-2.324005

H	4.444709	3.426149	-2.236247

C	2.786504	2.898178	-3.520902

H	3.152677	3.506558	-4.355785

C	1.615500	2.146618	-3.677274

H	1.077873	2.162669	-4.628186

C	1.152013	1.367001	-2.597786

C	0.456054	-3.780277	-0.759861

H	-0.521571	-3.896231	-1.251969

H	0.410298	-4.212724	0.258403

C	1.629517	-4.355525	-1.574274

H	1.414879	-4.289237	-2.663869

H	1.866019	-5.426450	-1.390145

C	2.835009	-3.494908	-1.334179

C	4.246990	-3.922367	-1.656574

H	4.268245	-4.339756	-2.682428

O	2.634755	-2.344199	-0.849804

C	0.596031	4.434019	1.519735

H	-0.150916	4.388830	2.335410

H	1.459825	5.045312	1.849539

H	0.141689	4.896475	0.630691

C	2.966246	-2.231864	3.007389

H	3.973614	-1.986941	3.393483

H	2.260610	-2.349676	3.852357

H	3.027695	-3.165809	2.432189

C	4.825364	2.841582	0.168562

H	5.134488	2.653082	1.207884

H	5.679816	2.647428	-0.509815

H	4.508840	3.896720	0.062097

C	-0.694268	0.540797	-3.895964

H	-0.080714	0.192258	-4.750133

H	-1.518893	-0.166588	-3.724119

H	-1.107586	1.541931	-4.125281

C	-5.304146	2.705022	0.809752

C	-5.999106	-2.239840	0.087221

H	-6.692699	0.365853	0.545370

H	-2.669042	2.004483	0.442562

H	-3.263244	-2.228305	-0.041952

F	-5.454732	-3.375251	0.608801

F	-7.199273	-2.038368	0.704124

F	-6.268000	-2.499155	-1.228022

F	-6.585319	2.838645	0.357934

F	-5.328600	2.968010	2.150399

F	-4.560436	3.688983	0.228266

C	5.274897	-2.800060	-1.474143

H	4.970213	-1.882793	-2.008589

H	6.246808	-3.111184	-1.909391

C	5.531672	-2.481319	0.008219

C	6.045756	-1.086924	0.319635

H	5.202456	-0.373920	0.232848

H	6.446176	-1.047049	1.344416

H	6.817632	-0.768698	-0.405535

H	4.501480	-4.761561	-0.977243

O	5.338933	-3.324417	0.882074

**TS-1-Coor-E-T**

SCF Done: -2815.83814708 A.U.

Ni	4.028162	13.991485	6.357535

P	2.089073	14.905782	6.474360

O	3.503920	13.141541	8.060011

O	4.198355	16.707839	7.564940

O	-0.390120	16.657880	6.373619

O	1.781115	16.544763	4.004213

O	0.271395	12.518904	5.998056

N	1.415483	14.223592	7.892979

C	2.306533	13.433576	8.482866

C	1.898665	12.806916	9.788926

C	2.802166	11.983115	10.485419

C	2.423210	11.386588	11.699011

C	1.141979	11.603696	12.230307

C	0.240545	12.426823	11.533962

C	0.613633	13.026905	10.321658

C	1.920617	16.712426	6.880533

C	3.018561	17.407162	7.449893

C	2.897895	18.741840	7.892170

H	3.757922	19.269443	8.311644

C	1.660456	19.386319	7.792388

H	1.562417	20.422870	8.134819

C	0.540568	18.722093	7.277748

H	-0.423005	19.234895	7.231354

C	0.669332	17.387448	6.840043

C	0.982993	14.565915	5.024749

C	0.970654	15.437905	3.902777

C	0.175231	15.181583	2.769149

H	0.172940	15.866803	1.918389

C	-0.616848	14.026956	2.740233

H	-1.237718	13.819578	1.861229

C	-0.614018	13.126139	3.810096

H	-1.223882	12.221410	3.759874

C	0.193215	13.385395	4.939947

C	4.606607	14.843720	4.716067

H	3.935030	15.585098	4.259406

H	4.708840	13.981609	4.028395

C	5.980157	15.463724	5.029275

H	6.601896	15.697824	4.137412

H	5.829223	16.429952	5.557285

C	6.722614	14.584789	6.000979

C	8.230573	14.600790	6.093445

H	8.611066	15.638038	6.147068

H	8.574182	14.026060	6.967381

H	8.663178	14.155721	5.176053

O	6.048904	13.836444	6.745805

H	-0.077832	13.665241	9.764169

H	0.843879	11.125775	13.167105

H	3.794045	11.814917	10.056995

C	3.423063	10.548051	12.461219

C	-1.122826	12.711152	12.120210

F	-1.122526	13.858102	12.862639

F	-1.544170	11.707323	12.943882

F	-2.069706	12.869255	11.151391

F	2.813120	9.661800	13.300986

F	4.227361	9.832487	11.624638

F	4.244351	11.322853	13.231105

C	5.173032	17.194302	8.492867

H	5.898179	16.374473	8.609762

H	5.691937	18.097017	8.112858

H	4.714005	17.422887	9.473008

C	-1.689176	17.247135	6.413792

H	-1.757058	18.137688	5.758078

H	-2.373651	16.469568	6.043021

H	-1.974016	17.530193	7.445581

C	-0.577006	11.369883	5.999368

H	-0.382817	10.866553	6.958167

H	-1.645273	11.655390	5.943661

H	-0.336577	10.680614	5.166114

C	1.777863	17.486313	2.931240

H	2.144142	17.032707	1.989497

H	0.768011	17.908810	2.765269

H	2.463002	18.289044	3.242856

C	3.497391	11.680008	5.248222

C	4.696636	11.289319	5.732951

H	2.593259	11.668085	5.864484

H	3.362942	11.953284	4.194563

H	5.594653	11.267813	5.103628

H	4.822696	11.005626	6.782799

**1-Coor-E-T**

SCF Done: -2815.84483489 A.U.

Ni	1.190296	-1.949028	-0.539466

P	1.270532	0.186808	0.107525

O	-0.765849	-1.608995	-0.390316

O	1.601989	-2.031443	2.031788

O	1.638592	2.691521	1.743921

O	4.100610	1.041039	0.555096

O	0.451737	1.571107	-2.398176

N	-0.375381	0.661944	0.115862

C	-1.148159	-0.387706	-0.114769

C	-2.635254	-0.164608	-0.059663

C	-3.156240	1.111441	0.230895

C	-4.543636	1.312243	0.278097

C	-5.428100	0.247265	0.034712

C	-4.907809	-1.023452	-0.255517

C	-3.519595	-1.231248	-0.301856

C	1.692481	0.339085	1.896593

C	1.775628	-0.831783	2.689990

C	2.006364	-0.757570	4.079638

H	2.079806	-1.661125	4.688466

C	2.126424	0.500418	4.682394

H	2.300355	0.561968	5.762699

C	2.013204	1.680973	3.937348

H	2.093891	2.650029	4.435274

C	1.786706	1.597549	2.548439

C	2.242976	1.409590	-0.856999

C	3.600244	1.693484	-0.537153

C	4.361582	2.588413	-1.315981

H	5.402245	2.800211	-1.062426

C	3.775026	3.190007	-2.435702

H	4.365007	3.886809	-3.041983

C	2.459466	2.895763	-2.813650

H	2.034646	3.351470	-3.710777

C	1.706240	1.984094	-2.043743

C	3.137310	-2.149913	-0.721840

H	3.296736	-3.244104	-0.642516

H	3.614711	-1.669708	0.148820

C	3.755795	-1.599930	-2.023448

H	3.279180	-2.056260	-2.911800

C	5.261128	-1.894499	-2.044084

C	5.692360	-3.198905	-2.711195

H	5.543525	-3.120413	-3.805811

H	6.754516	-3.403494	-2.505720

H	5.069809	-4.045344	-2.366422

O	6.086680	-1.140115	-1.529235

H	3.620741	-0.506922	-2.080388

C	0.699611	-3.980594	-0.829932

H	1.527009	-4.607690	-0.477365

H	-0.250854	-4.054667	-0.290377

C	0.764245	-3.331697	-2.055922

H	-0.135091	-2.892395	-2.500566

H	1.637472	-3.437286	-2.708361

C	1.670081	3.984591	2.349371

H	0.865382	4.098979	3.101072

H	2.649242	4.188807	2.824978

H	1.507907	4.696457	1.526476

C	1.613064	-3.232813	2.809761

H	1.424722	-4.044518	2.092661

H	2.594177	-3.389230	3.297585

H	0.814775	-3.222175	3.575816

C	5.508362	1.142781	0.817592

H	5.678157	0.521293	1.709672

H	6.085480	0.739583	-0.032601

H	5.802560	2.186452	1.041151

C	-0.157885	2.163818	-3.545381

H	0.410333	1.943231	-4.470369

H	-1.157483	1.709235	-3.612312

H	-0.259509	3.260178	-3.429423

C	-5.842536	-2.164529	-0.585025

C	-5.092037	2.669237	0.655849

H	-6.508965	0.406884	0.070848

H	-3.105307	-2.218630	-0.521545

H	-2.455072	1.930789	0.413187

F	-6.020511	-2.292503	-1.933825

F	-5.360520	-3.358554	-0.136264

F	-7.078632	-1.987915	-0.036392

F	-5.206129	2.805528	2.010178

F	-4.287041	3.680478	0.222670

F	-6.334150	2.879919	0.130917

**TS-Isom-1**

SCF Done: -2815.829025 A.U.

Ni	1.161570	-2.282155	-0.459027

P	1.493564	0.016589	0.298257

O	-0.697731	-1.696933	-0.488273

O	0.812705	-1.992090	2.111209

O	3.171874	2.106417	2.025504

O	4.238144	-0.041661	-0.443881

O	0.541802	2.912390	-0.569334

N	-0.170736	0.454036	0.390914

C	-0.982033	-0.500388	-0.026763

C	-2.453953	-0.169468	0.005351

C	-2.871530	1.110137	0.416671

C	-4.233003	1.445469	0.411596

C	-5.197436	0.505761	0.009775

C	-4.780646	-0.773843	-0.390908

C	-3.416808	-1.111910	-0.397957

C	2.018501	0.035261	2.073445

C	1.570431	-1.081885	2.824327

C	1.872170	-1.245056	4.187879

H	1.516663	-2.114049	4.746804

C	2.645050	-0.262540	4.821158

H	2.891531	-0.373125	5.883174

C	3.105165	0.860396	4.123168

H	3.697224	1.615266	4.645952

C	2.785879	1.016565	2.755614

C	2.367275	1.382611	-0.585093

C	3.708418	1.108459	-0.971159

C	4.434635	1.971739	-1.815078

H	5.459470	1.737588	-2.111496

C	3.822861	3.149569	-2.261569

H	4.376643	3.829719	-2.919004

C	2.518703	3.480193	-1.872705

H	2.074200	4.418116	-2.213675

C	1.795180	2.614131	-1.019749

C	0.854639	-2.945938	-2.271573

H	-0.135685	-3.421198	-2.200973

H	1.638889	-3.662262	-2.559545

C	0.867058	-1.724867	-3.172578

H	0.339175	-1.975035	-4.125362

C	2.250483	-1.215828	-3.590912

C	2.270004	0.117904	-4.326059

H	3.234981	0.256306	-4.837566

H	1.438822	0.194013	-5.050809

H	2.139880	0.937066	-3.592637

O	3.284468	-1.846844	-3.368746

H	0.275581	-0.888997	-2.751672

C	2.993417	-3.044225	-0.232219

H	3.516784	-2.947827	-1.190806

H	3.450492	-2.540133	0.626844

C	2.017519	-4.051226	-0.042334

H	1.690214	-4.313585	0.972081

H	1.822118	-4.815311	-0.802015

C	3.926554	3.126294	2.679170

H	3.360766	3.578738	3.516980

H	4.893943	2.741009	3.056979

H	4.113269	3.888857	1.908222

C	0.041771	-2.956122	2.840252

H	-0.576135	-2.458250	3.609766

H	-0.606529	-3.433697	2.091373

H	0.687134	-3.717374	3.319925

C	5.459547	-0.533925	-1.011266

H	5.646374	-1.497458	-0.514640

H	5.349902	-0.694167	-2.099396

H	6.305245	0.150307	-0.804694

C	-0.097983	4.088995	-1.063889

H	-0.184662	4.075011	-2.168225

H	-1.104744	4.082959	-0.619666

H	0.436095	5.008737	-0.752791

C	-5.796740	-1.776986	-0.886500

C	-4.652328	2.817332	0.885425

H	-6.258660	0.767398	0.007818

H	-3.084843	-2.103969	-0.715732

H	-2.101832	1.823456	0.725843

F	-5.458669	-3.051773	-0.539171

F	-7.042686	-1.532314	-0.388326

F	-5.901195	-1.753887	-2.248673

F	-4.654293	2.904266	2.247136

F	-3.799574	3.787614	0.433133

F	-5.904558	3.148652	0.460786

**1-Coor-E-C**

SCF Done: -2815.83449074 A.U.

Ni	-1.298864	2.059044	-0.124494

P	-1.736087	-0.208260	0.008910

O	0.517139	1.409205	0.074275

O	-2.047957	1.409093	2.436284

O	-2.482066	-3.030528	0.859679

O	-4.670529	-0.605906	0.015903

O	-0.848695	-1.213013	-2.693400

N	-0.143960	-0.867167	-0.007930

C	0.738804	0.109706	0.076460

C	2.185986	-0.287631	0.194456

C	2.554219	-1.645763	0.126499

C	3.902764	-2.016898	0.227438

C	4.900392	-1.042932	0.406747

C	4.531999	0.308940	0.481698

C	3.183500	0.687604	0.373622

C	-2.323228	-0.813810	1.654020

C	-2.369248	0.102557	2.734206

C	-2.714259	-0.316824	4.036175

H	-2.754833	0.393190	4.865126

C	-2.995212	-1.670103	4.261112

H	-3.259434	-2.001543	5.271856

C	-2.932951	-2.610831	3.225227

H	-3.141997	-3.663166	3.431798

C	-2.587984	-2.181806	1.926465

C	-2.748988	-1.033694	-1.292132

C	-4.159222	-1.130853	-1.148646

C	-4.966088	-1.714992	-2.144236

H	-6.047020	-1.801484	-2.012672

C	-4.364343	-2.175113	-3.322771

H	-4.985624	-2.630130	-4.102454

C	-2.987907	-2.037049	-3.533462

H	-2.545229	-2.373722	-4.473566

C	-2.186377	-1.445580	-2.532405

C	-0.584459	3.894544	0.010100

H	-0.326728	3.872069	1.088470

H	-1.377906	4.639954	-0.172171

C	0.652084	4.185261	-0.828549

H	1.335591	3.313468	-0.833776

C	-2.693723	2.652750	-1.488618

H	-2.299058	3.549827	-1.979463

H	-2.870150	1.778294	-2.128170

C	-3.248934	2.703470	-0.203438

H	-3.890655	1.896552	0.165821

H	-3.307037	3.654303	0.340465

C	5.581593	1.364739	0.742589

C	4.292587	-3.469606	0.077596

C	-0.228262	-1.640591	-3.906241

H	0.832543	-1.367889	-3.803134

H	-0.314112	-2.736213	-4.042308

H	-0.657273	-1.126031	-4.788420

C	-1.964607	2.346354	3.513660

H	-1.637771	3.290737	3.053219

H	-2.948098	2.493703	4.000367

H	-1.222304	2.025869	4.269138

C	-2.652360	-4.429118	1.091451

H	-1.914305	-4.811189	1.822868

H	-3.675283	-4.664119	1.446048

H	-2.485465	-4.909640	0.115920

C	-6.076630	-0.715875	0.245401

H	-6.402063	-1.773749	0.264284

H	-6.249682	-0.262673	1.232971

H	-6.657780	-0.166695	-0.520722

F	5.668191	1.668866	2.071266

F	6.822285	0.962015	0.344654

F	4.476282	-3.805280	-1.234837

F	3.335758	-4.306087	0.568927

F	5.459408	-3.753553	0.725095

F	5.299213	2.529077	0.089714

H	5.950565	-1.335182	0.488846

H	2.890585	1.739192	0.428467

H	1.764423	-2.391595	-0.000626

C	0.402605	4.619777	-2.270353

C	1.641662	4.725868	-3.158958

H	1.980134	3.707584	-3.432331

H	1.400872	5.278482	-4.080452

H	2.483738	5.210453	-2.632202

H	1.255544	5.008263	-0.375242

O	-0.715674	4.878643	-2.716420

**TS-1-Ins-E-C**

SCF Done: -2815.8245673 A.U.

Ni	1.598755	-1.575669	-0.783864

P	1.763201	0.260561	0.361505

O	-0.276775	-1.013672	-0.951849

O	4.600176	0.812510	1.228394

O	0.452937	-0.807346	2.800856

O	2.135843	3.022964	1.417510

O	2.883831	0.359793	-2.425328

N	0.136666	0.799048	0.501374

C	-0.638945	0.030471	-0.247516

C	-2.103827	0.362427	-0.297919

C	-2.978396	-0.447353	-1.043055

C	-4.356121	-0.175539	-1.060295

C	-4.874660	0.913798	-0.344891

C	-3.997685	1.730122	0.392346

C	-2.622156	1.457718	0.420695

C	2.488198	0.138784	2.053731

C	3.850578	0.442005	2.317327

C	4.386720	0.346052	3.617554

H	5.430781	0.600680	3.813312

C	3.566529	-0.095685	4.663342

H	3.978831	-0.170167	5.676051

C	2.238545	-0.471591	4.432031

H	1.627422	-0.848697	5.255114

C	1.711492	-0.380735	3.125725

C	2.535782	1.704195	-0.496492

C	2.569667	2.983452	0.121866

C	3.026229	4.120382	-0.575213

H	3.050372	5.101196	-0.094504

C	3.447472	3.980899	-1.903699

H	3.801283	4.862291	-2.450737

C	3.419034	2.739654	-2.549872

H	3.747099	2.660237	-3.588619

C	2.957372	1.605704	-1.846918

C	1.039862	-3.243735	-1.977815

H	1.479294	-3.946227	-2.698638

H	0.553690	-2.446344	-2.571694

C	0.039654	-3.987085	-1.098073

H	0.565136	-4.620566	-0.347958

H	-0.591148	-3.287506	-0.523393

C	-0.851236	-4.954431	-1.905028

C	-2.216767	-5.263298	-1.299737

H	-2.683782	-6.111719	-1.823717

H	-2.135437	-5.480403	-0.218311

H	-2.872409	-4.376110	-1.396296

O	-0.485478	-5.447750	-2.968421

C	2.953540	-3.140397	-1.255727

H	2.827575	-4.158040	-0.860732

H	3.414570	-3.124161	-2.251622

C	3.397546	-2.123542	-0.328745

H	4.136293	-1.386283	-0.664989

H	3.442124	-2.364299	0.742508

C	-4.537757	2.940130	1.119849

C	-5.261503	-1.066697	-1.878166

C	3.138759	0.253804	-3.828791

H	4.193329	0.489668	-4.071201

H	2.930799	-0.796087	-4.084540

H	2.469992	0.914838	-4.412213

C	5.963353	1.181715	1.439489

H	6.346273	1.468133	0.448477

H	6.049553	2.043066	2.129773

H	6.557840	0.334882	1.834691

C	-0.403893	-1.251990	3.853082

H	-0.003555	-2.156368	4.352184

H	-0.565340	-0.457505	4.607004

H	-1.360914	-1.495277	3.368102

C	2.026615	4.295705	2.055239

H	1.345393	4.968779	1.500018

H	1.608009	4.088763	3.051366

H	3.014633	4.784400	2.166798

F	-4.500352	4.056492	0.333812

F	-5.837197	2.767109	1.499767

F	-4.999150	-2.390360	-1.646834

F	-5.091259	-0.864809	-3.216304

F	-6.579253	-0.860763	-1.601685

F	-3.816952	3.222139	2.242250

H	-5.948376	1.117728	-0.349306

H	-1.930176	2.073211	1.002531

H	-2.562621	-1.293188	-1.597116

**1-beta-T**

SCF Done: -2815.85941444 A.U.

Ni	-0.902229	-2.211903	-0.477969

P	-1.343056	-0.141596	-0.422260

O	0.949900	-1.691658	-0.318871

N	0.204066	0.547832	-0.167056

C	1.132446	-0.401218	-0.185859

C	2.561929	0.040800	-0.044364

C	2.878641	1.403550	0.118408

C	4.215376	1.804224	0.262713

C	5.251369	0.854673	0.246052

C	4.934388	-0.503215	0.083825

C	3.598362	-0.910717	-0.060742

C	-1.874570	0.702127	-1.975376

C	-1.823707	0.017927	-3.217054

C	-2.142886	0.676612	-4.423928

H	-2.110810	0.146086	-5.378108

C	-2.492869	2.031640	-4.392067

H	-2.736747	2.544739	-5.329376

C	-2.523615	2.747942	-3.189264

H	-2.782567	3.809290	-3.192080

C	-2.205467	2.083639	-1.986811

C	-2.502725	0.352257	0.921825

C	-3.902924	0.338123	0.682555

C	-4.828006	0.679446	1.688687

H	-5.901814	0.679270	1.488195

C	-4.352145	1.010131	2.963973

H	-5.065201	1.276724	3.752388

C	-2.983843	0.982791	3.255584

H	-2.638771	1.214748	4.265625

C	-2.061820	0.632048	2.244748

C	-2.592197	-3.062786	-0.498015

H	-3.124885	-2.968138	-1.455872

H	-3.235257	-2.824790	0.364044

C	-1.716883	-4.288106	-0.336082

H	-1.802038	-4.983921	-1.194084

C	-1.838268	-5.026750	1.010806

H	-1.809876	-4.288944	1.833390

H	-2.830392	-5.510071	1.067026

H	-0.585350	-3.991329	-0.420332

H	6.290760	1.168245	0.373480

H	3.339049	-1.966203	-0.178297

H	2.060409	2.129059	0.137141

C	6.038525	-1.532573	0.011312

C	4.546691	3.273847	0.384287

F	7.172723	-1.111992	0.642218

F	5.666969	-2.714806	0.581292

F	6.382352	-1.809421	-1.281657

F	5.725835	3.478058	1.040791

F	3.579095	3.958506	1.058429

F	4.668184	3.864742	-0.841269

C	-0.743726	-6.078932	1.231283

C	0.243115	-7.821484	2.890326

H	-0.033131	-8.428015	3.766428

H	0.492597	-8.481320	2.038607

H	1.158688	-7.244775	3.123879

O	-2.185037	2.707148	-0.769813

O	-0.719944	0.515698	2.474475

O	-4.277769	-0.040177	-0.583195

O	-1.435875	-1.299675	-3.174614

H	-0.728060	-6.814465	0.398018

H	0.263869	-5.612272	1.217223

C	-0.895684	-6.867207	2.537505

O	-1.877004	-6.735272	3.263743

C	-2.433521	4.112344	-0.725613

H	-2.331569	4.393807	0.333007

H	-1.694939	4.673193	-1.330277

H	-3.455932	4.358082	-1.074075

C	-5.669908	-0.052193	-0.899723

H	-6.223978	-0.774158	-0.268611

H	-6.121069	0.952913	-0.788538

H	-5.729055	-0.364412	-1.953124

C	-0.225821	0.824087	3.777830

H	-0.444276	1.872981	4.058265

H	-0.644268	0.144112	4.545703

H	0.863389	0.681823	3.717047

C	-1.238174	-1.981873	-4.414931

H	-0.489213	-1.465906	-5.045767

H	-0.862617	-2.979756	-4.142681

H	-2.185029	-2.087081	-4.980014

**TS-1-Ins-E-T**

SCF Done: -2815.80725992 A.U.

Ni	1.116078	-2.009333	-0.156174

P	1.309523	0.213414	0.312652

O	-0.744565	-1.608364	-0.081748

O	2.105197	-1.607534	2.543919

O	1.371789	2.959641	1.530230

O	4.176323	0.814288	0.566520

O	0.471126	1.665638	-2.238338

N	-0.337332	0.703561	0.257057

C	-1.108260	-0.354361	0.085297

C	-2.595036	-0.128802	0.075131

C	-3.113738	1.168780	0.250427

C	-4.500182	1.381243	0.238651

C	-5.385746	0.306304	0.050164

C	-4.867154	-0.986226	-0.125314

C	-3.480119	-1.206482	-0.111762

C	1.746149	0.687264	2.049722

C	2.041031	-0.312739	3.009903

C	2.254807	0.015248	4.365699

H	2.482240	-0.757691	5.103223

C	2.170199	1.354808	4.764642

H	2.332446	1.611921	5.817700

C	1.878146	2.371203	3.846942

H	1.812682	3.408242	4.184591

C	1.664829	2.034581	2.493568

C	2.302164	1.344941	-0.768895

C	3.687525	1.521144	-0.509455

C	4.496210	2.348750	-1.313343

H	5.556024	2.489479	-1.088640

C	3.921950	2.987520	-2.420332

H	4.542093	3.634782	-3.050888

C	2.575256	2.792267	-2.748492

H	2.155692	3.278554	-3.632279

C	1.773285	1.956712	-1.939250

C	3.156843	-2.384140	-0.760870

H	3.965727	-3.126679	-0.696097

H	3.418537	-1.587395	-0.050898

C	3.019017	-1.918821	-2.212610

H	1.966284	-1.996428	-2.569512

H	3.252692	-0.841576	-2.315939

C	3.865303	-2.723153	-3.207819

C	3.934657	-2.172592	-4.628499

H	4.524930	-1.236221	-4.636695

H	4.412168	-2.905882	-5.296464

H	2.927772	-1.915733	-5.006220

O	4.447510	-3.758927	-2.888823

C	2.040145	-4.018252	-0.371464

H	2.442883	-4.454210	-1.294546

H	2.586283	-4.344716	0.523754

C	0.616961	-3.890216	-0.270095

H	0.115970	-4.047908	0.694138

H	-0.011206	-4.045099	-1.156457

C	-5.802157	-2.142429	-0.396129

C	-5.045867	2.767237	0.493440

C	-0.131378	2.321139	-3.353713

H	-0.116976	3.422053	-3.234937

H	0.364174	2.050011	-4.307171

H	-1.173515	1.969016	-3.370583

C	2.240655	-2.656594	3.505096

H	3.216537	-2.610104	4.027033

H	1.423535	-2.630701	4.251190

H	2.180986	-3.592389	2.928931

C	1.187731	4.316653	1.932318

H	0.365937	4.413899	2.667859

H	2.114986	4.747115	2.359587

H	0.923541	4.860957	1.013381

C	5.543284	1.003400	0.935417

H	6.231687	0.665664	0.136098

H	5.753529	2.062828	1.177554

H	5.693629	0.386192	1.833710

F	-7.050155	-1.919287	0.107938

F	-5.949350	-2.364005	-1.736665

F	-6.281139	2.939132	-0.060841

F	-4.230564	3.734857	-0.016166

F	-5.172433	3.020955	1.829704

F	-5.341416	-3.304507	0.147338

H	-6.465820	0.474635	0.040478

H	-3.068045	-2.210535	-0.240941

H	-2.409350	1.993481	0.391627

**1-beta-C**

SCF Done: -2815.85507638 A.U.

Ni	0.392310	2.114917	0.579809

P	0.718029	-0.076022	0.453076

O	-1.320750	1.755644	-0.080433

N	-0.832841	-0.571490	-0.098797

C	-1.612218	0.481640	-0.269495

C	-3.018793	0.231108	-0.735383

C	-3.912290	1.303074	-0.917537

C	-5.222175	1.060615	-1.361678

C	-5.654535	-0.248482	-1.627273

C	-4.760738	-1.317133	-1.444385

C	-3.450200	-1.082432	-1.001971

C	2.016641	-0.556565	-0.776252

C	3.362681	-0.270928	-0.417076

C	4.434879	-0.489240	-1.305651

H	5.461877	-0.257767	-1.016075

C	4.160315	-1.000013	-2.580160

H	4.986195	-1.177210	-3.278591

C	2.848475	-1.283994	-2.979895

H	2.661703	-1.681409	-3.980251

C	1.776873	-1.062506	-2.084962

C	0.999270	-1.132715	1.935425

C	1.213933	-2.533255	1.835399

C	1.382461	-3.328248	2.988855

H	1.550939	-4.404767	2.909964

C	1.316891	-2.721940	4.249060

H	1.443360	-3.337194	5.147309

C	1.082774	-1.348179	4.388538

H	1.026742	-0.905096	5.385303

C	0.919212	-0.559038	3.230744

C	0.325194	4.046259	0.615441

H	-0.403424	4.413439	1.351611

H	0.191220	4.458150	-0.394344

C	1.696056	3.740814	1.082775

H	1.863486	4.021292	2.139541

C	2.902074	4.110009	0.194281

H	2.981712	5.212553	0.154983

H	3.834493	3.743580	0.659954

H	1.856409	2.523159	1.208171

H	-6.674811	-0.434166	-1.972934

H	-2.738646	-1.900663	-0.857870

H	-3.567939	2.318351	-0.704497

C	-5.226579	-2.737050	-1.669863

C	-6.150534	2.225524	-1.618338

F	-5.628766	-3.326616	-0.505818

F	-6.282835	-2.797824	-2.531710

F	-4.233319	-3.518319	-2.184848

F	-7.462493	1.863042	-1.523480

F	-5.971347	2.742071	-2.870790

F	-5.941050	3.244168	-0.737451

C	2.825877	3.528899	-1.225614

C	4.200740	2.999070	-3.360562

H	5.228300	2.812098	-3.707838

H	3.569956	2.119348	-3.590520

H	3.770541	3.857995	-3.908804

O	0.666753	0.792427	3.284299

O	1.233338	-3.040624	0.566568

O	0.476042	-1.306472	-2.422506

O	3.529514	0.234975	0.849668

H	2.297148	2.545919	-1.192285

H	2.217992	4.152547	-1.907828

C	4.195235	3.257794	-1.859877

O	5.228602	3.207519	-1.192518

C	0.423343	1.375848	4.566507

H	-0.418637	0.877764	5.084155

H	1.323586	1.339068	5.211006

H	0.159835	2.425379	4.366327

C	0.194318	-1.780623	-3.738232

H	0.526256	-1.062262	-4.513766

H	0.664567	-2.765466	-3.929938

H	-0.900120	-1.884870	-3.780777

C	4.855903	0.592417	1.261993

H	5.524784	-0.289630	1.266537

H	5.277217	1.383658	0.614248

H	4.748590	0.969569	2.290333

C	1.418167	-4.446873	0.405246

H	0.606087	-5.021148	0.891835

H	1.391103	-4.622571	-0.680362

H	2.395339	-4.779397	0.807046

### **5.5.2 Geometries of Ni-7**

**1-cycle5-T**

SCF Done: -3503.89595452 A.U.

Ni	1.174865	-2.433468	-0.619651

P	0.995645	-0.336045	-0.270298

O	-0.747114	-2.444620	-0.276261

O	2.354566	-1.736140	2.091193

O	0.792749	2.550489	0.623673

O	3.722591	1.029003	-0.494923

O	-0.258007	0.168290	-2.945167

N	-0.691141	-0.092346	-0.116393

C	-1.302637	-1.276848	-0.116217

C	-2.788432	-1.283945	0.113437

C	-3.478599	-2.509344	0.176114

C	-4.860797	-2.529054	0.419093

C	-5.568231	-1.330900	0.610261

C	-4.878620	-0.109303	0.544045

C	-3.498111	-0.081219	0.290251

C	1.579989	0.399289	1.327026

C	2.157359	-0.387167	2.352599

C	2.416978	0.139351	3.631651

H	2.848428	-0.505239	4.402666

C	2.097932	1.475018	3.904346

H	2.295629	1.886248	4.900299

C	1.524949	2.287647	2.917696

H	1.276648	3.334772	3.114571

C	1.270652	1.744326	1.647975

C	1.631659	0.756691	-1.611438

C	2.902514	1.380731	-1.560144

C	3.348837	2.243563	-2.574617

H	4.335804	2.707842	-2.497943

C	2.524417	2.474751	-3.684962

H	2.869584	3.140014	-4.484204

C	1.290862	1.825453	-3.809248

H	0.664599	1.959549	-4.695734

C	0.868237	0.952952	-2.789529

C	3.025255	-2.435559	-1.159128

H	3.685878	-1.683406	-0.699768

H	2.964369	-2.228691	-2.247321

C	3.534607	-3.869067	-0.901068

H	4.397607	-4.184091	-1.527642

H	3.883722	-3.964915	0.150532

C	2.386574	-4.829497	-1.033773

C	2.590377	-6.300825	-1.288831

H	3.335060	-6.717945	-0.585767

H	1.639064	-6.847481	-1.201616

H	3.002281	-6.445825	-2.306411

O	1.224154	-4.369110	-0.910722

H	-2.954005	0.865582	0.224740

H	-6.642833	-1.349186	0.809117

H	-2.914221	-3.435836	0.039957

C	-5.601846	-3.846295	0.418084

C	-5.606231	1.186849	0.812768

F	-5.396524	1.620100	2.098754

F	-6.954882	1.065590	0.655800

F	-5.184327	2.188661	-0.006908

F	-5.972747	-4.212340	-0.844878

F	-4.837200	-4.859306	0.914695

F	-6.743314	-3.789662	1.164644

C	4.669750	1.931990	-0.015486

C	5.967419	1.440923	0.202196

C	4.328219	3.253507	0.322578

C	6.935733	2.286520	0.767320

H	6.200211	0.408386	-0.077898

C	5.309546	4.088612	0.877294

H	3.304016	3.603718	0.162002

C	6.612851	3.611772	1.102104

H	7.949753	1.906990	0.937048

H	5.048034	5.118476	1.146095

H	7.371817	4.270073	1.538478

C	-0.310219	3.370979	0.859221

C	-0.305484	4.630981	0.238953

C	-1.418158	2.935453	1.607419

C	-1.426325	5.466107	0.374404

H	0.568636	4.937159	-0.345173

C	-2.531244	3.780903	1.731989

H	-1.409961	1.937287	2.054427

C	-2.539544	5.047167	1.121145

H	-1.424241	6.450422	-0.107706

H	-3.409422	3.428782	2.282959

H	-3.414351	5.698858	1.219547

C	3.448841	-2.398716	2.633287

C	4.735219	-1.827812	2.676765

C	3.232943	-3.721931	3.058577

C	5.805420	-2.595908	3.161227

H	4.889449	-0.799886	2.333413

C	4.316729	-4.478346	3.532026

H	2.219392	-4.133638	3.015405

C	5.604969	-3.919780	3.589060

H	6.807823	-2.153749	3.196424

H	4.147600	-5.507944	3.867728

H	6.446786	-4.510953	3.965191

C	-1.402201	0.707418	-3.530971

C	-2.095808	-0.109944	-4.437466

C	-1.893168	1.974904	-3.171679

C	-3.295724	0.354320	-4.998260

H	-1.690098	-1.096264	-4.684552

C	-3.089981	2.427081	-3.747797

H	-1.353525	2.581541	-2.438079

C	-3.793922	1.623937	-4.661575

H	-3.841061	-0.282228	-5.704264

H	-3.481743	3.410572	-3.464518

H	-4.730514	1.982781	-5.101964

**TS-1-Coor-CO-T**

SCF Done: -3617.20681674 A.U.

Ni	-0.508088	2.661235	0.799387

P	-1.042530	0.548559	0.288625

O	1.317316	1.930454	0.572504

N	0.464040	-0.261319	0.366316

C	1.440926	0.634702	0.456615

C	2.852344	0.118902	0.398476

C	3.105750	-1.260013	0.261935

C	4.425200	-1.727033	0.172038

C	5.506279	-0.830609	0.229408

C	5.251580	0.540771	0.377962

C	3.932441	1.017636	0.458445

C	-2.218341	-0.340223	1.384689

C	-3.576007	-0.615681	1.075813

C	-4.400777	-1.326727	1.962910

H	-5.439194	-1.533476	1.691808

C	-3.883358	-1.742806	3.198757

H	-4.529236	-2.290008	3.894310

C	-2.574295	-1.425325	3.575244

H	-2.179390	-1.699659	4.557259

C	-1.766059	-0.703948	2.678109

C	-1.484432	0.208777	-1.474404

C	-1.747135	-1.105610	-1.936012

C	-1.910786	-1.396364	-3.298765

H	-2.122326	-2.425012	-3.605737

C	-1.800236	-0.361586	-4.238308

H	-1.941776	-0.576391	-5.303066

C	-1.509558	0.944223	-3.826832

H	-1.396981	1.754615	-4.552786

C	-1.329488	1.216670	-2.457446

C	-2.338531	3.138393	1.400600

H	-2.282139	4.060350	2.002204

H	-2.591857	2.299665	2.072501

C	-3.386554	3.298549	0.306924

C	-3.194724	4.491397	-0.624063

C	-4.110176	4.549523	-1.843454

H	-3.712819	3.861015	-2.613715

H	-5.136231	4.214339	-1.607859

H	-4.129238	5.569649	-2.258052

O	-2.350319	5.363253	-0.417894

H	3.722725	2.085791	0.565081

H	6.533488	-1.197196	0.165255

H	2.257890	-1.950534	0.229735

C	4.670445	-3.201322	-0.048957

C	6.388765	1.535009	0.450079

F	7.608769	0.933204	0.362226

F	6.362986	2.236705	1.619397

F	6.312479	2.450481	-0.559090

F	4.461236	-3.549599	-1.360649

F	5.947347	-3.565039	0.255074

F	3.831836	-3.974336	0.694935

C	0.026692	4.325948	1.144800

O	0.410772	5.392119	1.388274

H	-3.504369	2.386158	-0.308578

H	-4.390837	3.451367	0.772279

O	-1.903693	-2.108888	-0.988357

O	-1.018317	2.506241	-2.039081

O	-0.529562	-0.218013	3.057114

O	-4.052281	-0.100246	-0.118349

C	0.326436	-1.012622	3.821224

C	1.038431	-0.370808	4.846344

C	0.534240	-2.371569	3.528788

C	1.969088	-1.106915	5.595994

C	1.461841	-3.094840	4.294032

C	2.180100	-2.469678	5.327786

H	0.858140	0.691782	5.038244

H	-0.010579	-2.842155	2.704798

H	2.528913	-0.609476	6.395968

H	1.633611	-4.153102	4.067030

H	2.906238	-3.040682	5.916367

C	-5.265895	-0.566778	-0.637718

C	-6.350214	0.321608	-0.686066

C	-5.358298	-1.859965	-1.177695

C	-7.549455	-0.094440	-1.287577

C	-6.564479	-2.264914	-1.769662

C	-7.660571	-1.386499	-1.826803

H	-6.242770	1.322728	-0.256598

H	-4.485520	-2.518528	-1.139159

H	-8.399681	0.595427	-1.330666

H	-6.643902	-3.270882	-2.197071

H	-8.597818	-1.707661	-2.294078

C	-1.133948	-3.269742	-1.096881

C	0.194766	-3.250253	-1.555466

C	-1.724965	-4.459811	-0.642635

C	0.932413	-4.444432	-1.559712

C	-0.974570	-5.646135	-0.654334

C	0.352565	-5.644840	-1.114271

H	0.646059	-2.306073	-1.872534

H	-2.755776	-4.440545	-0.273741

H	1.977990	-4.423781	-1.884476

H	-1.434707	-6.575718	-0.300371

H	0.936232	-6.571579	-1.117174

C	0.014583	3.192757	-2.704698

C	-0.090328	4.591898	-2.751379

C	1.137740	2.529500	-3.228568

C	0.944801	5.331582	-3.344794

C	2.158085	3.286375	-3.825558

C	2.066922	4.686439	-3.890211

H	-0.955418	5.088094	-2.301038

H	1.216338	1.440374	-3.165343

H	0.867635	6.424235	-3.377000

H	3.036684	2.771789	-4.230935

H	2.869645	5.270228	-4.353274

**1-Coor-CO-T**

SCF Done: -3617.204525 A.U.

Ni	0.451048	-2.523478	-0.103772

P	0.964146	-0.356592	-0.160274

O	-1.357155	-1.793244	0.176082

O	1.893932	-1.828545	2.338166

O	1.647206	2.539462	0.412036

O	3.968751	0.172981	-0.388304

O	-0.034307	0.071371	-2.941652

N	-0.568244	0.407951	-0.152443

C	-1.513334	-0.499089	0.063966

C	-2.925786	-0.006386	0.219079

C	-3.961351	-0.923708	0.479345

C	-5.278209	-0.469817	0.652702

C	-5.575167	0.900295	0.576552

C	-4.540939	1.813752	0.313862

C	-3.223447	1.366810	0.128704

C	1.697472	0.351058	1.379926

C	1.955360	-0.456877	2.515460

C	2.303452	0.109198	3.755936

H	2.507086	-0.542766	4.609531

C	2.386061	1.501972	3.877152

H	2.661591	1.943438	4.841186

C	2.142662	2.335346	2.777764

H	2.218495	3.423558	2.858937

C	1.805898	1.753790	1.544873

C	1.913586	0.320661	-1.582812

C	3.306398	0.584831	-1.533125

C	3.987129	1.158348	-2.620106

H	5.060025	1.354718	-2.545805

C	3.278896	1.449504	-3.795197

H	3.808313	1.889050	-4.647797

C	1.919337	1.137589	-3.911308

H	1.370728	1.314853	-4.840709

C	1.256729	0.551368	-2.817364

C	2.269288	-3.108151	-0.630879

H	2.464217	-4.093748	-0.177025

C	-0.053004	-4.221479	0.181335

O	-0.411536	-5.264924	0.536046

H	-2.413746	2.069609	-0.087753

H	-6.600066	1.252052	0.720652

H	-3.717182	-1.986916	0.549435

C	-4.831248	3.296419	0.299460

C	-6.392736	-1.469366	0.866126

F	-6.140556	3.562441	0.031198

F	-4.078071	3.955971	-0.622525

F	-4.554219	3.870148	1.515686

F	-6.878460	-1.937219	-0.321015

F	-5.971820	-2.551921	1.577865

F	-7.446176	-0.919417	1.537231

C	2.379510	-3.180047	-2.151186

H	3.431504	-3.416091	-2.442269

H	2.184187	-2.200237	-2.631155

C	1.525739	-4.239187	-2.846907

C	1.511615	-4.196187	-4.373038

H	0.888779	-3.343494	-4.705755

H	2.523710	-4.033392	-4.786258

H	1.082321	-5.127193	-4.774309

H	2.995347	-2.387832	-0.222543

O	0.885146	-5.088352	-2.229203

C	0.861368	3.690167	0.477219

C	-0.350847	3.728680	1.188475

C	1.292011	4.792521	-0.278833

C	-1.134283	4.891955	1.138057

H	-0.678970	2.847538	1.747173

C	0.497813	5.949808	-0.318035

H	2.235248	4.727994	-0.831204

C	-0.713826	6.005459	0.389956

H	-2.095341	4.911100	1.662090

H	0.833047	6.811669	-0.906251

H	-1.333229	6.907969	0.352778

C	5.156471	0.800934	-0.003875

C	5.221930	2.192699	0.180052

C	6.255825	-0.023056	0.280364

C	6.418240	2.760583	0.643645

H	4.338954	2.805782	-0.025063

C	7.443043	0.559217	0.752406

H	6.166255	-1.104065	0.133368

C	7.530251	1.949771	0.930175

H	6.475949	3.844828	0.792619

H	8.304161	-0.079996	0.977334

H	8.459348	2.400329	1.295679

C	1.596651	-2.651216	3.430937

C	2.478860	-3.707528	3.701864

C	0.409421	-2.486120	4.165181

C	2.165466	-4.614206	4.727811

H	3.390330	-3.809650	3.104587

C	0.114427	-3.394838	5.192740

H	-0.271293	-1.663801	3.923559

C	0.987985	-4.459081	5.477325

H	2.848504	-5.444211	4.940439

H	-0.812446	-3.275884	5.765046

H	0.747139	-5.168463	6.276316

C	-0.999042	0.843673	-3.590349

C	-1.919335	0.157363	-4.398060

C	-1.102585	2.230308	-3.384996

C	-2.954311	0.874784	-5.017569

H	-1.817829	-0.925360	-4.524157

C	-2.138821	2.933453	-4.018243

H	-0.394791	2.739878	-2.724266

C	-3.064854	2.263370	-4.835955

H	-3.675905	0.341781	-5.646867

H	-2.229750	4.013129	-3.854875

H	-3.873639	2.819757	-5.321752

**TS-1-Ins-CO-T**

SCF Done: -3617.17612183 A.U.

Ni	0.338581	-2.473787	0.217812

P	0.886796	-0.270691	-0.147109

O	-1.422197	-1.791607	0.325329

N	-0.673227	0.439282	-0.002876

C	-1.590565	-0.491677	0.201418

C	-3.023228	-0.043343	0.289486

C	-3.343400	1.326378	0.238572

C	-4.684626	1.737251	0.288850

C	-5.717418	0.792445	0.402166

C	-5.394477	-0.572739	0.467113

C	-4.056265	-0.992477	0.409616

C	2.029228	0.519598	1.067964

C	1.618372	1.060921	2.314522

C	2.556687	1.569626	3.233245

H	2.202145	2.006073	4.171144

C	3.924288	1.512294	2.938066

H	4.650146	1.908761	3.656740

C	4.373121	0.934644	1.743372

H	5.439053	0.851472	1.512570

C	3.421693	0.438545	0.837679

C	1.384761	0.372003	-1.802495

C	1.565946	-0.511281	-2.893083

C	1.711764	-0.040784	-4.209897

H	1.855812	-0.751358	-5.028489

C	1.680184	1.339971	-4.447804

H	1.798189	1.712848	-5.471249

C	1.530238	2.250060	-3.393475

H	1.528779	3.329759	-3.569576

C	1.394693	1.760301	-2.082792

C	1.973496	-3.888358	0.131683

H	2.112490	-4.568230	-0.721865

C	0.194380	-4.200033	0.348788

O	-0.188693	-5.313463	0.509013

C	2.618211	-4.405878	1.416542

H	3.713609	-4.527528	1.289221

H	2.235479	-5.419642	1.659228

C	2.365514	-3.523113	2.640549

C	3.164051	-3.846222	3.893051

H	3.155218	-4.931426	4.103092

H	4.221621	-3.556988	3.739953

H	2.763892	-3.291164	4.754832

H	2.399352	-2.912375	-0.168108

O	1.552060	-2.597956	2.612929

H	-3.796350	-2.053424	0.450127

H	-6.760844	1.115822	0.439347

H	-2.529127	2.053118	0.161401

C	-6.492519	-1.594370	0.658931

C	-5.009858	3.206958	0.158515

F	-7.683617	-1.160547	0.152291

F	-6.699179	-1.864975	1.981231

F	-6.195248	-2.777684	0.052869

F	-6.269655	3.497983	0.589066

F	-4.142739	3.983694	0.864971

F	-4.932510	3.621563	-1.147297

O	1.651223	-1.867003	-2.598906

O	1.342493	2.622254	-1.000546

O	0.264275	1.119824	2.566403

O	3.819859	-0.275144	-0.294295

C	1.185605	-2.797499	-3.530476

C	2.042269	-3.858109	-3.865465

C	-0.122816	-2.733022	-4.041640

C	1.578898	-4.872106	-4.720147

C	-0.568662	-3.748657	-4.900668

C	0.276130	-4.819740	-5.241744

H	3.057992	-3.872915	-3.456912

H	-0.778112	-1.903537	-3.757935

H	2.244552	-5.702137	-4.982676

H	-1.590037	-3.707166	-5.295399

H	-0.082252	-5.611534	-5.908221

C	0.544316	3.764361	-1.075964

C	-0.780881	3.705847	-1.541398

C	1.086967	4.959715	-0.580206

C	-1.568192	4.866649	-1.504673

C	0.287782	6.114092	-0.552018

C	-1.037783	6.072976	-1.014176

H	-1.185495	2.752511	-1.893874

H	2.119090	4.968081	-0.215174

H	-2.611850	4.814923	-1.831731

H	0.707650	7.049505	-0.164771

H	-1.659749	6.974151	-0.983858

C	4.875151	0.206193	-1.066913

C	5.741758	-0.749622	-1.621941

C	5.034346	1.575331	-1.346223

C	6.783344	-0.327794	-2.462317

C	6.085686	1.981298	-2.181906

C	6.963408	1.037120	-2.741550

H	5.587186	-1.808170	-1.389320

H	4.334387	2.301143	-0.921093

H	7.460626	-1.072615	-2.895566

H	6.211013	3.047103	-2.404620

H	7.780279	1.363203	-3.394330

C	-0.180527	1.104603	3.890147

C	-0.980155	2.172683	4.323644

C	0.084165	0.003050	4.723299

C	-1.521379	2.138524	5.619427

C	-0.458329	-0.011708	6.017097

C	-1.258663	1.052316	6.469985

H	-1.175723	3.005351	3.640650

H	0.684212	-0.829946	4.340752

H	-2.150688	2.967486	5.962577

H	-0.265598	-0.869630	6.671731

H	-1.683299	1.029477	7.479666

**1-cycle6-C**

SCF Done: -3617.19887233 A.U.

Ni	0.201273	-2.458369	0.059802

P	0.866728	-0.212762	0.006363

O	-1.521889	-1.701356	-0.103728

O	-0.246393	-0.723960	2.614598

O	3.748779	0.982975	0.647370

O	2.024653	-1.204539	-2.601618

O	1.367134	2.788710	-0.055245

N	-0.692805	0.521677	-0.001213

C	-1.645103	-0.393393	-0.089618

C	-3.063801	0.093512	-0.208681

C	-4.117502	-0.829654	-0.351464

C	-5.437173	-0.375638	-0.497491

C	-5.722158	0.999905	-0.505508

C	-4.671177	1.917940	-0.353785

C	-3.348954	1.471242	-0.200108

C	1.678339	0.219490	1.616339

C	0.959502	-0.083389	2.805574

C	1.500522	0.133656	4.084757

H	0.911780	-0.119815	4.970631

C	2.796553	0.653142	4.199699

H	3.224292	0.831112	5.192434

C	3.556507	0.924618	3.054708

H	4.574740	1.318636	3.127478

C	3.003488	0.682905	1.785466

C	1.690429	0.788472	-1.308811

C	2.159387	0.178782	-2.493551

C	2.644850	0.932715	-3.577900

H	2.984701	0.423644	-4.484326

C	2.659676	2.329931	-3.482620

H	3.029681	2.924948	-4.325078

C	2.202017	2.976775	-2.326056

H	2.219050	4.067336	-2.240772

C	1.725706	2.204954	-1.252596

C	-0.584660	-4.135637	0.408971

O	-1.447288	-4.308809	1.254192

C	-0.017820	-5.349394	-0.373724

H	-0.855847	-5.749085	-0.973647

H	0.248492	-6.130114	0.364505

C	1.177551	-5.036024	-1.295812

H	1.601213	-5.961843	-1.721835

H	0.838111	-4.409721	-2.144706

C	2.246213	-4.240787	-0.574519

C	3.686841	-4.660680	-0.642197

H	4.326848	-3.959660	-0.086157

H	3.803909	-5.684894	-0.237349

H	4.012059	-4.696043	-1.700441

O	1.929582	-3.220710	0.087735

C	-4.938380	3.404208	-0.402359

C	-6.567181	-1.375310	-0.588628

H	-6.749576	1.351284	-0.629125

H	-2.523611	2.177857	-0.070457

H	-3.885051	-1.897586	-0.349466

F	-6.184824	-2.516749	-1.228195

F	-7.641578	-0.868446	-1.261670

F	-7.013170	-1.743908	0.648350

F	-6.266336	3.696762	-0.311181

F	-4.301267	4.066285	0.602875

F	-4.489582	3.953696	-1.576728

C	4.996717	0.413901	0.459080

C	5.438154	-0.753256	1.112924

C	5.812463	1.048235	-0.498686

C	6.708469	-1.268947	0.807350

H	4.798501	-1.242938	1.852755

C	7.075115	0.514657	-0.794945

H	5.438239	1.949285	-0.995109

C	7.533185	-0.643852	-0.142574

H	7.053783	-2.172691	1.323313

H	7.707671	1.016081	-1.536459

H	8.524736	-1.050993	-0.367937

C	-1.324226	-0.571723	3.487061

C	-2.185547	-1.677510	3.582172

C	-1.601344	0.631639	4.158668

C	-3.329173	-1.578110	4.388166

H	-1.960572	-2.579886	3.003202

C	-2.747649	0.707504	4.966298

H	-0.940134	1.495339	4.039765

C	-3.611094	-0.393277	5.090029

H	-4.006581	-2.436392	4.462576

H	-2.970244	1.643993	5.490684

H	-4.505054	-0.323571	5.719323

C	3.014511	-1.935094	-3.246611

C	4.383656	-1.742035	-2.979947

C	2.587631	-2.953541	-4.118109

C	5.322808	-2.584868	-3.593562

H	4.704111	-0.949922	-2.295424

C	3.539903	-3.794877	-4.716845

H	1.517486	-3.061809	-4.324251

C	4.909872	-3.614845	-4.458433

H	6.387547	-2.437265	-3.380631

H	3.207007	-4.584903	-5.399772

H	5.650983	-4.267297	-4.932720

C	0.632017	3.973014	-0.062242

C	-0.513283	4.122922	-0.863796

C	1.026108	4.977214	0.835375

C	-1.272107	5.298205	-0.755888

H	-0.809755	3.313108	-1.537248

C	0.258676	6.149367	0.929691

H	1.918471	4.822867	1.450353

C	-0.888559	6.314773	0.136383

H	-2.184899	5.400674	-1.351645

H	0.562137	6.936011	1.629966

H	-1.488483	7.227506	0.219441

**TS-2-Coor-E-C**

SCF Done: -3695.75693954 A.U.

Ni	4.182339	13.327985	5.467549

P	1.971038	14.326193	5.428188

O	3.348065	12.367838	6.956561

O	2.660682	15.454289	8.267130

O	2.274575	16.695576	3.687021

O	1.584425	13.249702	2.621338

O	-0.680682	15.765099	5.998795

N	1.258189	13.460272	6.735750

C	2.090677	12.577277	7.260554

C	1.543812	11.671422	8.333107

C	2.326202	10.608295	8.823460

C	1.812907	9.746023	9.804401

C	0.514777	9.931278	10.308409

C	-0.261274	10.995746	9.824155

C	0.248123	11.864728	8.845788

C	2.245476	16.078694	5.987228

C	2.487418	16.461030	7.329294

C	2.655171	17.809847	7.694898

H	2.819001	18.063053	8.746506

C	2.635434	18.801975	6.707381

H	2.778439	19.851422	6.988185

C	2.485200	18.458540	5.356813

H	2.519662	19.217995	4.570555

C	2.309386	17.107839	5.018050

C	0.465722	14.465172	4.341281

C	0.414527	13.847036	3.066682

C	-0.773489	13.783833	2.316211

H	-0.778804	13.280393	1.346345

C	-1.940257	14.362769	2.830505

H	-2.867052	14.317036	2.247885

C	-1.932191	15.005330	4.072582

H	-2.832744	15.478254	4.475099

C	-0.735947	15.049720	4.811771

C	5.886459	12.863491	6.155669

O	6.830730	12.344510	5.590608

C	6.010822	13.367964	7.616903

H	6.405051	12.519736	8.206754

H	5.024585	13.645989	8.016253

C	6.987422	14.551699	7.680914

H	7.192016	14.843311	8.730116

H	7.969260	14.267782	7.249618

C	6.467522	15.767338	6.914323

C	7.229963	17.073253	7.072874

H	7.117517	17.446884	8.108647

H	8.312266	16.921492	6.904279

H	6.840193	17.827669	6.373115

O	5.470950	15.689139	6.193492

C	-1.664258	11.223221	10.336994

C	2.642241	8.573708	10.275578

H	0.117863	9.261418	11.075058

H	-0.341982	12.707429	8.473972

H	3.335087	10.471744	8.425639

F	2.285104	8.167086	11.528338

F	3.972288	8.874127	10.305951

F	2.499640	7.493535	9.452021

F	-2.586756	11.103437	9.329145

F	-2.015766	10.333875	11.307494

F	-1.818457	12.474671	10.853683

C	4.583873	12.573491	3.626045

C	4.953597	13.924295	3.638152

H	5.340264	11.794117	3.764413

H	3.603554	12.269962	3.246569

H	4.277028	14.698665	3.262046

H	6.002991	14.208837	3.765039

C	1.690461	12.872996	1.278100

C	1.767764	13.845198	0.265944

C	1.815090	11.506571	0.984855

C	1.972376	13.431482	-1.059204

H	1.673179	14.905425	0.523533

C	2.028402	11.107866	-0.345327

H	1.745810	10.775166	1.796725

C	2.104892	12.066303	-1.368710

H	2.036968	14.183572	-1.853761

H	2.131912	10.042331	-0.578872

H	2.270689	11.751783	-2.404852

C	-1.741014	15.668674	6.896060

C	-2.181807	16.859693	7.496339

C	-2.291929	14.426912	7.260279

C	-3.185792	16.802550	8.477748

H	-1.732641	17.810077	7.189516

C	-3.292351	14.385603	8.242804

H	-1.917442	13.513310	6.788893

C	-3.744135	15.569572	8.852580

H	-3.533803	17.730658	8.945668

H	-3.696729	13.416192	8.550841

H	-4.523466	15.528602	9.621206

C	2.176082	15.619598	9.562036

C	2.992971	15.160612	10.608754

C	0.896022	16.140016	9.822027

C	2.524375	15.228701	11.929841

H	3.980230	14.749422	10.374437

C	0.444487	16.207669	11.148430

H	0.259232	16.465099	8.994050

C	1.252552	15.756803	12.206303

H	3.160397	14.866936	12.745847

H	-0.558299	16.601048	11.349493

H	0.889163	15.808062	13.238344

C	1.653485	17.494964	2.731569

C	2.304233	17.626953	1.492985

C	0.395636	18.083994	2.953775

C	1.685272	18.356894	0.465175

H	3.286745	17.163617	1.355524

C	-0.205213	18.816739	1.919315

H	-0.099338	17.956436	3.921218

C	0.432476	18.956770	0.674417

H	2.193332	18.464244	-0.500000

H	-1.187285	19.273410	2.087292

H	-0.045237	19.529580	-0.127723

**2-Coor-E-C**

SCF Done: -3695.76308611 A.U.

Ni	1.476707	-1.882729	-0.705617

P	1.142968	0.362098	-0.123284

O	-0.384187	-1.993553	-0.185176

O	2.939021	-0.771912	1.990056

O	0.592996	3.234221	0.849244

O	3.622594	1.990468	-0.605098

O	-0.525793	0.852058	-2.630879

N	-0.555641	0.343363	0.183216

C	-1.026208	-0.889573	0.136364

C	-2.468423	-1.091553	0.518128

C	-3.303552	0.014236	0.769899

C	-4.636573	-0.185009	1.161074

C	-5.156588	-1.482963	1.296391

C	-4.325845	-2.583927	1.035857

C	-2.989469	-2.391887	0.651313

C	1.704615	1.192729	1.432140

C	2.452963	0.463656	2.389506

C	2.726132	0.986581	3.666635

H	3.317228	0.402943	4.377383

C	2.235608	2.254067	4.006551

H	2.450430	2.666410	4.998608

C	1.494947	3.008960	3.088334

H	1.121125	4.005845	3.340093

C	1.242148	2.474551	1.813498

C	1.447874	1.572078	-1.483580

C	2.657345	2.307744	-1.554506

C	2.906807	3.240182	-2.573979

H	3.850946	3.791633	-2.586875

C	1.942516	3.428701	-3.574609

H	2.132003	4.147252	-4.379604

C	0.766863	2.669005	-3.585806

H	0.032499	2.770970	-4.390046

C	0.541733	1.731210	-2.561169

C	1.393910	-3.767962	-0.918296

C	0.366209	-4.338889	-1.895657

H	-0.622868	-4.113094	-1.456595

H	0.428244	-3.762547	-2.834633

C	0.549799	-5.839727	-2.145048

C	1.838931	-6.167669	-2.905518

C	3.508000	-1.712876	-1.077044

H	3.818839	-0.706086	-0.783540

H	3.967955	-2.546372	-0.532481

C	2.831725	-1.947320	-2.277953

H	2.832209	-2.937420	-2.750796

H	2.532048	-1.107019	-2.918593

C	2.213495	-7.641221	-3.002167

H	1.341134	-8.267076	-3.265301

H	3.017867	-7.780296	-3.740631

H	2.570933	-7.988482	-2.013537

H	-0.299532	-6.235747	-2.738995

H	0.542648	-6.404034	-1.193510

O	2.096668	-4.450070	-0.193351

O	2.537336	-5.291391	-3.412366

C	-4.886055	-3.985684	1.110353

C	-5.495369	1.010571	1.503787

F	-5.948645	-4.066703	1.961096

F	-3.950400	-4.882786	1.533990

F	-5.320868	-4.416289	-0.111496

F	-5.266083	2.060112	0.669408

F	-5.240501	1.457096	2.776536

F	-6.826200	0.721148	1.449402

H	-2.332521	-3.245306	0.466314

H	-2.892808	1.021174	0.651313

H	-6.195265	-1.633666	1.601590

C	4.516676	2.970501	-0.170557

C	4.073454	4.233072	0.260719

C	5.872458	2.614461	-0.100947

C	5.012744	5.149503	0.756899

C	6.798578	3.539434	0.406496

C	6.374546	4.809139	0.831850

H	3.007409	4.476288	0.215592

H	6.182908	1.621793	-0.442454

H	4.673159	6.134199	1.097953

H	7.857982	3.264864	0.462786

H	7.100879	5.529463	1.223564

C	-1.777678	1.321015	-3.023879

C	-2.313384	2.520216	-2.521784

C	-2.522275	0.498870	-3.884980

C	-3.609592	2.897421	-2.904315

C	-3.820057	0.887360	-4.252045

C	-4.366490	2.087905	-3.768832

H	-1.727638	3.132833	-1.829580

H	-2.076574	-0.431299	-4.252127

H	-4.034107	3.826642	-2.507997

H	-4.403646	0.247095	-4.923330

H	-5.379319	2.388239	-4.058367

C	3.198687	-1.750491	2.954203

C	2.192854	-2.198336	3.828391

C	4.473172	-2.336482	2.954027

C	2.480239	-3.245041	4.717084

C	4.743683	-3.389280	3.843282

C	3.752486	-3.843314	4.728246

H	1.201514	-1.734938	3.800990

H	5.231619	-1.966285	2.257035

H	1.698421	-3.602567	5.396708

H	5.736358	-3.853315	3.842679

H	3.967242	-4.664528	5.420563

C	-0.580718	3.905686	1.186216

C	-1.575975	3.313150	1.983781

C	-0.770358	5.176393	0.618322

C	-2.771918	4.010445	2.212640

C	-1.972653	5.861894	0.857028

C	-2.974268	5.285227	1.655042

H	-1.415575	2.310021	2.389261

H	0.020442	5.607661	-0.004232

H	-3.560780	3.536263	2.805485

H	-2.121605	6.854432	0.416580

H	-3.912117	5.821446	1.835695

**TS-2-Ins-E-C**

SCF Done: -3695.75574198 A.U.

Ni	-1.079782	-1.996852	0.182080

P	0.873605	-1.069630	0.009842

O	-1.720956	-0.128782	0.059372

N	0.522312	0.602702	0.159691

C	-0.792040	0.788917	0.121065

C	-1.286812	2.209370	0.143456

C	-2.662828	2.471934	0.015343

C	-3.139665	3.791719	0.062130

C	-2.248480	4.865873	0.207607

C	-0.871861	4.603747	0.314381

C	-0.390913	3.285596	0.294473

C	2.089641	-1.510876	1.318579

C	3.211635	-2.356340	1.119268

C	4.120154	-2.628870	2.156470

H	4.995842	-3.252859	1.958804

C	3.886208	-2.097997	3.432828

H	4.590867	-2.314001	4.243526

C	2.751869	-1.320297	3.690438

H	2.537100	-0.939261	4.692580

C	1.858380	-1.047676	2.638609

C	1.725243	-1.082595	-1.625751

C	2.849810	-0.246756	-1.837978

C	3.390971	-0.039731	-3.117613

H	4.258583	0.615360	-3.238841

C	2.808176	-0.691933	-4.212554

H	3.226439	-0.545609	-5.214657

C	1.711221	-1.546543	-4.043728

H	1.269494	-2.069666	-4.895962

C	1.176699	-1.736928	-2.755592

C	-3.055767	-2.448138	0.608906

C	-3.225657	-1.992594	2.064510

H	-3.666470	-2.830297	2.642955

H	-2.234193	-1.802233	2.516143

C	-4.116973	-0.751067	2.160575

C	-4.305526	-0.226915	3.579788

C	-1.994082	-3.896174	0.399658

H	-2.422765	-4.387538	1.290462

H	-2.409704	-4.296329	-0.534716

C	-0.515955	-3.789728	0.439055

H	0.036818	-4.181816	-0.423384

H	-0.014355	-3.939921	1.406301

C	-5.327680	0.892882	3.746309

H	-5.180819	1.686926	2.991323

H	-6.348196	0.492619	3.590320

H	-5.260897	1.317393	4.759809

H	-5.101830	-0.935375	1.690850

H	-3.655816	0.058605	1.561032

O	-3.909988	-2.303697	-0.254057

O	-3.664728	-0.667718	4.534776

C	0.109379	5.751095	0.390630

C	-4.629988	4.034367	0.022898

F	0.619904	6.050090	-0.847168

F	1.169750	5.463613	1.192579

F	-0.469560	6.891638	0.861647

F	-4.936924	5.318244	-0.310818

F	-5.203705	3.794694	1.246272

F	-5.254234	3.213898	-0.866933

H	0.676747	3.071897	0.399175

H	-3.348104	1.632174	-0.129576

H	-2.620187	5.893214	0.238157

O	3.397071	-2.843352	-0.162943

O	0.652259	-0.413320	2.868981

O	0.120154	-2.605693	-2.532454

O	3.443453	0.292844	-0.712649

C	4.116173	-4.015673	-0.387037

C	3.868241	-5.198266	0.333763

C	5.035302	-3.996021	-1.448449

C	4.564661	-6.366233	-0.012774

C	5.717154	-5.174728	-1.788412

C	5.489573	-6.360811	-1.071050

H	3.140446	-5.198232	1.151183

H	5.192520	-3.061705	-1.996304

H	4.374029	-7.289585	0.546093

H	6.433380	-5.161505	-2.617679

H	6.026982	-7.277560	-1.336622

C	0.593512	0.652043	3.772940

C	-0.512950	0.694894	4.634827

C	1.552763	1.679806	3.760401

C	-0.651550	1.786840	5.506727

C	1.401912	2.758399	4.645220

C	0.304020	2.816335	5.521137

H	-1.257857	-0.107263	4.611935

H	2.390583	1.636123	3.057764

H	-1.516287	1.823599	6.178576

H	2.143083	3.565736	4.636916

H	0.191279	3.664012	6.205867

C	3.897423	1.610974	-0.735499

C	5.140574	1.866880	-0.136150

C	3.107681	2.655945	-1.246363

C	5.598389	3.191552	-0.049347

C	3.577269	3.974760	-1.149483

C	4.822002	4.247190	-0.554982

H	5.724630	1.029643	0.259257

H	2.130660	2.429205	-1.682971

H	6.568619	3.395684	0.417749

H	2.948074	4.794308	-1.512250

H	5.179784	5.279664	-0.478758

C	-0.763748	-2.888552	-3.580596

C	-0.889394	-4.225289	-3.987160

C	-1.568929	-1.878788	-4.134331

C	-1.841210	-4.555685	-4.966452

C	-2.510024	-2.222581	-5.116272

C	-2.649511	-3.557767	-5.534549

H	-0.245801	-4.986523	-3.534250

H	-1.463999	-0.847109	-3.784278

H	-1.946616	-5.598576	-5.285958

H	-3.148117	-1.442271	-5.545859

H	-3.391401	-3.819300	-6.296759

**2-cycle5-T**

SCF Done: -3695.7924402 A.U.

Ni	0.154763	-2.154007	0.396085

P	1.057929	-0.250306	0.073419

O	-1.518407	-1.187180	0.110008

O	2.161207	2.538713	0.031403

O	1.447632	-1.123166	3.010786

O	0.597701	-0.585900	-2.831021

O	4.130023	-0.066969	0.318150

N	-0.258250	0.738345	-0.409279

C	-1.392285	0.055648	-0.264400

C	-2.676451	0.790171	-0.535552

C	-3.907620	0.139135	-0.332879

C	-5.113759	0.819469	-0.562214

C	-5.107108	2.156462	-0.991394

C	-3.877719	2.803676	-1.198078

C	-2.667752	2.126837	-0.977709

C	1.690966	0.728172	1.509406

C	2.017745	2.096531	1.333154

C	2.268897	2.948742	2.421348

H	2.516018	3.998803	2.239841

C	2.209224	2.426746	3.720502

H	2.410713	3.080111	4.576711

C	1.921318	1.073572	3.938134

H	1.899639	0.657831	4.949327

C	1.668675	0.235699	2.835539

C	2.338252	-0.189749	-1.251935

C	1.927681	-0.285345	-2.605156

C	2.843296	-0.203109	-3.669863

H	2.480795	-0.276730	-4.698994

C	4.205678	-0.058935	-3.384859

H	4.927889	0.007453	-4.206152

C	4.662301	-0.026615	-2.059801

H	5.728091	0.065219	-1.833546

C	3.733498	-0.117153	-1.008852

C	1.779892	-3.180730	0.507930

H	2.672612	-2.669535	0.901539

H	1.966366	-3.470422	-0.546371

C	1.420615	-4.416925	1.361027

H	1.497747	-4.161448	2.440371

H	2.077310	-5.301449	1.213117

C	-0.022999	-4.762981	1.125967

C	-0.586504	-6.139004	1.400301

H	-0.356886	-6.404869	2.451397

O	-0.775116	-3.850006	0.702378

C	-3.848156	4.258178	-1.605773

C	-6.425710	0.122485	-0.286167

H	-6.047070	2.682506	-1.176716

H	-1.705524	2.617767	-1.150061

H	-3.899273	-0.902431	-0.000871

F	-6.371323	-1.203725	-0.600338

F	-6.766718	0.199932	1.035321

F	-7.455443	0.669848	-0.993697

F	-2.847071	4.521304	-2.489464

F	-5.019404	4.658384	-2.177062

F	-3.639152	5.074897	-0.522242

C	-2.083166	-6.254345	1.097066

H	-2.656279	-5.443417	1.580944

H	-2.477766	-7.206371	1.507449

C	-2.366442	-6.262600	-0.415431

C	-3.776323	-5.885929	-0.841071

H	-4.538051	-6.367189	-0.200186

H	-3.900543	-4.792256	-0.722464

H	-3.941241	-6.152324	-1.896257

H	-0.024149	-6.856046	0.770098

O	-1.493150	-6.565819	-1.224051

C	1.681279	3.796253	-0.329146

C	0.403680	4.239701	0.055551

C	2.492541	4.568151	-1.175865

C	-0.060942	5.475290	-0.420297

H	-0.217428	3.604288	0.693523

C	2.014710	5.803693	-1.641908

H	3.478710	4.188194	-1.461466

C	0.741176	6.261626	-1.266092

H	-1.069911	5.805511	-0.152326

H	2.645359	6.409436	-2.302716

H	0.369762	7.222955	-1.637296

C	0.817324	-1.562473	4.174123

C	-0.470957	-1.115303	4.518694

C	1.472183	-2.541724	4.939055

C	-1.102124	-1.660207	5.647001

H	-0.966889	-0.361589	3.899055

C	0.824193	-3.084015	6.061849

H	2.479887	-2.858314	4.649787

C	-0.460593	-2.644362	6.420456

H	-2.108223	-1.319245	5.916322

H	1.333196	-3.846740	6.662164

H	-0.961790	-3.066434	7.298273

C	5.384775	-0.531133	0.704434

C	6.084238	0.251902	1.637248

C	5.894297	-1.769862	0.273035

C	7.310383	-0.210069	2.140112

H	5.651633	1.204079	1.959847

C	7.125772	-2.213354	0.779053

H	5.329838	-2.372333	-0.445375

C	7.838342	-1.439064	1.710858

H	7.856582	0.399532	2.868841

H	7.525702	-3.177840	0.445523

H	8.798135	-1.793720	2.101891

C	-0.071471	-0.000936	-3.904733

C	0.047957	1.369403	-4.195747

C	-0.941132	-0.826127	-4.635245

C	-0.708362	1.907816	-5.247834

H	0.709213	1.998915	-3.592639

C	-1.696440	-0.269585	-5.679143

H	-1.015969	-1.886443	-4.373219

C	-1.579686	1.094770	-5.993115

H	-0.625035	2.976820	-5.474480

H	-2.377288	-0.910316	-6.250971

H	-2.169966	1.524706	-6.809610

**TS-Isom-CO-T**

SCF Done: -3617.18815082 A.U.

Ni	-3.139454	2.716091	5.046498

P	-3.042731	0.487967	5.204403

O	-1.066677	2.334901	5.708015

O	-3.708043	2.518706	7.250254

O	-3.415369	-2.214010	6.656305

O	-5.901450	-0.103524	5.365922

O	-2.068501	-1.123297	2.724764

N	-1.397297	0.053199	5.211528

C	-0.656747	1.119134	5.545104

C	0.815054	0.860975	5.760903

C	1.668655	1.936353	6.069242

C	3.032845	1.709822	6.313250

C	3.556412	0.408174	6.269639

C	2.703156	-0.665532	5.963361

C	1.342733	-0.443742	5.698330

C	-3.515932	0.156752	6.959958

C	-3.672193	1.249084	7.840171

C	-3.798264	1.074316	9.227526

H	-3.912927	1.939291	9.884981

C	-3.771913	-0.227959	9.749001

H	-3.871173	-0.373755	10.830228

C	-3.627927	-1.340334	8.910655

H	-3.610010	-2.358094	9.311624

C	-3.499334	-1.138947	7.525295

C	-3.956429	-0.696029	4.143614

C	-5.356614	-0.852735	4.338322

C	-6.118850	-1.752347	3.577300

H	-7.187241	-1.866576	3.777790

C	-5.487506	-2.491890	2.566762

H	-6.075600	-3.193392	1.964693

C	-4.125390	-2.323802	2.299036

H	-3.635800	-2.864644	1.484331

C	-3.373237	-1.417403	3.071409

C	-3.508061	2.563750	3.091662

H	-4.034181	3.473958	2.765968

C	-3.550289	4.452924	4.958264

O	-3.949342	5.544570	4.940865

H	0.673364	-1.271004	5.444221

H	4.615671	0.231655	6.473992

H	1.244313	2.942991	6.122964

C	3.236998	-2.079035	5.996084

C	3.949382	2.883759	6.573955

F	2.622734	-2.880289	5.086378

F	3.040251	-2.654621	7.227741

F	4.577729	-2.126964	5.754546

F	4.376364	3.452497	5.408116

F	3.324389	3.863403	7.286663

F	5.063733	2.516386	7.269320

C	-2.158290	2.431878	2.399976

H	-2.323358	2.233257	1.312568

H	-1.581509	1.556988	2.755335

C	-1.281761	3.688350	2.449455

C	0.183641	3.499600	2.078748

H	0.303409	2.827187	1.209620

H	0.656214	4.473308	1.876915

H	0.702310	3.020205	2.931241

H	-4.153076	1.688669	2.920123

O	-1.733845	4.788822	2.757934

C	-3.500291	3.643586	8.086704

C	-4.616300	4.323158	8.591374

C	-2.189233	4.081397	8.317108

C	-4.405980	5.477733	9.364733

H	-5.623237	3.954176	8.371706

C	-1.997825	5.237091	9.091290

H	-1.357043	3.535032	7.861599

C	-3.100816	5.932361	9.617336

H	-5.267351	6.025047	9.763326

H	-0.980483	5.600404	9.274115

H	-2.943268	6.835914	10.216267

C	-2.520587	-3.245731	6.943970

C	-2.964173	-4.558401	6.716290

C	-1.199046	-2.984460	7.347018

C	-2.067196	-5.624138	6.898752

H	-3.997980	-4.725369	6.397062

C	-0.314669	-4.059055	7.523696

H	-0.870921	-1.950110	7.486199

C	-0.744293	-5.379844	7.303478

H	-2.409796	-6.650580	6.724738

H	0.724493	-3.853968	7.801836

H	-0.047494	-6.213871	7.440313

C	-1.212111	-2.153073	2.338437

C	-0.366091	-1.900382	1.246710

C	-1.134550	-3.361840	3.052089

C	0.568291	-2.874601	0.862637

H	-0.449837	-0.946588	0.716011

C	-0.199620	-4.328151	2.650873

H	-1.784885	-3.531282	3.915780

C	0.652296	-4.092049	1.558315

H	1.230341	-2.679302	0.011410

H	-0.130090	-5.267141	3.211196

H	1.382519	-4.850219	1.255536

C	-7.280610	0.114878	5.421994

C	-7.949729	0.799495	4.392709

C	-7.952088	-0.282713	6.587727

C	-9.317336	1.077535	4.538327

H	-7.399410	1.111137	3.499229

C	-9.319105	0.009743	6.722721

H	-7.395243	-0.805087	7.371852

C	-10.005118	0.685087	5.700043

H	-9.844872	1.614078	3.741651

H	-9.848304	-0.295795	7.632314

H	-11.071674	0.909791	5.808600

**1-Coor-CO-C**

SCF Done: -3617.20619428 A.U.

Ni	0.335200	2.506182	0.013374

P	-0.937771	0.633276	-0.049433

O	1.769593	1.249986	-0.125737

O	-1.123983	1.966699	2.663113

O	-2.504958	-1.953964	0.304293

O	-3.826434	1.376352	0.054724

O	-0.452491	0.003428	-2.990699

N	0.267391	-0.569093	-0.294020

C	1.471531	-0.026614	-0.250635

C	2.654175	-0.951730	-0.322172

C	2.469076	-2.334465	-0.514306

C	3.577529	-3.194273	-0.546921

C	4.879635	-2.687817	-0.400585

C	5.062798	-1.307672	-0.221043

C	3.958163	-0.442376	-0.178921

C	-1.735089	-0.017771	1.483470

C	-1.624841	0.676339	2.713797

C	-2.054643	0.100907	3.923557

H	-1.968878	0.668983	4.853722

C	-2.591260	-1.192932	3.915555

H	-2.930876	-1.641086	4.855786

C	-2.722457	-1.910147	2.719501

H	-3.155747	-2.914472	2.701377

C	-2.301558	-1.315077	1.518691

C	-2.173603	0.489403	-1.402399

C	-3.531516	0.831601	-1.185372

C	-4.500002	0.704012	-2.193713

H	-5.538780	0.975568	-1.987844

C	-4.104699	0.254384	-3.461549

H	-4.851239	0.163165	-4.258219

C	-2.761738	-0.027761	-3.737532

H	-2.439092	-0.328135	-4.738377

C	-1.803287	0.118238	-2.718170

H	4.091955	0.632948	-0.036198

H	5.741324	-3.359078	-0.440006

H	1.452305	-2.717003	-0.643262

C	6.452890	-0.751402	-0.010397

C	3.355048	-4.683327	-0.682737

F	7.410655	-1.550592	-0.561017

F	6.747495	-0.632544	1.317841

F	6.587950	0.490038	-0.557803

F	4.474436	-5.334835	-1.105965

F	3.002178	-5.243069	0.519637

F	2.352804	-4.971818	-1.555942

C	1.692726	3.960468	0.036132

H	2.334856	3.565567	0.842346

C	2.408529	3.971635	-1.307675

H	1.315400	4.958171	0.311495

H	2.836353	2.968598	-1.515685

H	3.282657	4.661516	-1.293657

C	1.544018	4.337021	-2.515636

C	2.296478	4.706440	-3.791997

O	0.313971	4.331719	-2.483917

H	3.126478	4.002621	-3.987176

H	1.604932	4.726082	-4.648366

H	2.750067	5.709698	-3.675127

C	-0.871416	3.738122	0.083951

O	-1.690381	4.562551	0.154092

C	-0.444925	2.494472	3.764381

C	0.614768	1.805494	4.380907

C	-0.799771	3.790854	4.168301

C	1.315799	2.429394	5.424165

H	0.884646	0.800555	4.041228

C	-0.082510	4.404979	5.207491

H	-1.623966	4.301170	3.660257

C	0.971923	3.727091	5.841191

H	2.145125	1.898714	5.905454

H	-0.354931	5.418280	5.523346

H	1.527537	4.208850	6.652902

C	-2.151870	-3.296368	0.170501

C	-3.004209	-4.102601	-0.601410

C	-0.954394	-3.807734	0.701489

C	-2.650535	-5.440463	-0.841240

H	-3.926063	-3.672526	-1.006381

C	-0.614185	-5.146011	0.451740

H	-0.294307	-3.150966	1.275408

C	-1.459175	-5.966866	-0.315697

H	-3.315054	-6.072349	-1.441507

H	0.334593	-5.535954	0.834675

H	-1.184708	-7.009401	-0.509178

C	-5.101435	1.221021	0.600672

C	-5.707941	2.365784	1.140130

C	-5.716420	-0.040042	0.689412

C	-6.953833	2.244110	1.775434

H	-5.196309	3.329738	1.056056

C	-6.965716	-0.143150	1.319686

H	-5.210125	-0.919225	0.278815

C	-7.588337	0.993871	1.863230

H	-7.431620	3.135038	2.198314

H	-7.449998	-1.123517	1.394827

H	-8.562394	0.904406	2.356325

C	-0.008113	-1.002002	-3.847520

C	-0.452479	-2.330412	-3.724722

C	0.970857	-0.649023	-4.789963

C	0.090372	-3.308960	-4.571077

H	-1.196863	-2.589309	-2.965401

C	1.509238	-1.641469	-5.623915

H	1.295598	0.394330	-4.855328

C	1.069116	-2.971550	-5.521886

H	-0.246629	-4.347054	-4.473169

H	2.273933	-1.369125	-6.360251

H	1.490672	-3.743021	-6.175334

**TS-1-Ins-CO-C**

SCF Done: -3617.18980709 A.U.

Ni	0.932688	-1.646463	1.356565

P	-0.693969	-0.569729	0.396398

O	2.048412	-0.219088	0.653948

N	0.124742	0.755892	-0.306614

C	1.421951	0.705057	-0.029584

C	2.269028	1.840531	-0.532086

C	3.633521	1.907014	-0.193253

C	4.419522	2.980680	-0.639053

C	3.854863	4.000255	-1.423590

C	2.495342	3.928423	-1.766026

C	1.704951	2.853474	-1.329479

C	-1.466933	-1.628798	-0.898304

C	-2.446747	-2.564177	-0.483938

C	-3.033284	-3.483921	-1.367808

H	-3.780481	-4.193961	-1.003688

C	-2.620431	-3.481533	-2.706891

H	-3.067245	-4.192210	-3.411185

C	-1.634165	-2.595069	-3.155362

H	-1.308514	-2.597529	-4.199040

C	-1.051260	-1.683665	-2.253300

C	-2.073898	0.285726	1.267175

C	-3.027865	1.024200	0.527101

C	-4.007192	1.813354	1.154410

H	-4.729767	2.361586	0.542773

C	-4.034863	1.873668	2.552671

H	-4.791619	2.487071	3.054233

C	-3.103692	1.165156	3.323755

H	-3.115544	1.229197	4.414877

C	-2.128371	0.380412	2.681114

C	2.296733	-2.842612	2.535742

H	2.903411	-1.966740	2.234326

C	0.419971	-2.952496	2.339664

O	-0.149308	-3.827922	2.907582

C	2.830500	-4.132118	1.916956

H	2.266001	-5.008021	2.301404

H	3.885630	-4.316700	2.207790

C	2.730863	-4.172322	0.391868

C	3.478035	-5.290050	-0.317897

H	3.333224	-6.258291	0.195033

H	3.150723	-5.364040	-1.366034

H	4.563859	-5.074461	-0.296303

H	2.281767	-2.877352	3.634937

O	2.072698	-3.339014	-0.235124

H	0.646546	2.781104	-1.598193

H	4.466186	4.840688	-1.761717

H	4.060814	1.114857	0.426963

C	1.845876	5.027180	-2.575305

C	5.896894	3.009645	-0.321258

F	1.110458	4.525852	-3.606730

F	2.757084	5.892596	-3.101927

F	0.986004	5.769606	-1.808116

F	6.623146	2.318147	-1.248934

F	6.388903	4.282074	-0.301350

F	6.166026	2.446359	0.890971

O	-0.095647	-0.778041	-2.661140

O	-2.706675	-2.574898	0.878303

O	-1.123031	-0.256867	3.384203

O	-3.019773	0.881896	-0.851827

C	-3.070701	2.026949	-1.646971

C	-3.858002	1.961717	-2.807181

C	-2.301379	3.167016	-1.354285

C	-3.873330	3.056625	-3.686232

C	-2.325246	4.252043	-2.243732

C	-3.111019	4.203287	-3.408768

H	-4.437945	1.055351	-3.008764

H	-1.673823	3.178751	-0.458328

H	-4.487262	3.009299	-4.593019

H	-1.699149	5.126383	-2.036374

H	-3.121343	5.052578	-4.100501

C	-1.285512	-0.530122	4.743232

C	-2.329534	-1.352021	5.202881

C	-0.308914	-0.036084	5.621998

C	-2.393002	-1.669025	6.568330

C	-0.380797	-0.370340	6.984228

C	-1.422922	-1.181930	7.461813

H	-3.067448	-1.742948	4.494841

H	0.491653	0.598074	5.227682

H	-3.202331	-2.311831	6.932986

H	0.380576	0.011471	7.673823

H	-1.477267	-1.438345	8.525440

C	0.683526	-1.071714	-3.782142

C	0.591929	-0.216853	-4.890753

C	1.602876	-2.135071	-3.751452

C	1.434330	-0.435653	-5.993383

C	2.432126	-2.344046	-4.863951

C	2.350317	-1.500233	-5.985956

H	-0.125676	0.609389	-4.869995

H	1.675378	-2.760582	-2.854925

H	1.371396	0.231118	-6.860797

H	3.157676	-3.165660	-4.848266

H	3.005644	-1.667315	-6.847949

C	-3.929323	-3.023296	1.369844

C	-3.882677	-3.877172	2.484526

C	-5.154838	-2.574896	0.845910

C	-5.086183	-4.291138	3.077872

C	-6.347867	-3.005417	1.446458

C	-6.320225	-3.862405	2.560380

H	-2.906439	-4.196928	2.862978

H	-5.164241	-1.895397	-0.012030

H	-5.055850	-4.960001	3.945660

H	-7.306474	-2.657953	1.044334

H	-7.256526	-4.191748	3.023866

**1-cycle6-T**

SCF Done: -3617.21239165 A.U.

Ni	0.930919	2.040327	1.060710

P	0.883779	0.014358	0.329021

O	-0.991838	2.020025	0.540120

O	1.955170	2.094409	-1.533204

O	1.263130	-2.632561	-1.191159

O	3.612744	-1.262646	0.504467

O	-0.582144	-1.100078	2.716372

N	-0.766632	-0.260608	-0.016405

C	-1.447667	0.876596	0.114438

C	-2.896676	0.847640	-0.291190

C	-3.483505	-0.334718	-0.780087

C	-4.823765	-0.334992	-1.197551

C	-5.596713	0.834872	-1.119125

C	-5.013178	2.010042	-0.617843

C	-3.669927	2.020681	-0.210360

C	1.645085	-0.262933	-1.328754

C	2.089457	0.841211	-2.101776

C	2.560557	0.669599	-3.416851

H	2.899893	1.537765	-3.987704

C	2.563200	-0.610242	-3.986821

H	2.926192	-0.741535	-5.012192

C	2.100294	-1.718561	-3.266463

H	2.092955	-2.722786	-3.700589

C	1.644679	-1.530874	-1.951988

C	1.453460	-1.314440	1.468210

C	2.767373	-1.852844	1.419558

C	3.179262	-2.874377	2.291359

H	4.196340	-3.269074	2.224775

C	2.277628	-3.361857	3.249713

H	2.597329	-4.155367	3.934422

C	0.995470	-2.816171	3.371915

H	0.301311	-3.154674	4.146518

C	0.606412	-1.781019	2.501248

C	2.671620	1.984003	1.717239

O	3.723570	1.867407	1.099725

C	2.730733	2.229534	3.255941

H	3.600905	2.881737	3.460416

H	2.943448	1.247809	3.717998

C	1.444076	2.812435	3.888469

H	0.653021	2.035329	3.881867

H	1.621682	3.105857	4.938088

C	0.904041	3.975834	3.076614

C	0.620951	5.299630	3.736318

H	1.549634	5.702065	4.185131

H	0.212946	6.020370	3.012056

H	-0.093539	5.158130	4.569880

O	0.698534	3.829739	1.853239

C	-5.851186	3.257872	-0.462475

C	-5.407091	-1.595318	-1.792450

C	-1.753074	-1.826277	2.925699

C	0.223658	-3.440387	-1.647824

C	2.644688	3.177190	-2.092004

C	4.839199	-1.844309	0.183215

H	-6.639363	0.831500	-1.446808

H	-3.199152	2.933781	0.163989

H	-2.874973	-1.242849	-0.827113

F	-6.768910	-1.562566	-1.842317

F	-5.047001	-2.704094	-1.089150

F	-4.968321	-1.789495	-3.078235

F	-6.386578	3.349542	0.791839

F	-5.118008	4.389864	-0.661412

F	-6.895113	3.285099	-1.340834

C	-2.658775	-1.310878	3.866997

C	-3.884564	-1.963644	4.069206

C	-4.200519	-3.123697	3.342783

C	-3.285184	-3.623621	2.400586

C	-2.058552	-2.978868	2.180779

H	-2.393574	-0.405983	4.423503

H	-4.593609	-1.561889	4.802043

H	-5.157950	-3.630981	3.503465

H	-3.531041	-4.516440	1.814850

H	-1.353670	-3.346581	1.429047

C	4.905913	-3.097992	-0.447554

C	6.160422	-3.598431	-0.829108

C	7.327485	-2.850703	-0.593896

C	7.238809	-1.591863	0.024020

C	5.992245	-1.080772	0.419337

H	3.982538	-3.652309	-0.642944

H	6.223292	-4.574295	-1.324076

H	8.302774	-3.245851	-0.898736

H	8.144877	-1.001376	0.201424

H	5.886333	-0.099260	0.892277

C	1.903630	4.168430	-2.752281

C	2.571234	5.302093	-3.245517

C	3.960253	5.433755	-3.082090

C	4.684990	4.432123	-2.411951

C	4.031584	3.297198	-1.907245

H	0.821718	4.045007	-2.864433

H	2.000343	6.083312	-3.760066

H	4.476707	6.319087	-3.469295

H	5.766470	4.539012	-2.269258

H	4.563496	2.522052	-1.348828

C	0.275602	-4.795232	-1.278241

C	-0.777280	-5.648224	-1.644883

C	-1.872658	-5.155578	-2.373570

C	-1.913118	-3.796955	-2.731916

C	-0.867697	-2.931251	-2.374331

H	1.134367	-5.159567	-0.704905

H	-0.735814	-6.705509	-1.358646

H	-2.694274	-5.823323	-2.654270

H	-2.779055	-3.393745	-3.267682

H	-0.903778	-1.867439	-2.625064

**TS-2-Coor-E-T**

SCF Done: -3695.76511102 A.U.

Ni	1.920679	-1.435648	0.065804

P	0.770986	0.318537	0.645142

O	0.193697	-1.915592	-0.862614

N	-0.842733	-0.118532	0.261868

C	-0.838620	-1.199524	-0.514133

C	-2.166963	-1.637944	-1.073700

C	-2.222578	-2.728075	-1.961807

C	-3.448962	-3.125009	-2.518310

C	-4.631023	-2.437243	-2.200561

C	-4.575146	-1.353294	-1.308613

C	-3.353911	-0.957519	-0.740768

C	0.841127	0.901361	2.384329

C	-0.024216	0.415195	3.394738

C	0.054500	0.883167	4.720254

H	-0.632989	0.484635	5.471416

C	1.031361	1.825724	5.057786

H	1.099325	2.189156	6.089278

C	1.943463	2.291920	4.100551

H	2.720292	3.013812	4.365305

C	1.852540	1.811550	2.785060

C	0.907766	1.828228	-0.429968

C	1.674649	1.831543	-1.619827

C	1.555036	2.854058	-2.577908

H	2.158427	2.815194	-3.489077

C	0.667539	3.912420	-2.345863

H	0.576954	4.714751	-3.086467

C	-0.091110	3.963559	-1.170296

H	-0.777596	4.791611	-0.970657

C	0.030600	2.924177	-0.232059

C	3.460491	-1.003846	1.006043

O	3.571402	-0.938839	2.226801

C	4.708370	-0.901614	0.098717

H	5.532284	-0.455803	0.684862

H	4.478414	-0.243560	-0.755654

C	5.137700	-2.292054	-0.409050

H	6.041750	-2.217925	-1.043091

H	5.428857	-2.937322	0.448400

C	4.063874	-3.047603	-1.173158

C	4.499847	-4.137714	-2.124846

H	5.276177	-4.776761	-1.664712

H	3.637928	-4.748162	-2.433565

H	4.954595	-3.677510	-3.023802

O	2.849249	-2.804544	-1.037189

C	-5.824022	-0.557701	-1.007591

C	-3.500469	-4.335732	-3.421205

H	-5.583571	-2.741700	-2.641697

H	-3.301454	-0.123324	-0.034834

H	-1.293346	-3.248225	-2.210079

F	-4.577470	-4.304353	-4.258212

F	-3.588437	-5.494523	-2.703549

F	-2.382251	-4.436418	-4.196373

F	-6.960747	-1.266573	-1.259738

F	-5.869777	-0.149331	0.288655

F	-5.888659	0.576911	-1.779311

C	1.109638	-3.180582	2.096703

C	1.671946	-4.329986	1.673625

H	1.629110	-2.503080	2.783074

H	0.091365	-2.901155	1.808093

H	1.150758	-5.011707	0.991194

H	2.682535	-4.623924	1.984939

O	-0.667003	2.970402	0.962878

O	2.587928	0.797054	-1.799722

O	-0.879442	-0.617950	3.058865

O	2.703664	2.242838	1.779718

C	-2.138906	-0.695577	3.648966

C	-2.994433	0.417925	3.722843

C	-2.556891	-1.961141	4.091404

C	-4.280322	0.251962	4.258963

C	-3.849271	-2.111540	4.617946

C	-4.713085	-1.007530	4.708542

H	-2.660264	1.390785	3.349111

H	-1.866804	-2.807574	4.014814

H	-4.952969	1.115628	4.309180

H	-4.178270	-3.098657	4.962263

H	-5.720790	-1.128614	5.120732

C	3.927465	2.828867	2.111991

C	4.921722	2.092167	2.780612

C	4.162948	4.139715	1.670661

C	6.166930	2.695827	3.015464

C	5.417640	4.725816	1.906377

C	6.419145	4.009839	2.582392

H	4.713726	1.060368	3.082964

H	3.366887	4.677134	1.145786

H	6.948772	2.129785	3.535096

H	5.608379	5.748746	1.562367

H	7.394988	4.472045	2.768164

C	-2.007053	3.350699	0.959712

C	-2.434246	4.180422	2.009434

C	-2.918226	2.855416	0.009805

C	-3.794629	4.517835	2.105124

C	-4.273573	3.200682	0.119065

C	-4.716390	4.033027	1.162483

H	-1.698989	4.544292	2.734418

H	-2.562882	2.188178	-0.780709

H	-4.130952	5.166968	2.921778

H	-4.987954	2.787625	-0.600675

H	-5.776815	4.295507	1.243364

C	2.842636	0.355016	-3.104261

C	1.936879	-0.522850	-3.722831

C	4.051876	0.713305	-3.721170

C	2.247263	-1.030976	-4.993851

C	4.350195	0.193960	-4.993229

C	3.448268	-0.672621	-5.632715

H	1.031943	-0.825648	-3.185820

H	4.739050	1.392685	-3.205430

H	1.549465	-1.720279	-5.482719

H	5.289519	0.472728	-5.484390

H	3.682659	-1.073218	-6.625203

**2-Coor-E-T**

SCF Done: -3695.77237483 A.U.

Ni	0.190096	-2.399455	-0.648920

P	-0.329442	-0.224573	-0.409913

O	1.786545	-1.848360	0.414880

O	-2.980622	0.588764	-1.709379

O	1.693582	0.382317	-2.506221

O	-0.976006	2.640250	0.251218

O	-2.358663	-1.819885	1.212390

N	1.094055	0.404815	0.311396

C	1.930716	-0.572543	0.644145

C	3.182317	-0.181059	1.385362

C	3.460617	1.166860	1.681822

C	4.618151	1.504165	2.401152

C	5.514271	0.508073	2.822899

C	5.240671	-0.834327	2.517069

C	4.081321	-1.179034	1.805179

C	-0.613411	0.678696	-1.990017

C	-1.900825	1.049085	-2.451631

C	-2.080567	1.758414	-3.651031

H	-3.089092	2.030323	-3.974201

C	-0.958722	2.085040	-4.426063

H	-1.093445	2.630950	-5.366445

C	0.323596	1.674651	-4.042380

H	1.196846	1.877717	-4.668586

C	0.481030	0.950373	-2.847412

C	-1.597901	0.395024	0.783133

C	-1.665995	1.776718	1.087355

C	-2.473286	2.272841	2.124973

H	-2.500686	3.348330	2.321696

C	-3.244413	1.374125	2.870496

H	-3.888043	1.749175	3.673812

C	-3.226998	0.001498	2.588776

H	-3.847393	-0.694151	3.159125

C	-2.406687	-0.478589	1.552131

C	-1.042573	-2.702092	-2.007499

C	-2.533163	-2.949204	-1.810149

H	-2.680435	-3.606152	-0.935802

H	-2.972639	-1.978542	-1.512061

C	-3.238453	-3.494196	-3.057756

C	-4.758826	-3.542719	-2.921257

C	0.990699	-4.364440	-0.668581

H	0.765485	-4.826374	-1.636418

H	2.042028	-4.145249	-0.454046

C	0.028676	-4.262558	0.326559

H	0.308456	-3.974858	1.346268

H	-0.979360	-4.671418	0.197128

C	-5.514059	-4.285266	-4.017947

H	-6.596403	-4.112834	-3.916062

H	-5.172115	-3.971547	-5.021378

H	-5.312539	-5.371002	-3.939281

H	-2.866544	-4.502122	-3.328926

H	-2.992686	-2.862530	-3.935838

O	-0.505927	-2.685673	-3.110654

O	-5.354520	-3.013888	-1.980895

C	6.227922	-1.911608	2.903677

C	4.864696	2.945959	2.780331

F	7.101748	-2.178695	1.888666

F	5.603072	-3.085213	3.206345

F	6.975496	-1.552954	3.987191

F	6.175772	3.186087	3.066856

F	4.147248	3.297988	3.896010

F	4.494947	3.804539	1.791437

H	3.853125	-2.222148	1.571922

H	2.761850	1.936764	1.341644

H	6.414155	0.775226	3.382881

C	2.875406	1.086380	-2.733344

C	3.015229	2.434430	-2.359516

C	3.952065	0.365963	-3.273860

C	4.254256	3.065357	-2.547913

H	2.169933	2.966212	-1.912056

C	5.187177	1.009864	-3.447715

H	3.807397	-0.684482	-3.546048

C	5.341790	2.359996	-3.091645

H	4.371953	4.113778	-2.251210

H	6.030998	0.450748	-3.867693

H	6.307062	2.858980	-3.230169

C	-4.164703	1.326263	-1.639928

C	-4.166365	2.720396	-1.459162

C	-5.362863	0.594469	-1.667711

C	-5.394071	3.387186	-1.327907

H	-3.217268	3.262150	-1.408401

C	-6.580776	1.277384	-1.524833

H	-5.330000	-0.493921	-1.785790

C	-6.603579	2.672668	-1.362614

H	-5.400986	4.473983	-1.185431

H	-7.517769	0.709356	-1.544114

H	-7.557775	3.200238	-1.256744

C	-0.267751	3.711024	0.790993

C	0.487207	3.593108	1.971510

C	-0.268663	4.904557	0.050336

C	1.244700	4.690525	2.409153

H	0.492685	2.645893	2.518587

C	0.492824	5.993958	0.502182

H	-0.858054	4.961869	-0.870639

C	1.248582	5.893234	1.681639

H	1.860700	4.589052	3.308384

H	0.491667	6.926172	-0.074203

H	1.846116	6.743182	2.028211

C	-2.982467	-2.761004	2.038993

C	-4.158306	-3.376336	1.580242

C	-2.381107	-3.142605	3.250576

C	-4.735785	-4.397446	2.354726

H	-4.600066	-3.061753	0.628321

C	-2.974110	-4.159472	4.015184

H	-1.462034	-2.645659	3.578285

C	-4.149853	-4.788931	3.569777

H	-5.651541	-4.885617	2.002679

H	-2.509693	-4.465300	4.959461

H	-4.605659	-5.585180	4.168355

**TS-Isom-2**

SCF Done: -3695.75083734 A.U.

Ni	1.873354	2.943690	-2.717091

P	1.767894	0.641210	-2.023986

O	3.797784	2.539493	-2.642239

O	-1.017155	-0.264373	-1.711120

O	2.805314	0.874927	0.901852

O	1.721473	-2.419461	-1.509125

O	1.009312	0.883923	-4.906396

N	3.455623	0.337545	-1.813067

C	4.191760	1.360903	-2.219507

C	5.685617	1.158980	-2.232210

C	6.244702	-0.059195	-1.803164

C	7.633351	-0.255975	-1.860652

C	8.479756	0.760615	-2.331644

C	7.921388	1.981953	-2.742847

C	6.532572	2.182747	-2.697882

C	0.932721	0.181547	-0.438722

C	-0.435818	-0.197575	-0.453382

C	-1.129333	-0.547028	0.715650

H	-2.172065	-0.870164	0.654517

C	-0.465946	-0.473543	1.949669

H	-1.000707	-0.739720	2.868074

C	0.861484	-0.037547	2.018793

H	1.378357	0.062967	2.977760

C	1.542999	0.295818	0.832663

C	1.397062	-0.747427	-3.197454

C	1.524296	-2.112378	-2.845791

C	1.388505	-3.142184	-3.793940

H	1.489055	-4.183849	-3.474645

C	1.122234	-2.810932	-5.127997

H	1.004625	-3.606345	-5.872269

C	0.994057	-1.472329	-5.521892

H	0.784151	-1.208656	-6.562023

C	1.142718	-0.457747	-4.559618

C	2.307608	4.311447	-1.510947

C	1.701084	3.913268	-0.155040

H	0.737730	3.403577	-0.315233

H	2.389193	3.167834	0.286906

C	1.545403	5.129151	0.770931

C	0.457095	6.104017	0.308775

C	0.979813	4.275606	-3.955909

H	1.212605	5.311955	-3.691648

H	1.313920	3.949927	-4.950843

C	0.003268	3.543507	-3.249335

H	-0.467909	2.660971	-3.694520

H	-0.516571	4.010965	-2.404292

C	0.350614	7.415404	1.075841

H	1.219090	8.054985	0.826081

H	-0.573470	7.943415	0.795365

H	0.378836	7.250613	2.168578

H	2.502948	5.675808	0.864057

H	1.286481	4.787944	1.794030

O	3.006528	5.273367	-1.704429

O	-0.285470	5.844025	-0.635607

C	8.826264	3.109703	-3.184352

C	8.198452	-1.599585	-1.462619

F	10.008740	2.646940	-3.685347

F	9.134468	3.940270	-2.144481

F	8.245483	3.878790	-4.147176

F	9.552075	-1.569002	-1.309039

F	7.927716	-2.553767	-2.412279

F	7.660985	-2.052120	-0.296955

H	6.087635	3.125373	-3.027759

H	5.576108	-0.837888	-1.423982

H	9.560160	0.602848	-2.378978

C	1.339937	1.279313	-6.207263

C	0.374571	1.993038	-6.934168

C	2.626054	1.055691	-6.730427

C	0.705469	2.493640	-8.204925

H	-0.616511	2.151604	-6.497341

C	2.938498	1.554085	-8.003891

H	3.365985	0.506552	-6.139771

C	1.983102	2.273383	-8.743982

H	-0.043793	3.055434	-8.773828

H	3.941154	1.388892	-8.413958

H	2.237122	2.664797	-9.734915

C	2.691420	-3.360402	-1.171143

C	2.374776	-4.261605	-0.141283

C	3.967639	-3.354459	-1.762426

C	3.353697	-5.167201	0.300883

H	1.372595	-4.237891	0.298859

C	4.934853	-4.263900	-1.309542

H	4.195658	-2.624308	-2.544526

C	4.632584	-5.173525	-0.280456

H	3.109081	-5.873613	1.102481

H	5.938409	-4.235370	-1.746432

H	5.393546	-5.879257	0.069951

C	3.779378	0.305403	1.719596

C	4.660484	1.191047	2.362032

C	3.931628	-1.086045	1.847997

C	5.703243	0.675407	3.146588

H	4.522917	2.269975	2.235638

C	4.975600	-1.585034	2.641614

H	3.254615	-1.763757	1.319729

C	5.862517	-0.712393	3.294593

H	6.392391	1.366105	3.645746

H	5.101817	-2.669649	2.731880

H	6.677707	-1.111589	3.907619

C	-2.402511	-0.205586	-1.859379

C	-3.163283	0.845103	-1.315689

C	-2.997477	-1.187298	-2.667360

C	-4.539985	0.897972	-1.581937

H	-2.677967	1.607586	-0.698316

C	-4.374406	-1.115942	-2.931747

H	-2.371950	-1.983837	-3.082136

C	-5.150020	-0.078729	-2.388115

H	-5.136551	1.716418	-1.163175

H	-4.841313	-1.879338	-3.564272

H	-6.224567	-0.028077	-2.594554

**TS-2-Ins-E-T**

SCF Done: -3695.74965325 A.U.

Ni	1.382695	-1.694720	0.115647

P	1.721819	0.532425	0.227564

O	-0.466672	-1.165054	0.182870

N	0.113019	1.129044	0.140417

C	-0.742448	0.120708	0.177478

C	-2.207685	0.453975	0.234630

C	-3.165804	-0.575551	0.286853

C	-4.532384	-0.264368	0.368323

C	-4.957125	1.073007	0.410544

C	-3.999101	2.099211	0.358919

C	-2.632266	1.796421	0.263646

C	2.209543	1.142871	1.899053

C	2.452774	0.236460	2.961176

C	2.703495	0.690595	4.268347

H	2.903483	-0.027068	5.067799

C	2.685097	2.067970	4.530124

H	2.879126	2.422163	5.548668

C	2.432338	2.994477	3.511525

H	2.424803	4.071016	3.705969

C	2.203889	2.522189	2.207891

C	2.712695	1.458044	-1.019302

C	4.076227	1.806327	-0.811161

C	4.825740	2.465459	-1.800635

H	5.868296	2.728211	-1.603737

C	4.224249	2.777370	-3.029001

H	4.809564	3.290469	-3.800134

C	2.899905	2.411754	-3.290396

H	2.429293	2.612885	-4.257232

C	2.168727	1.737253	-2.295252

C	3.250916	-2.099265	-0.502925

C	3.310935	-1.986907	-2.046963

H	3.706876	-0.973826	-2.233549

H	4.086708	-2.702890	-2.382222

C	2.017839	-2.196662	-2.852468

C	1.778537	-3.628965	-3.321840

C	2.375351	-3.516010	0.125130

H	2.756334	-4.089461	-0.742112

H	2.931747	-3.682494	1.056066

C	0.924973	-3.571239	0.240809

H	0.474090	-3.723137	1.229349

H	0.312740	-3.890751	-0.611565

C	0.733456	-3.820839	-4.413698

H	-0.202233	-3.286842	-4.163750

H	1.101236	-3.389910	-5.364686

H	0.528232	-4.892376	-4.558907

H	1.989659	-1.523040	-3.729917

H	1.135031	-1.887523	-2.243566

O	4.272870	-1.868220	0.148397

O	2.391222	-4.587169	-2.845380

C	-4.432165	3.542275	0.478668

C	-5.553139	-1.379171	0.352993

F	-4.309442	3.992782	1.768961

F	-5.736752	3.718782	0.124517

F	-3.678310	4.367939	-0.296341

F	-5.099382	-2.489713	1.000522

F	-5.856951	-1.761571	-0.922992

F	-6.724069	-1.007138	0.945414

H	-1.879103	2.588014	0.210584

H	-2.825853	-1.614205	0.271682

H	-6.020953	1.312958	0.484512

O	2.413646	-1.109201	2.636820

O	2.060937	3.408455	1.151656

O	4.604086	1.473589	0.418489

O	0.920133	1.208371	-2.598865

C	1.169776	4.471955	1.265529

C	-0.093640	4.329809	1.866965

C	1.553397	5.683514	0.666657

C	-0.977908	5.419461	1.862719

C	0.658268	6.765240	0.672783

C	-0.606754	6.639249	1.270307

H	-0.379306	3.368753	2.304423

H	2.542173	5.758638	0.202614

H	-1.975532	5.298983	2.297809

H	0.956440	7.711949	0.207784

H	-1.304289	7.483748	1.268056

C	2.669386	-2.071599	3.620067

C	1.656230	-2.456211	4.513728

C	3.920539	-2.707532	3.610838

C	1.913442	-3.493176	5.425544

C	4.160722	-3.746029	4.526002

C	3.162815	-4.137436	5.434813

H	0.686465	-1.948589	4.485077

H	4.666995	-2.392636	2.875126

H	1.129538	-3.802702	6.125955

H	5.132365	-4.252758	4.524257

H	3.355116	-4.948813	6.145276

C	5.988293	1.553068	0.616086

C	6.782560	0.437386	0.309047

C	6.532389	2.705907	1.201564

C	8.157513	0.490976	0.587832

C	7.909880	2.743706	1.474846

C	8.723613	1.640885	1.165659

H	6.304402	-0.455310	-0.107857

H	5.875340	3.547306	1.443739

H	8.788142	-0.375823	0.359339

H	8.345356	3.637936	1.934926

H	9.797030	1.674170	1.382868

C	-0.042805	2.040310	-3.167961

C	-0.890642	1.464156	-4.127877

C	-0.213878	3.373932	-2.757311

C	-1.919950	2.236558	-4.687700

C	-1.243533	4.134505	-3.331883

C	-2.097554	3.574144	-4.297285

H	-0.734841	0.420877	-4.421128

H	0.436790	3.795788	-1.985583

H	-2.583035	1.788022	-5.436257

H	-1.386047	5.171080	-3.006724

H	-2.901329	4.174549	-4.736799

**2-cycle5-C**

SCF Done: -3695.7775439 A.U.

Ni	0.356088	-2.288931	0.339315

P	0.706480	-0.111026	-0.226136

O	-1.403093	-1.923474	-0.183581

O	-0.622315	0.592380	2.317072

O	3.529806	1.098456	0.062746

O	2.152933	-1.821550	-2.228418

O	0.886661	2.717165	-1.399356

N	-0.919714	0.372812	-0.545466

C	-1.716133	-0.681651	-0.490379

C	-3.167994	-0.481098	-0.827183

C	-3.653131	0.795593	-1.170021

C	-5.005161	0.966803	-1.507746

C	-5.889028	-0.124773	-1.497440

C	-5.405480	-1.394673	-1.144274

C	-4.053654	-1.575577	-0.813067

C	1.383185	0.978466	1.105236

C	0.616532	1.196896	2.282303

C	1.082041	2.009696	3.332194

H	0.444315	2.188755	4.201667

C	2.363916	2.567827	3.249034

H	2.732088	3.201265	4.063930

C	3.191475	2.302042	2.149618

H	4.209512	2.698112	2.097387

C	2.697964	1.503441	1.100545

C	1.498774	0.440601	-1.804573

C	2.099820	-0.503768	-2.668640

C	2.527693	-0.157137	-3.963205

H	2.969696	-0.920078	-4.610351

C	2.365646	1.161861	-4.406173

H	2.695608	1.438064	-5.413742

C	1.788928	2.132014	-3.575429

H	1.673119	3.168070	-3.907189

C	1.362195	1.764409	-2.288243

C	-0.156008	-4.106385	0.805964

H	-0.072290	-4.649602	-0.155347

H	-1.198861	-4.132642	1.155725

C	0.860266	-4.603789	1.847663

H	1.101508	-5.689205	1.804833

H	0.490802	-4.428559	2.880752

C	2.105492	-3.780630	1.735427

C	3.418416	-4.150216	2.392196

H	4.025750	-4.705936	1.648565

O	2.042796	-2.716338	1.055149

C	-6.358809	-2.564726	-1.060660

C	-5.494102	2.327037	-1.947910

H	-6.940721	0.012707	-1.761605

H	-3.666702	-2.562277	-0.546828

H	-2.957070	1.639980	-1.168227

F	-5.198255	2.562430	-3.266656

F	-6.846095	2.453745	-1.821936

F	-4.918268	3.331777	-1.232687

F	-7.433203	-2.406280	-1.887362

F	-5.752492	-3.738215	-1.395935

F	-6.851607	-2.720054	0.204034

C	4.199889	-2.921378	2.879648

H	4.424871	-2.242136	2.034741

H	5.178638	-3.218309	3.304144

C	3.413578	-2.126567	3.926645

C	4.073114	-0.882462	4.497073

H	3.608798	-0.617133	5.459650

H	5.164108	-1.004176	4.616403

H	3.907338	-0.040741	3.795167

H	3.217442	-4.845477	3.225805

O	2.278061	-2.472175	4.255849

C	-1.279327	0.423049	3.539853

C	-0.733160	-0.387023	4.551430

C	-2.547105	1.007750	3.677106

C	-1.478831	-0.598642	5.721806

H	0.249687	-0.852767	4.419580

C	-3.282665	0.779275	4.851478

H	-2.942151	1.620572	2.860939

C	-2.750301	-0.018984	5.877059

H	-1.063620	-1.233386	6.513071

H	-4.275947	1.228852	4.961488

H	-3.326877	-0.195631	6.791775

C	4.554012	1.934733	-0.373490

C	5.824040	1.358245	-0.540738

C	4.318202	3.277399	-0.718522

C	6.870512	2.137494	-1.059965

H	5.973451	0.309765	-0.262630

C	5.376076	4.045870	-1.226547

H	3.312396	3.692703	-0.604186

C	6.652824	3.482859	-1.399052

H	7.862116	1.689768	-1.192008

H	5.195911	5.091476	-1.501347

H	7.472071	4.089317	-1.800136

C	3.259685	-2.610030	-2.514155

C	3.013124	-3.979578	-2.716162

C	4.574421	-2.106651	-2.525080

C	4.092000	-4.850133	-2.931727

H	1.979018	-4.338278	-2.703199

C	5.641344	-2.990378	-2.751115

H	4.752764	-1.039754	-2.358389

C	5.409552	-4.361411	-2.954482

H	3.897498	-5.916683	-3.093486

H	6.665519	-2.599300	-2.763531

H	6.248671	-5.042758	-3.132003

C	-0.055498	3.648299	-1.832528

C	0.058066	4.951086	-1.321359

C	-1.123999	3.292403	-2.674281

C	-0.909146	5.909485	-1.662352

H	0.892048	5.194592	-0.654948

C	-2.084238	4.261005	-3.004679

H	-1.206590	2.263566	-3.036507

C	-1.980060	5.570854	-2.505369

H	-0.822046	6.926481	-1.263053

H	-2.936427	3.976423	-3.630242

H	-2.736732	6.319437	-2.763718

**TS-1-Coor-E-T**

SCF Done: -3582.44209927 A.U.

Ni	-1.041937	2.105211	-1.324650

P	-0.859574	0.075786	-0.640922

O	0.790163	2.280389	-0.603896

O	-2.615112	1.624957	1.358455

O	-0.704725	-2.703053	0.587555

O	-3.498260	-1.259658	-1.189259

O	0.994621	-1.102738	-2.795978

N	0.780173	-0.032156	-0.153685

C	1.338209	1.175948	-0.185828

C	2.745273	1.295757	0.334598

C	3.468696	0.156173	0.734280

C	4.766653	0.291580	1.252278

C	5.361965	1.558315	1.367099

C	4.643842	2.692772	0.955427

C	3.342807	2.565677	0.444213

C	-1.654239	-0.524613	0.937812

C	-2.380457	0.332700	1.803252

C	-2.768166	-0.070657	3.094917

H	-3.306334	0.626838	3.741920

C	-2.442219	-1.355409	3.545000

H	-2.744587	-1.669185	4.550149

C	-1.733558	-2.237566	2.723145

H	-1.477514	-3.247772	3.055834

C	-1.343267	-1.812504	1.441792

C	-1.208646	-1.267023	-1.867372

C	-2.502444	-1.833882	-1.975141

C	-2.801230	-2.867149	-2.877612

H	-3.816961	-3.269295	-2.926304

C	-1.786439	-3.352014	-3.713247

H	-2.008525	-4.151534	-4.428696

C	-0.503084	-2.795442	-3.672609

H	0.285395	-3.143279	-4.345832

C	-0.226570	-1.750755	-2.770421

C	-2.839235	1.987558	-2.047244

H	-3.396218	1.057100	-1.864761

H	-2.647318	2.076828	-3.133409

C	-3.618742	3.207460	-1.521504

H	-4.447222	3.536948	-2.186331

H	-4.106773	2.967352	-0.553264

C	-2.683336	4.348851	-1.225850

C	-3.189683	5.763143	-1.070999

H	-2.369994	6.439076	-0.782882

H	-3.641355	6.113585	-2.018609

H	-3.988117	5.797005	-0.305806

O	-1.466093	4.092889	-1.079889

H	2.769514	3.443354	0.133704

H	6.371797	1.660143	1.772562

H	3.001413	-0.827459	0.629796

C	5.494943	-0.936002	1.746912

C	5.295964	4.055241	1.008021

F	5.884486	4.375672	-0.182612

F	6.270842	4.117926	1.961126

F	4.392936	5.039471	1.279720

F	6.837036	-0.729704	1.864705

F	5.044341	-1.317574	2.987091

F	5.312123	-2.004000	0.923317

C	0.133244	1.980788	-3.653233

C	0.141393	3.324280	-3.502647

H	0.907730	1.344805	-3.215876

H	-0.610004	1.474844	-4.281325

H	-0.614018	3.962519	-3.976873

H	0.897940	3.825596	-2.890593

C	2.154680	-1.823277	-3.082162

C	3.033401	-1.273722	-4.029075

C	2.478052	-3.002709	-2.388985

C	4.253954	-1.917983	-4.286533

H	2.753855	-0.350682	-4.547119

C	3.698151	-3.638399	-2.663607

H	1.791484	-3.399998	-1.634961

C	4.588471	-3.102698	-3.610371

H	4.943489	-1.489703	-5.022833

H	3.958471	-4.552021	-2.117493

H	5.541768	-3.602483	-3.813859

C	-4.527036	-2.048217	-0.679740

C	-5.821859	-1.504492	-0.721172

C	-4.283574	-3.292914	-0.071906

C	-6.885700	-2.217942	-0.146356

H	-5.977614	-0.535985	-1.207470

C	-5.359197	-3.997036	0.489825

H	-3.263466	-3.686881	-0.034854

C	-6.660471	-3.466468	0.456537

H	-7.897091	-1.797023	-0.180387

H	-5.173705	-4.965712	0.967892

H	-7.493443	-4.022889	0.899619

C	0.357903	-3.463064	1.072407

C	1.354560	-2.909602	1.896104

C	0.442534	-4.793049	0.628002

C	2.446308	-3.705303	2.274710

H	1.279088	-1.862769	2.203879

C	1.541563	-5.577369	1.016442

H	-0.346653	-5.192786	-0.017258

C	2.543585	-5.039322	1.840513

H	3.239644	-3.264656	2.887148

H	1.608289	-6.616134	0.673063

H	3.400960	-5.652707	2.137928

C	-3.607148	2.394446	1.959451

C	-4.951801	1.975697	1.978420

C	-3.236992	3.660157	2.444016

C	-5.928063	2.840230	2.497664

H	-5.218132	0.987316	1.589243

C	-4.227591	4.516569	2.953208

H	-2.183018	3.953715	2.411622

C	-5.572467	4.110616	2.984595

H	-6.975891	2.519151	2.514279

H	-3.941456	5.503203	3.335152

H	-6.340566	4.779976	3.386814

**1-Coor-E-T**

SCF Done: -3582.44575348 A.U.

Ni	0.771725	-2.275978	-1.616218

P	1.000130	-0.297514	-0.511649

O	-1.107945	-2.068045	-1.003269

O	2.954837	-2.108967	1.056927

O	1.100190	2.301419	1.056952

O	3.751743	0.881029	-0.993282

O	-0.738758	1.187916	-2.530266

N	-0.600058	-0.016159	0.041902

C	-1.409479	-1.007163	-0.312206

C	-2.842250	-0.923733	0.148949

C	-3.308150	0.189384	0.872798

C	-4.636915	0.229148	1.326336

C	-5.517390	-0.829944	1.054668

C	-5.055119	-1.933395	0.319119

C	-3.726566	-1.983309	-0.129459

C	1.920590	0.048194	1.070330

C	2.685325	-0.935533	1.743907

C	3.211215	-0.716881	3.031198

H	3.810707	-1.496127	3.509269

C	2.968165	0.500681	3.676691

H	3.380931	0.675336	4.676390

C	2.231931	1.508218	3.043053

H	2.057216	2.474493	3.525121

C	1.728163	1.278279	1.751317

C	1.458190	1.142080	-1.595684

C	2.788024	1.625007	-1.668839

C	3.146714	2.729320	-2.460428

H	4.186562	3.066962	-2.483315

C	2.158087	3.367979	-3.222131

H	2.426779	4.226012	-3.848422

C	0.842377	2.890624	-3.227713

H	0.074168	3.355800	-3.851922

C	0.506174	1.774113	-2.437173

C	2.618995	-2.414883	-2.256646

H	2.782180	-3.482153	-2.490836

H	3.325513	-2.108862	-1.471549

C	2.753093	-1.554908	-3.523409

H	3.015800	-0.509165	-3.260853

C	1.447266	-1.460611	-4.285585

C	1.456812	-1.083988	-5.751957

H	1.927757	-1.893611	-6.342054

H	0.431832	-0.916413	-6.115783

H	2.068034	-0.176757	-5.913961

O	0.376405	-1.669536	-3.689329

H	3.567748	-1.898584	-4.196502

C	0.839314	-4.141682	-0.703667

H	1.879352	-4.404657	-0.484029

H	0.175303	-4.028958	0.159666

C	0.309344	-4.277917	-1.990033

H	-0.772858	-4.253708	-2.153856

H	0.923544	-4.641364	-2.822074

C	-6.009522	-3.048318	-0.041669

C	-5.094224	1.403438	2.159459

H	-6.549680	-0.796379	1.411903

H	-3.354492	-2.842273	-0.693636

H	-2.618980	1.016261	1.068422

F	-6.586208	-2.840043	-1.262495

F	-5.379668	-4.255694	-0.102828

F	-7.026195	-3.160927	0.862055

F	-4.583753	1.341481	3.432128

F	-4.682429	2.590485	1.634702

F	-6.450730	1.456339	2.281915

C	0.177079	3.113258	1.709969

C	0.199738	4.481399	1.391677

C	-0.793996	2.588914	2.581614

C	-0.762532	5.333743	1.957524

H	0.965143	4.857345	0.704807

C	-1.749379	3.453093	3.137797

H	-0.805224	1.515805	2.793572

C	-1.736345	4.825438	2.832499

H	-0.745151	6.401845	1.712134

H	-2.527089	3.040561	3.788336

H	-2.488377	5.492195	3.267847

C	3.161316	-3.290848	1.765194

C	4.252761	-4.084932	1.376810

C	2.266842	-3.729983	2.759137

C	4.448834	-5.333260	1.989545

H	4.929268	-3.716163	0.599291

C	2.481350	-4.975333	3.368498

H	1.415869	-3.102362	3.041992

C	3.568760	-5.781267	2.988127

H	5.298580	-5.954647	1.684895

H	1.783688	-5.322011	4.139331

H	3.725753	-6.754544	3.465240

C	4.862566	1.519429	-0.442923

C	6.102023	0.877122	-0.593285

C	4.747610	2.707516	0.300545

C	7.241948	1.435157	0.005777

H	6.154922	-0.049890	-1.173067

C	5.898117	3.257316	0.885762

H	3.767388	3.178499	0.421542

C	7.146468	2.627743	0.741853

H	8.210326	0.935210	-0.109830

H	5.813061	4.181521	1.468770

H	8.038997	3.062725	1.204436

C	-1.874520	1.973473	-2.711284

C	-2.827868	1.502382	-3.628334

C	-2.108296	3.132646	-1.950473

C	-4.028575	2.210131	-3.792328

H	-2.618033	0.585795	-4.188616

C	-3.310887	3.832425	-2.132463

H	-1.367609	3.463698	-1.215901

C	-4.272337	3.378932	-3.051895

H	-4.776352	1.843138	-4.504561

H	-3.501801	4.731153	-1.535148

H	-5.210813	3.928530	-3.182848

**TS-Isom-1**

SCF Done: -3582.43030013 A.U.

Ni	0.294904	-1.022630	5.556835

P	1.173445	-1.411614	3.508490

O	-0.966098	0.132525	4.355601

O	2.075579	0.978431	5.078751

O	2.973129	-1.323935	0.968599

O	3.703785	-2.585020	4.297485

O	-0.238859	-3.695682	1.872043

N	-0.076355	-0.930068	2.441950

C	-0.945193	-0.149483	3.083096

C	-2.019029	0.489723	2.239429

C	-2.970079	1.335480	2.840091

C	-3.949558	1.966806	2.056857

C	-3.986690	1.769538	0.667106

C	-3.040673	0.920349	0.069416

C	-2.067098	0.275830	0.848414

C	2.479730	-0.177449	3.029899

C	2.685928	0.974092	3.830898

C	3.467368	2.056236	3.391028

H	3.599586	2.929818	4.034087

C	4.067994	1.999514	2.126446

H	4.681766	2.839268	1.782708

C	3.898628	0.879784	1.306141

H	4.364570	0.819076	0.318103

C	3.104763	-0.188125	1.759509

C	1.801159	-3.052376	2.956050

C	3.084957	-3.452155	3.407448

C	3.666501	-4.676101	3.041402

H	4.660442	-4.940483	3.412022

C	2.941441	-5.544616	2.214846

H	3.376607	-6.508552	1.928658

C	1.649934	-5.216084	1.786535

H	1.066794	-5.908814	1.173598

C	1.078401	-3.987565	2.170321

C	0.291374	-2.935231	5.974590

H	0.160782	-3.029320	7.064096

H	1.237720	-3.402216	5.661952

C	-0.924611	-3.523245	5.262117

H	-1.000138	-3.234885	4.196273

C	-2.268673	-3.274778	5.958801

C	-3.512318	-3.446251	5.094993

H	-3.596586	-2.580310	4.410374

H	-3.442396	-4.349508	4.460881

H	-4.413234	-3.493983	5.726064

O	-2.349752	-2.980378	7.150221

H	-0.821781	-4.635619	5.234039

C	-0.495669	-0.280503	7.303327

H	-1.293511	-0.975860	7.590728

H	-0.792293	0.740296	7.033692

C	0.846002	-0.601715	7.519637

H	1.626812	0.163227	7.438262

H	1.137974	-1.517281	8.043904

C	-3.017924	0.743861	-1.431340

C	-5.002073	2.821550	2.725836

H	-4.742101	2.271873	0.057536

H	-1.333693	-0.398107	0.395447

H	-2.923577	1.492225	3.921319

F	-2.040130	1.517563	-2.007313

F	-2.756045	-0.542275	-1.788160

F	-4.196302	1.104325	-2.012299

F	-4.494584	3.504712	3.791322

F	-5.536916	3.737051	1.867786

F	-6.036209	2.064557	3.196731

C	1.890185	2.210926	5.728589

C	2.844323	2.649765	6.659619

C	0.703514	2.923654	5.498098

C	2.606135	3.841708	7.365247

H	3.753626	2.061237	6.821048

C	0.479842	4.112214	6.211596

H	-0.031642	2.518608	4.795419

C	1.428583	4.574271	7.140597

H	3.343241	4.193730	8.095587

H	-0.445871	4.674527	6.045545

H	1.245806	5.500595	7.696006

C	5.096055	-2.490535	4.323590

C	5.710299	-2.479196	5.585478

C	5.847076	-2.323970	3.146600

C	7.100482	-2.302078	5.668370

H	5.093190	-2.607069	6.480593

C	7.236522	-2.156808	3.246469

H	5.339690	-2.315636	2.176896

C	7.867990	-2.145586	4.502328

H	7.584250	-2.292989	6.651627

H	7.827677	-2.023145	2.333277

H	8.952967	-2.011712	4.571158

C	2.600313	-1.164659	-0.366250

C	3.221125	-2.002079	-1.307615

C	1.582145	-0.272926	-0.748550

C	2.814556	-1.940916	-2.651301

H	4.006037	-2.690122	-0.977108

C	1.187106	-0.223305	-2.093627

H	1.093186	0.344208	0.010842

C	1.801295	-1.052731	-3.049036

H	3.299453	-2.590436	-3.389074

H	0.372975	0.448904	-2.384679

H	1.486933	-1.009540	-4.097429

C	-0.808468	-4.163543	0.687930

C	-2.077113	-4.757518	0.781805

C	-0.188796	-3.970943	-0.559061

C	-2.733089	-5.167222	-0.389506

H	-2.535259	-4.885292	1.767777

C	-0.854107	-4.393119	-1.719954

H	0.789918	-3.484697	-0.616866

C	-2.123396	-4.991684	-1.642545

H	-3.724555	-5.628604	-0.318278

H	-0.377113	-4.235076	-2.693579

H	-2.637521	-5.312758	-2.554913

**1-Coor-E-C**

SCF Done: -3582.4389905 A.U.

Ni	-0.136267	2.864441	-0.646618

P	-0.946003	0.696404	-0.158375

O	1.530069	1.760359	-0.993150

O	-2.068415	2.365792	2.145580

O	-1.569999	-2.147281	0.711010

O	-3.976595	0.742359	-0.100064

O	-0.354012	-0.442378	-2.952757

N	0.497734	-0.224354	-0.227608

C	1.527368	0.511390	-0.636566

C	2.873835	-0.170874	-0.682464

C	3.015543	-1.531344	-0.352060

C	4.286396	-2.130147	-0.361126

C	5.425541	-1.387246	-0.710073

C	5.280659	-0.033632	-1.054138

C	4.014981	0.571895	-1.039124

C	-1.643600	0.120681	1.463542

C	-2.013065	1.023968	2.490566

C	-2.395847	0.575529	3.768213

H	-2.698226	1.296934	4.531520

C	-2.407341	-0.798622	4.038197

H	-2.707924	-1.150131	5.031488

C	-2.085862	-1.728050	3.042730

H	-2.134474	-2.804251	3.232219

C	-1.733054	-1.261912	1.763604

C	-2.127797	-0.034401	-1.396566

C	-3.529390	0.001186	-1.189277

C	-4.438047	-0.596265	-2.078691

H	-5.511072	-0.545695	-1.873917

C	-3.947394	-1.226909	-3.229951

H	-4.647439	-1.688528	-3.935170

C	-2.577215	-1.222918	-3.515105

H	-2.188661	-1.665475	-4.436822

C	-1.685200	-0.605399	-2.618086

C	0.928002	4.467726	-1.087570

H	0.438453	5.144205	-1.806991

H	1.819541	4.002381	-1.537291

C	1.244834	5.177902	0.234177

H	0.404699	5.844020	0.529786

C	-1.420997	3.258306	-2.131536

H	-1.870895	2.318754	-2.471471

H	-0.928880	3.846400	-2.914996

C	-1.850999	3.867027	-0.924307

H	-1.706182	4.942878	-0.764021

H	-2.629702	3.396482	-0.314548

C	6.484417	0.764812	-1.496442

C	4.425317	-3.572557	0.066164

F	7.646600	0.263871	-0.985602

F	6.617160	0.766865	-2.855842

F	3.463363	-4.362374	-0.484975

F	4.307316	-3.703575	1.427008

F	5.635509	-4.100250	-0.275575

F	6.396750	2.070417	-1.107938

H	6.412371	-1.856749	-0.713015

H	3.891209	1.626958	-1.296526

H	2.120829	-2.104999	-0.092287

C	1.376338	4.182322	1.365185

C	2.257026	4.488642	2.553621

H	2.007998	5.480444	2.974614

H	2.151041	3.714988	3.328054

H	3.312557	4.537384	2.223721

H	2.138250	5.835338	0.175257

O	0.733721	3.118272	1.300851

C	-0.919353	-3.358845	0.921755

C	-1.484480	-4.497482	0.323938

C	0.296653	-3.437320	1.623838

C	-0.821414	-5.730673	0.433317

H	-2.430829	-4.401131	-0.218409

C	0.947886	-4.676217	1.722849

H	0.726704	-2.530563	2.059850

C	0.392302	-5.825355	1.133358

H	-1.261228	-6.620892	-0.030787

H	1.912493	-4.733060	2.237172

H	0.909923	-6.787454	1.211421

C	-1.945144	3.356021	3.112166

C	-0.965172	3.318616	4.122443

C	-2.798923	4.465133	2.985340

C	-0.860034	4.396855	5.014606

H	-0.298771	2.454758	4.199608

C	-2.673063	5.542115	3.876921

H	-3.551210	4.466952	2.190391

C	-1.708548	5.511870	4.897721

H	-0.101584	4.365137	5.805570

H	-3.341348	6.404890	3.775766

H	-1.618766	6.349307	5.598045

C	0.323206	-1.469937	-3.605991

C	1.151030	-1.107338	-4.680449

C	0.254129	-2.801107	-3.158662

C	1.914643	-2.095943	-5.320651

H	1.189462	-0.059167	-4.993961

C	1.018248	-3.778235	-3.814122

H	-0.372164	-3.054701	-2.297816

C	1.847517	-3.433113	-4.895392

H	2.563728	-1.815982	-6.158163

H	0.975573	-4.815222	-3.462378

H	2.444927	-4.201594	-5.397929

C	-5.110482	0.329920	0.597311

C	-6.015265	1.330187	0.987787

C	-5.310269	-1.011774	0.968387

C	-7.135392	0.981144	1.757909

H	-5.829178	2.364883	0.682865

C	-6.439158	-1.345848	1.731339

H	-4.582583	-1.771550	0.667581

C	-7.354526	-0.355958	2.128554

H	-7.842974	1.760425	2.063157

H	-6.597041	-2.389787	2.025581

H	-8.232288	-0.625492	2.725858

**TS-1-Ins-E-C**

SCF Done: -3582.42786157 A.U.

Ni	1.267873	-2.337237	0.298784

P	1.183426	-0.172761	0.199765

O	-0.630106	-2.175811	-0.201744

N	-0.485494	0.169054	-0.017369

C	-1.135530	-0.969337	-0.225973

C	-2.605128	-0.891197	-0.529453

C	-3.335768	-2.074964	-0.736717

C	-4.711235	-2.021012	-1.009686

C	-5.370304	-0.785014	-1.098135

C	-4.638283	0.397747	-0.897076

C	-3.266433	0.349600	-0.604441

C	1.769132	0.814369	1.640090

C	3.061822	1.392235	1.697349

C	3.473257	2.185393	2.781087

H	4.477540	2.617582	2.787960

C	2.590475	2.388875	3.851401

H	2.907783	3.000009	4.703580

C	1.331724	1.777307	3.868531

H	0.657596	1.886590	4.722821

C	0.944058	0.973794	2.780675

C	1.873282	0.638583	-1.317136

C	1.631234	2.010879	-1.575029

C	1.969375	2.611907	-2.798235

H	1.771569	3.677484	-2.947666

C	2.560186	1.831671	-3.800773

H	2.822599	2.288665	-4.761167

C	2.816800	0.471762	-3.589045

H	3.265305	-0.145916	-4.372231

C	2.474804	-0.112948	-2.355644

C	0.935812	-4.430048	0.227762

H	1.494303	-5.354849	0.032325

H	0.436735	-4.156185	-0.721081

C	-0.065187	-4.671482	1.352656

H	0.449372	-4.717338	2.338988

H	-0.799855	-3.851492	1.425993

C	-0.798875	-6.021814	1.205669

C	-2.180918	-6.100894	1.845108

H	-2.527145	-7.145591	1.878652

H	-2.177853	-5.666101	2.861946

H	-2.896737	-5.503797	1.247259

O	-0.301338	-6.974182	0.611663

C	2.784031	-3.733726	0.809318

H	2.704865	-4.379157	1.694635

H	3.312879	-4.203831	-0.028930

C	3.061089	-2.334595	1.032591

H	3.774031	-1.826336	0.372266

H	3.025403	-1.941332	2.058416

C	-5.314874	1.740652	-1.048774

C	-5.461873	-3.310778	-1.248450

F	-4.865708	2.644728	-0.135885

F	-5.074360	2.280970	-2.286930

F	-5.081683	-4.282254	-0.364001

F	-5.226739	-3.805869	-2.497850

F	-6.809424	-3.152468	-1.125010

F	-6.668273	1.654580	-0.914092

H	-6.442153	-0.743352	-1.307177

H	-2.693879	1.264063	-0.423558

H	-2.810666	-3.031603	-0.674710

O	2.616065	-1.480193	-2.159243

O	1.136019	2.777794	-0.529342

O	-0.214154	0.219580	2.833832

O	3.928803	1.064359	0.663526

C	-1.377294	0.786914	3.353725

C	-1.808864	2.068688	2.970123

C	-2.146612	-0.011068	4.215110

C	-3.025719	2.553313	3.473042

C	-3.365358	0.485830	4.703364

C	-3.806653	1.768752	4.339408

H	-1.207340	2.660930	2.273854

H	-1.782256	-1.006635	4.488633

H	-3.371616	3.547785	3.169635

H	-3.969119	-0.134625	5.375360

H	-4.757837	2.153366	4.723026

C	4.948045	1.946070	0.302739

C	6.238106	1.410113	0.161260

C	4.684399	3.294429	0.004188

C	7.279560	2.239268	-0.286272

C	5.737584	4.111992	-0.432480

C	7.035016	3.590816	-0.579698

H	6.408628	0.356737	0.406217

H	3.664826	3.679443	0.103516

H	8.288223	1.825335	-0.396627

H	5.538148	5.163197	-0.669928

H	7.850726	4.235564	-0.924197

C	0.078778	3.656166	-0.762812

C	-0.998735	3.324756	-1.603231

C	0.092798	4.864294	-0.046509

C	-2.071103	4.222215	-1.722776

C	-0.986743	5.752481	-0.178402

C	-2.069150	5.437557	-1.016161

H	-0.999916	2.365602	-2.128785

H	0.941769	5.088980	0.607490

H	-2.927638	3.950222	-2.348328

H	-0.976587	6.696494	0.378319

H	-2.911981	6.130351	-1.112231

C	3.729531	-2.147839	-2.665215

C	5.025103	-1.599816	-2.620490

C	3.512615	-3.452031	-3.142254

C	6.106211	-2.372679	-3.072909

C	4.606628	-4.213782	-3.581426

C	5.905251	-3.677951	-3.553038

H	5.179912	-0.586033	-2.237447

H	2.492543	-3.848422	-3.163693

H	7.116566	-1.948940	-3.040331

H	4.437923	-5.230435	-3.953954

H	6.755485	-4.273993	-3.901289

**1-beta-T**

SCF Done: -3582.45758525 A.U.

Ni	-0.209856	-2.252102	-0.521346

P	-0.921873	-0.260001	-0.424504

O	1.537778	-1.506799	-0.187390

N	0.489837	0.599557	0.035445

C	1.536759	-0.219599	0.048658

C	2.879973	0.386134	0.345692

C	3.003125	1.754882	0.652429

C	4.268178	2.307742	0.908289

C	5.420171	1.505297	0.870738

C	5.294056	0.138107	0.575619

C	4.033562	-0.420328	0.313119

C	-1.390992	0.682975	-1.940396

C	-1.340496	0.106349	-3.231955

C	-1.482424	0.888781	-4.392899

H	-1.448317	0.408197	-5.374616

C	-1.671496	2.271028	-4.271768

H	-1.789076	2.881063	-5.174100

C	-1.738269	2.878581	-3.011008

H	-1.904140	3.954561	-2.905054

C	-1.602777	2.082158	-1.862553

C	-2.238196	-0.007651	0.838725

C	-3.606213	0.160632	0.508485

C	-4.596013	0.309073	1.496088

H	-5.641159	0.433778	1.200484

C	-4.225065	0.266674	2.846116

H	-4.990568	0.385437	3.620914

C	-2.892903	0.048172	3.218033

H	-2.599772	-0.008169	4.269946

C	-1.918188	-0.113361	2.217588

C	-1.757788	-3.320965	-0.726297

H	-2.174611	-3.276203	-1.742981

H	-2.526809	-3.184036	0.050710

C	-0.750397	-4.418468	-0.458061

H	-0.666196	-5.130255	-1.302489

C	-0.901381	-5.132278	0.898540

H	-1.034882	-4.377013	1.692823

H	-1.831465	-5.730544	0.882699

H	0.336004	-3.977691	-0.457592

H	6.401579	1.936973	1.083036

H	3.923549	-1.484052	0.086725

H	2.098322	2.369288	0.690705

C	6.529098	-0.728365	0.480931

C	4.385315	3.790765	1.174586

F	7.553402	-0.240870	1.238661

F	6.285361	-2.005022	0.891909

F	6.987889	-0.807067	-0.803696

F	5.568452	4.117000	1.767493

F	3.383209	4.244118	1.976486

F	4.314035	4.512859	0.010277

C	0.285176	-6.030474	1.268942

C	1.231595	-7.680899	3.042097

H	1.136452	-7.949351	4.105350

H	1.104646	-8.591806	2.425911

H	2.249706	-7.299660	2.839718

O	-1.753135	2.626339	-0.596566

C	-1.114281	3.827754	-0.286400

C	-1.818594	4.730131	0.525791

C	0.206003	4.093906	-0.689319

C	-1.191364	5.917139	0.936150

C	0.820409	5.283744	-0.269550

C	0.125857	6.199678	0.539589

H	-2.840091	4.487835	0.836521

H	0.742984	3.361257	-1.298558

H	-1.739234	6.622481	1.571331

H	1.860406	5.476271	-0.552563

H	0.614072	7.123725	0.867387

O	-0.590066	-0.329068	2.531053

O	-3.935066	0.057244	-0.834203

O	-1.204055	-1.271496	-3.313061

C	-5.064971	0.709453	-1.327331

C	-5.943464	-0.042783	-2.122523

C	-5.272313	2.083817	-1.116589

C	-7.048998	0.591659	-2.710852

C	-6.386592	2.701761	-1.703728

C	-7.277568	1.961477	-2.500127

H	-5.747640	-1.109428	-2.271268

H	-4.555495	2.650196	-0.514321

H	-7.736977	0.007896	-3.332832

H	-6.551870	3.773641	-1.545515

H	-8.143454	2.451901	-2.958001

C	-0.251812	-1.060069	3.670804

C	-0.839420	-2.305870	3.955555

C	0.786906	-0.551522	4.465927

C	-0.371976	-3.049746	5.049802

C	1.243237	-1.305349	5.559162

C	0.667709	-2.552328	5.854996

H	-1.646835	-2.687167	3.322607

H	1.228017	0.416866	4.210259

H	-0.809326	-4.033615	5.250632

H	2.055793	-0.912225	6.180684

H	1.031776	-3.138394	6.705913

C	-0.491237	-1.829219	-4.374425

C	0.793173	-1.375538	-4.725895

C	-1.069580	-2.933351	-5.021214

C	1.495611	-2.039543	-5.742849

C	-0.350521	-3.592568	-6.031285

C	0.930212	-3.147560	-6.398039

H	1.231860	-0.520162	-4.202616

H	-2.071927	-3.259763	-4.725567

H	2.498580	-1.692383	-6.015923

H	-0.799946	-4.454779	-6.536935

H	1.487186	-3.662461	-7.188197

H	0.429922	-6.845382	0.529533

H	1.232482	-5.448986	1.244232

C	0.172632	-6.647449	2.668347

O	-0.719918	-6.325478	3.449118

**TS-1-Ins-E-T**

SCF Done: -3582.41270499 A.U.

Ni	0.410899	-2.432457	-0.790986

P	1.012559	-0.387367	0.024094

O	-1.336824	-1.675030	-0.678672

N	-0.501965	0.432019	0.011423

C	-1.460732	-0.414082	-0.331527

C	-2.877821	0.090276	-0.314137

C	-3.157389	1.428376	0.024799

C	-4.485761	1.878547	0.072400

C	-5.547468	1.008914	-0.228291

C	-5.265908	-0.320953	-0.578022

C	-3.940327	-0.781473	-0.617928

C	1.432370	-0.227334	1.823212

C	1.551773	-1.374877	2.643278

C	1.631521	-1.282374	4.044468

H	1.731163	-2.191927	4.643372

C	1.593697	-0.019700	4.649445

H	1.661537	0.059053	5.740049

C	1.503899	1.144152	3.874806

H	1.501649	2.135716	4.337034

C	1.433592	1.030308	2.476121

C	2.212271	0.710759	-0.854849

C	3.575889	0.795391	-0.477122

C	4.478223	1.652895	-1.128488

H	5.520708	1.694368	-0.800880

C	4.025673	2.429422	-2.205290

H	4.724607	3.096532	-2.721840

C	2.706104	2.319273	-2.660462

H	2.355370	2.880715	-3.531450

C	1.823311	1.442451	-2.003929

C	2.374814	-3.131605	-1.356571

H	3.005272	-4.030921	-1.411489

H	2.734233	-2.560121	-0.490186

C	2.465589	-2.386783	-2.691000

H	1.526497	-1.852098	-2.951344

H	3.219018	-1.574486	-2.620747

C	2.854854	-3.267352	-3.882978

C	2.855818	-2.586800	-5.247701

H	3.339641	-1.594103	-5.207338

H	3.359941	-3.226751	-5.988086

H	1.811108	-2.419488	-5.574589

O	3.139402	-4.458184	-3.758001

C	0.922572	-4.538037	-1.267010

H	1.298702	-4.924018	-2.221935

H	1.319983	-5.069866	-0.392828

C	-0.447955	-4.124495	-1.213159

H	-1.036992	-4.305120	-0.304578

H	-1.025685	-4.018921	-2.140703

C	-6.390483	-1.251482	-0.970986

C	-4.779104	3.291286	0.520778

F	-6.573883	-1.272024	-2.324336

F	-6.142179	-2.535604	-0.586292

F	-3.831176	4.171181	0.098955

F	-4.817897	3.381008	1.890435

F	-5.986034	3.734381	0.067262

F	-7.579128	-0.878431	-0.414705

H	-6.580710	1.363601	-0.190329

H	-3.713125	-1.818672	-0.876791

H	-2.323291	2.100969	0.244359

O	1.453670	2.160921	1.672676

O	1.656366	-2.604187	2.002747

O	0.571574	1.177572	-2.535788

O	3.994338	-0.084457	0.513870

C	0.657767	3.255975	2.003099

C	-0.672464	3.107870	2.434285

C	1.214230	4.529325	1.798646

C	-1.447112	4.255282	2.663111

C	0.426743	5.668145	2.033303

C	-0.902270	5.536583	2.467736

H	-1.091161	2.104991	2.557904

H	2.250273	4.611551	1.453959

H	-2.493730	4.141735	2.963414

H	0.859760	6.662621	1.875948

H	-1.515158	6.426916	2.644755

C	1.114512	-3.740259	2.604495

C	-0.203404	-3.762119	3.096253

C	1.908857	-4.898612	2.613578

C	-0.719494	-4.962535	3.608235

C	1.374523	-6.094609	3.120622

C	0.062936	-6.130640	3.622071

H	-0.810469	-2.851900	3.069582

H	2.931938	-4.847024	2.227134

H	-1.747065	-4.984835	3.988512

H	1.992309	-6.999682	3.128000

H	-0.349589	-7.064322	4.019036

C	-0.232325	2.235569	-2.957030

C	-0.986555	2.033419	-4.124019

C	-0.350674	3.422066	-2.212329

C	-1.866764	3.038364	-4.554188

C	-1.230709	4.419222	-2.659695

C	-1.988615	4.235245	-3.828960

H	-0.878023	1.093209	-4.674448

H	0.222272	3.546165	-1.288524

H	-2.457817	2.882316	-5.463782

H	-1.334223	5.340817	-2.076232

H	-2.676690	5.017247	-4.167789

C	5.034675	0.277181	1.371006

C	6.027565	-0.685341	1.611467

C	5.048371	1.516651	2.034354

C	7.051889	-0.399269	2.527676

C	6.083889	1.790871	2.940397

C	7.087287	0.838734	3.190347

H	5.983164	-1.642422	1.081959

H	4.248390	2.239291	1.846093

H	7.828502	-1.148555	2.718255

H	6.098202	2.754025	3.463246

H	7.890409	1.059673	3.901688

**1-beta-C**

SCF Done: -3582.44919050 A.U.

Ni	0.379914	2.195177	0.439632

P	0.792720	0.015243	0.282456

O	-1.332500	1.774208	-0.184205

N	-0.723331	-0.518405	-0.328756

C	-1.561161	0.499219	-0.435510

C	-2.965336	0.198638	-0.879640

C	-3.916119	1.232295	-0.974793

C	-5.232501	0.945066	-1.369027

C	-5.616652	-0.372706	-1.664335

C	-4.665541	-1.402241	-1.570530

C	-3.344865	-1.121832	-1.187728

C	2.160461	-0.618826	-0.789017

C	3.483646	-0.358592	-0.341998

C	4.617305	-0.741832	-1.074988

H	5.618705	-0.539269	-0.687591

C	4.432292	-1.395898	-2.300800

H	5.307129	-1.710920	-2.880433

C	3.149174	-1.669801	-2.785820

H	3.007391	-2.198265	-3.732408

C	2.020921	-1.284864	-2.033356

C	0.928053	-0.978141	1.835243

C	0.822873	-2.389404	1.801439

C	0.752048	-3.157783	2.977320

H	0.666932	-4.246197	2.908540

C	0.811910	-2.506285	4.215117

H	0.771077	-3.094807	5.138526

C	0.947536	-1.112549	4.294865

H	1.014422	-0.610346	5.263636

C	1.001101	-0.360066	3.107507

C	0.136146	4.097282	0.658897

H	-0.539527	4.293782	1.502886

H	-0.160784	4.581231	-0.281089

C	1.574572	3.908318	0.946921

H	1.841707	4.132806	1.996054

C	2.612669	4.458308	-0.052026

H	2.641373	5.559786	0.048605

H	3.618022	4.087308	0.214210

H	1.853557	2.707890	0.975915

H	-6.644346	-0.595063	-1.962985

H	-2.591774	-1.912559	-1.120095

H	-3.612129	2.253217	-0.730054

C	-5.077819	-2.835104	-1.816775

C	-6.226072	2.072666	-1.531731

F	-5.346558	-3.485870	-0.639623

F	-6.204661	-2.922540	-2.579116

F	-4.100565	-3.546874	-2.442322

F	-7.512712	1.643511	-1.383782

F	-6.137577	2.643448	-2.769572

F	-6.017454	3.066427	-0.622260

C	2.322938	4.070145	-1.511289

C	3.313011	4.262396	-3.901263

H	4.226414	4.046347	-4.476128

H	2.480340	3.626569	-4.255866

H	3.007696	5.311183	-4.083102

O	1.173164	1.016926	3.121138

O	0.875774	-2.991167	0.558355

O	0.744726	-1.616188	-2.436125

O	3.560365	0.254314	0.899594

C	0.856328	1.742406	4.271693

C	1.879998	2.499182	4.863355

C	-0.462178	1.795565	4.757431

C	1.575367	3.326076	5.957708

C	-0.749341	2.619842	5.856265

C	0.264295	3.386868	6.457785

H	2.896031	2.427311	4.461489

H	-1.244506	1.203423	4.271826

H	2.370242	3.920481	6.422204

H	-1.775700	2.670043	6.237038

H	0.030705	4.031983	7.311635

C	0.010227	-4.040875	0.262757

C	-1.360415	-3.980101	0.570685

C	0.546879	-5.129684	-0.442775

C	-2.199060	-5.027295	0.159150

C	-0.304470	-6.171442	-0.845446

C	-1.676339	-6.125644	-0.546129

H	-1.756644	-3.106839	1.097272

H	1.617198	-5.140877	-0.671957

H	-3.272887	-4.964655	0.363854

H	0.111379	-7.023385	-1.395522

H	-2.337986	-6.937225	-0.867692

C	0.432385	-1.753203	-3.783851

C	-0.396698	-2.834212	-4.127366

C	0.828567	-0.811891	-4.752089

C	-0.829694	-2.972790	-5.454791

C	0.394133	-0.969789	-6.077071

C	-0.434202	-2.047062	-6.434866

H	-0.694479	-3.542452	-3.348028

H	1.466852	0.029856	-4.465374

H	-1.479657	-3.813596	-5.722074

H	0.699946	-0.237030	-6.832862

H	-0.772747	-2.161029	-7.470392

C	4.768452	0.767375	1.379352

C	5.451194	1.798628	0.710384

C	5.217802	0.279540	2.616967

C	6.607937	2.336002	1.298837

C	6.371403	0.834085	3.194820

C	7.071253	1.859563	2.537194

H	5.088036	2.185852	-0.247877

H	4.656101	-0.520583	3.109174

H	7.141547	3.141025	0.781279

H	6.725002	0.456769	4.161143

H	7.972838	2.287600	2.988998

H	1.906780	3.033160	-1.531077

H	1.536620	4.701050	-1.965561

C	3.561355	4.052974	-2.413339

O	4.692167	3.853984	-1.970213

### **5.5.3 Geometries of Ni-9**

**1-cycle5-T**

SCF Done: -3463.47551075 A.U.

C	-1.391139	1.298202	1.126398

C	-1.624463	2.691481	0.980285

C	-1.819280	3.535783	2.084738

H	-2.004909	4.602078	1.931875

C	-1.768343	2.988420	3.374987

H	-1.910686	3.641409	4.243348

C	-1.561302	1.619500	3.566638

H	-1.542193	1.184835	4.569779

C	-1.394778	0.782351	2.446492

C	-2.129832	0.360914	-1.651044

C	-3.493044	0.696785	-1.458083

C	-4.399034	0.772522	-2.532319

H	-5.435803	1.063793	-2.342586

C	-3.954992	0.472917	-3.825093

H	-4.651568	0.546699	-4.667611

C	-2.632268	0.074057	-4.056877

H	-2.288318	-0.178717	-5.062850

C	-1.741975	-0.005939	-2.971604

C	0.703332	1.021278	-0.845273

C	0.905461	2.145657	-1.659454

H	0.047876	2.664151	-2.099124

C	2.211463	2.591250	-1.926145

H	2.378036	3.463904	-2.566557

C	3.303389	1.917953	-1.347804

H	4.322050	2.275877	-1.538295

C	3.128208	0.791972	-0.525442

C	1.802196	0.278979	-0.295957

C	-0.288594	-4.233096	1.181737

Ni	-0.143230	-1.674705	0.277909

O	1.612434	-0.841507	0.369437

P	-0.868414	0.246750	-0.308381

C	0.114763	-5.600390	1.669724

C	-1.726976	-3.811237	1.055549

C	-1.809677	-2.628811	0.066768

H	-2.385653	-4.676732	0.823546

H	-2.028264	-3.470742	2.070250

H	-1.780854	-2.986525	-0.982902

H	-2.736289	-2.050097	0.215161

O	0.589506	-3.389918	0.875160

C	4.285246	0.073739	0.065895

C	4.367885	-0.175491	1.450483

C	5.344601	-0.422757	-0.720388

C	5.414808	-0.907629	2.024193

C	6.417071	-1.140512	-0.169671

C	6.449750	-1.390436	1.210001

F	5.341499	-0.237123	-2.060027

F	7.408250	-1.606390	-0.958865

F	7.471870	-2.083652	1.749975

F	5.444596	-1.130343	3.357394

F	3.431812	0.324913	2.287988

H	1.206700	-5.663520	1.791502

H	-0.385429	-5.829532	2.629248

H	-0.220770	-6.368893	0.946813

O	-0.434300	-0.417176	-3.104687

O	-3.890968	1.017171	-0.170881

O	-1.703009	3.152407	-0.321511

O	-1.291457	-0.590920	2.613826

C	0.029168	-1.003702	-4.284486

C	1.141542	-0.406666	-4.896318

C	-0.518379	-2.202222	-4.774198

C	1.707674	-1.017824	-6.026202

H	1.554584	0.511361	-4.466401

C	0.053406	-2.795641	-5.910510

H	-1.372865	-2.659440	-4.265153

C	1.163772	-2.206940	-6.539692

H	2.581057	-0.560450	-6.504337

H	-0.366505	-3.731029	-6.297806

H	1.608800	-2.679414	-7.422137

C	-5.158655	0.675866	0.289537

C	-5.791055	-0.538289	-0.037116

C	-5.750786	1.575973	1.191908

C	-7.034870	-0.835994	0.541158

H	-5.313197	-1.235986	-0.731521

C	-6.990516	1.259075	1.766554

H	-5.223108	2.502814	1.437782

C	-7.640928	0.056944	1.440866

H	-7.528795	-1.781376	0.288491

H	-7.450793	1.960982	2.471175

H	-8.611395	-0.184534	1.887691

C	-1.566546	4.517192	-0.589528

C	-0.384117	5.208408	-0.272034

C	-2.613489	5.145534	-1.280530

C	-0.265311	6.556089	-0.644607

H	0.427879	4.686005	0.242860

C	-2.476224	6.492342	-1.654198

H	-3.512760	4.570236	-1.521888

C	-1.307155	7.201586	-1.333595

H	0.654647	7.100619	-0.403541

H	-3.289864	6.987192	-2.196360

H	-1.204345	8.252640	-1.624808

C	-0.570785	-1.066275	3.721942

C	-1.266167	-1.699794	4.763234

C	0.830406	-0.988712	3.713063

C	-0.533927	-2.255301	5.828116

H	-2.360088	-1.748413	4.732792

C	1.546917	-1.545127	4.783424

H	1.342004	-0.538525	2.857421

C	0.869030	-2.175259	5.842792

H	-1.066084	-2.748817	6.649620

H	2.641418	-1.492574	4.770450

H	1.433301	-2.609550	6.675670

**TS-1-Coor-CO-T**

SCF Done: -3576.77704325 A.U.

C	-1.846623	3.317075	1.173355

C	-0.825270	4.184510	1.641030

C	-1.116789	5.322525	2.417911

H	-0.300842	5.963342	2.762005

C	-2.445302	5.597747	2.757169

H	-2.678585	6.484012	3.357620

C	-3.480002	4.735219	2.367881

H	-4.515986	4.932903	2.655229

C	-3.168191	3.590415	1.615532

C	-2.806360	1.591626	-1.068222

C	-3.367190	2.661309	-1.811762

C	-4.286848	2.441981	-2.852293

H	-4.717963	3.291950	-3.387573

C	-4.617384	1.126358	-3.200104

H	-5.315392	0.945837	-4.025265

C	-4.072933	0.038355	-2.509245

H	-4.323829	-0.986205	-2.792675

C	-3.193680	0.277080	-1.436618

C	-0.000956	2.077574	-0.898945

C	0.191084	3.134248	-1.804800

H	-0.510962	3.973874	-1.819794

C	1.292055	3.122575	-2.673587

H	1.462918	3.956870	-3.361889

C	2.191586	2.043197	-2.634409

H	3.056865	2.035312	-3.306939

C	2.028561	0.976168	-1.734356

C	0.897771	0.964260	-0.844312

C	-2.582699	-1.976243	2.367002

Ni	-0.520729	0.185076	1.470049

O	0.688671	-0.066744	-0.046001

P	-1.449272	1.805977	0.189583

C	-3.567515	-3.089585	2.689432

C	-2.997528	-0.545903	2.690128

C	-1.805194	0.372069	2.975806

H	-3.564727	-0.188935	1.805644

H	-3.724322	-0.545647	3.531567

H	-2.099471	1.432416	3.031675

H	-1.341563	0.098169	3.939183

O	-1.488881	-2.217792	1.849291

C	2.992290	-0.153499	-1.703586

C	3.624969	-0.560529	-0.510082

C	3.323113	-0.878160	-2.867221

C	4.517631	-1.640838	-0.467329

C	4.222588	-1.954229	-2.853225

C	4.820602	-2.340021	-1.645179

F	2.751004	-0.560420	-4.052072

F	4.505136	-2.627463	-3.988863

F	5.686198	-3.372354	-1.617245

F	5.104957	-1.998669	0.693068

F	3.408061	0.111139	0.638043

H	-4.589657	-2.819584	2.365564

H	-3.253553	-4.030100	2.212289

H	-3.610375	-3.233750	3.786715

C	0.728127	-0.244775	2.656753

O	1.546351	-0.418118	3.463539

O	-2.660990	-0.747730	-0.673364

O	-2.998390	3.939987	-1.425934

O	0.473867	3.794120	1.389860

O	-4.138124	2.687293	1.208630

C	-5.367583	2.609116	1.862256

C	-6.513288	2.591449	1.050879

C	-5.463802	2.443697	3.256179

C	-7.772181	2.409606	1.644980

H	-6.398849	2.708856	-0.031334

C	-6.730434	2.272098	3.835795

H	-4.557998	2.449965	3.870535

C	-7.886515	2.254627	3.036388

H	-8.667711	2.393208	1.013657

H	-6.810594	2.143465	4.921258

H	-8.871022	2.117506	3.496526

C	1.494721	4.737200	1.275863

C	2.719434	4.410475	1.879022

C	1.348475	5.914931	0.521767

C	3.810072	5.280378	1.729712

H	2.801938	3.478405	2.446549

C	2.446417	6.779074	0.390136

H	0.390815	6.144330	0.044323

C	3.677780	6.468331	0.991364

H	4.767926	5.025694	2.196992

H	2.337605	7.697462	-0.198163

H	4.531127	7.145986	0.880028

C	-2.612845	-2.053238	-1.188317

C	-1.370938	-2.530694	-1.629636

C	-3.748263	-2.878492	-1.154615

C	-1.275990	-3.859122	-2.072485

H	-0.499935	-1.870011	-1.574521

C	-3.638438	-4.204974	-1.605894

H	-4.698154	-2.484712	-0.777317

C	-2.406264	-4.694866	-2.069343

H	-0.308371	-4.244986	-2.412108

H	-4.519625	-4.856515	-1.587977

H	-2.324245	-5.730854	-2.416182

C	-3.113568	5.015142	-2.307887

C	-2.571460	4.978950	-3.605930

C	-3.699877	6.185444	-1.800227

C	-2.637301	6.132727	-4.402418

H	-2.102911	4.061919	-3.976618

C	-3.750358	7.333176	-2.606819

H	-4.094660	6.180841	-0.779338

C	-3.225698	7.310225	-3.909709

H	-2.215525	6.110101	-5.413641

H	-4.206038	8.248481	-2.212591

H	-3.270166	8.206851	-4.537312

**1-Coor-CO-T**

SCF Done: -3576.78577474 A.U.

C	1.384020	-0.655831	-1.531202

C	2.373780	0.037624	-2.268710

C	2.844946	-0.432736	-3.505705

H	3.625715	0.116181	-4.038571

C	2.294280	-1.606044	-4.036932

H	2.647835	-1.976116	-5.005683

C	1.298564	-2.314620	-3.354152

H	0.862305	-3.221161	-3.779959

C	0.855424	-1.840283	-2.106179

C	1.973080	0.557980	1.170879

C	3.263064	-0.037607	1.212384

C	4.256790	0.420640	2.095892

H	5.241552	-0.052651	2.096413

C	3.957274	1.467963	2.976513

H	4.732406	1.834478	3.658809

C	2.679983	2.039424	3.018105

H	2.441978	2.839449	3.723151

C	1.690135	1.558757	2.141324

C	-0.354024	1.412384	-0.575643

C	0.091038	2.681033	-0.979726

H	1.147108	2.949159	-0.873782

C	-0.825668	3.606600	-1.502868

H	-0.491583	4.602175	-1.813441

C	-2.175592	3.235753	-1.645149

H	-2.889559	3.948545	-2.073696

C	-2.641157	1.966257	-1.262738

C	-1.728619	1.024787	-0.667975

Ni	-0.972474	-1.341373	0.752819

O	-2.166904	-0.137480	-0.222907

P	0.643296	0.008728	0.027085

C	0.334548	-2.482176	1.745463

H	-0.008534	-3.522498	1.603479

C	0.416961	-2.117933	3.224714

H	-0.556910	-2.230830	3.740392

H	1.321572	-2.390134	1.266719

C	1.475633	-2.901836	4.020111

O	2.429940	-3.467150	3.490351

H	0.683988	-1.045458	3.347289

C	1.276755	-2.939487	5.533833

H	2.177848	-3.335528	6.027209

H	1.034992	-1.936175	5.930911

H	0.416079	-3.592401	5.776414

C	-4.066778	1.585516	-1.425784

C	-4.443799	0.414502	-2.114499

C	-5.115118	2.364553	-0.895358

C	-5.781146	0.018286	-2.246677

C	-6.463176	1.998994	-1.026203

C	-6.797919	0.816804	-1.702069

F	-3.504441	-0.355498	-2.708180

F	-6.099270	-1.111009	-2.914467

F	-8.088485	0.453559	-1.833819

F	-7.436936	2.768121	-0.496334

F	-4.840002	3.498869	-0.212191

C	-2.294400	-2.208525	1.561259

O	-3.146042	-2.742249	2.145544

O	0.394439	2.030225	2.141664

O	3.470380	-1.091792	0.349997

O	-0.140770	-2.477359	-1.387311

O	2.900194	1.166770	-1.662235

C	-0.719783	-3.661792	-1.869287

C	-0.026770	-4.878484	-1.766615

C	-2.037142	-3.599536	-2.345888

C	-0.671773	-6.059793	-2.167638

H	0.994688	-4.891396	-1.372803

C	-2.669359	-4.791593	-2.736704

H	-2.548300	-2.633132	-2.391374

C	-1.990453	-6.019033	-2.652554

H	-0.142601	-7.016251	-2.089798

H	-3.700270	-4.756728	-3.106246

H	-2.490436	-6.944735	-2.957843

C	3.477951	2.180094	-2.429323

C	4.700758	2.696501	-1.971814

C	2.825251	2.737723	-3.543449

C	5.284857	3.778775	-2.648817

H	5.167018	2.253834	-1.085685

C	3.426772	3.813570	-4.214270

H	1.859396	2.337620	-3.866474

C	4.655215	4.336040	-3.773870

H	6.238314	4.185023	-2.293006

H	2.922147	4.252695	-5.082310

H	5.115427	5.178170	-4.301855

C	-0.071492	2.882275	3.146536

C	-0.113482	2.477397	4.491898

C	-0.605310	4.114475	2.740592

C	-0.684623	3.336300	5.443805

H	0.291281	1.502224	4.780855

C	-1.181653	4.958563	3.702955

H	-0.580371	4.380258	1.679103

C	-1.217753	4.576928	5.054339

H	-0.722154	3.026621	6.494416

H	-1.606934	5.918858	3.390695

H	-1.668096	5.239451	5.801401

C	4.696777	-1.774482	0.370103

C	5.695419	-1.399464	-0.540726

C	4.853723	-2.870434	1.231724

C	6.887327	-2.141118	-0.584243

H	5.523511	-0.549210	-1.208248

C	6.052785	-3.600197	1.175603

H	4.044418	-3.141145	1.919997

C	7.068566	-3.239128	0.273626

H	7.672881	-1.861093	-1.295213

H	6.188070	-4.460487	1.840816

H	7.999017	-3.816526	0.234931

**TS-1-Ins-CO-T**

SCF Done: -3576.76514759 A.U.

C	2.432669	0.946632	2.579986

C	1.882720	1.686016	3.657486

C	2.686860	2.513543	4.465967

H	2.228265	3.067860	5.289249

C	4.053743	2.633106	4.189790

H	4.675663	3.286380	4.811840

C	4.627380	1.964910	3.098873

H	5.684861	2.086217	2.849139

C	3.809203	1.149385	2.301246

C	2.432849	-1.717974	1.310550

C	2.982397	-2.403778	2.421507

C	3.666589	-3.623559	2.278471

H	4.098591	-4.113139	3.155361

C	3.781435	-4.193427	1.004152

H	4.302286	-5.150014	0.886283

C	3.240110	-3.561066	-0.121218

H	3.318285	-4.013107	-1.113120

C	2.582903	-2.328171	0.038888

C	-0.151167	-0.634589	2.064302

C	-0.428598	-1.276600	3.285812

H	0.379752	-1.442027	4.004768

C	-1.732025	-1.684602	3.591751

H	-1.951035	-2.171606	4.547526

C	-2.764662	-1.438794	2.668683

H	-3.789913	-1.742758	2.908942

C	-2.524927	-0.795436	1.444329

C	-1.185272	-0.398234	1.100322

Ni	0.742295	0.747715	-0.569227

O	-0.966388	0.141148	-0.090379

P	1.479027	-0.134527	1.424460

C	2.276742	1.837458	-1.627984

H	2.840867	1.208298	-0.915456

C	2.491109	3.321443	-1.334381

H	2.235967	3.554771	-0.281283

H	2.620363	1.542398	-2.631791

C	3.927854	3.795715	-1.590340

O	4.827677	3.026540	-1.921265

H	1.813022	3.951629	-1.945367

C	4.176093	5.291651	-1.425347

H	3.822367	5.644364	-0.438723

H	5.249267	5.508682	-1.537677

H	3.607197	5.857089	-2.187733

C	-3.630308	-0.540355	0.484821

C	-4.469742	-1.577514	0.030020

C	-3.894935	0.750452	-0.018936

C	-5.511328	-1.355590	-0.882621

C	-4.922150	0.995838	-0.941150

C	-5.736391	-0.061553	-1.373256

F	-4.273990	-2.847085	0.455999

F	-6.288161	-2.377818	-1.299839

F	-6.733665	0.165948	-2.250478

F	-5.147542	2.244423	-1.399618

F	-3.171117	1.806196	0.401323

C	0.492121	1.685021	-2.003522

O	0.028042	2.280115	-2.919430

O	1.980453	-1.679476	-1.028326

O	2.884343	-1.763412	3.650112

O	0.513214	1.631999	3.819710

O	4.277837	0.553128	1.138572

C	2.392315	-1.947898	-2.339933

C	1.394641	-2.293382	-3.263637

C	3.729653	-1.772528	-2.736171

C	1.746448	-2.470590	-4.611641

H	0.362509	-2.408008	-2.918097

C	4.065498	-1.962342	-4.086039

H	4.487422	-1.485976	-1.999480

C	3.079443	-2.310758	-5.025406

H	0.971416	-2.735684	-5.339302

H	5.104869	-1.824198	-4.404681

H	3.348920	-2.450732	-6.077809

C	2.897092	-2.494784	4.837881

C	2.099930	-3.638838	5.025378

C	3.666775	-1.969028	5.888948

C	2.094649	-4.265388	6.281196

H	1.492331	-4.022916	4.200169

C	3.643516	-2.603129	7.141330

H	4.264383	-1.068901	5.711660

C	2.863668	-3.754292	7.340791

H	1.474083	-5.155905	6.431939

H	4.243782	-2.194999	7.962245

H	2.851109	-4.248832	8.317961

C	-0.059660	1.796843	5.079625

C	-1.211512	2.596513	5.146720

C	0.423977	1.124942	6.216619

C	-1.882811	2.730265	6.371563

H	-1.569290	3.091190	4.238323

C	-0.254202	1.275946	7.435963

H	1.311982	0.489121	6.141471

C	-1.405611	2.077093	7.520246

H	-2.783770	3.351657	6.425414

H	0.117375	0.751540	8.323785

H	-1.931306	2.186572	8.474979

C	5.622454	0.182774	1.027373

C	6.292837	0.559865	-0.146647

C	6.245532	-0.608956	2.007370

C	7.616691	0.129461	-0.336929

H	5.781025	1.191043	-0.881667

C	7.571085	-1.021294	1.803425

H	5.690656	-0.900097	2.904504

C	8.259893	-0.656539	0.633539

H	8.148499	0.423007	-1.249052

H	8.063364	-1.641606	2.561141

H	9.293710	-0.984810	0.480048

1-cycle6-C

**SCF Done: -3576.78151123 A.U.**

C	-1.923237	0.816453	0.835715

C	-1.512856	1.715639	1.860275

C	-2.384233	2.101767	2.895363

H	-2.031523	2.789749	3.667838

C	-3.691338	1.599432	2.922931

H	-4.375214	1.914895	3.718559

C	-4.130192	0.696355	1.947402

H	-5.148261	0.296983	1.959084

C	-3.232720	0.293435	0.944454

C	-1.600122	-0.047198	-2.000894

C	-2.566752	0.860137	-2.505540

C	-3.212112	0.644851	-3.735923

H	-3.947786	1.368240	-4.097734

C	-2.877562	-0.483959	-4.497373

H	-3.362480	-0.642544	-5.467049

C	-1.904634	-1.388147	-4.054903

H	-1.613161	-2.249305	-4.662355

C	-1.279746	-1.158243	-2.816186

C	0.632456	1.296452	-0.786107

C	0.540862	2.417532	-1.629250

H	-0.432676	2.706263	-2.038641

C	1.684380	3.168475	-1.935241

H	1.614938	4.044641	-2.588672

C	2.927210	2.776326	-1.409705

H	3.827532	3.347850	-1.662363

C	3.056077	1.649595	-0.579404

C	1.885682	0.895106	-0.222317

C	1.618425	-2.036692	2.340990

Ni	0.600707	-1.314543	0.935014

O	2.008842	-0.121312	0.616220

P	-0.708907	0.126226	-0.386479

C	1.897602	-3.557580	2.278151

C	1.122683	-4.309768	1.175686

H	1.241768	-5.401190	1.287084

H	1.685448	-3.986696	3.276261

C	-0.342138	-3.925594	1.166988

O	-0.670606	-2.711088	1.140210

O	1.998663	-1.374145	3.290655

H	1.527183	-4.033410	0.180646

C	-1.407313	-4.984524	1.183769

H	-1.281718	-5.635416	2.070860

H	-2.412366	-4.535912	1.184310

H	-1.290272	-5.633115	0.293622

C	4.380756	1.239473	-0.047594

C	4.874223	-0.073400	-0.199469

C	5.214913	2.143764	0.642351

C	6.108523	-0.475713	0.328820

C	6.461566	1.769893	1.165645

C	6.909680	0.450477	1.011962

F	4.171043	-0.993558	-0.892932

F	6.541823	-1.745414	0.164952

F	8.104567	0.076170	1.511728

F	7.224890	2.665717	1.826843

F	4.816033	3.420310	0.841276

H	2.984566	-3.661957	2.105626

O	-0.235778	-1.963672	-2.376229

O	-2.786782	2.002630	-1.754547

O	-3.632273	-0.595097	-0.056087

O	-0.213171	2.158141	1.779275

C	-3.972236	2.725494	-1.905781

C	-3.861775	4.096017	-2.185458

C	-5.225639	2.126780	-1.690455

C	-5.026333	4.877506	-2.254526

H	-2.869766	4.533075	-2.337331

C	-6.381017	2.918471	-1.771966

H	-5.279527	1.061259	-1.448233

C	-6.287245	4.292437	-2.054029

H	-4.943834	5.948673	-2.470182

H	-7.360946	2.458057	-1.601666

H	-7.193537	4.905056	-2.111475

C	-0.250569	-3.332296	-2.601727

C	-1.420845	-4.110919	-2.517065

C	0.999465	-3.934888	-2.836712

C	-1.328503	-5.501975	-2.690390

H	-2.384501	-3.632737	-2.315528

C	1.074635	-5.327332	-2.994953

H	1.889687	-3.300364	-2.895680

C	-0.086696	-6.117029	-2.927978

H	-2.240337	-6.108261	-2.634650

H	2.048173	-5.794334	-3.182439

H	-0.025268	-7.202129	-3.064102

C	-4.384159	-1.723519	0.231993

C	-4.390840	-2.370624	1.482585

C	-5.115525	-2.252079	-0.849617

C	-5.157342	-3.537242	1.643306

H	-3.809226	-1.963370	2.313986

C	-5.866456	-3.423283	-0.673312

H	-5.076953	-1.736198	-1.814992

C	-5.897904	-4.069660	0.574354

H	-5.171270	-4.033287	2.621052

H	-6.437071	-3.826831	-1.517643

H	-6.495113	-4.977207	0.713182

C	0.367304	2.847924	2.851407

C	0.164460	4.230048	2.990191

C	1.230799	2.140287	3.701042

C	0.828957	4.914057	4.021249

H	-0.494919	4.753455	2.290001

C	1.890444	2.840717	4.724125

H	1.397692	1.069837	3.534872

C	1.688822	4.221617	4.890455

H	0.680230	5.993764	4.136924

H	2.573877	2.300519	5.389007

H	2.210506	4.760718	5.689135

**TS-2-Coor-E-C**

SCF Done: -3655.33484117 A.U.

C	-2.175417	3.006053	1.283773

C	-1.388600	4.119609	1.672308

C	-1.865983	5.091810	2.573826

H	-1.215434	5.924185	2.856071

C	-3.156847	4.974922	3.098805

H	-3.532650	5.733649	3.794146

C	-3.967129	3.882206	2.757405

H	-4.978377	3.781355	3.160942

C	-3.459299	2.908529	1.882547

C	-2.939919	1.497716	-1.119774

C	-3.691760	2.591634	-1.623775

C	-4.702958	2.423243	-2.587899

H	-5.284744	3.287759	-2.919079

C	-4.935386	1.151342	-3.124430

H	-5.706663	1.018912	-3.891340

C	-4.177119	0.051987	-2.706259

H	-4.323336	-0.935209	-3.152296

C	-3.202920	0.235323	-1.708270

C	-0.144770	2.050252	-0.779253

C	-0.080206	3.079852	-1.739018

H	-0.943875	3.739775	-1.877112

C	1.076772	3.280088	-2.499095

H	1.126060	4.087713	-3.236758

C	2.188324	2.442120	-2.287943

H	3.104975	2.599963	-2.868465

C	2.163869	1.412742	-1.337093

C	0.965018	1.164476	-0.570335

C	0.410139	-1.753518	1.914390

Ni	-0.592355	-0.256929	1.376833

O	0.947140	0.142645	0.257204

P	-1.616867	1.600684	0.196404

C	-0.248579	-3.085661	1.505112

H	-0.518762	-0.117970	4.377537

C	-1.491967	-3.368958	2.363987

H	-1.201677	-3.527838	3.425210

H	0.490809	-3.896093	1.636965

C	-2.531257	-2.263338	2.338272

O	-2.264422	-1.103176	1.950569

O	1.468359	-1.712209	2.511228

H	-1.975963	-4.312071	2.045400

C	-3.929296	-2.592980	2.801312

H	-3.902503	-3.171316	3.744253

H	-4.523948	-1.677060	2.938572

H	-4.420788	-3.236435	2.045588

C	3.349104	0.543448	-1.120293

C	3.942611	-0.181472	-2.172964

C	3.937504	0.397271	0.153101

C	5.053973	-1.015055	-1.978478

C	5.040869	-0.437839	0.376585

C	5.603640	-1.146408	-0.695522

F	3.430135	-0.107685	-3.423774

F	5.588693	-1.700294	-3.012741

F	6.671852	-1.944562	-0.494649

F	5.580279	-0.548936	1.608260

F	3.465035	1.098290	1.203980

C	0.499188	1.223987	3.054412

C	-0.561477	0.806270	3.789140

H	1.428762	0.649046	3.013793

H	0.487649	2.181316	2.522561

H	-0.538569	-3.032400	0.440972

H	-1.485150	1.393204	3.853106

O	-2.368184	-0.803655	-1.319749

O	-3.431477	3.836058	-1.071977

O	-0.082078	4.163837	1.216947

O	-4.226773	1.828113	1.462163

C	0.541407	5.383540	0.963833

C	-0.113707	6.444202	0.312487

C	1.899371	5.475788	1.309726

C	0.606372	7.613749	0.024053

H	-1.167372	6.349564	0.031670

C	2.606299	6.648863	1.006506

H	2.383938	4.624661	1.798019

C	1.964013	7.723660	0.369055

H	0.099356	8.440879	-0.486108

H	3.666694	6.720579	1.273708

H	2.519212	8.639058	0.137197

C	-5.246993	1.311930	2.247712

C	-6.415150	0.921470	1.569038

C	-5.113630	1.093713	3.632400

C	-7.457928	0.312022	2.283462

H	-6.484348	1.103845	0.491835

C	-6.170798	0.492923	4.334777

H	-4.194754	1.391870	4.146006

C	-7.344170	0.099462	3.667911

H	-8.368674	0.010721	1.753404

H	-6.069873	0.326400	5.413543

H	-8.163754	-0.367904	4.224290

C	-2.787372	-2.121964	-1.424344

C	-1.862954	-3.038753	-1.955409

C	-4.037787	-2.556609	-0.943115

C	-2.194556	-4.402801	-2.002845

H	-0.901048	-2.666961	-2.323028

C	-4.359098	-3.921978	-1.009745

H	-4.743178	-1.828618	-0.530198

C	-3.441671	-4.850013	-1.534581

H	-1.474141	-5.116492	-2.418103

H	-5.336685	-4.261706	-0.647342

H	-3.700379	-5.913223	-1.583224

C	-3.698012	5.008041	-1.774560

C	-3.319649	5.189143	-3.118150

C	-4.269021	6.059209	-1.037165

C	-3.532254	6.437517	-3.723058

H	-2.864325	4.363949	-3.674560

C	-4.467403	7.303810	-1.655411

H	-4.538587	5.884949	0.009494

C	-4.105914	7.496827	-2.999176

H	-3.236475	6.582618	-4.768302

H	-4.912091	8.124539	-1.081274

H	-4.265861	8.468279	-3.479254

**2-Coor-E-C**

SCF Done: -3655.35216218 A.U.

C	2.136783	0.541457	-1.263911

C	1.881782	1.781594	-1.901249

C	2.684634	2.248180	-2.961777

H	2.450812	3.209186	-3.427863

C	3.745833	1.461381	-3.418617

H	4.362993	1.814241	-4.252286

C	4.003811	0.202899	-2.852367

H	4.808325	-0.432339	-3.231615

C	3.189724	-0.246906	-1.802298

C	2.080210	-0.834462	1.408678

C	3.394219	-0.437911	1.750326

C	4.101882	-1.039131	2.806737

H	5.124683	-0.715340	3.021013

C	3.482759	-2.038767	3.567539

H	4.033505	-2.519707	4.383248

C	2.167812	-2.436080	3.291182

H	1.673325	-3.208539	3.886370

C	1.476345	-1.823987	2.231886

C	0.004342	1.056186	0.832021

C	0.471755	2.060507	1.696488

H	1.548462	2.167613	1.874902

C	-0.431503	2.930887	2.321284

H	-0.072603	3.720511	2.989339

C	-1.805234	2.788426	2.066382

H	-2.517750	3.470370	2.544318

C	-2.301549	1.796456	1.201526

C	-1.390783	0.879389	0.570405

C	-2.388358	-2.250992	-1.487881

Ni	-0.749638	-1.477406	-0.919483

O	-1.860659	-0.087721	-0.205157

P	1.030118	-0.207931	0.014527

C	-3.157438	-1.440895	-2.529755

H	-3.459724	-0.504269	-2.029903

C	-4.352050	-2.201601	-3.113567

H	-4.968001	-1.521925	-3.737695

H	-2.447768	-1.147909	-3.322974

C	-3.935211	-3.385917	-3.991463

O	-2.767278	-3.566109	-4.332834

C	-5.046744	-4.334269	-4.424235

H	-4.683176	-5.010055	-5.213490

H	-5.365780	-4.938859	-3.553392

H	-5.938357	-3.782729	-4.774743

C	0.133314	-2.487952	-2.501976

C	0.348350	-3.188354	-1.310556

H	-0.630494	-2.813919	-3.219600

H	0.887078	-1.786626	-2.882376

H	-0.298596	-4.032101	-1.043470

H	1.287256	-3.093471	-0.757428

O	-2.792611	-3.285592	-0.986807

H	-5.022527	-2.567040	-2.313085

C	-3.762511	1.673514	0.963438

C	-4.315107	1.697819	-0.332307

C	-4.678142	1.538377	2.028025

C	-5.689151	1.557445	-0.568775

C	-6.060298	1.414722	1.821196

C	-6.569223	1.420903	0.514652

F	-4.235559	1.501081	3.304264

F	-6.900411	1.277490	2.867575

F	-7.893781	1.297503	0.301086

F	-6.172082	1.571272	-1.830271

F	-3.517370	1.867175	-1.413322

O	4.008927	0.524833	0.956938

O	0.186438	-2.188426	1.888178

O	3.324221	-1.508435	-1.241516

O	0.741230	2.460021	-1.520031

C	4.637222	1.626901	1.526785

C	5.536059	2.307555	0.684143

C	4.369090	2.102695	2.823648

C	6.176826	3.464085	1.149531

H	5.705877	1.924585	-0.327528

C	5.023798	3.261547	3.273527

H	3.662912	1.574998	3.471145

C	5.929217	3.945254	2.447098

H	6.877154	3.990421	0.491051

H	4.814229	3.631581	4.283679

H	6.435100	4.846753	2.808678

C	-0.746866	-2.558934	2.862223

C	-0.800940	-1.956193	4.131254

C	-1.704005	-3.507047	2.467076

C	-1.816322	-2.338722	5.021821

H	-0.069069	-1.192868	4.411192

C	-2.716780	-3.869901	3.367635

H	-1.667728	-3.918043	1.453746

C	-2.772663	-3.297760	4.649538

H	-1.864108	-1.868366	6.010608

H	-3.468545	-4.604692	3.058321

H	-3.563917	-3.586953	5.349525

C	4.464590	-2.273906	-1.512292

C	4.307524	-3.452677	-2.255869

C	5.708711	-1.911992	-0.969833

C	5.421147	-4.284328	-2.459604

H	3.321303	-3.706619	-2.657280

C	6.814002	-2.748631	-1.188164

H	5.789681	-0.994782	-0.378777

C	6.674449	-3.933882	-1.931324

H	5.305150	-5.208892	-3.036135

H	7.787909	-2.476110	-0.765864

H	7.540328	-4.585010	-2.093615

C	0.702724	3.849190	-1.467649

C	-0.551990	4.433664	-1.711833

C	1.810980	4.636556	-1.107227

C	-0.689590	5.825308	-1.612905

H	-1.401557	3.785909	-1.949049

C	1.655666	6.029233	-1.020366

H	2.774293	4.165051	-0.889694

C	0.412324	6.630352	-1.275968

H	-1.667706	6.282306	-1.801540

H	2.517284	6.645649	-0.738831

H	0.300574	7.717724	-1.204979

**TS-2-Ins-E-C**

SCF Done: -3655.3413182 A.U.

C	2.910839	0.395278	3.147289

C	2.155136	0.562063	4.340459

C	2.715724	1.129568	5.499620

H	2.115743	1.205643	6.409883

C	4.035370	1.595537	5.464502

H	4.479271	2.029436	6.367302

C	4.791863	1.521443	4.288510

H	5.814659	1.905655	4.248312

C	4.218647	0.944723	3.141160

C	3.479042	-1.208539	0.678595

C	4.392793	-2.115908	1.276556

C	5.351323	-2.813961	0.524327

H	6.049102	-3.491212	1.023613

C	5.390129	-2.631591	-0.865514

H	6.120243	-3.188929	-1.462890

C	4.505024	-1.754770	-1.498845

H	4.523179	-1.619750	-2.583432

C	3.570308	-1.038854	-0.725225

C	1.016541	-1.738507	2.087238

C	1.239012	-2.778249	3.006150

H	2.139920	-2.772301	3.626393

C	0.304020	-3.815662	3.134478

H	0.470918	-4.627647	3.850157

C	-0.851667	-3.802637	2.330997

H	-1.582972	-4.615036	2.419703

C	-1.112401	-2.761491	1.427225

C	-0.174533	-1.675848	1.287076

C	-0.727639	1.803601	-0.633755

Ni	0.823793	0.826149	0.415804

O	-0.410015	-0.689640	0.447432

P	2.139702	-0.367082	1.650143

C	-1.774476	2.111882	0.443766

H	-2.033463	1.136164	0.890105

C	-3.044652	2.751612	-0.141209

H	-3.889723	2.667107	0.570425

H	-1.339723	2.744602	1.236279

C	-2.879337	4.219104	-0.531412

O	-1.802653	4.809072	-0.430685

C	-4.121997	4.927928	-1.059156

H	-3.880533	5.970064	-1.318220

H	-4.512826	4.404218	-1.951741

H	-4.929713	4.910871	-0.303348

C	0.768891	2.721583	-0.543370

C	1.797985	2.459780	0.489502

H	1.134432	2.656302	-1.577299

H	0.171812	3.639059	-0.367784

H	2.837517	2.391462	0.141463

H	1.689856	2.950134	1.469317

O	-1.031724	1.405932	-1.753277

H	-3.357244	2.186555	-1.041706

C	-2.345839	-2.768207	0.597042

C	-3.304089	-1.740993	0.684118

C	-2.614724	-3.800810	-0.322823

C	-4.452521	-1.713425	-0.117531

C	-3.764783	-3.807988	-1.126931

C	-4.686526	-2.756126	-1.026252

F	-1.742295	-4.822480	-0.469838

F	-3.984522	-4.811124	-2.002276

F	-5.793470	-2.748802	-1.794803

F	-5.345245	-0.705509	-0.006092

F	-3.147556	-0.743645	1.585816

O	2.645646	-0.204417	-1.332260

O	4.333426	-2.211980	2.655902

O	0.855631	0.104990	4.294123

O	4.928690	0.829666	1.957757

C	4.897320	-3.312387	3.307500

C	5.827436	-3.049100	4.324336

C	4.470851	-4.623356	3.030437

C	6.344967	-4.119338	5.071749

H	6.122368	-2.013575	4.520889

C	5.003510	-5.682944	3.780740

H	3.725658	-4.798367	2.248101

C	5.940104	-5.436516	4.799780

H	7.069919	-3.919299	5.868719

H	4.674176	-6.707113	3.572217

H	6.347960	-6.268814	5.383626

C	2.996336	0.529736	-2.468466

C	4.248309	1.157163	-2.605989

C	1.986136	0.703088	-3.429478

C	4.491111	1.946123	-3.741788

H	5.016856	1.030467	-1.837018

C	2.245390	1.503229	-4.553431

H	1.007698	0.241575	-3.266617

C	3.497085	2.119943	-4.719567

H	5.464806	2.437024	-3.852776

H	1.457679	1.643917	-5.302022

H	3.694167	2.739752	-5.600934

C	-0.084932	0.491408	5.249971

C	-0.366412	1.844348	5.510831

C	-0.822554	-0.534935	5.859738

C	-1.392876	2.163554	6.413229

H	0.209287	2.629094	5.009802

C	-1.851909	-0.198809	6.752873

H	-0.590767	-1.574723	5.608734

C	-2.136323	1.147119	7.037399

H	-1.617998	3.216060	6.620130

H	-2.434169	-0.996683	7.227288

H	-2.939216	1.404405	7.736721

C	5.913088	1.751416	1.611987

C	5.737719	3.140222	1.755355

C	7.066256	1.228030	1.002725

C	6.742291	4.004476	1.293020

H	4.827423	3.532889	2.218421

C	8.057043	2.106943	0.538141

H	7.162223	0.142766	0.895860

C	7.903002	3.495560	0.685780

H	6.608014	5.086761	1.401365

H	8.956564	1.699918	0.062580

H	8.680432	4.178148	0.326176

**2-cycle5-T**

SCF Done: -3655.37373424 A.U.

C	2.300372	-0.055589	1.546816

C	1.797456	-0.677265	2.724319

C	2.599846	-0.876980	3.862162

H	2.174810	-1.355812	4.748015

C	3.927871	-0.433606	3.844955

H	4.562106	-0.591434	4.724407

C	4.448170	0.230846	2.727694

H	5.478174	0.597937	2.717914

C	3.627202	0.441951	1.604676

C	2.066545	0.504522	-1.422603

C	3.035109	-0.406035	-1.918951

C	3.591740	-0.276887	-3.201617

H	4.343609	-0.991264	-3.546759

C	3.162072	0.774031	-4.024415

H	3.583538	0.876587	-5.030730

C	2.218896	1.702727	-3.574230

H	1.895327	2.532077	-4.208653

C	1.693274	1.574739	-2.274163

C	0.239474	-1.369099	-0.207195

C	0.731946	-2.682333	-0.206392

H	1.761681	-2.879755	0.107196

C	-0.100097	-3.743815	-0.601998

H	0.273311	-4.773146	-0.590699

C	-1.422278	-3.475242	-0.994149

H	-2.075012	-4.302283	-1.297210

C	-1.950264	-2.171559	-0.975513

C	-1.115759	-1.069902	-0.574844

C	-2.089648	3.155279	1.953009

Ni	-0.553628	1.480187	0.470190

O	-1.575955	0.164232	-0.554296

P	1.127916	0.195628	0.145588

C	-3.326775	3.833530	2.510768

H	-3.125855	4.163891	3.544478

C	-4.557643	2.915852	2.455375

H	-4.756370	2.596429	1.414320

H	-3.514758	4.745252	1.907685

C	-4.360235	1.654022	3.302815

O	-3.298766	1.456098	3.895605

C	-5.507736	0.660796	3.335852

H	-6.475331	1.162659	3.518300

H	-5.577475	0.168044	2.348258

H	-5.325008	-0.104679	4.105250

C	-0.722019	3.327434	2.543593

C	0.329446	2.837787	1.527098

H	-0.576693	4.366416	2.915544

H	-0.724071	2.688266	3.451726

H	0.559584	3.621743	0.778924

H	1.265355	2.544325	2.030803

O	-2.203999	2.400643	0.954549

H	-5.467070	3.449008	2.794286

C	-3.358823	-1.908179	-1.363172

C	-4.250113	-1.219371	-0.513993

C	-3.879433	-2.322433	-2.606855

C	-5.568847	-0.931793	-0.888860

C	-5.201614	-2.061565	-2.997538

C	-6.052384	-1.356540	-2.134300

F	-3.090449	-2.978860	-3.487651

F	-5.655150	-2.468803	-4.202000

F	-7.324567	-1.094726	-2.495599

F	-6.392685	-0.266820	-0.041599

F	-3.863956	-0.841995	0.722732

O	0.824372	2.524199	-1.761623

O	3.434218	-1.397362	-1.039933

O	4.110951	1.087897	0.479853

O	0.449851	-0.969491	2.714015

C	-0.081373	-1.951890	3.552161

C	0.489308	-3.233627	3.643346

C	-1.269420	-1.633462	4.229003

C	-0.134520	-4.201724	4.444793

H	1.401851	-3.465710	3.085789

C	-1.883333	-2.617642	5.019962

H	-1.708339	-0.635810	4.122791

C	-1.319037	-3.898677	5.137269

H	0.305215	-5.203135	4.517002

H	-2.811121	-2.374525	5.550222

H	-1.802929	-4.660047	5.758852

C	5.031305	2.125493	0.594687

C	6.021916	2.190015	-0.399682

C	4.936367	3.121999	1.583730

C	6.929835	3.259648	-0.398544

H	6.055350	1.408581	-1.165290

C	5.857491	4.180592	1.575398

H	4.149798	3.067373	2.342445

C	6.856691	4.255068	0.590061

H	7.701012	3.310455	-1.175559

H	5.784358	4.958832	2.343816

H	7.570178	5.086183	0.590547

C	-0.039537	3.188224	-2.645383

C	-1.236665	2.560506	-3.025833

C	0.263756	4.499371	-3.042312

C	-2.135507	3.264432	-3.842820

H	-1.460665	1.559026	-2.640902

C	-0.647728	5.190456	-3.859233

H	1.198128	4.961960	-2.707360

C	-1.843053	4.573745	-4.264807

H	-3.076558	2.788514	-4.141441

H	-0.420569	6.214662	-4.176689

H	-2.550972	5.116439	-4.901199

C	4.096210	-2.530852	-1.517863

C	3.479528	-3.407128	-2.428411

C	5.357985	-2.816585	-0.974619

C	4.153404	-4.579085	-2.804928

H	2.482995	-3.173575	-2.815546

C	6.016270	-3.997452	-1.354788

H	5.799504	-2.116362	-0.258612

C	5.420062	-4.877593	-2.272693

H	3.677618	-5.268320	-3.511728

H	7.000817	-4.226381	-0.931529

H	5.937326	-5.796795	-2.568714

**TS-Isom-CO-T**

SCF Done: -3576.760854 A.U.

C	-1.163465	3.971393	-2.801240

C	-1.001199	5.315957	-3.238565

C	-2.076609	6.223935	-3.243406

H	-1.916341	7.247222	-3.590862

C	-3.330457	5.807240	-2.782408

H	-4.169023	6.512374	-2.790627

C	-3.523863	4.509555	-2.293631

H	-4.496212	4.184948	-1.914077

C	-2.438712	3.617501	-2.283642

C	-0.439951	1.099409	-3.178795

C	-1.179967	0.901163	-4.371241

C	-1.557284	-0.382207	-4.801375

H	-2.135790	-0.500843	-5.721236

C	-1.168743	-1.493697	-4.042098

H	-1.448854	-2.498865	-4.376156

C	-0.433943	-1.342946	-2.859713

H	-0.135298	-2.211684	-2.268483

C	-0.090667	-0.048019	-2.433021

C	1.516243	2.994404	-3.958696

C	1.380534	3.461726	-5.276588

H	0.413249	3.835173	-5.625042

C	2.483235	3.444558	-6.142502

H	2.387119	3.816825	-7.167742

C	3.720821	2.962493	-5.678439

H	4.587083	2.964322	-6.350184

C	3.885478	2.490530	-4.366083

C	2.759890	2.464701	-3.465545

Ni	1.435576	2.411999	-0.851475

O	2.871977	1.968396	-2.252889

P	0.219365	2.761277	-2.700308

C	1.149162	4.290593	-0.246026

H	0.791800	4.879779	-1.102166

C	2.516364	4.778633	0.214444

H	3.301083	4.490532	-0.517423

H	0.384623	4.302864	0.547588

C	2.630679	6.308424	0.370548

O	1.823345	7.096430	-0.114807

H	2.831456	4.311585	1.167419

C	3.837670	6.792504	1.169699

H	4.760777	6.275893	0.848709

H	3.953173	7.881689	1.057933

H	3.693228	6.552587	2.240779

C	5.201171	1.995265	-3.888271

C	5.909574	0.984142	-4.569427

C	5.810039	2.510519	-2.724934

C	7.146822	0.498600	-4.120561

C	7.038399	2.033326	-2.247304

C	7.712676	1.023583	-2.949927

F	5.391237	0.427831	-5.688448

F	7.787969	-0.477287	-4.797635

F	8.899579	0.565229	-2.505875

F	7.587979	2.553707	-1.129514

F	5.232926	3.523420	-2.043468

C	1.726193	1.949574	0.845212

O	1.752855	1.673367	1.979334

O	0.585011	0.170275	-1.235376

O	-1.544798	2.046316	-5.056521

O	0.271418	5.683128	-3.617065

O	-2.569461	2.321108	-1.817697

C	0.499030	6.978037	-4.110657

C	0.285547	7.246203	-5.471651

C	1.019411	7.946808	-3.241061

C	0.601256	8.519982	-5.970744

H	-0.112908	6.462271	-6.123569

C	1.332144	9.215544	-3.757212

H	1.180186	7.698174	-2.186320

C	1.123970	9.505535	-5.116105

H	0.443370	8.738336	-7.032976

H	1.743725	9.979411	-3.087946

H	1.372980	10.496841	-5.510875

C	-3.521608	2.008996	-0.847033

C	-4.304701	0.866317	-1.073504

C	-3.627235	2.736432	0.351983

C	-5.210485	0.449500	-0.085146

H	-4.185078	0.317905	-2.013110

C	-4.543374	2.311749	1.326200

H	-2.997186	3.616605	0.513917

C	-5.336970	1.171081	1.113251

H	-5.822140	-0.443237	-0.257804

H	-4.628215	2.873350	2.263471

H	-6.047140	0.844589	1.880612

C	1.308232	-0.902750	-0.667854

C	2.653126	-1.070303	-1.027100

C	0.688374	-1.705847	0.299569

C	3.388559	-2.091691	-0.404179

H	3.099731	-0.376611	-1.747858

C	1.439210	-2.724603	0.910800

H	-0.357730	-1.524894	0.567074

C	2.784825	-2.921002	0.557343

H	4.443124	-2.232396	-0.666555

H	0.970171	-3.360029	1.670371

H	3.366659	-3.713914	1.040087

C	-1.879647	1.969894	-6.411482

C	-0.959626	1.496944	-7.363670

C	-3.128554	2.480373	-6.796177

C	-1.312933	1.527443	-8.721415

H	0.018386	1.129202	-7.037930

C	-3.463991	2.511917	-8.159584

H	-3.812462	2.851700	-6.026477

C	-2.562110	2.032116	-9.123510

H	-0.600158	1.163208	-9.469875

H	-4.437252	2.911391	-8.466129

H	-2.828562	2.055863	-10.185791

**1-Coor-CO-C**

SCF Done: -3576.79205387 A.U.

C	1.424069	1.103785	-0.988259

C	1.528222	2.410266	-0.442714

C	2.069430	3.477885	-1.184224

H	2.115052	4.473624	-0.735261

C	2.524948	3.252256	-2.487118

H	2.953225	4.080499	-3.062309

C	2.424990	1.983609	-3.071694

H	2.769614	1.803557	-4.092979

C	1.855512	0.935438	-2.330408

C	1.821584	-1.720904	-0.172888

C	3.218230	-1.550531	-0.016439

C	4.110778	-2.635807	-0.076945

H	5.182084	-2.467165	0.061968

C	3.600091	-3.922499	-0.286768

H	4.287742	-4.774298	-0.333527

C	2.222643	-4.143289	-0.416028

H	1.825181	-5.150008	-0.567488

C	1.344606	-3.047253	-0.345275

C	0.284148	0.005019	1.613525

C	1.209648	-0.049728	2.668600

H	2.258282	-0.286083	2.459354

C	0.790071	0.212990	3.980685

H	1.502676	0.170249	4.810813

C	-0.551752	0.553577	4.219335

H	-0.878925	0.778426	5.240816

C	-1.496980	0.625199	3.179298

C	-1.095540	0.306584	1.837902

Ni	-1.550067	-0.404293	-0.850557

O	-1.992958	0.278143	0.862828

P	0.610794	-0.331471	-0.145593

C	-3.471483	-0.148831	-1.323233

H	-3.838477	-0.686347	-2.212006

C	-3.687027	1.354720	-1.429153

H	-3.368021	1.850450	-0.489335

H	-3.916070	-0.567652	-0.406246

C	-2.967725	2.053328	-2.582215

O	-2.113816	1.497238	-3.274872

H	-4.771321	1.595646	-1.522861

C	-3.362310	3.505660	-2.838498

H	-3.382124	4.084783	-1.897167

H	-2.663000	3.968019	-3.552048

H	-4.385472	3.545450	-3.259363

C	-2.908999	0.990943	3.457040

C	-3.677306	0.311782	4.425502

C	-3.556418	2.039123	2.772289

C	-5.014311	0.641935	4.692695

C	-4.894467	2.381000	3.008789

C	-5.628025	1.680541	3.977836

F	-3.138678	-0.713976	5.122258

F	-5.716133	-0.039556	5.621258

F	-6.911672	2.006866	4.222948

F	-5.475052	3.391630	2.327740

F	-2.881551	2.779002	1.859119

C	-1.426750	-1.165619	-2.393875

O	-1.387097	-1.762634	-3.394298

O	-0.022862	-3.187577	-0.477067

O	3.654261	-0.274665	0.310487

O	0.973453	2.615047	0.803459

O	1.740391	-0.348560	-2.843456

C	1.709074	-0.534233	-4.231986

C	2.605839	-1.468468	-4.771014

C	0.760640	0.119967	-5.037675

C	2.559946	-1.747381	-6.146788

H	3.314807	-1.974511	-4.107633

C	0.735106	-0.163845	-6.411630

H	0.045339	0.818199	-4.590569

C	1.631162	-1.091849	-6.970937

H	3.255889	-2.479125	-6.572461

H	-0.004920	0.336551	-7.046358

H	1.598566	-1.309356	-8.044146

C	1.470472	3.580349	1.672584

C	0.516842	4.248215	2.459226

C	2.846286	3.821489	1.839778

C	0.949172	5.178625	3.415388

H	-0.542347	4.011156	2.320444

C	3.259808	4.762672	2.795719

H	3.576863	3.277004	1.233130

C	2.318597	5.445863	3.583876

H	0.207203	5.699472	4.031295

H	4.330859	4.955097	2.928418

H	2.650699	6.177587	4.328345

C	4.927739	0.148781	-0.068582

C	5.397021	0.001590	-1.385531

C	5.693121	0.813189	0.903315

C	6.656252	0.521021	-1.721194

H	4.773362	-0.501410	-2.130736

C	6.947305	1.335848	0.549147

H	5.297253	0.911278	1.919238

C	7.435002	1.188008	-0.759667

H	7.025072	0.411938	-2.747411

H	7.547364	1.854075	1.305566

H	8.415781	1.593152	-1.030998

C	-0.606896	-4.451644	-0.328965

C	-1.245897	-5.016672	-1.442166

C	-0.632824	-5.079660	0.927555

C	-1.923386	-6.238056	-1.290711

H	-1.214716	-4.491604	-2.401729

C	-1.307543	-6.302738	1.061346

H	-0.140241	-4.604699	1.782159

C	-1.952706	-6.884383	-0.044162

H	-2.430146	-6.683275	-2.154217

H	-1.338166	-6.796652	2.039037

H	-2.483249	-7.836088	0.068872

**TS-1-Ins-CO-C**

SCF Done: -3576.77522907 A.U.

C	2.528680	0.722711	2.455029

C	1.893683	1.671569	3.294880

C	2.632219	2.589257	4.066350

H	2.103914	3.313631	4.691996

C	4.028585	2.579176	3.988910

H	4.608964	3.294565	4.582011

C	4.696188	1.689033	3.135129

H	5.785859	1.699801	3.049776

C	3.944851	0.790123	2.360523

C	2.418397	-2.009860	1.215231

C	2.987813	-2.676738	2.329347

C	3.642452	-3.912311	2.194435

H	4.093989	-4.389507	3.068135

C	3.699926	-4.518559	0.931294

H	4.198930	-5.487864	0.822409

C	3.117672	-3.914119	-0.187844

H	3.136765	-4.399211	-1.167201

C	2.484070	-2.666397	-0.038299

C	-0.098892	-0.757878	1.979926

C	-0.410105	-1.404361	3.187442

H	0.393292	-1.700050	3.870441

C	-1.749115	-1.653778	3.523634

H	-2.000160	-2.149471	4.466940

C	-2.769060	-1.231466	2.654773

H	-3.816518	-1.401743	2.928267

C	-2.485943	-0.572187	1.444769

C	-1.119911	-0.353275	1.057350

Ni	0.987033	0.590304	-0.522121

O	-0.831891	0.191092	-0.112997

P	1.567931	-0.377300	1.331363

C	0.746401	1.790388	-2.264864

H	1.322215	1.939012	-3.190582

C	-0.055434	3.031282	-1.874195

H	0.609221	3.823594	-1.469449

H	0.081325	0.924935	-2.463248

C	-0.808131	3.643759	-3.068232

O	-0.566531	3.314453	-4.226175

H	-0.776375	2.797167	-1.068780

C	-1.877234	4.671540	-2.719094

H	-1.493194	5.419905	-2.001080

H	-2.234784	5.172255	-3.632006

H	-2.726820	4.163807	-2.224340

C	-3.576792	-0.106639	0.551581

C	-4.600041	-0.972270	0.111800

C	-3.651393	1.226146	0.095660

C	-5.632794	-0.549933	-0.738694

C	-4.664538	1.669041	-0.764301

C	-5.663265	0.779015	-1.185013

F	-4.600217	-2.269692	0.491683

F	-6.585848	-1.414941	-1.141403

F	-6.646610	1.199406	-2.003032

F	-4.699713	2.958083	-1.176708

F	-2.744980	2.145888	0.503995

C	2.222777	1.548519	-1.236422

O	3.210200	2.196939	-1.404536

O	1.814236	-2.067567	-1.091725

O	2.917593	-2.003218	3.537423

O	0.516508	1.743868	3.215833

O	4.532549	-0.097936	1.479591

C	2.252797	-2.269720	-2.404582

C	1.273472	-2.586689	-3.358355

C	3.593982	-2.066907	-2.773514

C	1.647222	-2.705067	-4.706743

H	0.237861	-2.729690	-3.033952

C	3.952251	-2.198616	-4.124148

H	4.336757	-1.803330	-2.013984

C	2.984648	-2.516617	-5.093011

H	0.886167	-2.948303	-5.456523

H	4.995107	-2.037879	-4.420121

H	3.271526	-2.610742	-6.145845

C	3.068956	-2.686430	4.745587

C	2.287590	-3.811500	5.064845

C	3.959427	-2.132746	5.678653

C	2.418352	-4.391187	6.336324

H	1.589205	-4.218777	4.326802

C	4.072641	-2.719470	6.949030

H	4.538569	-1.246857	5.399363

C	3.308456	-3.850625	7.280255

H	1.811083	-5.266969	6.591386

H	4.765131	-2.289045	7.681086

H	3.401848	-4.307041	8.271553

C	-0.267990	2.012330	4.333597

C	-1.510659	2.614771	4.071986

C	0.094234	1.640237	5.640439

C	-2.387204	2.866627	5.136502

H	-1.780451	2.849218	3.037881

C	-0.793595	1.907120	6.695011

H	1.052187	1.145673	5.826885

C	-2.031673	2.523603	6.452447

H	-3.356940	3.334802	4.932838

H	-0.512860	1.618562	7.714587

H	-2.718193	2.727529	7.281203

C	5.866999	0.094438	1.098913

C	6.191710	1.095161	0.168564

C	6.836081	-0.791012	1.593036

C	7.523093	1.210836	-0.261216

H	5.402914	1.747215	-0.220569

C	8.163234	-0.665205	1.149630

H	6.538808	-1.569593	2.302656

C	8.509661	0.336389	0.227707

H	7.786839	1.984579	-0.991037

H	8.926655	-1.354744	1.527244

H	9.545761	0.431231	-0.115529

**1-cycle6-T**

SCF Done: -3576.79793661 A.U.

C	-1.623112	-0.203410	1.729846

C	-0.875683	-0.612873	2.860837

C	-1.279504	-0.292266	4.170720

H	-0.668894	-0.617057	5.017468

C	-2.465010	0.422447	4.365479

H	-2.789314	0.665085	5.383602

C	-3.256992	0.816246	3.277570

H	-4.194815	1.356647	3.429590

C	-2.828026	0.506969	1.977481

C	-2.163470	-1.160253	-1.090124

C	-1.943705	-1.063564	-2.490906

C	-2.781484	-1.699577	-3.422489

H	-2.592132	-1.574787	-4.491992

C	-3.836284	-2.495502	-2.959742

H	-4.498067	-2.990180	-3.679219

C	-4.049614	-2.668822	-1.587004

H	-4.855575	-3.306636	-1.212642

C	-3.205850	-2.020052	-0.668901

C	-0.657506	1.379079	-0.473034

C	-1.567398	2.295545	-1.019420

H	-2.592578	1.983563	-1.241973

C	-1.152165	3.608808	-1.299051

H	-1.852069	4.328111	-1.737150

C	0.168283	3.993518	-1.007750

H	0.490181	5.021348	-1.213217

C	1.097945	3.093792	-0.456652

C	0.709333	1.729612	-0.212761

C	0.741214	-2.872398	-0.328942

Ni	1.109867	-1.082108	-0.020699

O	1.577720	0.831891	0.207026

P	-0.937591	-0.361998	0.018489

C	1.584276	-3.772549	0.613906

C	3.038919	-3.836175	0.110284

H	3.071818	-4.314585	-0.892699

H	1.149199	-4.788423	0.613275

C	3.701685	-2.477648	-0.033712

O	3.046340	-1.418750	-0.094645

O	0.062992	-3.389476	-1.214419

H	3.666688	-4.472630	0.763358

C	5.208687	-2.416571	-0.115815

H	5.638803	-2.646959	0.878493

H	5.596653	-3.178911	-0.816414

H	5.536612	-1.412758	-0.424747

C	2.499011	3.498927	-0.177906

C	3.336641	4.041936	-1.173287

C	3.077357	3.326707	1.095335

C	4.676911	4.377242	-0.927187

C	4.418185	3.629762	1.362905

C	5.223842	4.163526	0.346544

F	2.867088	4.232463	-2.426675

F	5.448978	4.885810	-1.910927

F	6.512574	4.473533	0.592949

F	4.932359	3.430698	2.598290

F	2.327215	2.878751	2.127337

H	1.561169	-3.371030	1.641281

O	-3.416861	-2.178701	0.693855

O	-0.885138	-0.270304	-2.885375

O	-3.570099	0.831173	0.858151

O	0.251078	-1.398750	2.651273

C	-0.217105	-0.552836	-4.080028

C	0.284968	-1.838784	-4.347718

C	0.014608	0.520993	-4.952112

C	1.017154	-2.043562	-5.527140

H	0.112265	-2.643685	-3.625806

C	0.758593	0.299477	-6.122434

H	-0.377610	1.510200	-4.696438

C	1.255155	-0.980959	-6.417200

H	1.414382	-3.041773	-5.745681

H	0.947567	1.134761	-6.806177

H	1.831950	-1.149557	-7.333302

C	-3.613422	-3.461609	1.199851

C	-4.504777	-3.579341	2.279393

C	-2.903165	-4.578500	0.720593

C	-4.692872	-4.832563	2.881876

H	-5.029889	-2.687018	2.634479

C	-3.111384	-5.825537	1.329967

H	-2.191240	-4.463072	-0.103362

C	-4.004147	-5.961242	2.407159

H	-5.388148	-4.925021	3.724087

H	-2.560798	-6.698502	0.960285

H	-4.158637	-6.939365	2.875638

C	-4.578767	1.795217	0.946166

C	-5.887194	1.394844	0.637336

C	-4.273988	3.136895	1.233689

C	-6.910854	2.356320	0.621608

H	-6.081250	0.342688	0.406878

C	-5.308839	4.084740	1.221733

H	-3.239110	3.424212	1.443438

C	-6.626401	3.699498	0.917767

H	-7.934572	2.050600	0.377986

H	-5.079364	5.133652	1.440974

H	-7.427697	4.446447	0.905819

C	1.347080	-1.215781	3.512121

C	1.666245	-2.227346	4.430923

C	2.151196	-0.075719	3.362147

C	2.816801	-2.080497	5.227303

H	1.017678	-3.105803	4.517421

C	3.292937	0.058661	4.166602

H	1.900102	0.662654	2.594827

C	3.626717	-0.939050	5.100958

H	3.074515	-2.861376	5.952115

H	3.922752	0.947310	4.045167

H	4.519431	-0.829641	5.726987

**TS-2-Coor-E-T**

SCF Done: -3655.34924911 A.U.

C	-2.325324	3.093396	1.494365

C	-1.509634	4.194103	1.852990

C	-1.994630	5.245154	2.654978

H	-1.328844	6.071938	2.916414

C	-3.312401	5.201380	3.120260

H	-3.693287	6.015970	3.746355

C	-4.145497	4.114847	2.817447

H	-5.170396	4.068302	3.194204

C	-3.638668	3.066409	2.034517

C	-3.016063	1.256444	-0.747433

C	-3.784686	2.252355	-1.408070

C	-4.697538	1.937554	-2.430074

H	-5.289263	2.733584	-2.890338

C	-4.818275	0.609695	-2.857992

H	-5.521271	0.359638	-3.660178

C	-4.048634	-0.397812	-2.268388

H	-4.121202	-1.436201	-2.603664

C	-3.165644	-0.069195	-1.224094

C	-0.294938	2.032495	-0.555495

C	-0.226610	3.040578	-1.532301

H	-1.021431	3.791850	-1.598236

C	0.860678	3.092681	-2.416170

H	0.929963	3.888579	-3.164729

C	1.865526	2.111010	-2.329595

H	2.710545	2.138149	-3.028125

C	1.825499	1.095842	-1.360685

C	0.726523	1.030373	-0.427585

C	-2.135246	0.001486	3.065477

Ni	-0.842112	0.033221	1.727003

O	0.656380	0.072190	0.463747

P	-1.712207	1.645138	0.534613

C	-2.889491	-1.334860	3.296387

H	2.489671	0.071728	3.252253

C	-2.419908	-2.528164	2.443598

H	-2.660862	-2.318104	1.382044

H	-3.954090	-1.125456	3.079142

C	-0.917437	-2.718308	2.497480

O	-0.155256	-1.746346	2.326438

O	-2.331926	0.921750	3.855901

H	-2.945567	-3.454496	2.735218

C	-0.340739	-4.091700	2.732915

H	-0.711146	-4.786393	1.954668

H	0.758924	-4.062795	2.711589

H	-0.690113	-4.489639	3.704948

C	2.868375	0.039145	-1.303665

C	3.111063	-0.816898	-2.394899

C	3.635822	-0.191493	-0.144532

C	4.055826	-1.851422	-2.348761

C	4.582783	-1.222423	-0.067054

C	4.797775	-2.054126	-1.176438

F	2.381549	-0.689440	-3.533555

F	4.240572	-2.661625	-3.413666

F	5.711300	-3.043412	-1.115178

F	5.301823	-1.410702	1.059319

F	3.491904	0.606181	0.933249

C	0.935945	1.508698	3.494653

C	1.698633	0.474021	3.893542

H	1.107277	2.002261	2.532196

H	0.127135	1.910316	4.114795

H	-2.824382	-1.568656	4.376723

H	1.542665	-0.019862	4.861251

O	-2.424751	-1.074034	-0.605855

O	-3.625445	3.555496	-0.965634

O	-0.182021	4.150986	1.465625

O	-4.406169	1.970148	1.679422

C	0.499938	5.325761	1.157288

C	-0.078044	6.352583	0.388779

C	1.837739	5.403302	1.577266

C	0.699258	7.471679	0.052889

H	-1.118742	6.271376	0.059466

C	2.603289	6.525098	1.225678

H	2.258842	4.583324	2.167303

C	2.038566	7.564569	0.467654

H	0.252437	8.272450	-0.547660

H	3.647850	6.585514	1.551568

H	2.639203	8.440218	0.198537

C	-5.592619	1.691473	2.363166

C	-6.778763	1.659609	1.613966

C	-5.580242	1.357384	3.729562

C	-7.977268	1.290252	2.246368

H	-6.744387	1.913140	0.549709

C	-6.788750	0.997923	4.347333

H	-4.629838	1.364426	4.274290

C	-7.987253	0.964553	3.612722

H	-8.906519	1.260797	1.666114

H	-6.789787	0.734654	5.411431

H	-8.924883	0.680593	4.103197

C	-1.888065	-2.079706	-1.427022

C	-0.785281	-1.796723	-2.248934

C	-2.413143	-3.378483	-1.334697

C	-0.203271	-2.837438	-2.988153

H	-0.376980	-0.782353	-2.286566

C	-1.819608	-4.412397	-2.080579

H	-3.281247	-3.566848	-0.693610

C	-0.716370	-4.144282	-2.906788

H	0.669721	-2.619438	-3.612023

H	-2.226950	-5.427953	-2.015781

H	-0.253176	-4.952591	-3.483229

C	-3.860825	4.640668	-1.806513

C	-3.382292	4.694692	-3.129165

C	-4.511534	5.743328	-1.227708

C	-3.578661	5.866707	-3.876158

H	-2.862240	3.833161	-3.559203

C	-4.692037	6.910176	-1.986386

H	-4.853499	5.670750	-0.190349

C	-4.233200	6.975283	-3.312716

H	-3.206307	5.912893	-4.905880

H	-5.198736	7.771119	-1.535885

H	-4.380031	7.886201	-3.902979

**2-Coor-E-T**

SCF Done: -3655.35734365 A.U.

C	-2.311685	0.408900	0.847093

C	-2.507202	1.564344	1.653400

C	-3.781938	1.948976	2.104184

H	-3.903772	2.868948	2.680941

C	-4.877935	1.128145	1.810216

H	-5.875661	1.418346	2.157949

C	-4.718620	-0.069550	1.102863

H	-5.568071	-0.731347	0.918319

C	-3.439230	-0.434222	0.644665

C	-0.725634	-1.032116	-1.280909

C	-1.537417	-0.712962	-2.395863

C	-1.503422	-1.467543	-3.580250

H	-2.156471	-1.203543	-4.416317

C	-0.622735	-2.553856	-3.664629

H	-0.590082	-3.150884	-4.582723

C	0.212860	-2.894698	-2.594443

H	0.900686	-3.740465	-2.665539

C	0.157923	-2.130421	-1.415039

C	0.131495	1.548400	-0.472700

C	-0.520534	2.652451	-1.038478

H	-1.614755	2.699944	-1.037771

C	0.234235	3.701020	-1.591892

H	-0.264674	4.572135	-2.029290

C	1.637249	3.610258	-1.596219

H	2.230251	4.419194	-2.039172

C	2.311573	2.499666	-1.057666

C	1.560645	1.435563	-0.442668

C	0.104884	-1.851382	2.572381

Ni	1.111352	-0.776057	1.400157

O	2.158600	0.413961	0.133419

P	-0.619507	0.035233	0.227013

C	-0.792026	-1.190248	3.625369

H	-0.359074	-0.224809	3.937131

C	-1.068600	-2.132983	4.810846

H	-1.587510	-1.565554	5.610275

H	-1.750209	-0.961286	3.118520

C	-2.000813	-3.288404	4.416701

O	-2.973730	-3.086018	3.691498

C	-1.684040	-4.659180	4.990685

H	-0.735028	-5.014067	4.547126

H	-1.529866	-4.605765	6.085033

H	-2.489952	-5.371701	4.757150

C	2.400986	-0.144368	2.933732

C	2.946724	-1.249060	2.282898

H	2.638241	0.871106	2.599791

H	1.941633	-0.240872	3.924547

H	3.610252	-1.109834	1.422942

H	2.891014	-2.253420	2.716905

O	0.201617	-3.070903	2.508316

H	-0.129330	-2.519894	5.244996

C	3.794292	2.421360	-1.077647

C	4.605829	3.422127	-0.506048

C	4.467495	1.332889	-1.667025

C	6.006735	3.342708	-0.502782

C	5.863855	1.218348	-1.662519

C	6.639338	2.232478	-1.080732

F	3.764172	0.361785	-2.293784

F	6.467927	0.154968	-2.235349

F	7.983666	2.142186	-1.081063

F	6.748087	4.317493	0.063817

F	4.040227	4.496629	0.089416

O	-2.418832	0.341413	-2.220874

O	0.953107	-2.432769	-0.316650

O	-3.198978	-1.622583	-0.005343

O	-1.361855	2.284757	1.931414

C	-2.868494	1.076532	-3.318510

C	-4.241176	1.368870	-3.360928

C	-1.986113	1.592261	-4.284471

C	-4.739032	2.178977	-4.393882

H	-4.894473	0.969479	-2.578402

C	-2.501066	2.392560	-5.315538

H	-0.915836	1.376157	-4.216031

C	-3.874306	2.687264	-5.376935

H	-5.809745	2.409729	-4.428507

H	-1.817601	2.798555	-6.069919

H	-4.266346	3.315831	-6.183834

C	2.054666	-3.292696	-0.494861

C	3.228337	-2.797712	-1.083171

C	1.965098	-4.601042	-0.001291

C	4.337375	-3.652422	-1.190525

H	3.266358	-1.759458	-1.424804

C	3.085806	-5.441588	-0.114721

H	1.038395	-4.931712	0.475429

C	4.268478	-4.972678	-0.711420

H	5.261552	-3.276424	-1.643732

H	3.031905	-6.466833	0.268798

H	5.138644	-5.633027	-0.796429

C	-4.255113	-2.520561	-0.222830

C	-4.595468	-3.445016	0.776909

C	-4.880660	-2.527481	-1.478400

C	-5.593806	-4.393795	0.500176

H	-4.083597	-3.417734	1.745184

C	-5.874394	-3.485102	-1.738694

H	-4.573808	-1.798924	-2.235096

C	-6.233712	-4.417302	-0.751010

H	-5.867951	-5.123021	1.271052

H	-6.364649	-3.502867	-2.718688

H	-7.008102	-5.164410	-0.957948

C	-1.352055	3.311025	2.877562

C	-1.721494	3.090089	4.216399

C	-0.841286	4.549712	2.459614

C	-1.589815	4.138480	5.140604

H	-2.105869	2.111991	4.522781

C	-0.706803	5.584775	3.398177

H	-0.543967	4.674291	1.413399

C	-1.084316	5.386018	4.736717

H	-1.876749	3.973251	6.185434

H	-0.303323	6.551873	3.077894

H	-0.979445	6.197715	5.464747

**TS-Isom-2**

SCF Done: -3655.34271854 A.U.

C	2.013358	-0.785386	0.478333

C	2.180107	0.197345	-0.532390

C	3.336129	0.241699	-1.337806

H	3.427832	1.016044	-2.103944

C	4.334426	-0.722069	-1.161667

H	5.227854	-0.697612	-1.795493

C	4.184688	-1.748839	-0.217253

H	4.940518	-2.530919	-0.108301

C	3.025915	-1.780062	0.574258

C	1.014055	-1.316557	3.226928

C	2.177331	-0.783280	3.837422

C	2.482942	-1.013202	5.190107

H	3.406694	-0.605262	5.610201

C	1.591161	-1.747247	5.980745

H	1.822484	-1.928650	7.035928

C	0.407473	-2.253742	5.432308

H	-0.304757	-2.815766	6.042322

C	0.132762	-2.034073	4.071693

C	-0.379682	0.625398	1.727966

C	0.230096	1.842469	2.081744

H	1.323027	1.921588	2.090603

C	-0.549616	2.953895	2.427991

H	-0.074184	3.904357	2.691114

C	-1.952496	2.833658	2.436047

H	-2.569721	3.694959	2.719557

C	-2.588279	1.636209	2.079613

C	-1.812203	0.477255	1.707399

C	-0.964328	-2.393294	-0.959991

Ni	-1.348453	-2.259082	0.863402

O	-2.409331	-0.648997	1.408982

P	0.469934	-0.971930	1.476718

C	0.336013	-3.017995	-1.471528

H	1.120679	-2.250990	-1.343078

C	0.248656	-3.436112	-2.946309

H	1.263432	-3.688298	-3.318844

H	0.607402	-3.860572	-0.817260

C	-0.638756	-4.660279	-3.194077

O	-1.019339	-5.385604	-2.277564

C	-1.020744	-4.933999	-4.642696

H	-1.447202	-5.944174	-4.738813

H	-1.780071	-4.193187	-4.959784

H	-0.156257	-4.817047	-5.321750

C	-1.650368	-4.311985	0.919790

C	-2.868140	-3.609069	0.926219

H	-1.320488	-4.836982	0.015390

H	-1.177258	-4.617925	1.861059

H	-3.463942	-3.511044	0.011551

H	-3.377312	-3.346826	1.861513

O	-1.829100	-1.948925	-1.686400

H	-0.103605	-2.596335	-3.573319

C	-4.069118	1.502211	2.125675

C	-4.827519	1.226247	0.971013

C	-4.776984	1.595345	3.337287

C	-6.218714	1.053883	1.018443

C	-6.165957	1.424727	3.414912

C	-6.891596	1.156602	2.245777

F	-4.107526	1.802999	4.500943

F	-6.800216	1.493598	4.606333

F	-8.228441	0.994460	2.300719

F	-6.918341	0.799808	-0.106699

F	-4.222942	1.133079	-0.227780

O	3.052993	-0.066899	3.028543

O	-1.030655	-2.540652	3.497804

O	2.749485	-2.822876	1.440976

O	1.106906	1.033439	-0.766247

C	3.724146	1.051902	3.516703

C	4.994738	1.294351	2.965484

C	3.150123	1.956110	4.429757

C	5.702495	2.444880	3.344688

H	5.401706	0.585328	2.236985

C	3.875677	3.098311	4.803225

H	2.149940	1.769860	4.831494

C	5.151030	3.347756	4.269136

H	6.693252	2.631846	2.915199

H	3.429741	3.803690	5.513616

H	5.708358	4.242211	4.567261

C	-2.183070	-2.580878	4.307920

C	-2.816679	-1.387375	4.688273

C	-2.715335	-3.833727	4.644998

C	-4.007596	-1.458586	5.426475

H	-2.396732	-0.422580	4.390457

C	-3.911164	-3.889386	5.382572

H	-2.198629	-4.744984	4.326906

C	-4.556365	-2.705579	5.775103

H	-4.515412	-0.529137	5.704867

H	-4.336432	-4.864040	5.647117

H	-5.490675	-2.753413	6.345043

C	3.774716	-3.653848	1.899974

C	3.630356	-5.033605	1.690982

C	4.859182	-3.137698	2.629801

C	4.592318	-5.910162	2.218328

H	2.767278	-5.402649	1.127788

C	5.817390	-4.025104	3.142687

H	4.930911	-2.059077	2.798569

C	5.689001	-5.410127	2.939393

H	4.482266	-6.988868	2.060402

H	6.664019	-3.629767	3.715427

H	6.438449	-6.097099	3.347119

C	1.293267	2.325855	-1.253680

C	0.342017	2.783233	-2.179609

C	2.314745	3.171565	-0.785376

C	0.422667	4.102436	-2.649579

H	-0.452197	2.104383	-2.505629

C	2.385996	4.485976	-1.272592

H	3.036265	2.805801	-0.047977

C	1.445868	4.956427	-2.204582

H	-0.321157	4.462212	-3.369299

H	3.179332	5.148923	-0.908648

H	1.506477	5.984887	-2.576571

**TS-2-Ins-E-T**

SCF Done: -3655.31686294 A.U.

C	2.921930	0.789541	2.888201

C	2.053272	1.050576	3.984946

C	2.514373	1.625805	5.182339

H	1.828029	1.767726	6.020557

C	3.855891	2.017419	5.273651

H	4.227323	2.456969	6.206047

C	4.724497	1.871289	4.185577

H	5.763634	2.204910	4.245898

C	4.248682	1.284274	2.998004

C	3.646468	-0.901753	0.510160

C	4.562380	-1.762736	1.166455

C	5.553433	-2.470805	0.465979

H	6.248692	-3.117529	1.007721

C	5.631517	-2.329605	-0.926761

H	6.406647	-2.870823	-1.480811

C	4.743192	-1.500280	-1.618559

H	4.808346	-1.385262	-2.703331

C	3.756404	-0.798740	-0.899028

C	1.239973	-1.487270	1.921653

C	1.417753	-2.299730	3.051365

H	2.210468	-2.066468	3.769293

C	0.574467	-3.402925	3.265855

H	0.708371	-4.038907	4.147422

C	-0.445612	-3.682252	2.339684

H	-1.108690	-4.538906	2.506068

C	-0.659517	-2.874737	1.208239

C	0.203906	-1.749530	0.968127

C	1.187043	2.786144	-0.007796

Ni	0.772838	0.767755	-0.137247

O	0.041778	-1.016094	-0.121229

P	2.246721	-0.054391	1.385433

C	1.191558	3.520385	1.334397

H	0.575637	2.979691	2.074039

C	0.663248	4.983006	1.198705

H	0.880171	5.516057	2.144614

H	2.235621	3.522671	1.692964

C	1.283142	5.738575	0.014306

O	0.618145	5.966639	-0.994655

C	2.746971	6.143630	0.123894

H	2.996785	6.862432	-0.671738

H	2.987924	6.568759	1.115178

H	3.365434	5.236214	-0.014412

C	-0.398113	2.541189	-0.615751

C	-0.577243	1.300949	-1.397888

H	-1.109453	2.654784	0.215929

H	-0.363672	3.465708	-1.225952

H	-1.457532	0.679805	-1.204582

H	-0.174201	1.265428	-2.420117

O	2.120574	2.909291	-0.817280

H	-0.429605	4.979928	1.047805

C	-1.750833	-3.181300	0.248257

C	-2.709310	-2.219390	-0.133692

C	-1.883706	-4.458849	-0.335132

C	-3.723907	-2.497525	-1.060187

C	-2.899203	-4.767730	-1.251881

C	-3.823698	-3.779841	-1.619559

F	-0.998919	-5.438120	-0.035504

F	-2.983565	-6.001741	-1.791820

F	-4.803199	-4.061641	-2.500583

F	-4.619201	-1.546431	-1.399617

F	-2.694136	-0.983027	0.410384

O	2.884481	0.069721	-1.532847

O	4.466401	-1.812315	2.546030

O	0.732149	0.699168	3.781156

O	5.059923	1.114578	1.892585

C	4.986427	-2.902261	3.246820

C	5.859621	-2.622103	4.309130

C	4.573020	-4.218006	2.972146

C	6.332648	-3.678489	5.104262

H	6.146114	-1.583524	4.502349

C	5.061544	-5.263725	3.770069

H	3.870201	-4.407430	2.155130

C	5.941144	-5.000100	4.834742

H	7.012962	-3.464025	5.936140

H	4.741246	-6.291103	3.562989

H	6.314506	-5.822034	5.455247

C	2.586422	-0.101103	-2.890559

C	2.683665	1.034657	-3.708825

C	2.106216	-1.324729	-3.388634

C	2.305877	0.934763	-5.058284

H	3.030080	1.974925	-3.270851

C	1.742501	-1.408960	-4.741518

H	2.010730	-2.185796	-2.720468

C	1.842451	-0.284607	-5.579986

H	2.376376	1.818251	-5.702877

H	1.364696	-2.358572	-5.137254

H	1.551305	-0.357552	-6.633535

C	-0.246632	0.953845	4.747162

C	-0.614372	2.267829	5.085541

C	-0.928441	-0.151535	5.277718

C	-1.673670	2.469646	5.984496

H	-0.075784	3.115038	4.648584

C	-1.991028	0.066494	6.169133

H	-0.628766	-1.158303	4.969089

C	-2.362925	1.372618	6.528640

H	-1.965899	3.491397	6.252249

H	-2.531744	-0.792538	6.581823

H	-3.192506	1.536630	7.224909

C	6.211231	1.880665	1.718068

C	6.189937	3.285977	1.775157

C	7.383127	1.190231	1.370686

C	7.365397	3.998545	1.493539

H	5.263094	3.807650	2.032917

C	8.548706	1.917316	1.082625

H	7.357920	0.097501	1.314422

C	8.546959	3.320280	1.148379

H	7.352519	5.093683	1.534932

H	9.463169	1.380170	0.806944

H	9.459384	3.883853	0.925624

**2-cycle5-C**

SCF Done: -3655.36785184 A.U.

C	-1.861909	-0.740601	-1.413999

C	-1.515909	-1.401438	-2.623765

C	-2.492992	-1.822290	-3.544705

H	-2.192722	-2.374604	-4.438960

C	-3.837842	-1.511626	-3.306390

H	-4.601267	-1.828547	-4.025684

C	-4.214650	-0.775345	-2.175612

H	-5.256243	-0.488118	-2.007620

C	-3.226801	-0.390019	-1.250097

C	-1.347239	-0.189599	1.463338

C	-1.947158	-1.326671	2.057601

C	-2.398835	-1.323243	3.388822

H	-2.871285	-2.216211	3.806441

C	-2.245296	-0.162003	4.155986

H	-2.596308	-0.150475	5.193826

C	-1.678518	0.993976	3.606623

H	-1.583663	1.910213	4.195218

C	-1.251718	0.975969	2.266743

C	0.744636	-1.459112	-0.042148

C	0.629630	-2.858272	-0.076586

H	-0.337596	-3.316031	-0.302707

C	1.751363	-3.668562	0.159413

H	1.662896	-4.759538	0.125673

C	2.983213	-3.066051	0.469156

H	3.856041	-3.692892	0.685601

C	3.129216	-1.669298	0.516403

C	2.006994	-0.827657	0.200063

C	0.041190	4.024902	-1.400273

Ni	0.846228	1.586664	-0.567829

O	2.183220	0.485158	0.139327

P	-0.562433	-0.191445	-0.221751

C	-0.847859	5.090514	-1.996875

H	-1.171365	5.747923	-1.162964

C	-2.083106	4.552988	-2.726014

H	-1.820318	3.710926	-3.393157

H	-0.232627	5.724552	-2.663630

C	-3.218846	4.112056	-1.794421

O	-3.242355	4.423594	-0.604472

C	-4.318806	3.286580	-2.442347

H	-4.540327	3.634083	-3.467771

H	-3.974792	2.237098	-2.520612

H	-5.230972	3.306437	-1.826771

C	1.390042	4.319260	-0.813494

C	2.170514	2.993681	-0.750993

H	1.893967	5.147691	-1.357270

H	1.206645	4.703500	0.213623

H	2.679016	2.759111	-1.705210

H	2.886976	2.935215	0.081243

O	-0.353478	2.824760	-1.359826

H	-2.501435	5.341957	-3.384527

C	4.432233	-1.042644	0.853184

C	5.616471	-1.356967	0.156346

C	4.548317	-0.093622	1.889979

C	6.848341	-0.757831	0.459082

C	5.763557	0.529733	2.203214

C	6.921469	0.194109	1.485877

F	3.473983	0.222240	2.645645

F	5.833270	1.434259	3.203733

F	8.098232	0.778739	1.786369

F	7.958323	-1.080193	-0.238130

F	5.590418	-2.248249	-0.860758

O	-0.770068	2.116096	1.647343

O	-2.145343	-2.424596	1.229946

O	-3.513050	0.462894	-0.197116

O	-0.170460	-1.648454	-2.804471

C	0.358939	-1.926326	-4.063583

C	0.099882	-1.115696	-5.183582

C	1.263024	-2.997802	-4.138316

C	0.745273	-1.403872	-6.396111

H	-0.592382	-0.272121	-5.099526

C	1.907229	-3.266575	-5.355671

H	1.459637	-3.587430	-3.237416

C	1.646935	-2.477815	-6.488678

H	0.547956	-0.774706	-7.271727

H	2.617631	-4.098720	-5.415414

H	2.150233	-2.692937	-7.437521

C	-4.749849	0.446337	0.443378

C	-5.428409	-0.746031	0.750313

C	-5.248392	1.693486	0.857988

C	-6.636561	-0.678030	1.461447

H	-5.003989	-1.707366	0.446157

C	-6.453683	1.740798	1.575114

H	-4.684029	2.601465	0.617383

C	-7.155670	0.560661	1.873879

H	-7.167994	-1.605475	1.704244

H	-6.845450	2.711231	1.900828

H	-8.097794	0.604737	2.431051

C	-0.251349	3.157066	2.422131

C	0.969650	2.997817	3.100163

C	-0.925357	4.388680	2.400048

C	1.508115	4.096495	3.788400

H	1.495965	2.039416	3.054423

C	-0.368011	5.479826	3.089726

H	-1.856208	4.478617	1.830351

C	0.842166	5.335552	3.789286

H	2.463029	3.984875	4.314354

H	-0.886138	6.445725	3.079545

H	1.271414	6.188649	4.326303

C	-2.177283	-3.711473	1.767730

C	-1.217967	-4.163172	2.692397

C	-3.157383	-4.581599	1.263338

C	-1.265137	-5.496575	3.126022

H	-0.444120	-3.478906	3.052152

C	-3.185430	-5.915643	1.699565

H	-3.877404	-4.206236	0.528994

C	-2.245920	-6.376422	2.636121

H	-0.517932	-5.851581	3.844878

H	-3.950070	-6.594661	1.305557

H	-2.272797	-7.416703	2.978112

**TS-1-Coor-E-T**

SCF Done: -3542.02407317 A.U.

C	-2.256951	3.087327	0.803976

C	-1.374629	4.159258	1.094211

C	-1.776183	5.271890	1.859728

H	-1.052207	6.063113	2.072835

C	-3.086410	5.338217	2.341091

H	-3.406229	6.202384	2.933858

C	-3.992783	4.297956	2.087159

H	-5.018862	4.343377	2.461515

C	-3.566966	3.182654	1.347491

C	-3.040732	1.166164	-1.335300

C	-3.817691	2.149429	-2.003910

C	-4.805847	1.809023	-2.945360

H	-5.408288	2.595322	-3.407950

C	-4.979431	0.465603	-3.298473

H	-5.728404	0.195655	-4.051292

C	-4.189756	-0.533337	-2.720117

H	-4.288513	-1.575722	-3.032377

C	-3.241481	-0.179551	-1.741605

C	-0.266938	1.894408	-1.181538

C	-0.246687	2.713991	-2.322157

H	-1.143164	3.274499	-2.609461

C	0.926654	2.830976	-3.080715

H	0.953947	3.474240	-3.966193

C	2.079269	2.132979	-2.677254

H	3.002498	2.235943	-3.259143

C	2.092491	1.316625	-1.533795

C	0.884288	1.147064	-0.764042

C	-1.150546	-2.461699	2.305442

Ni	-0.821662	0.095547	1.238121

O	0.840760	0.330457	0.259815

P	-1.697215	1.556598	-0.097774

C	-0.890151	-3.873586	2.773357

C	-2.519426	-1.843062	2.385789

C	-2.422357	-0.309163	2.274695

H	-3.076956	-2.260404	1.519705

H	-3.070397	-2.202716	3.282260

H	-3.370581	0.115221	1.914416

H	-2.183953	0.143653	3.256301

O	-0.228333	-1.780347	1.803641

C	3.322272	0.595845	-1.121373

C	3.844240	0.697867	0.184886

C	4.032928	-0.236195	-2.011667

C	4.987001	-0.001968	0.595507

C	5.187566	-0.935634	-1.631293

C	5.665780	-0.821310	-0.318315

F	3.595399	-0.404384	-3.281124

F	5.831731	-1.728768	-2.514086

F	6.773390	-1.489554	0.061497

F	5.452381	0.124479	1.856565

F	3.261753	1.512787	1.094209

H	-1.581150	-4.572763	2.265538

H	0.150737	-4.164701	2.565119

H	-1.089024	-3.960190	3.858594

C	0.223239	1.651620	3.060870

C	0.842459	0.504689	3.417696

H	-0.663555	2.021074	3.589878

H	0.629049	2.294366	2.274659

H	0.468413	-0.126050	4.233539

H	1.741154	0.164299	2.895127

O	-2.376154	-1.111891	-1.198542

O	-3.593998	3.467412	-1.643176

O	-0.058150	4.023542	0.692702

O	-4.427466	2.141217	1.032764

C	-2.662094	-2.471913	-1.210042

C	-1.585013	-3.327219	-1.498622

C	-3.918313	-2.991902	-0.840262

C	-1.772075	-4.716151	-1.426186

H	-0.618143	-2.888737	-1.763698

C	-4.090455	-4.384779	-0.780629

H	-4.745124	-2.313397	-0.606751

C	-3.023056	-5.251272	-1.072300

H	-0.932289	-5.382704	-1.652553

H	-5.067843	-4.791481	-0.496492

H	-3.165627	-6.336249	-1.024265

C	-3.891352	4.520423	-2.505476

C	-3.537919	4.511017	-3.867750

C	-4.468703	5.658203	-1.917303

C	-3.782948	5.655418	-4.642608

H	-3.077888	3.621577	-4.309437

C	-4.699998	6.796558	-2.704830

H	-4.714794	5.635060	-0.850834

C	-4.364220	6.798917	-4.068985

H	-3.506854	5.651945	-5.703165

H	-5.149283	7.684886	-2.246627

H	-4.549649	7.688022	-4.681215

C	-5.607748	1.944265	1.749164

C	-5.610644	1.776749	3.145865

C	-6.788747	1.810307	1.001937

C	-6.820729	1.485843	3.794421

H	-4.676119	1.870449	3.707367

C	-7.989756	1.510083	1.664159

H	-6.745481	1.933022	-0.084889

C	-8.011662	1.352120	3.059732

H	-6.828316	1.354088	4.882452

H	-8.912930	1.403568	1.083340

H	-8.951408	1.121876	3.573329

C	0.666361	5.107275	0.202977

C	2.059764	5.034812	0.372380

C	0.076546	6.176527	-0.494506

C	2.865645	6.059082	-0.143945

H	2.491712	4.165638	0.877509

C	0.899563	7.196529	-0.998012

H	-1.006451	6.204809	-0.647458

C	2.291951	7.148183	-0.822368

H	3.952359	6.002031	-0.013991

H	0.441912	8.030660	-1.542512

H	2.926345	7.948151	-1.219199

**1-Coor-E-T**

SCF Done: -3542.02986815 A.U.

C	-2.048146	1.234183	0.782185

C	-1.757468	2.530068	1.286434

C	-2.760265	3.368402	1.807115

H	-2.494994	4.356350	2.192057

C	-4.078877	2.902711	1.850129

H	-4.866336	3.547217	2.256241

C	-4.402843	1.609534	1.418695

H	-5.426407	1.233768	1.489132

C	-3.387826	0.773916	0.920959

C	-1.360421	-1.005063	-1.105453

C	-2.192637	-0.596393	-2.177968

C	-2.588253	-1.483436	-3.193015

H	-3.240824	-1.136361	-3.998387

C	-2.130854	-2.807204	-3.153463

H	-2.433529	-3.505978	-3.941162

C	-1.301283	-3.253760	-2.119167

H	-0.948420	-4.287464	-2.087263

C	-0.926372	-2.352487	-1.105958

C	0.485188	1.155674	-0.853112

C	0.188526	2.178060	-1.767868

H	-0.839849	2.539958	-1.866518

C	1.211822	2.737111	-2.549443

H	0.991856	3.546417	-3.253235

C	2.527145	2.265087	-2.401630

H	3.331527	2.707635	-3.000690

C	2.852516	1.252671	-1.481771

C	1.815873	0.653916	-0.684217

C	-1.945018	-0.986041	4.877818

Ni	0.745882	-0.926348	1.482401

O	2.086581	-0.331905	0.150661

P	-0.682122	0.174043	0.154043

C	-1.245480	-1.410605	6.165901

C	-1.061177	-0.365172	3.790058

C	-0.608233	-1.464093	2.807062

H	-1.652253	0.401905	3.262489

H	-0.190775	0.129403	4.260446

H	-0.140089	-2.308120	3.349971

H	-1.478709	-1.857746	2.253669

O	-3.153586	-1.165614	4.722081

C	4.253898	0.784535	-1.327641

C	4.912592	0.808237	-0.081393

C	4.999165	0.296152	-2.419978

C	6.226809	0.349241	0.082295

C	6.320518	-0.156348	-2.287104

C	6.936659	-0.132931	-1.027754

F	4.437705	0.229448	-3.649062

F	6.996227	-0.625823	-3.356982

F	8.204295	-0.566759	-0.885229

F	6.822130	0.389155	1.292697

F	4.291389	1.315548	1.006577

H	-0.919241	-0.510760	6.722822

H	-1.926791	-2.000540	6.798257

H	-0.332628	-1.994738	5.945500

C	2.255141	-0.822208	2.930467

C	2.289696	-2.070809	2.321000

H	1.808277	-0.686656	3.921665

H	2.870119	-0.004633	2.541827

H	1.862409	-2.958815	2.800113

H	2.921129	-2.238856	1.442428

O	-0.133822	-2.762742	-0.038713

O	-2.639176	0.712711	-2.132236

O	-3.628373	-0.522676	0.522548

O	-0.419378	2.873432	1.343618

C	-3.097748	1.340344	-3.292782

C	-2.284091	1.460260	-4.433496

C	-4.364637	1.941502	-3.236874

C	-2.764570	2.182120	-5.536829

H	-1.288874	1.005064	-4.441611

C	-4.827614	2.667665	-4.346073

H	-4.961433	1.841676	-2.324652

C	-4.033892	2.785428	-5.498839

H	-2.134992	2.281517	-6.428280

H	-5.815351	3.140486	-4.306385

H	-4.399621	3.350248	-6.363213

C	0.671610	-3.901212	-0.207917

C	0.300866	-5.088889	0.438703

C	1.863508	-3.803252	-0.943452

C	1.144653	-6.208716	0.337729

H	-0.633089	-5.123423	1.008777

C	2.693321	-4.931432	-1.038400

H	2.133725	-2.846986	-1.401603

C	2.336544	-6.133798	-0.401931

H	0.865707	-7.141871	0.839958

H	3.628873	-4.865915	-1.605188

H	2.990688	-7.009215	-0.477641

C	-0.015498	4.206027	1.279702

C	1.047441	4.576682	2.118802

C	-0.569245	5.121432	0.367153

C	1.557001	5.881779	2.046492

H	1.463934	3.836280	2.808917

C	-0.054648	6.425925	0.313568

H	-1.387395	4.812540	-0.290385

C	1.006345	6.812313	1.149561

H	2.388540	6.170835	2.698967

H	-0.483131	7.141375	-0.397640

H	1.404714	7.831340	1.098420

C	-4.864415	-1.122993	0.808075

C	-5.708129	-1.427188	-0.269942

C	-5.184045	-1.489390	2.125307

C	-6.907514	-2.115080	-0.021547

H	-5.410183	-1.139034	-1.282993

C	-6.390471	-2.170498	2.354850

H	-4.494349	-1.255014	2.946303

C	-7.252642	-2.483614	1.289213

H	-7.571479	-2.362726	-0.857560

H	-6.650070	-2.465302	3.377942

H	-8.189151	-3.019728	1.479510

**TS-Isom-1**

SCF Done: -3542.0116809 A.U.

C	3.190264	0.970007	-2.673247

C	3.328157	-0.236536	-1.934746

C	4.350696	-0.401754	-0.981855

H	4.460016	-1.359860	-0.468137

C	5.203103	0.668880	-0.688051

H	5.987385	0.542462	0.066428

C	5.041320	1.907612	-1.319025

H	5.665750	2.765433	-1.056487

C	4.037319	2.045268	-2.291699

C	2.870409	2.140539	-5.355802

C	4.081251	1.607747	-5.870104

C	4.718695	2.154369	-6.997124

H	5.659653	1.725009	-7.351050

C	4.126522	3.239425	-7.653938

H	4.611518	3.666536	-8.538584

C	2.932155	3.796043	-7.185176

H	2.472998	4.649675	-7.690004

C	2.324952	3.257054	-6.036096

C	1.350278	-0.207071	-4.857844

C	2.062095	-1.383404	-5.151256

H	3.081451	-1.509242	-4.777602

C	1.464099	-2.402761	-5.906545

H	2.014592	-3.323494	-6.127125

C	0.152169	-2.229690	-6.384080

H	-0.321380	-3.024302	-6.973472

C	-0.568331	-1.051222	-6.139578

C	0.018003	0.007986	-5.358309

C	0.060666	1.872567	-1.616498

Ni	-0.083333	2.244934	-3.530291

O	-0.661574	1.109696	-5.103864

P	1.930126	1.297613	-3.986791

C	-0.592605	0.496808	-1.537528

H	-1.577420	0.483189	-2.045574

H	0.014011	-0.273414	-2.058653

C	-1.834135	-1.124224	0.063956

C	-0.803301	4.133226	-3.081277

C	-1.845467	3.277438	-3.460372

H	-0.635180	4.406459	-2.034559

H	-0.321847	4.782523	-3.819942

H	-2.499777	2.817845	-2.708573

H	-2.196702	3.236459	-4.497671

C	-1.956420	-0.882843	-6.650235

C	-3.018881	-1.686767	-6.195229

C	-2.279547	0.104476	-7.598657

C	-4.336917	-1.512230	-6.645644

C	-3.587741	0.315659	-8.052417

C	-4.623266	-0.502505	-7.576118

F	-1.303867	0.882803	-8.121889

F	-3.853832	1.285963	-8.954334

F	-5.884954	-0.320779	-8.011552

F	-5.331077	-2.297333	-6.181137

F	-2.793408	-2.650267	-5.274391

O	4.643253	0.553651	-5.163081

O	1.180273	3.823683	-5.500594

O	3.753114	3.270881	-2.871479

O	2.441622	-1.253233	-2.236884

C	5.495742	-0.350533	-5.799280

C	6.677289	-0.687417	-5.119944

C	5.148069	-0.981581	-7.007496

C	7.528691	-1.661703	-5.664788

H	6.904676	-0.192581	-4.170273

C	6.014290	-1.946807	-7.543324

H	4.208803	-0.724163	-7.506108

C	7.204943	-2.289097	-6.878977

H	8.450822	-1.927364	-5.135737

H	5.747610	-2.442072	-8.483871

H	7.873505	-3.045906	-7.303366

C	0.334128	4.549554	-6.358359

C	-0.609820	3.848970	-7.122541

C	0.386848	5.951752	-6.354075

C	-1.515101	4.574679	-7.912362

H	-0.641325	2.758269	-7.061897

C	-0.527340	6.665032	-7.149793

H	1.131777	6.465583	-5.737286

C	-1.474599	5.980300	-7.929894

H	-2.259076	4.029281	-8.503926

H	-0.497915	7.760517	-7.155252

H	-2.185843	6.542765	-8.544811

C	4.755969	4.228706	-3.021365

C	4.449280	5.539818	-2.625266

C	5.987945	3.921235	-3.624896

C	5.393637	6.557165	-2.834765

H	3.478735	5.743116	-2.161686

C	6.924475	4.947498	-3.820025

H	6.195505	2.894534	-3.941258

C	6.633380	6.265396	-3.427350

H	5.158240	7.582049	-2.526383

H	7.885268	4.714455	-4.293027

H	7.368903	7.061486	-3.585733

C	2.307122	-2.357412	-1.387630

C	1.953670	-2.208395	-0.034536

C	2.432320	-3.627471	-1.971029

C	1.740286	-3.359534	0.740461

H	1.834060	-1.210357	0.397942

C	2.207255	-4.768045	-1.183793

H	2.683856	-3.703761	-3.032999

C	1.867667	-4.639079	0.172955

H	1.462767	-3.248850	1.794756

H	2.299499	-5.761438	-1.637040

H	1.695680	-5.531518	0.784516

H	1.102804	1.882522	-1.264096

H	-0.517351	2.617913	-1.046382

C	-0.791056	-0.021206	-0.095671

O	-0.150787	0.412319	0.858146

H	-2.844335	-0.714037	-0.126914

H	-1.670967	-1.929522	-0.675602

H	-1.796583	-1.535515	1.084441

**1-Coor-E-C**

SCF Done: -3542.02479117 A.U.

C	-1.875400	0.991241	0.669649

C	-2.382074	2.305713	0.519476

C	-3.039654	2.974674	1.566284

H	-3.441155	3.978415	1.401261

C	-3.159703	2.345537	2.812290

H	-3.668384	2.863139	3.632934

C	-2.646529	1.059740	3.016270

H	-2.731331	0.561571	3.986045

C	-2.017469	0.396322	1.948095

C	-1.561322	0.495067	-2.262509

C	-2.963208	0.462881	-2.492232

C	-3.520617	0.720466	-3.754268

H	-4.605201	0.693691	-3.887077

C	-2.664670	0.975285	-4.836176

H	-3.090096	1.157803	-5.829163

C	-1.276108	0.950241	-4.673136

H	-0.603989	1.100943	-5.522043

C	-0.735328	0.696220	-3.397520

C	0.768188	0.789694	-0.315849

C	1.141813	2.135765	-0.471442

H	0.451117	2.838617	-0.950049

C	2.390456	2.581616	-0.017477

H	2.691296	3.625399	-0.153587

C	3.252386	1.671344	0.619701

H	4.223644	2.014080	0.996442

C	2.906887	0.320212	0.783089

C	1.643059	-0.163929	0.296141

Ni	-0.374028	-2.165478	-0.097040

O	1.320295	-1.437289	0.458721

P	-0.882762	0.062886	-0.601460

C	0.255622	-3.896084	0.612293

H	0.161209	-3.630623	1.682737

C	1.685472	-4.257609	0.251341

H	2.169065	-4.847919	1.064236

H	-0.452147	-4.700956	0.351608

C	1.886148	-5.035722	-1.041719

O	0.975893	-5.296003	-1.830313

H	2.298927	-3.337414	0.181310

C	3.318183	-5.499470	-1.312646

H	4.055314	-4.733812	-1.013452

H	3.529379	-6.404591	-0.710121

H	3.437927	-5.756091	-2.376986

C	-2.237612	-2.949449	-0.468314

C	-1.398173	-3.117012	-1.578762

H	-2.966848	-2.133024	-0.431510

H	-2.399506	-3.768311	0.242682

H	-1.443568	-2.405457	-2.413501

H	-0.860504	-4.053744	-1.768322

C	3.819603	-0.614112	1.497306

C	4.435523	-1.702698	0.852724

C	4.098722	-0.459701	2.868140

C	5.273545	-2.599682	1.528726

C	4.937309	-1.337261	3.570675

C	5.531675	-2.413138	2.895179

F	4.219654	-1.921217	-0.463961

F	5.836664	-3.637544	0.872468

F	6.338380	-3.265592	3.555166

F	5.160919	-1.163046	4.889945

F	3.514750	0.542430	3.570421

O	0.624215	0.530254	-3.213245

O	-3.740121	0.108496	-1.399458

O	-1.538345	-0.899079	2.105101

O	-2.258443	2.884035	-0.738779

C	-0.967323	-1.246127	3.339805

C	-1.564376	-2.278791	4.077505

C	0.225858	-0.638465	3.764843

C	-0.955439	-2.704731	5.270845

H	-2.488735	-2.736659	3.710963

C	0.821466	-1.073241	4.958300

H	0.692243	0.141983	3.156196

C	0.233465	-2.102953	5.714436

H	-1.415226	-3.511885	5.852290

H	1.764133	-0.616448	5.278167

H	0.707011	-2.441595	6.642421

C	-2.075660	4.257458	-0.886006

C	-1.279488	5.024377	-0.014855

C	-2.653039	4.835112	-2.030274

C	-1.085847	6.386935	-0.291805

H	-0.817938	4.557864	0.860441

C	-2.443131	6.196747	-2.295397

H	-3.244255	4.204498	-2.702390

C	-1.665559	6.979895	-1.425936

H	-0.466396	6.985744	0.385553

H	-2.894337	6.646019	-3.187283

H	-1.507882	8.043727	-1.633111

C	-5.117737	0.351655	-1.416449

C	-5.982556	-0.752009	-1.379769

C	-5.611076	1.666338	-1.376258

C	-7.367690	-0.532544	-1.301143

H	-5.564559	-1.763309	-1.410161

C	-6.997457	1.870744	-1.305102

H	-4.908063	2.504662	-1.386616

C	-7.877992	0.775495	-1.267180

H	-8.048457	-1.390549	-1.269230

H	-7.390038	2.893282	-1.268785

H	-8.959015	0.942006	-1.206836

C	1.538805	1.258282	-3.971039

C	1.374339	2.629215	-4.238844

C	2.693070	0.574024	-4.384657

C	2.378601	3.310392	-4.943916

H	0.475027	3.150628	-3.896304

C	3.691349	1.271748	-5.080813

H	2.793686	-0.489948	-4.148473

C	3.537526	2.638566	-5.367757

H	2.254646	4.379173	-5.153123

H	4.593447	0.739445	-5.402799

H	4.317984	3.178367	-5.914880

**TS-1-Ins-E-C**

SCF Done: -3542.00875381 A.U.

C	-2.872641	2.381863	-2.054533

C	-2.027300	3.308011	-2.723814

C	-2.523897	4.497932	-3.286487

H	-1.846347	5.167037	-3.823062

C	-3.881452	4.806850	-3.141794

H	-4.277327	5.726695	-3.586241

C	-4.737835	3.958884	-2.428812

H	-5.793423	4.207959	-2.291296

C	-4.224793	2.772189	-1.874355

C	-3.474327	-0.456251	-1.277935

C	-4.268279	-0.775796	-2.410589

C	-5.186876	-1.838574	-2.403681

H	-5.789287	-2.045003	-3.292081

C	-5.313489	-2.621309	-1.247789

H	-6.027083	-3.452656	-1.234372

C	-4.554105	-2.344506	-0.107215

H	-4.660150	-2.942978	0.801470

C	-3.650842	-1.264564	-0.127492

C	-0.890142	0.113515	-2.420083

C	-0.962111	-0.102124	-3.806114

H	-1.833686	0.248048	-4.366504

C	0.087908	-0.751105	-4.473948

H	0.036568	-0.919972	-5.554768

C	1.216612	-1.166539	-3.743551

H	2.047615	-1.660633	-4.261449

C	1.326525	-0.935788	-2.363331

C	0.252848	-0.290385	-1.653913

Ni	-1.033775	1.017366	0.533766

O	0.327292	-0.090500	-0.352931

P	-2.149788	0.847597	-1.315160

C	0.260436	0.887823	2.246715

H	0.205532	0.968298	3.340943

C	1.559141	1.508273	1.739062

H	1.652212	1.406814	0.643517

H	0.203397	-0.185586	1.991396

C	1.881235	2.941464	2.136523

O	1.211239	3.602942	2.932315

H	2.411995	0.904611	2.130914

C	3.118277	3.533443	1.461300

H	2.805859	3.992465	0.502914

H	3.555938	4.323152	2.092764

H	3.870983	2.763954	1.217475

C	-2.308647	2.288528	1.257135

C	-1.459412	2.019959	2.389829

H	-2.194113	3.244289	0.725055

H	-3.327220	1.879339	1.252026

H	-0.829878	2.830987	2.774296

H	-1.885703	1.374022	3.167953

C	2.553939	-1.322245	-1.617407

C	2.968105	-2.661385	-1.488209

C	3.365066	-0.352441	-0.997426

C	4.114907	-3.024729	-0.764414

C	4.501254	-0.686802	-0.249906

C	4.882964	-2.032441	-0.137702

F	2.242022	-3.652540	-2.051819

F	4.475767	-4.320331	-0.655127

F	5.980732	-2.369262	0.566793

F	5.240199	0.276622	0.345324

F	3.064149	0.960508	-1.122986

O	-2.942338	-0.920595	1.015339

O	-4.126814	0.060640	-3.504510

O	-0.697792	2.950211	-2.817697

O	-5.031289	1.898399	-1.164349

C	-2.610670	-1.926310	1.930069

C	-3.095985	-1.809547	3.241479

C	-1.735850	-2.963950	1.564568

C	-2.697408	-2.752491	4.204138

H	-3.775490	-0.988158	3.491052

C	-1.352809	-3.902137	2.535145

H	-1.355588	-3.016187	0.539750

C	-1.830216	-3.800357	3.854103

H	-3.070970	-2.666088	5.230728

H	-0.665995	-4.710388	2.259711

H	-1.522203	-4.533484	4.607454

C	-4.550535	-0.355622	-4.769112

C	-4.031166	-1.515459	-5.371572

C	-5.433490	0.488813	-5.459097

C	-4.421746	-1.833408	-6.681461

H	-3.325841	-2.145022	-4.820090

C	-5.808162	0.161447	-6.772144

H	-5.802989	1.391668	-4.962939

C	-5.309460	-1.000229	-7.384274

H	-4.019355	-2.734562	-7.157836

H	-6.495366	0.819093	-7.316183

H	-5.606145	-1.253262	-8.407929

C	0.287674	3.907017	-3.061170

C	0.435132	5.043521	-2.245655

C	1.199632	3.628472	-4.090662

C	1.507883	5.916908	-2.484049

H	-0.279357	5.233318	-1.438333

C	2.271929	4.507300	-4.310846

H	1.063664	2.721110	-4.687279

C	2.427085	5.654250	-3.514846

H	1.628380	6.804206	-1.852052

H	2.989299	4.291074	-5.110459

H	3.264176	6.338276	-3.691648

C	-6.156726	2.358760	-0.482411

C	-6.110739	3.471932	0.376797

C	-7.325449	1.590609	-0.608304

C	-7.260061	3.818974	1.103440

H	-5.186883	4.050174	0.474331

C	-8.463744	1.945918	0.131978

H	-7.319282	0.720461	-1.272202

C	-8.438323	3.062029	0.984533

H	-7.227668	4.684210	1.775355

H	-9.375866	1.345989	0.037026

H	-9.329879	3.338393	1.557723

**1-beta-T**

SCF Done: -3542.04565836 A.U.

C	3.069322	0.343181	2.673538

C	2.278068	0.869440	3.735125

C	2.858765	1.514301	4.842547

H	2.217382	1.898129	5.639945

C	4.248425	1.678944	4.890299

H	4.704556	2.177222	5.752833

C	5.056821	1.236588	3.836232

H	6.140701	1.383329	3.850014

C	4.460882	0.603137	2.731506

C	3.408013	-1.562555	0.358318

C	4.183553	-2.547036	1.025323

C	5.041107	-3.415697	0.331790

H	5.635383	-4.150258	0.881411

C	5.118256	-3.323522	-1.065880

H	5.774666	-4.007239	-1.615647

C	4.359909	-2.381672	-1.766807

H	4.400666	-2.320739	-2.857615

C	3.515353	-1.508913	-1.053075

C	0.891569	-1.586636	1.841573

C	0.962586	-2.538113	2.871980

H	1.879659	-2.635056	3.460996

C	-0.143822	-3.356202	3.151035

H	-0.097634	-4.093357	3.959525

C	-1.320354	-3.207050	2.396822

H	-2.189162	-3.836849	2.620377

C	-1.428613	-2.247360	1.375234

C	-0.302464	-1.408328	1.061346

Ni	1.040080	0.758668	-0.014697

O	-0.375035	-0.520668	0.088755

P	2.214217	-0.462315	1.249373

C	-0.234309	3.555601	0.376482

H	0.002429	3.158953	1.379830

C	-1.739057	3.382932	0.130725

H	-1.974908	2.305691	-0.002573

H	0.033241	4.628963	0.395135

C	-2.622814	3.868335	1.282113

O	-2.150222	4.263333	2.347849

H	-2.067476	3.874585	-0.808166

C	-4.127106	3.786406	1.052055

C	2.005626	2.392852	-0.172631

C	0.641448	2.823345	-0.659936

H	2.331581	2.837834	0.780247

H	2.808253	2.368613	-0.927074

H	0.675200	3.339193	-1.640862

H	0.032547	1.875349	-0.986492

C	-2.689026	-2.089027	0.606016

C	-3.302284	-3.179959	-0.045127

C	-3.340105	-0.844300	0.478287

C	-4.484404	-3.048557	-0.789200

C	-4.511905	-0.683747	-0.272477

C	-5.092429	-1.790709	-0.907671

F	-2.740498	-4.408435	0.014201

F	-5.031090	-4.118498	-1.403192

F	-6.226650	-1.647486	-1.620818

F	-5.105598	0.529847	-0.368818

F	-2.863177	0.248672	1.116039

H	-4.665994	4.117087	1.953147

H	-4.425990	4.413559	0.190843

H	-4.412296	2.747068	0.804605

O	2.686041	-0.627819	-1.730947

O	4.095927	-2.546461	2.405168

O	0.914148	0.786419	3.566260

O	5.245656	0.136784	1.686545

C	0.060946	0.962912	4.664943

C	0.068409	0.042794	5.726632

C	-0.852822	2.025237	4.622080

C	-0.855197	0.203248	6.770696

H	0.777547	-0.790733	5.717297

C	-1.776432	2.166318	5.671169

H	-0.851500	2.724749	3.779649

C	-1.778181	1.263469	6.746876

H	-0.859664	-0.512768	7.600486

H	-2.495981	2.991574	5.636472

H	-2.500780	1.379956	7.562171

C	6.236571	0.933911	1.126474

C	7.275468	0.241621	0.476916

C	6.195508	2.340787	1.117642

C	8.283689	0.965481	-0.176085

H	7.269126	-0.852992	0.489156

C	7.217501	3.048548	0.464810

H	5.376906	2.871056	1.612605

C	8.264472	2.370903	-0.181285

H	9.092450	0.423517	-0.679485

H	7.185228	4.144155	0.457995

H	9.057182	2.932959	-0.686577

C	4.531837	-3.658707	3.131838

C	3.909383	-4.910193	2.981556

C	5.537535	-3.454791	4.088214

C	4.318518	-5.974122	3.800201

H	3.111507	-5.033964	2.242478

C	5.930681	-4.527196	4.905389

H	5.988354	-2.462109	4.183514

C	5.327574	-5.787517	4.761184

H	3.836327	-6.952117	3.691528

H	6.713871	-4.374041	5.656288

H	5.638323	-6.621679	5.399610

C	3.132471	-0.015323	-2.901978

C	2.205284	0.084035	-3.952482

C	4.409699	0.566168	-3.001954

C	2.565318	0.773536	-5.120991

H	1.220016	-0.379367	-3.838227

C	4.755635	1.244835	-4.181172

H	5.116079	0.488829	-2.169123

C	3.840047	1.352297	-5.242145

H	1.844559	0.851579	-5.942896

H	5.748531	1.701630	-4.262841

H	4.117954	1.886458	-6.157131

**TS-1-Ins-E-T**

SCF Done: -3541.99308507 A.U.

C	2.965281	1.493007	2.137484

C	2.417650	2.566999	2.885832

C	3.229298	3.549991	3.485354

H	2.762710	4.362101	4.049486

C	4.617874	3.478110	3.334213

H	5.255307	4.235894	3.803088

C	5.202514	2.464579	2.561311

H	6.285358	2.419076	2.419682

C	4.375087	1.509618	1.947296

C	2.807178	-1.378103	1.413957

C	3.322063	-1.875856	2.638417

C	3.907956	-3.149614	2.740515

H	4.302350	-3.494198	3.700101

C	3.974565	-3.961450	1.599838

H	4.426241	-4.957150	1.671692

C	3.491562	-3.509419	0.367587

H	3.561973	-4.130900	-0.529060

C	2.928615	-2.222045	0.282379

C	0.349009	-0.063584	2.153375

C	0.155802	-0.298133	3.526258

H	1.004718	-0.228955	4.212268

C	-1.119951	-0.601638	4.020909

H	-1.273395	-0.766770	5.092336

C	-2.207498	-0.662683	3.132004

H	-3.209608	-0.885491	3.516140

C	-2.052450	-0.419127	1.757534

C	-0.743989	-0.132218	1.228776

Ni	1.035180	0.690930	-0.800989

O	-0.595763	0.033243	-0.073347

P	1.909595	0.245694	1.261150

C	2.654122	1.923481	-1.528082

H	3.302827	1.323221	-0.875096

C	2.171732	3.238951	-0.912173

H	2.780833	3.501297	-0.022787

H	3.249168	2.144447	-2.426280

C	2.210132	4.435293	-1.869511

O	2.660407	4.352299	-3.011842

H	1.137977	3.160528	-0.508260

C	1.633500	5.741637	-1.333105

H	1.970119	5.941904	-0.299768

H	1.912470	6.576989	-1.993686

H	0.529031	5.668045	-1.300827

C	1.379181	1.192776	-2.941567

C	0.117059	0.672472	-2.520075

H	1.393903	2.193615	-3.389397

H	2.085202	0.494818	-3.409606

H	-0.758173	1.330443	-2.455184

H	-0.098893	-0.398558	-2.622836

C	-3.216995	-0.470938	0.837192

C	-4.065413	-1.595898	0.778413

C	-3.537659	0.599411	-0.024311

C	-5.165814	-1.666018	-0.088477

C	-4.623207	0.548153	-0.910078

C	-5.444658	-0.588336	-0.940785

F	-3.820945	-2.673363	1.560423

F	-5.947411	-2.766479	-0.118018

F	-6.497661	-0.642086	-1.780264

F	-4.896501	1.594120	-1.718896

F	-2.811819	1.736398	-0.001673

O	2.528305	-1.704629	-0.941726

O	3.275624	-1.007319	3.717626

O	1.038591	2.676419	2.899427

O	4.881063	0.499254	1.144284

C	3.373680	-1.495562	5.021743

C	2.476609	-2.458955	5.517531

C	4.336124	-0.903464	5.854453

C	2.563507	-2.838137	6.865869

H	1.720386	-2.889905	4.853932

C	4.404876	-1.287559	7.203351

H	5.008362	-0.147756	5.435846

C	3.524719	-2.257194	7.711381

H	1.865840	-3.586342	7.258757

H	5.153954	-0.826974	7.857267

H	3.583282	-2.556220	8.763546

C	2.108935	-2.586947	-1.942729

C	0.931714	-3.338420	-1.783350

C	2.839798	-2.622216	-3.140237

C	0.491376	-4.143602	-2.844985

H	0.370100	-3.272041	-0.846408

C	2.382352	-3.427892	-4.197017

H	3.752779	-2.024298	-3.229064

C	1.212389	-4.191106	-4.051601

H	-0.429270	-4.726903	-2.731234

H	2.947141	-3.458564	-5.135589

H	0.858824	-4.817361	-4.877827

C	0.374033	3.191666	4.009726

C	-0.774847	3.955603	3.746903

C	0.762777	2.903442	5.330455

C	-1.536215	4.443344	4.819137

H	-1.061523	4.145156	2.707784

C	-0.005081	3.406437	6.392107

H	1.650110	2.291690	5.519887

C	-1.153152	4.177316	6.144497

H	-2.434378	5.036608	4.613754

H	0.295011	3.181765	7.422102

H	-1.748537	4.563103	6.978999

C	6.200793	0.541297	0.687477

C	6.646642	1.565192	-0.166490

C	7.032606	-0.537358	1.023882

C	7.952655	1.505903	-0.676838

H	5.974584	2.388598	-0.428066

C	8.334298	-0.587205	0.499416

H	6.643563	-1.326014	1.675570

C	8.799177	0.433530	-0.346030

H	8.305187	2.299510	-1.345281

H	8.986382	-1.429799	0.755458

H	9.815820	0.391561	-0.751716

**1-beta-C**

SCF Done: -3542.04028027 A.U.

C	-1.819187	-0.245651	0.693186

C	-2.844985	-0.937062	-0.008563

C	-4.066953	-1.277133	0.593131

H	-4.841586	-1.783893	0.012371

C	-4.260854	-0.976062	1.949283

H	-5.203787	-1.253426	2.433480

C	-3.255004	-0.355353	2.696873

H	-3.386328	-0.152809	3.763349

C	-2.045534	0.003760	2.069062

C	-0.797222	1.072940	-1.733327

C	-1.831730	2.043890	-1.746544

C	-2.248653	2.666766	-2.936237

H	-3.068489	3.389552	-2.916053

C	-1.592513	2.356716	-4.136330

H	-1.902460	2.852705	-5.062915

C	-0.536646	1.439225	-4.163291

H	-0.002029	1.217744	-5.090879

C	-0.155424	0.804308	-2.966749

C	0.836026	1.301941	0.668406

C	0.545846	2.623745	1.053630

H	-0.459574	3.025127	0.888740

C	1.527498	3.415499	1.662300

H	1.300708	4.440474	1.973254

C	2.813072	2.882228	1.865384

H	3.590119	3.502372	2.327090

C	3.143013	1.575480	1.471429

C	2.136827	0.736619	0.876638

Ni	1.212752	-1.516769	-0.409785

O	2.445744	-0.512509	0.559115

P	-0.255252	0.144825	-0.221223

C	0.186348	-4.573466	-0.516861

H	-0.797861	-4.656765	-1.011266

C	-0.044940	-4.275536	0.973185

H	-0.143209	-3.170402	1.108450

H	0.685548	-5.553693	-0.635812

C	-1.322321	-4.894593	1.546661

O	-2.287534	-5.185276	0.839372

H	0.821765	-4.559237	1.598742

C	-1.350726	-5.099266	3.055415

C	2.317814	-3.081005	-0.626460

C	1.037673	-3.505834	-1.233037

H	3.157472	-2.851861	-1.296511

H	2.612097	-3.491286	0.348599

H	0.262493	-2.553172	-1.291412

H	1.111596	-3.690590	-2.320549

C	4.512672	1.037362	1.675626

C	5.128702	1.037818	2.943622

C	5.265436	0.496433	0.612388

C	6.416187	0.522684	3.154938

C	6.548076	-0.037332	0.800293

C	7.128627	-0.021753	2.077116

F	4.469724	1.527472	4.019384

F	6.966950	0.534771	4.387580

F	8.365999	-0.521988	2.265675

F	7.237484	-0.547421	-0.242695

F	4.773611	0.502516	-0.642802

H	-0.634597	-5.894698	3.338857

H	-1.027896	-4.181195	3.581002

H	-2.361415	-5.386180	3.383434

O	0.936650	-0.049622	-2.927647

O	-2.445357	2.293649	-0.530183

O	-2.562162	-1.206774	-1.337215

O	-0.991836	0.517949	2.801251

C	-3.403336	-2.019226	-2.104900

C	-3.597241	-3.376866	-1.794864

C	-3.956811	-1.445828	-3.260984

C	-4.372327	-4.161189	-2.664903

H	-3.149812	-3.817420	-0.896118

C	-4.721263	-2.248274	-4.123925

H	-3.775765	-0.386207	-3.469369

C	-4.934343	-3.604554	-3.826853

H	-4.527480	-5.220313	-2.430381

H	-5.153855	-1.807090	-5.029083

H	-5.533481	-4.227279	-4.500281

C	-1.224425	1.384566	3.865795

C	-0.376193	1.248858	4.976990

C	-2.189879	2.406388	3.813911

C	-0.503289	2.141619	6.051360

H	0.377809	0.455554	4.976313

C	-2.309586	3.286027	4.900967

H	-2.830259	2.512062	2.932581

C	-1.472278	3.158724	6.022046

H	0.160976	2.038158	6.916756

H	-3.059289	4.084704	4.861912

H	-1.570227	3.851787	6.864641

C	-3.236202	3.428665	-0.346846

C	-2.728218	4.724208	-0.551069

C	-4.528968	3.227288	0.162446

C	-3.540156	5.828307	-0.249044

H	-1.712156	4.857041	-0.936358

C	-5.325756	4.342742	0.467913

H	-4.884504	2.203557	0.317783

C	-4.837607	5.643234	0.259343

H	-3.149370	6.840300	-0.403944

H	-6.334792	4.190614	0.867201

H	-5.463414	6.510493	0.496116

C	1.230601	-0.867432	-4.018480

C	0.239543	-1.607844	-4.689140

C	2.588115	-1.000417	-4.354244

C	0.624696	-2.481339	-5.718908

H	-0.812276	-1.503048	-4.403788

C	2.955732	-1.884188	-5.381102

H	3.331947	-0.415496	-3.804368

C	1.978632	-2.623607	-6.069126

H	-0.143273	-3.061591	-6.243163

H	4.014028	-1.990356	-5.644779

H	2.270262	-3.309745	-6.871555

# **6. Supplementary References**

1.	Göttker-Schnetmann I, Mecking S. A Practical Synthesis of [(tmeda)Ni(CH3)2], Isotopically Labeled [(tmeda)Ni(13CH3)2], and Neutral Chelated-Nickel Methyl Complexes. *Organometallics* 2020, **39**(18)**:** 3433–3440.

2.	Baur M, Lin F, Morgen TO, Odenwald L, Mecking S. Polyethylene materials with in-chain ketones from nonalternating catalytic copolymerization. *Science* 2021, **374**(6567)**:** 604–607.

3.	Wang C, Xia J, Zhang Y, Hu X, Jian Z. Photodegradable polar-functionalized polyethylenes. *National Science Review* 2023, **10**(7)**:** nwad039.

4.	Tang S, Seidel FW, Nozaki K. High Density Polyethylenes Bearing Isolated In-Chain Carbonyls. *Angewandte Chemie International Edition* 2021, **60**(51)**:** 26506–26510.

5.	Baur M, Mecking S. Polyethylenes with Combined In-Chain and Side-Chain Functional Groups from Catalytic Terpolymerization of Carbon Monoxide and Acrylate. *ACS Macro Letters* 2022, **11**(10)**:** 1207–1211.

6.	X-RED version 1.31 (2005) Stoe Data Reduction Program.

7.	Dolomanov OV, Bourhis LJ, Gildea RJ, Howard JAK, Puschmann H. OLEX2: a complete structure solution, refinement and analysis program. *Journal of Applied Crystallography* 2009, **42**(2)**:** 339–341.

8.	Sheldrick G. SHELXT - Integrated space-group and crystal-structure determination. *Acta Crystallographica Section A* 2015, **71**(1)**:** 3–8.

9.	Bourhis LJ, Dolomanov OV, Gildea RJ, Howard JAK, Puschmann H. The anatomy of a comprehensive constrained, restrained refinement program for the modern computing environment - Olex2 dissected. *Acta Crystallographica Section A* 2015, **71**(1)**:** 59–75.

10.	Spek A. Single-crystal structure validation with the program PLATON. *Journal of Applied Crystallography* 2003, **36**(1)**:** 7–13.

11.	Poater A, Cosenza B, Correa A, Giudice S, Ragone F, Scarano V*, et al.* Samb*V*ca: A Web Application for the Calculation of the Buried Volume of N-Heterocyclic Carbene Ligands. *European Journal of Inorganic Chemistry* 2009, **2009**(13)**:** 1759–1766.

12.	Falivene L, Cao Z, Petta A, Serra L, Poater A, Oliva R*, et al.* Towards the online computer-aided design of catalytic pockets. *Nature Chemistry* 2019, **11**(10)**:** 872–879.

13.	Poater A, Ragone F, Giudice S, Costabile C, Dorta R, Nolan SP*, et al.* Thermodynamics of N-heterocyclic carbene dimerization: The balance of sterics and electronics. *Organometallics* 2008, **27**(12)**:** 2679–2681.

14.	Poater A, Ragone F, Mariz R, Dorta R, Cavallo L. Comparing the enantioselective power of steric and electrostatic effects in transition-metal-catalyzed asymmetric synthesis. *Chemistry* 2010, **16**(48)**:** 14348–14353.

15.	Iberl S, Voccia M, Ritacco I, Odenwald L, Baur M, Falivene L*, et al.* Keto-Polyethylene Material from Pd(II)-Catalyzed Copolymerization with Continuous Carbon Monoxide Feed. *ACS Catalysis* 2025, **15**(10)**:** 8259–8267.

16.	Xin BS, Sato N, Tanna A, Oishi Y, Konishi Y, Shimizu F. Nickel Catalyzed Copolymerization of Ethylene and Alkyl Acrylates. *Journal of the American Chemical Society* 2017, **139**(10)**:** 3611–3614.

17.	Sheldrick G. Crystal structure refinement with SHELXL. *Acta Crystallographica Section C* 2015, **71**(1)**:** 3–8.

18.	Ortmann P, Wimmer FP, Mecking S. Long-Spaced Polyketones from ADMET Copolymerizations as Ideal Models for Ethylene/CO Copolymers. *ACS Macro Letters* 2015, **4**(7)**:** 704–707.

19.	Frisch MJ, Trucks GW, Schlegel HB, Scuseria GE, Robb MA, Cheeseman JR*, et al.* Gaussian 16 Rev. C.01. Wallingford, CT; 2016.

20.	Perdew JP. Density-functional approximation for the correlation energy of the inhomogeneous electron gas. *Physical Review B* 1986, **33**(12)**:** 8822–8824.

21.	Perdew JP. Erratum: Density-functional approximation for the correlation energy of the inhomogeneous electron gas. *Physical Review B* 1986, **34**(10)**:** 7406.

22.	Becke AD. Density-functional exchange-energy approximation with correct asymptotic behavior. *Physical Review A* 1988, **38**(6)**:** 3098–3100.

23.	Weigend F, Ahlrichs R. Balanced basis sets of split valence, triple zeta valence and quadruple zeta valence quality for H to Rn: Design and assessment of accuracy. *Physical Chemistry Chemical Physics* 2005, **7**(18)**:** 3297–3305.

24.	Häussermann U, Dolg M, Stoll H, Preuss H, Schwerdtfeger P, Pitzer RM. Accuracy of energy-adjusted quasirelativistic ab initio pseudopotentials. *Molecular Physics* 1993, **78**(5)**:** 1211–1224.

25.	Küchle W, Dolg M, Stoll H, Preuss H. Energy‐adjusted pseudopotentials for the actinides. Parameter sets and test calculations for thorium and thorium monoxide. *The Journal of Chemical Physics* 1994, **100**(10)**:** 7535–7542.

26.	Leininger T, Nicklass A, Stoll H, Dolg M, Schwerdtfeger P. The accuracy of the pseudopotential approximation. II. A comparison of various core sizes for indium pseudopotentials in calculations for spectroscopic constants of InH, InF, and InCl. *The Journal of Chemical Physics* 1996, **105**(3)**:** 1052–1059.

27.	Pracht P, Bohle F, Grimme S. Automated exploration of the low-energy chemical space with fast quantum chemical methods. *Physical Chemistry Chemical Physics* 2020, **22**(14)**:** 7169–7192.

28.	Pracht P, Grimme S, Bannwarth C, Bohle F, Ehlert S, Feldmann G*, et al.* CREST—A program for the exploration of low-energy molecular chemical space. *The Journal of Chemical Physics* 2024, **160**(11).

29.	Bannwarth C, Ehlert S, Grimme S. GFN2-xTB—An Accurate and Broadly Parametrized Self-Consistent Tight-Binding Quantum Chemical Method with Multipole Electrostatics and Density-Dependent Dispersion Contributions. *Journal of Chemical Theory and Computation* 2019, **15**(3)**:** 1652–1671.

30.	Zhao Y, Truhlar DG. The M06 suite of density functionals for main group thermochemistry, thermochemical kinetics, noncovalent interactions, excited states, and transition elements: two new functionals and systematic testing of four M06-class functionals and 12 other functionals. *Theoretical Chemistry Accounts* 2008, **120**(1)**:** 215–241.

31.	Schäfer A, Huber C, Ahlrichs R. Fully optimized contracted Gaussian basis sets of triple zeta valence quality for atoms Li to Kr. *The Journal of Chemical Physics* 1994, **100**(8)**:** 5829–5835.

32.	Tomasi J, Persico M. Molecular Interactions in Solution: An Overview of Methods Based on Continuous Distributions of the Solvent. *Chemical Reviews* 1994, **94**(7)**:** 2027–2094.

33.	Barone V, Cossi M. Quantum Calculation of Molecular Energies and Energy Gradients in Solution by a Conductor Solvent Model. *The Journal of Physical Chemistry A* 1998, **102**(11)**:** 1995–2001.

34.	Lu T, Chen F. Multiwfn: A Multifunctional Wavefunction Analyzer. *J. Comput. Chem.* **2012**, *33*, 580–592.

35.	Boto R A, Peccati F, Laplaza R, Quan C, Carbone A, Piquemal J-P, Maday Y, Contreras-García J. NCIPLOT4: Fast, Robust, and Quantitative Analysis of Noncovalent Interactions. *J. Chem. Theory Comput.* **2020**, *16*, 4150–4158.
